# Development of an immune-related gene signature applying Ridge method for improving immunotherapy responses and clinical outcomes in lung adenocarcinoma

**DOI:** 10.7717/peerj.19121

**Published:** 2025-05-08

**Authors:** Zhen Chen, Yongjun Zhang

**Affiliations:** 1 Department of Cardiothoracic Surgery, The First College of Clinical Medical Science, China Three Gorges University, Yichang, China; 2 Department of Cardiothoracic Surgery, Xiangyang Central Hospital, Xiangyang, China

**Keywords:** Lung adenocarcinoma, Immunotherapy, Molecular biology, Immunology, Computational biology and bioinformatics

## Abstract

**Background:**

Lung adenocarcinoma (LUAD) is a major cause of cancer mortality. Considering the critical role of tumor infiltrating lymphocytes in effective immunotherapy, this study was designed to screen molecular markers related to tumor infiltrating cells in LUAD, aiming to improve immunotherapy response during LUAD therapy.

**Methods:**

The ConsensusClusterPlus method was used for clustering immune molecular subtypes of LUAD. Immune cell infiltration and immunotherapeutic potential in each subtype was evaluated employing single-sample gene set enrichment analysis (ssGSEA), Tumor Immune Dysfunction and Exclusion (TIDE), and Immunophenoscore (IPS). Immune-related co-expression modules were classified by weighted gene co-expression network analysis (WGCNA) analysis. The sequencing data of immune-related genes were comprehensively analyzed by introducing a new computational framework and 10 machine learning algorithms (a total of 101 combinations) to determine the prognostic genes, which were further combined to develop an immune prognostic signature (IMMPS) using the stepCox and Ridge methods. The expression of the signature genes was validated by quantitative real-time PCR (qRT-PCR).

**Results:**

Samples from The Cancer Genome Atlas dataset (TCGA-LUAD) were divided into two subtypes (immunosuppressive subgroup C1 and immune-activated subgroup C2); notably, the C2 subgroup was more likely to benefit from immunotherapy (*p* < 0.05). An IMMPS developed based on seven immune infiltrating cell-related genes (*SEMA7A, EFHD2, CHST11, SLC24A4, MAL, JCHAIN*, and *SCARF1*) could accurately predict the overall survival of LUAD in five LUAD cohorts, with an average C-index higher than 0.69. LUAD patients with a low IMMPS value had a higher immune cell infiltration (*p* < 0.05). In addition, the IMMPS exhibited better prediction performance in comparison to 154 published gene signatures, suggesting that the IMMPS was an independent prognostic risk factor for evaluating the overall survival of LUAD patients. Since *BTNL9* was the most relevant immune checkpoint gene, *in vitro* experiment showed that the expression of the seven key genes (*SEMA7A, EFHD2, CHST11, SLC24A4, MAL, JCHAIN*, and *SCARF1*) in LUAD cell lines was consistent with that in normal lung epithelial cells after inhibiting *BTNL9* expression (*p* < 0.05).

**Conclusions:**

Our results contributed to a better understanding of immunological characteristics of LUAD. The IMMPS could serve as a promising tool for improving the clinical outcome of patients suffering from LUAD.

## Introduction

Lung cancer is one of the most lethal and widely diagnosed cancers in the world (Siegel, Miller & Jemal, 2019; Ding, Lv & Hua, 2022; Rice et al., 2016). Non-small cell lung cancer (NSCLC), which accounts for approximately 80% of all the cancer cases in the lung, could be mainly divided into lung squamous cell carcinoma (LUSC) and lung adenocarcinoma (LUAD) (Qian et al., 2023; Guo et al., 2021). Noticeably, the two types of NSCLC are increasingly regarded as separate clinical entities since they share distinctively different molecular features and prognosis (Tian, 2017). As indicated by previously published studies, the tumor microenvironment (TME) of LUAD is enriched with different types of immune cells related to clinical outcomes (Dai et al., 2019; Zheng, Hu & Yao, 2017). Immunotherapies based on immune checkpoint inhibitors (ICIs) for treating lung cancer has been proven to be effective (Chae et al., 2018; Zhou & Gao, 2022). Several clinical trials of neoadjuvant ICI therapy also demonstrated their efficacy in resectable lung cancer (Gao et al., 2020; Forde et al., 2018). Additionally, a variety of ICIs, including atezolizumab targeting PD-L1 and navulizumab targeting PD-1 (Malhotra, Jabbour & Aisner, 2017), have been approved by the Food and Drug Administration (FDA) as second-line therapies for NSCLC treatment. However, influenced by various biological and molecular characteristics of different NSCLC subtypes, only around 15% of NSCLC patients can benefit from taking ICI, whereas many NSCLC patients have poor clinical outcomes (Schoenfeld & Hellmann, 2020). Currently, we face a lack of accurately prognostic biomarkers for NSCLC, especially prognostic markers related to immune. Hence, analysis of the TME in LUAD to improve immunotherapy strategies has great clinical significance.

Immune infiltration in TME has been widely found to be related to cancer prognosis, including LUAD (Dickerson et al., 2023; Okcu et al., 2023; Tang et al., 2023). For instance, counts of two lymphocyte populations (CD8/CD45RO, CD3/CD45RO, and CD3/CD8) in the tumor core (CT) and invasive margins (IM) can serve as prognostic markers for colorectal cancer (Pagès et al., 2009). An internationally recognized approach for the risk evaluation of colon cancer is to determine the immune score for patients using the four density percentiles of CD3^+^ and CD8^+^ T cells in CT and IM (Pagès et al., 2018). These findings pointed to the potential to facilitate clinical decision-making process based on cancer immune infiltration (Fridman et al., 2012). Currently, characterization of molecular analyses have been employed to evaluate immune infiltration in cancer patients, such as ESTIMATE (Yoshihara et al., 2013), CIBERSORT (Chen et al., 2018), and TIMER analyses (Li et al., 2020), laying a solid foundation for further study of clinical cancer features and immune infiltration. TME subtypes have been previously classified (Bagaev et al., 2021) by transcriptome analysis, and four unique TME subtypes conserved in 20 different tumors were discovered. TME subtypes are also connected with patients’ response to immunotherapy in a range of cancers. Recently, potential immunotherapy benefit for colon cancer patients in different subtypes has been successfully predicted utilizing immune-infiltrating cell scores, and accordingly a consensus immune-associated lncRNA signature for predicting the clinical outcomes of colon cancer was developed (Liu et al., 2022). The above findings supported the efficacy of developing a TME-based molecular signature to optimize precision therapies for cancer patients.

Tumor heterogeneity with differences ranging from genotype to phenotype between individual patients is an important feature of cancers. Ideally, biomarkers are expected to reflect a broad spectrum of gene expressions in tumor tissues, which therefore require a combination of multiple genes to address the problem of heterogeneity (Koncina et al., 2020). A variety of prognostic signatures have been developed applying high-throughput data and bioinformatics algorithms (Zhao et al., 2023; Scortegagna et al., 2023; Pan et al., 2022). In the last 5 years, more than 154 prognostic gene models have been created and validated as candidate biomarkers for LUAD. However, limitations such as a lack of rigorous validation in different cohorts or inappropriate modelling also hinder the application of these features in clinical setting.

The current work developed a novel computational framework to determine tumor infiltration in LUAD and to discover immune-correlated genes by conducting comprehensive analysis based on immune-infiltrating cells in the TME. The potential importance of these genes as predictive biomarkers for LUAD prognosis and immunotherapy was also analyzed.

## Materials and Methods

### The acquisition of LUAD patient datasets

The transcriptional profiles of patients with LUAD and their clinical information were collected from The Cancer Genome Atlas (TCGA, https://portal.gdc.cancer.gov) and Gene Expression Omnibus (GEO, http://www.ncbi.nlm.nih.gov/geo) under the following criteria: (1) Complete data on overall survival (OS) and survival status, stage, gender, age; (2) IlluminaHiSeq platform or Affymetrix HG-U133_Plus 2.0 platform; and (3) Sample size larger than 200. After filtering, 1,327 LUAD patients from five datasets were included in this study. Specifically, GSE30219 dataset contained 83 patients (Rousseaux et al., 2013); GSE31210 dataset contained 226 patients (Okayama et al., 2012); GSE50081 dataset contained 127 patients from (Der et al., 2014); GSE72094 dataset contained 398 patients (Schabath et al., 2016); 493 patients were collected from TCGA-LUAD (https://xenabrowser.net/datapages/?cohort=GDC%20TCGA%20Lung%20Adenocarcinoma%20(LUAD)&removeHub=https%3A%2F%2Fxena.treehouse.gi.ucsc.edu%3A443). The TCGA-LUAD dataset served as a training dataset for analyzing the features of immune genes, while the other four datasets were independent test datasets for validation. Detailed clinical information of the patients from these five groups was shown in Table 1.

**Table 1:** Information on clinicopathologic characteristics of patients in five datasets (GSE30219, GSE31210, GSE50081, GSE72094, and TCGA-LUAD).

Variable	GSE30219, *N* = 83^1^	GSE31210, *N* = 226^1^	GSE50081, *N* = 127^1^	GSE72094, *N* = 398^1^	TCGA, *N* = 493^1^
**Age**	60 (55, 69)	61 (55, 65)	70 (63, 76)	70 (64, 76)	66 (59, 72)
**Gender**					
Female	18 (22%)	121 (54%)	62 (49%)	222 (56%)	264 (54%)
Male	65 (78%)	105 (46%)	65 (51%)	176 (44%)	229 (46%)
**Smoker**					
Current	0 (NA%)	0 (0%)	36 (31%)	0 (0%)	116 (24%)
Ever	0 (NA%)	111 (49%)	56 (49%)	300 (91%)	294 (61%)
Never	0 (NA%)	115 (51%)	23 (20%)	31 (9.4%)	69 (14%)
Unknown	83	0	12	67	14
**T Stage**					
T1	69 (83%)	0 (NA%)	43 (34%)	0 (NA%)	164 (33%)
T2	12 (14%)	0 (NA%)	82 (65%)	0 (NA%)	265 (54%)
T3	2 (2.4%)	0 (NA%)	2 (1.6%)	0 (NA%)	43 (8.8%)
T4	0 (0%)	0 (NA%)	0 (0%)	0 (NA%)	18 (3.7%)
Unknown	0	226	0	398	3
**N Stage**					
N0	80 (96%)	0 (NA%)	94 (74%)	0 (NA%)	319 (66%)
N1	3 (3.6%)	0 (NA%)	33 (26%)	0 (NA%)	93 (19%)
N2	0 (0%)	0 (NA%)	0 (0%)	0 (NA%)	68 (14%)
N3	0 (0%)	0 (NA%)	0 (0%)	0 (NA%)	2 (0.4%)
Unknown	0	226	0	398	11
**M Stage**					
M0	83 (100%)	0 (NA%)	127 (100%)	0 (NA%)	327 (93%)
M1	0 (0%)	0 (NA%)	0 (0%)	0 (NA%)	24 (6.8%)
Unknown	0	226	0	398	142
**Stage**					
I	0 (0%)	0 (0%)	0 (0%)	0 (0%)	265 (55%)
II	0 (0%)	0 (0%)	0 (0%)	0 (0%)	116 (24%)
III	0 (0%)	0 (0%)	0 (0%)	0 (0%)	79 (16%)
IV	0 (0%)	0 (0%)	0 (0%)	0 (0%)	25 (5.2%)
Stage I	69 (83%)	168 (74%)	92 (72%)	254 (65%)	0 (0%)
Stage II	13 (16%)	58 (26%)	35 (28%)	67 (17%)	0 (0%)
Stage III	1 (1.2%)	0 (0%)	0 (0%)	57 (15%)	0 (0%)
Stage IV	0 (0%)	0 (0%)	0 (0%)	15 (3.8%)	0 (0%)
Unknown	0	0	0	5	8
**Status**					
Alive	40 (48%)	191 (85%)	76 (60%)	285 (72%)	315 (64%)
Dead	43 (52%)	35 (15%)	51 (40%)	113 (28%)	178 (36%)
**OS.time**	2,070 (855, 3,435)	1,744 (1,246, 2,050)	1,595 (697, 2,097)	824 (540, 1,012)	669 (434, 1,148)

**Note:**

1 Median (IQR) or Frequency (%).

### Gene expression profiling data processing

Background correction, quantile normalization, and log-2 transformation of GEO microarray datasets were processed in the R package oligo (Carvalho & Irizarry, 2010), and the Robust Multi-array Average algorithm was used for normalizing all the microarray datasets. According to the annotation files of the microarrays, the median value of multiple probes mapping to the same gene was taken, while a probe was deleted when it was mapped to multiple genes. Subsequently, transcripts per kilobase million (TPM) was converted from the RNA-seq read count in TCGA-LUAD.

### Assessment of immune infiltrating cells

Marker genes for 28 types of highly reliable immune-infiltrating cells were collected from a previous study (Charoentong et al., 2017). Single-sample gene set enrichment analysis (ssGSEA) in the R package GSVA (Hänzelmann, Castelo & Guinney, 2013) was conducted for calculating the proportion of immune-infiltrating cells in each patient, followed by verifying the results by ESTIMATE (Yoshihara et al., 2013), CIBERPSORT (Newman et al., 2015), TIMER (Li et al., 2016), EPIC (https://gfellerlab.shinyapps.io/EPIC_1-1/) (Racle et al., 2017), and MCP-counter (Becht et al., 2016) algorithms.

### Classification of immune-related molecular subtypes

Patients in the TCGA-LUAD cohort were classified applying a clustering method of unsupervised resampling based on the components of 28 types of immune infiltrating cells. The R package ConsensusClusterPlus (Wilkerson & Hayes, 2010) was used to calculate immune infiltrating cell scores for individual samples as a data matrix. Specifically, 80% of the samples were randomly selected for each iteration, and the distance between samples was measured by Pearson correlation coefficient. This was process was repeated 1,000 times, where samples were partitioned into up to k (maximum k = 10) clusters using the partioning around medoids (PAM) algorithm. Subsequently, the optimal number of clusters was determined according to consensus score matrix, cumulative distribution function (CDF) curve and proportion of ambiguous clustering (Pagès et al., 2018) score. The significance of the clustering results was assessed using sigclust (Harrison et al., 2003).

### Screening tumor-infiltrating immune-related gene markers

A novel computational framework for a comprehensive analysis of immune-infiltrating cells in the TME was created to identify immune genes related to tumor infiltration. Multiple machine learning methods were used to establish an immunity prognostic signature (IMMPS). The median absolute deviation (MAD) (Okayama et al., 2012) of all genome-wide protein-coding genes was computed, and potentially deregulated genes were defined as having a MAD greater than top 50%. Weighted gene co-expression network analysis (WGCNA) (Langfelder & Horvath, 2008) was constructed and the appropriate soft threshold β was calculated to satisfy a scale-free network. Additionally, the weighted adjacency matrix was converted to topological overlap matrix (TOM) and modules were identified by the dynamic tree cutting method. Next, immune-related modules were recognized based on the correlation of each module with immune clusters. The relationship between the prognosis of LUAD and genes in the immune-related modules was analyzed by univariate Cox regression analysis to filter significantly prognostically relevant genes as a hub gene set.

In addition, a total of 101 combinations of 10 machine learning algorithms, including Least absolute shrinkage and selection operator (LASSO), CoxBoost, Ridge, generalized boosted regression modeling (GBM), partial least squares regression for Cox (plsRcox), supervised principal components (SuperPC), elastic network (Enet), stepwise Cox, random survival forest (RSF), survival support vector machine (survival-SVM) together with 10-fold cross-validation were introduced to select candidate genes with the highest C-index for IMMPS. The stepCox (both) and Ridge were used to develop the IMMPS model. Specifically, the former was applied to filter the most valuable TIICGs, while the later was used to fit the most reliable model. After the Ridge regression, the IMMPS formula for each variable was as follow:

$$IMMPS = \mathop \sum \limits_{k = 1}^n {x^k}{a^k}$$

where *n* denoted the number of feature genes, ${a^k}$ denoted the ridge regression coefficient of the k^th^ feature gene, and ${x^k}$ denoted the expression level of the k^th^ feature gene.

### Enrichment analysis

Gene oncology (GO) and Kyoto Encyclopedia of Genes and Genomes (KEGG) enrichment analyses of specific gene modules were performed using the R package “clusterProfiler” (Yu et al., 2012).

### Immunotherapy response prediction

GSE126044 (Cho et al., 2020) and GSE135222 (Kim, Choi & Jung, 2020) were two cohorts containing follow-up information of each NSCLC patient’s response to anti-PD-1/PD-L1 therapy. Subclass mapping was conducted using the SubMap (Hoshida et al., 2007) algorithm to evaluate the response of immune molecular subtypes to immunotherapy, and potential treatment benefit was determined by Tumor Immune Dysfunction and Exclusion (TIDE) algorithm.

### Cell culture and transfection

Human normal lung epithelial cells BEAS-2B (BNCC359274) and human LUAD cells lines PC-9 (BNCC340767) and H1395 (BNCC100270) were purchased from Bena Biotechnology Co. (Beijing, China). Penicillin/streptomycin and Dulbecco’s modified Eagle’s medium (A1896701; DMEM, Gibco, Grand Island, NY, USA) with 10% fetal bovine serum (FBS, Gibco, USA) was used for cell culturing at 37 °C in 5% CO_2_.

*BTNL9* plays a critical role in immune regulation, especially in TME that may affect the activity of immune infiltrating cells (Zhang et al., 2023). In this study, to investigate the regulatory effects of *BTNL9* on the expression of the seven immune-related genes, human LUAD cell lines (PC9 and H1395) and normal lung epithelial cells (BEAS-2B) were transfected with *BTNL9*-specific siRNA (5′-3′ sequence: GCCTCTAACTCCACAACAACACT) using Lipofectamine 3000 (Thermo Fisher, Waltham, MA, USA) following the manufacturer’s protocol. After 48 h, the transfection efficiency was tested by qRT-PCR.

### Western blot testing

Cells were lysed using RIPA buffer supplemented with protease inhibitors. Protein samples were separated on 15% SDS-PAGE gel, subsequently transferred onto PVDF membranes (Beyotime, Shanghai, China) and blocked. The membranes were incubated overnight at 4 °C with primary antibodies against *BTNL9* (1:1,000) and GAPDH (1:1,000), and then treated with HRP-conjugated secondary antibodies for 1 h at room temperature. Finally, the protein bands were visualized utilizing the ECL system (Amersham Biosciences, Inc, Buckinghamshire, UK) and the band intensities were measured using Quantity One software (BioRad, Hercules, CA, USA).

### Quantitative real-time PCR (qRT-PCR)

Total RNA was extracted from BEAS-2B, PC9 and H1395 (Thermo Fisher, Waltham, MA, USA) using TRIzol reagent. The HiScript II SuperMix (Vazyme, China) was employed for isolating cDNA from 500 ng of RNA. QRT-PCR was carried out with the use of the SYBR Green Master Mix in ABI 7500 System (Thermo Fisher Scientific, Waltham, MA, USA). PCR amplification began with 45 cycles a 94 °C for 10 min, at 94 °C for 10 seconds (s), and 60 °C for 45 s. GAPDH was an internal reference. All the primer sequences were shown in Table 2.

**Table 2:** Sequence of primers.

Gene	Forward primer sequence (5′-3′)	Reverse primer sequence (5′-3′)
SEMA7A	TTCAGCCCGGACGAGAACT	GAACCGAGGGATCTTCCCAT
EFHD2	GGATGAGGACTTTGACAGCAAGC	TTGACACCCTCACTGGAGACGT
CHST11	CACAAGCCGTAAGCGGAGG	CATGGGGTCGCTGTACTTCC
SLC24A4	ATGGCCCCAGTGAATGGGA	CCAGCCACATCTTCGCTCAG
MAL	ACCGCTGCCCTCTTTTACC	GAAGCCGTCTTGCATCGTGAT
JCHAIN	TCCTGGCGGTTTTTATTAAGGC	AGTAATCCGGGCACACTTACAT
SCARF1	CCGATCAGACCTCAAGGACAG	CCCAGGGTAGCTTGTGGGA
BTNL9	GGACCTGTTCAGTCTGGAAAC	TCTGGACCACCAACTCTTTCT
GAPDH	CTGGGCTACACTGAGCACC	AAGTGGTCGTTGAGGGCAATG

### Statistical analyses

Heatmap was generated using the R package ComplexHeatmap (Gu, Eils & Schlesner, 2016). Wilcox test and Kruskal-Wallis test were employed to analyze two-group differences and comparisons between multiple groups, respectively. The correlation between two groups of samples was calculated by Pearson’s correlation test. Prognostic differences among patients were assessed using Kaplan-Meier. The R package “timeROC” was used to plot time-dependent receiver operating characteristic (ROC) curves (Blanche, Dartigues & Jacqmin-Gadda, 2013). All the statistical analyses were conducted in R 3.6.4 (R Core Team, 2021). GraphPad Prism V.8.0.2 was applied for the statistical analysis on the experimental data, which were compared using one-way analysis of variance or unpaired *t*-test. A *p* < 0.05 indicated a statistical significance.

## Results

### Development and validation of immune infiltration molecular subtypes

**Figure 1:**
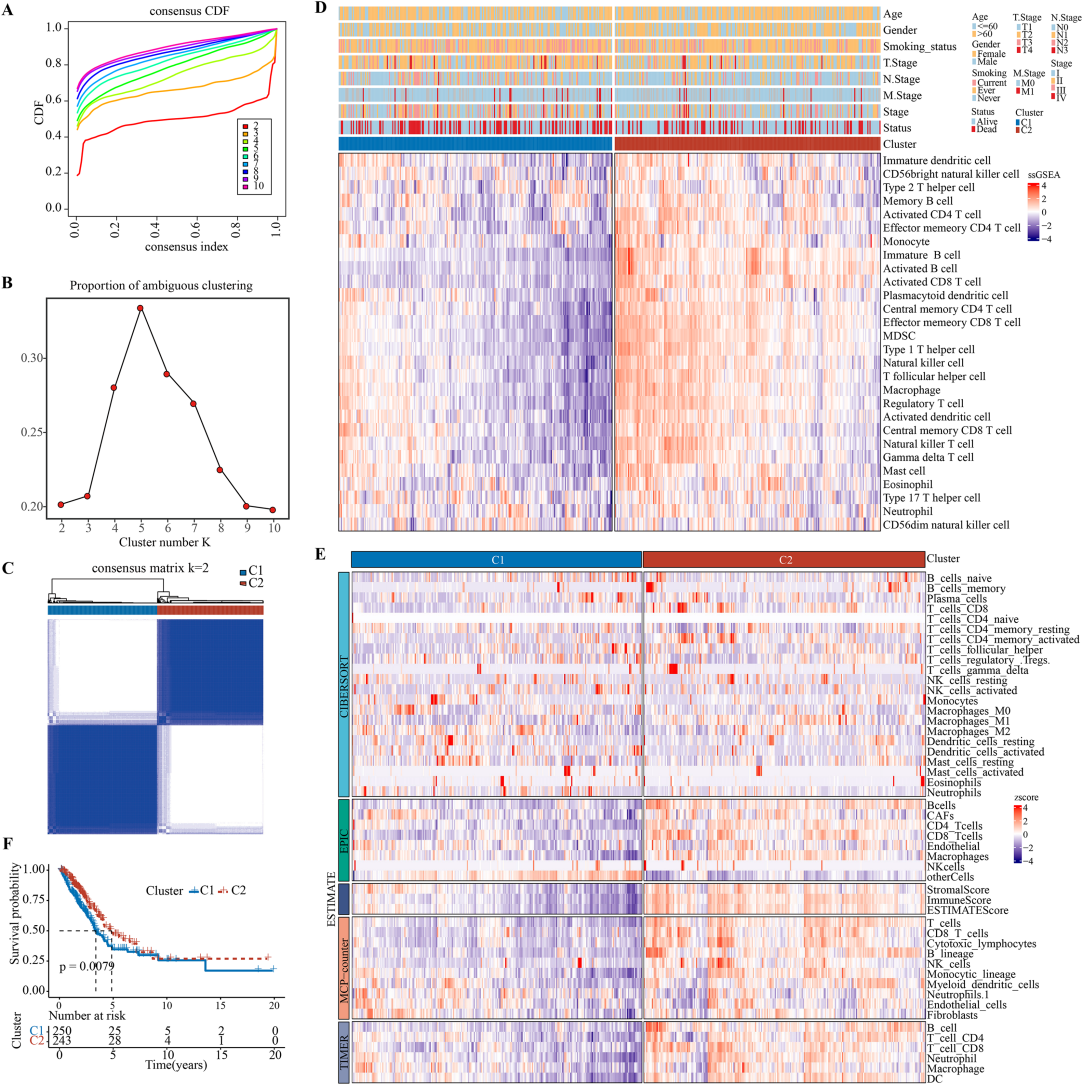
Development and validation of immune infiltration consensus clusters. (A) The CDF curves of consensus matrix for each k (indicated by colors). (B) The proportion of ambiguous clustering score, with a low PAC value indicating a flat middle segment. The optimal k (k = 2) was determined according to the lowest value of PAC. (C) When k = 2, consensus score matrix for all samples, with a higher consensus score between two samples indicating a higher chance of the two being grouped into the same cluster in different iterations. Each cell in the figure represents the consistency score between the two samples. Blue color indicates high consistency (strong similarity); white color indicates low consistency (weak similarity). (D) The infiltration abundance of 28 immune cell subsets was determined by ssGSEA for two clusters. (E) The stability and robustness of the ssGSEA results were further confirmed by the algorithms of ESTIMATE, CIBERPSORT, TIMER, MCP-counter, and EPIC. (F) Kaplan–Meier curves of OS in the C1-*vs*-C2 in TCGA-LUAD (log-rank test: *P* = 0.0079).

Charoentong et al. (2017), Bindea et al. (2013) revealed that at least 28 different types of immune cell types are crucially involved in tumor infiltration. Immune cell infiltration in the TME of LUAD were analyzed by single-sample gene set enrichment analysis (ssGSEA). Patients were classified by consensus clustering. CDF curves of the consensus score matrix (Fig. 1A) and PAC results (Fig. 1B) showed that the optimal number of clusters was when k = 2 (Fig. 1C). The sigclust analysis detected significant differences (*p* = 1.141007e−05) between the two consensus clusters of C1 and C2 in terms of immune infiltration, with C1 showing a notably lower overall infiltration than C2 (Fig. 1D, Fig. S2A). In addition, ESTIMATE, CIBERPSORT, TIMER, EPIC, and MCP-counter results showed similar results (Fig. 1E, Figs. S2B–S2F). Hence, C1 and C2 were accordingly defined as an immunosuppressed and immune-activated subtypes. Survival analyses all demonstrated a significantly worse prognosis for C1 than C2 (Fig. 1F).

### The expression patterns of immune checkpoint genes in immune-activated subtype and potential benefits from immunotherapy

**Figure 2:**
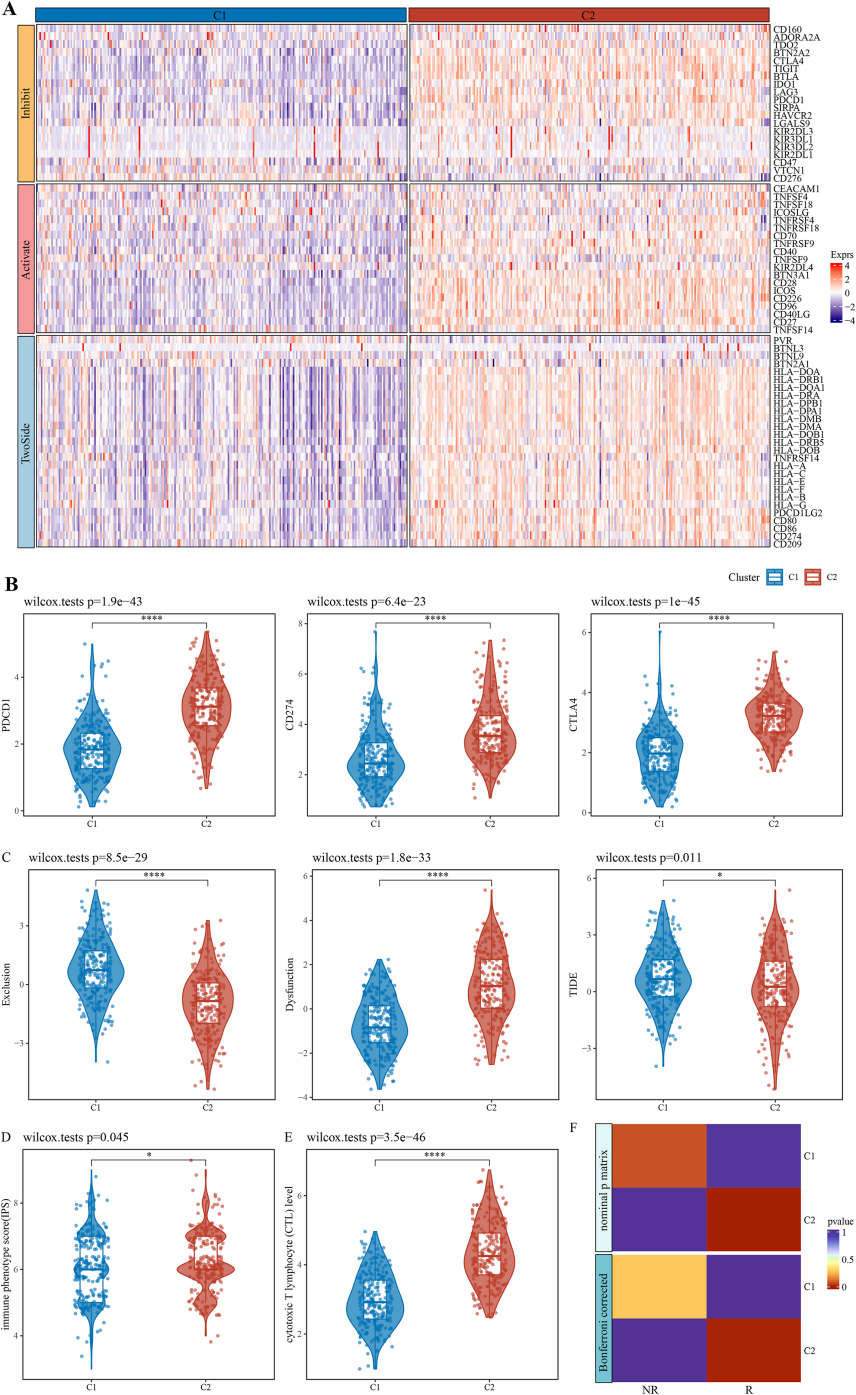
Expression pattern of immune checkpoint genes in immune-activated subtypes and potential benefits of immunotherapy. (A) Expression pattern of ICG in patients with two immune subtypes. (B) Expression distribution of classical immune checkpoint genes PDCD1, CD274, and CTLA4 in the two immune subtypes. (C) Distribution of T cell exclusion and dysfunction in the two immune subtypes analyzed by TIDE. (D) Distribution differences of immune phenotype score in the two immune subtypes. (E) Distribution differences of the two immune subtypes at the cytotoxic T lymphocyte (CTL) level. (F) Submap analyses show that patients in the immune activation subtypes are similar to those who responded to anti- PD-1 treatment. * *p* < 0.05; **** *p* < 0.0001.

After literature review, a total of 79 immune checkpoint genes (ICGs) (Table S1) mainly involved in ligand-receptor interactions were selected. These ICGs affect immune activity in different ways, including stimulation, inhibition or a combination of both. Analysis of expression patterns of these genes in the C1 and C2 subtypes demonstrated that almost all the ICGs, such as PDCD1, CD274 and CTLA4 (Fig. 2B), were upregulated in C2 (Fig. 2A). TIDE model could evaluate T cell exclusion and dysfunction signatures across 33,000 samples in 188 tumor cohorts stored in public databases, including TCGA (Weinstein et al., 2013), METABRIC (Curtis et al., 2012), and PRECOG (Gentles et al., 2015). Applying TIDE model, it was observed that the C1 subtype had significantly higher T cell exclusion and the C2 subtype had significantly higher T cell dysfunction, and that C2 subtype might benefit from taking immunotherapy based on combined TIDE scores (Fig. 2C). Recent studies have confirmed immunophenoscore (IPS) as an accurate predictor of ICI response, with a higher IPS predicting greater sensitivity to ICI treatment. Here, we found a significantly higher IPS score of C2 than C1 (Fig. 2D, *p* = 0.045), which was also validated by the cytotoxic T lymphocyte score (Fig. 2E). Further, the gene expression patterns of immune phenotypes of NSCLC patients who received ICIs were compared by SubMap analysis. Notably, the gene expression profiles of lung cancer patients who responded to anti-PD-1 immunotherapy were highly similar to those with immune-activated subtypes in both the training and validation cohorts (Fig. 2F). These results indicated that patients with immune-activated subtypes could benefit from receiving ICI immunotherapy, especially more from anti-PD-1 therapy.

### Identification of immune infiltration-associated gene modules and prognostic features of the genes

**Figure 3:**
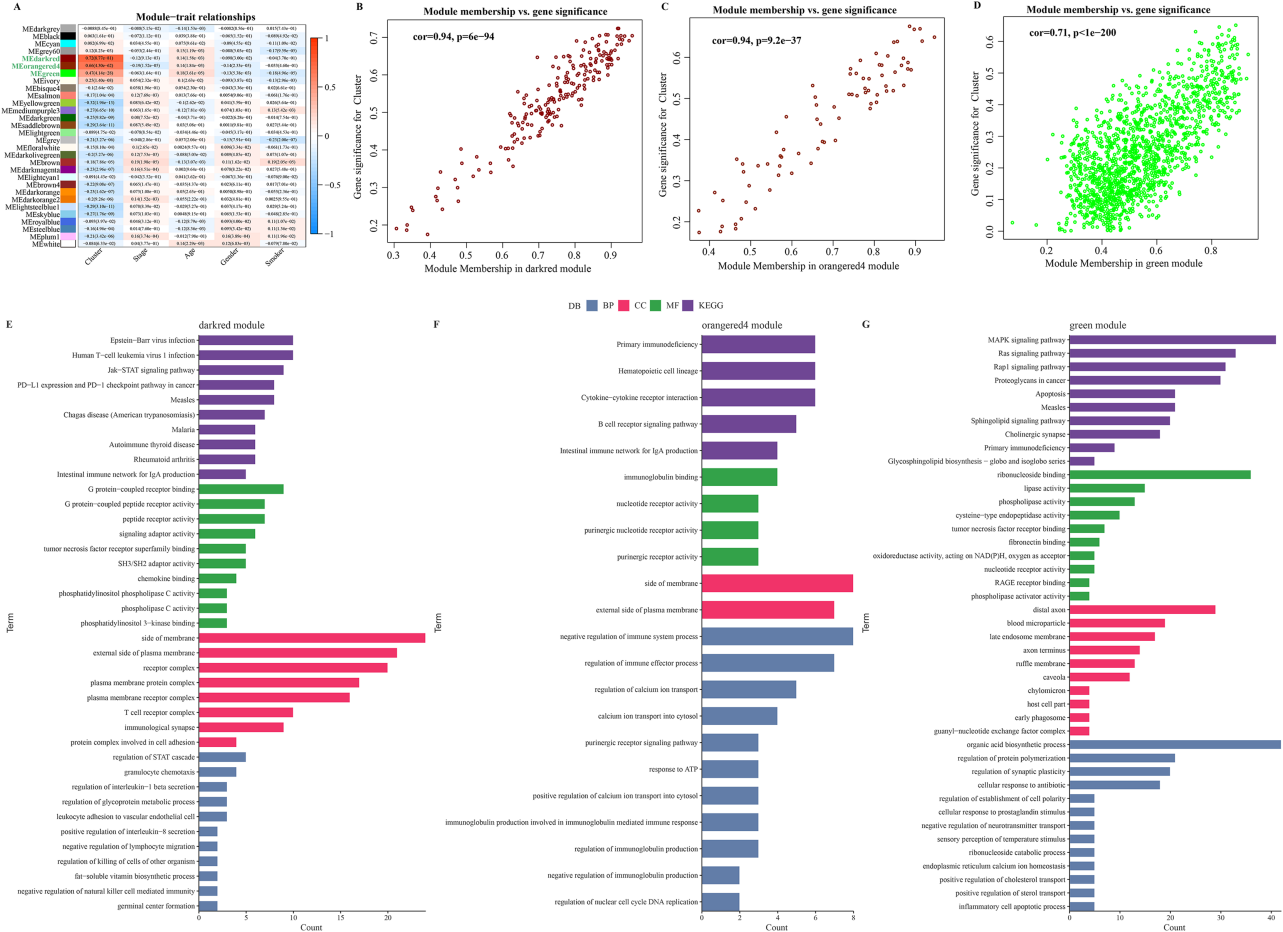
Identification of immune infiltration-associated gene modules and prognostic features of the genes. (A) Correlation analysis between gene modules and clinical features. (B–D). Close correlation between GS and MM in the darkred, orangered4 and green modules (*p* < 0.0001). Statistical tests: Pearson’s correlation coefficient, two-sided unpaired t-test. (E–G). Enrichment results of genes in darkred, orangered4 and green module to KEGG Pathway, biological process (BP), cellular component (CC) and molecular function (MF).

The soft threshold β = 8 was selected (Figs. S2A, S2B) to ensure a scale-free nature of the network. A total of 30 gene modules were detected, as indicated by different colors, and the heatmap displayed the characteristic gene neighborhood of the modules (Fig. S2C). Additionally, the modules was weakly correlated with AJCC Stage, Age and Gender (Fig. 3A). Specifically, darkred, orangered4, and green modules were considered as the modules most closely correlated with C2 (immune activation) subgroup. Among the three modules, the correlation coefficient between module membership (MM) and gene significance (GS) of the darkred, orange4, and green modules reached 0.94, 0.94, and 0.71 (Figs. 3B–3D), respectively, indicating that the identification of these gene modules was reliable. Further functional enrichment analysis showed that the darkred module was enriched to pathways of human T−cell leukemia virus 1 infection, PD−L1 expression, PD−1 checkpoint pathway in cancer, *etc*. (Fig. 3E). The green module was significantly enriched to Cytokine-cytokine receptor interaction, B cell receptor signaling pathway, and other pathways (Fig. 3F). The orangered4 module was significantly enriched to primary immunodeficiency, MAPK signaling pathway and some other pathways (Fig. 3G). These pathways all play critical role in immune regulation. Noticeably, biological processes such as cytokine activity, cytokine receptor activity, cytokine binding, and cytokine receptor binding were enriched multiple times. Under stringent criteria, genes significantly associated with LUAD prognosis were identified by performing one-way survival analysis from at least four cohorts, and finally 26 genes were screened as potential prognostic markers with significant immune relevance in LUAD (Fig. S2E).

### Development of an immune-correlated prognostic signature and assessment

**Figure 4:**
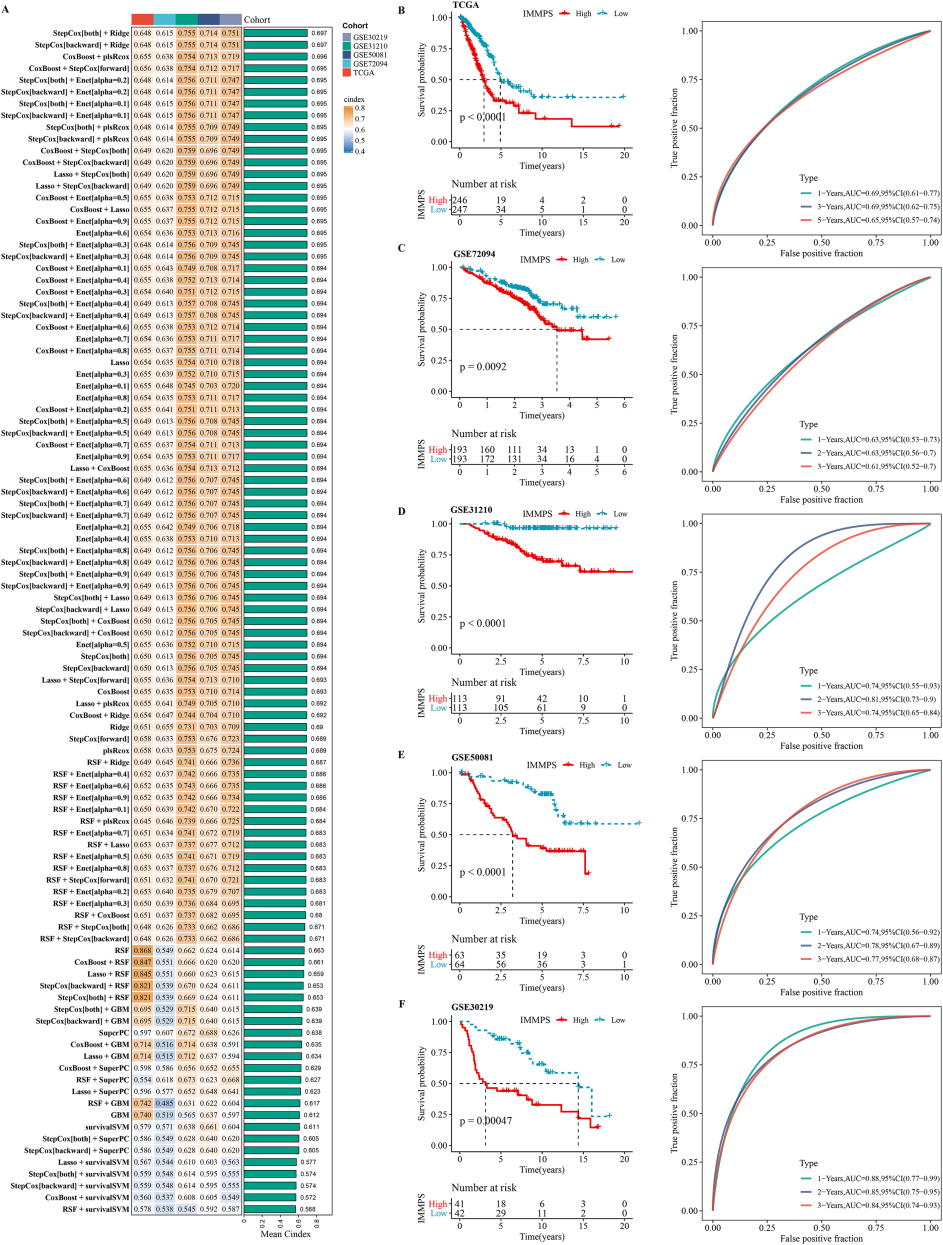
Construction and assessment of immune-correlated prognostic characteristics. (A) Heatmap of c-index distribution of 101 prediction models in five cohorts. (B–F) Kaplan–Meier curves and ROC of patients in low- and high-risk groups after risk assessment of patients using IMMPS in different cohorts.

Using machine learning-based integration analysis, an immune-related gene signature was developed based on the expression profiles of 26 immune-related genes. In the TCGA-LUAD dataset, 101 predictive models were developed by the global leave-one-out cross-validation (LOOCV) framework and their C-index in all the validation datasets was calculated. Interestingly, the optimal model was the combination of stepCox and Ridge, which showed the highest average C-index (0.697) in all the validation datasets (Fig. 4A). In step cox regression, when the Akaike Information Criterion (Pagès et al., 2018) reached its minimum value, the optimal combination of seven variable genes were identified. Therefore, the final model (IMMPS) was built with these seven genes applying regression analysis with Ridge: IMMPS = 0.093*SEMA7A + 0.145*EFHD2 + 0.108*CHST11 − 0.502*SLC24A4 − 0.089*MAL − 0.074*JCHAIN − 0.128* *SCARF1*. Each patient was assigned with a risk score by the model and weighted for their regression coefficient. Based on the optimal cut-off value, all the patients were categorized into high- and low- risk groups (Figs. 4B–4F). It was observed that the OS of high-risk patients was significantly worse than the low-risk patients in the TCGA-LUAD training dataset and the five validation datasets (all *p* < 0.05). ROC analysis showed that the IMMPS reached 1-, 3-, and 5-year area under the curves (AUCs) of 0.69, 0.69, and 0.65 in TCGA-LUAD, respectively. In GSE72094, the AUC values were consistent at 0.63 for all three time points. For GSE31210, 1-, 3-, and 5-year AUCs were 0.74, 0.81, and 0.74, respectively, while in GSE50081, they were 0.74, 0.78, and 0.77, respectively. Finally, the GSE30219 cohort showed AUC values of 0.88, 0.85, and 0.84 at the corresponding time points. These data supported a stable and robust performance of IMMPS across different cohorts. Consistently, multivariate Cox regression analysis revealed that IMMPS was an independent risk factor for LUAD (*p* < 0.001, Fig. S3).

### Comparison of gene expression-based prognostic features for LUAD

**Figure 5:**
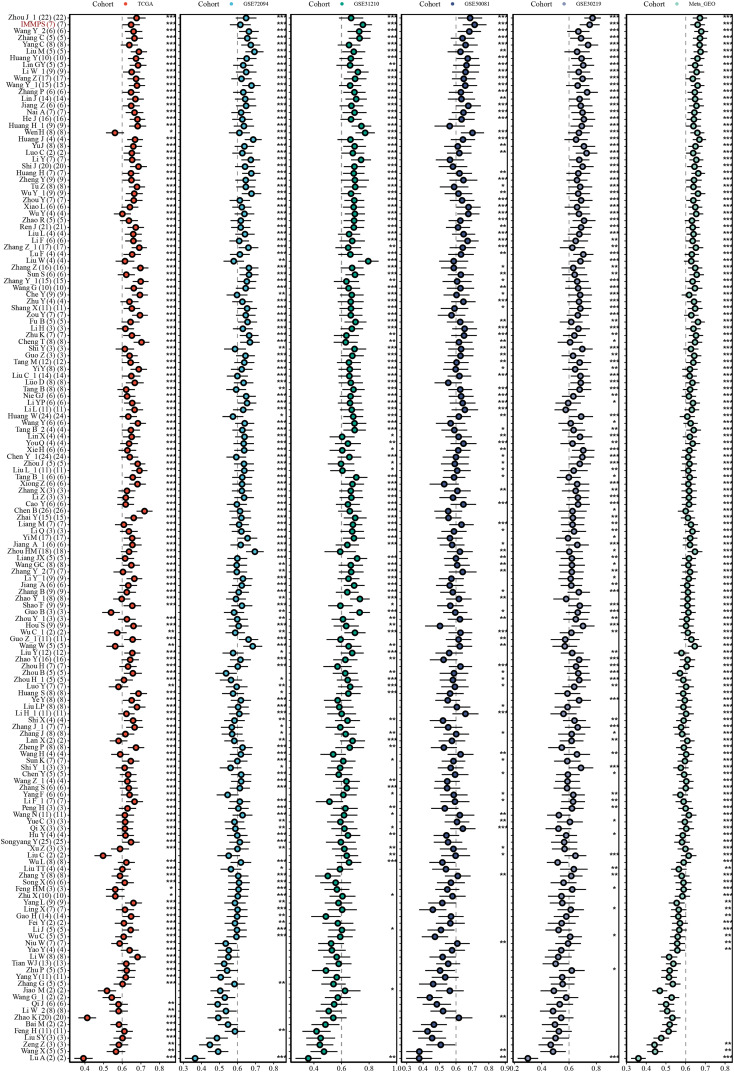
The c-index comparison of IMMPS with 154 gene signatures in five datasets. * *p* < 0.05; ** *p* < 0.01; *** *p* < 0.001; blank denotes *p* > 0.05. Each dot indicates the corresponding c-index. The horizontal lines near the dots indicates the confidence intervals. IMMPS and 154 signatures are shown vertically, and six data cohorts are shown horizontally.

Next-generation sequencing and big data technology has promoted the prediction of genes by machine learning-based methods (Ahluwalia, Kolhe & Gahlay, 2021). This study comprehensively compared the performance of published signatures to the IMMPS. A severe lack of miRNA and lncRNA data in the validation dataset excluded the miRNA and lncRNA signatures, resulting in the collection of a total of 154 signatures (Table S2). These signatures were related to different biological processes, such as ferroptosis, WNT, stemness, autophagy, hypoxia, adipogenesis, glycolysis, epigenetics, immune response, vitamin D, aging, epithelial-mesenchymal transition, N6-methyladenosine, Toll-like receptor signaling, and drug sensitivity. The c-index of each signature in all the datasets were calculated to evaluate their predictive performance (Fig. 5). According to the c-index, ranking of the signatures in each dataset showed that one signature (0.6%) outperformed the IMMPS in three datasets. Conversely, 49 signatures (31.8%) exhibited a lower c-index than the IMMPS in three cohorts (60% of the total cohorts), 47 signatures (30.5%) showed a lower c-index than the IMMPS in four cohorts (80% of the total cohorts), and 57 signatures (37%) had a lower c-index than the IMMPS across all cohorts (Fig. S4). These results supported the stability of the IMMPS. We also noted that while most of the models performed well in their own training set and some specific external datasets, their performance was notably poor in other datasets. This discrepancy could be attributed to poor model generalization due to overfitting problem. Next, two machine learning algorithms were employed to reduce the gene numbers in our signature, which thereby manifested a stronger generalization ability. In comparison to the model developed by (Deng et al., 2022), the IMMPS contained only seven genes. Fewer genes with comparable predictive performance could significantly reduce the cost in clinical testing. These results confirmed that IMMPS had the advantage of higher stability but lower detection cost in comparison with previous gene signatures.

### The impact of the IMMPS on ICI treatment

**Figure 6:**
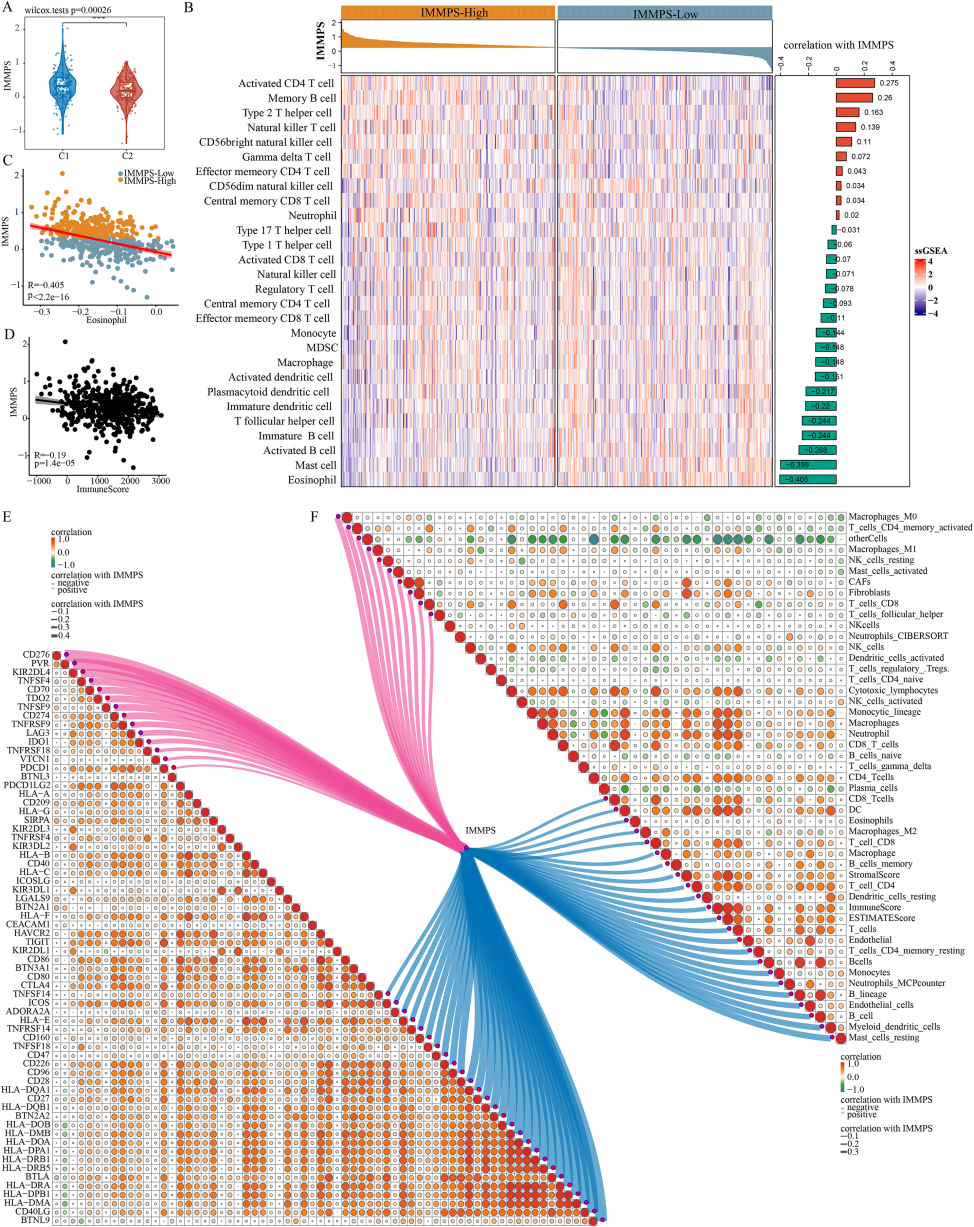
Impact of IMMPS on ICI treatment. (A) Differences in the distribution of IMMPS in the immunoactivated (C2) and immunosuppressed (C1) subtypes. (B) Differences in the distribution of 28 immune-infiltrating cells in patients with different IMMPS levels. The colors in the heatmap indicate the immune-cell infiltration scores after z-score. The right bar graph shows the correlation between IMMPS and the corresponding immune infiltration scores, with pink indicating positive correlation and green indicating negative correlation. The top bar graph represents the IMMPS scores of patients in the TCGA-LUAD cohort. (C, D) The correlation plot between IMMPS and eosinophil as well as ImmuneScore, each dot represents a sample. (E, F) Correlation of IMMPS with the expression of immune checkpoint genes and immune infiltrating cells obtained by different algorithms, respectively. Each dot indicated the correlation coefficient between immune checkpoint genes. Larger dots indicate higher correlation coefficients, lines indicate immune checkpoints significantly correlated with IMMPS, thicker lines indicated higher correlation, blue indicates negative correlation, red indicated positive correlation.

Comparison on the differences of the IMMPS in the two subtypes showed a significantly lower IMMPS in C2 patients than in C1 patients (Fig. 6A, *p* = 0.00026). The patients were then classified into two groups of IMMPS-High and IMMPS-Low using the IMMPS. It can be observed that immune infiltrating cell scores of patients with IMMPS-High were significantly lower (Fig. 6B). A total of 18 types of immune infiltrating cells (64.3%) were negatively correlated with the IMMPS. In particular, eosinophils had the strongest correlation with IMMPS (Fig. 6C), and patients with low IMMPS but more eosinophils had a lower risk of Coronavirus disease 2019 (COVID-19) and better clinical outcomes (Xie et al., 2021). The stability of the results was verified using five other algorithms to avoid bias from different algorithms. IMMPS-High patients showed remarkably lower immune infiltrating cell scores (Figs. S5A–S5E), which was verified by correlation analysis (Fig. 6F). These results validated a negative correlation between immune infiltration and IMMPS (Fig. 6D), specifically, patients who had low IMMPS and higher immune cell infiltration could benefit more from taking immunotherapy. Additionally, analysis on the correlation between immune checkpoint genes and the IMMPS (Fig. 6E) revealed that most of the HLA Class II genes were significantly negatively related to IMMPS.

### Experimental verification using cell lines

**Figure 7:**
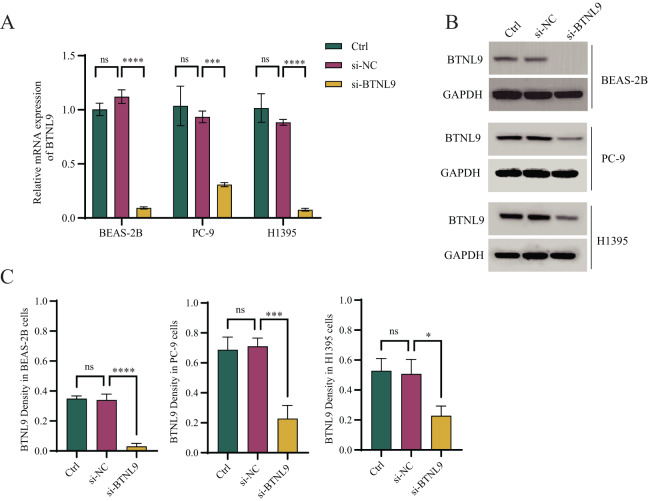
Based on qRT-PCR and Western blot assays to validate the knockdown efficiency of *BTNL9* in LUAD cell lines. (A) Based on qRT-PCR to validate mRNA expression levels after knockdown of *BTNL9* in LUAD cell lines. (B, C) Western blotting-based assay to validate the protein expression level and its quantitative analysis after knockdown of *BTNL9* in LUAD cell line. Ctrl refers to the blank control group; si-NC refers to the control group transfected with non-targeting siRNA; and si-BTNL9 refers to the experimental group transfected with *BTNL9*-specific siRNA. *p < 0.05; ***p < 0.001; ****p < 0.0001; ns, no significant difference.

**Figure 8:**
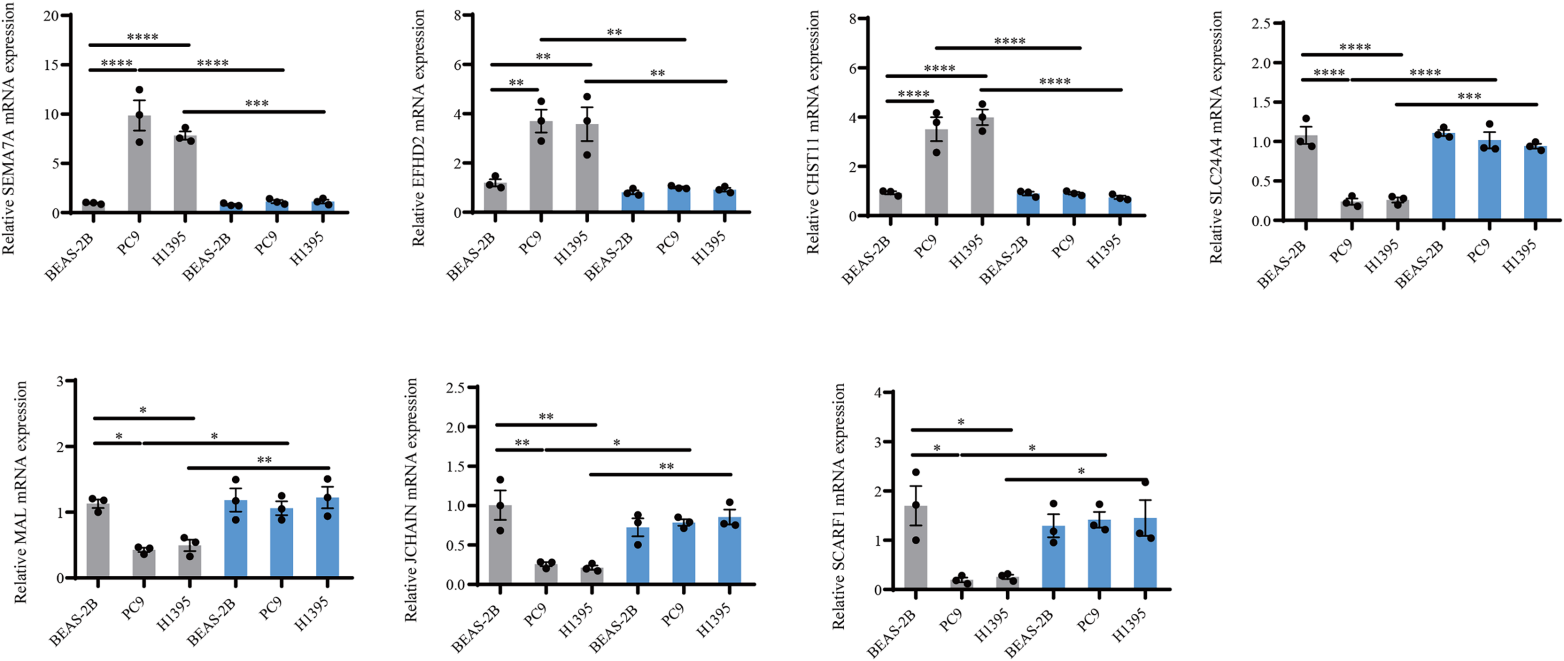
PCR validation of the reliability of the IMMPS model. QRT-PCR was used to detect *SEMA7A*, *EFHD2*, *CHST11*, *SLC24A4*, *MAL*, *JCHAIN* and *SCARF1* expression in BEAS-2B, PC9, H1395 cell lines after *BTNL9* silence (*n* = 3). Gray color serves as a baseline reference for normal controls to assess gene expression changes in LUAD cells; blue color indicates expression changes in mRNA levels of seven genes after knockdown of BTNL9. *p < 0.05; ** *p* < 0.01; *** *p* < 0.001; **** *p* < 0.0001. The results were presented as mean ± SEM.

Considering that *BTNL9* was the most relevant immune checkpoint gene, *BTNL9* expression was inhibited in BEAS-2B, PC9, and H1395 cell lines and simultaneously the expression of seven genes in the IMMPS model was measured. First, the knockdown efficiency of *BTNL9* in the LUAD cell lines was detected based on qRT-PCR and Western blot assays. As shown in Fig. 7A, knockdown of *BTNL9* significantly downregulated the mRNA expression level of *BTNL9* in human normal lung epithelial cells BEAS-2B and LUAD cell lines PC9 and H1395. Similarly, *BTNL9* protein levels were significantly lowered in the si-*BTNL9* group in comparison to the blank control group (Ctrl) and the control group transfected with non-targeting siRNA (si-NC) (Figs. 7B,7C). After *BTNL9* knockdown, the mRNA expression levels of the seven genes in three cell lines (BEAS-2B, PC9, and H1395) were detected. The results showed that after knockdown of *BTNL9*, the expression of the seven genes in LUAD cell lines (PC9 and H1395) demonstrated significant differences than before the knockdown. This suggested that *BTNL9* may be an upstream regulator of these genes, and that knockdown of *BTNL9* changed the expression of these seven genes in LUAD cells, indicating that these genes may be involved in the occurrence and development of LUAD (Fig. 8).

## Discussion

Immunotherapy is a fast developing area of therapeutic molecularly targeted therapies for the treatment of advanced tumors, but not all patients respond actively to immunotherapy. Predictive features are urgently needed to be determined in order to accurately identify patients who can potentially benefit from immunotherapies. The TME is composed of multiple cell types interacting with each other *via* growth factors, cytokines, and chemokines (Duan et al., 2020), providing a basis for evaluating the effectiveness of immunotherapy (Zemek et al., 2019). Tumors can be classified as either immune-cold or immune-hot according to the features of the TME. Specifically, immune-cold tumors are characterized by immunosuppressive TME and insensitivity to immunotherapy, while immune-hot tumors had a high sensitivity to immunotherapy with active T-cell infiltration (Gajewski, 2015). Therefore, the use of practical biomarkers to differentiate the two types of tumors may be able to evaluate the response to immunotherapy.

This study determined immune-hot and immune-cold LUAD tumors using consensus clustering. Genes related to immune-hot LUAD were filtered by WGCNA, and based on the expression profiles of these genes, an integrated pipeline was developed to build the IMMPS. ROC and C-index analyses showed a high accuracy and stability of the IMMPS in one training cohort and four independent cohorts, along with a high generalization ability. These findings supported a great potential of IMMPS in clinical applications.

We collected 154 gene signatures published in the last 5 years. Despite the fact that considerable signatures have been proposed, only a few have been clinically validated or translated into clinical use (Zeng et al., 2022; Wang et al., 2020). This may be explained by a poor model generalization due to overfitting problem. Noticeably, the model proposed by Deng et al. (2022) showed a similar performance to the IMMPS across all the cohorts, but the applicability of their model was severely limited by a high cost to detect a total of 22 genes in the model. However, the IMMPS developed with fewer featured genes by the combination of two machine learning algorithms had a better generalization capability.

Over the past few years, checkpoint inhibitor-based immunotherapies have been substantially advanced for many cancer types, including LUAD (Havel, Chowell & Chan, 2019). Although immune checkpoint genes, such as PD-L1, are available biomarkers in clinical practice, their expression could not be independently used to indicate ICI response (Lupo et al., 2018). Our study found that immune-hot tumors had a lower IMMPS. Moreover, the IMMPS showed a significant negative correlation with a greater number of immune checkpoint genes, particularly HLA Class II genes. A study reported that HLA class II-restricted neoantigens could influence patients’ responses to ICB in a way distinct and complementary to the responses mediated by HLA class I (Shao et al., 2022). Moreover, an early study also identified HLA class II molecule as a potential marker of immune checkpoint blockade (ICB) in NSCLC (Mei et al., 2022). These results indicated that LUAD patients with a low IMMPS could benefit from ICI treatment.

The IMMPS model contained seven genes, which all played an important role in the development of inflammation or cancer. *SEMA7A* has been reported to exert an anti-inflammatory effect on the human body possibly through converting pro-inflammatory M1 macrophages into anti-inflammatory M2 macrophages (Körner et al., 2021). However, in the research related to atherosclerosis, Hong et al. (2020) found that *SEMA7A* could promote ontological to mesenchymal transition through TGF- β 2/Smad signaling. Therefore, it was speculated that *SEMA7A* can serve as a target for vascular growth inhibition in cancer treatment. *EFHD2* could promote the tolerance of NSCLC to cisplatin treatment through NOX4-ROS-ABCC1 axis (Fan et al., 2020). Study showed that *CHST11* is related to lung cancer (Li et al., 2022), consistently, our qPCR results also confirmed that this gene was high-expressed in advanced LUAD patients and may be involved in the metastasis of NSCLC through dysregulation of ceruloplasmin and intracellular iron balance (Chang et al., 2022). *SLC24A4* has been identified as a methylation driver gene and used to construct a riskscore model for LUAD prognosis (Ren et al., 2020). *MAL*, a member of MAL family of integral membrane proteins, has the potential to be considered as a biomarker for cancer (Labat-de-Hoz et al., 2023). *JCHAIN* is one of the marker genes of B cells in LUAD tissue (Zhang et al., 2023). Ma et al. (2021) found that LUAD patients with a high expression of *JCHAIN* have a longer survival time. Consistently, our qPCR results also revealed a low expression of *JCHAIN* in advanced LUAD patients. This indicated that *JCHAIN* may be a prognostic marker for LUAD. A recent study reported that advanced hepatocellular carcinoma patients with a high level of *SCARF1* have a favorable overall survival (Patten et al., 2020), suggesting an anti-tumor effect of the gene. Overall, the seven genes discovered by this study had significant biological significance and could be used as targets for LUAD treatment in the future.

However, some limitations of this research should be equally acknowledged. Firstly, all the samples analyzed in this study were retrospective, therefore future validation of the IMMPS using prospective multi-center cohorts is imperative. Secondly, some of the clinical and molecular features in the public datasets were highly underrepresented, potentially obscuring the associations between IMMPS and certain variables.

## Conclusions

Applying multiple bioinformatics and machine learning algorithms, the current research developed a stable and robust gene signature for assessing the potential benefit of immunotherapy and the prognosis of LUAD patients. The IMMPS model was a promising tool to optimize clinical decision-making and a monitoring protocol for individual LUAD patient.

## Supplemental Information

10.7717/peerj.19121/supp-1 Supplemental Information 1 Raw experimental data.

10.7717/peerj.19121/supp-2 Supplemental Information 2 A. The distribution of 28 immune cell subsets infiltration between two clusters. B. The distribution of immune score inferred by ESTIMATE algorithm between two clusters in the TCGA-LUAD cohort. C. The distribution of 22 immune cell subsets infiltration by CIBERSORT algorithm between two clusters in the TCGA-LUAD cohort. D. The distribution of 6 immune cell subsets infiltration by TIMER algorithm between two clusters in the TCGA-LUAD cohort. D. The distribution of 7 immune cell subsets infiltration by EPIC algorithm between two clusters in the TCGA-LUAD cohort. E. The distribution of 10 immune cell subsets infiltration by MCP-counter algorithm between two clusters in the TCGA-LUAD cohort. Here, * denotes p<0.05, ** denotes p<0.01, *** denotes p<0.001 and NS denotes p>0.05.

10.7717/peerj.19121/supp-3 Supplemental Information 3 A: Analysis of the scale-free fit index for various soft-thresholding powers (β). B. Analysis of the mean connectivity for various soft-thresholding powers. C. Heatmap of all differentially expressed genes clustered based on a dissimilarity measure (1-TOM). D. Distribution of the number of genes in each module. E. Risk status of the 26 genes in different cohorts.

10.7717/peerj.19121/supp-4 Supplemental Information 4 A-F. Forest plot of prognostic characteristics from multifactorial survival analyses to compare AJCC Stage, Smoker, Age, Gender, and IMMPS in different cohorts.

10.7717/peerj.19121/supp-5 Supplemental Information 5 154 gene signatures in five datasets with c-index sorted above or below the IMMPS statistics. Green indicates that the c-index of the signature in the corresponding dataset is lower than the IMMPS. Pink color indicates that the c-index of the signature is above than IMMPS in the corresponding dataset. The five data cohorts are represented horizontally and the corresponding 154 signatures are represented vertically.

10.7717/peerj.19121/supp-6 Supplemental Information 6 A. The distribution of immune score inferred by ESTIMATE algorithm between two clusters in the TCGA-LUAD cohort. B. The distribution of 22 immune cell subsets infiltration by CIBERSORT algorithm between two clusters in the TCGA-LUAD cohort. C. The distribution of 6 immune cell subsets infiltration by TIMER algorithm between two clusters in the TCGA-LUAD cohort. D. The distribution of 7 immune cell subsets infiltration by EPIC algorithm between two clusters in the TCGA-LUAD cohort. E. The distribution of 10 immune cell subsets infiltration by MCP-counter algorithm between two clusters in the TCGA-LUAD cohort. *denotes p<0.05, ** denotes p<0.01, *** denotes p<0.001,NS denotes p>0.05.

10.7717/peerj.19121/supp-7 Supplemental Information 7 Summary of immune checkpoint genes collections.

10.7717/peerj.19121/supp-8 Supplemental Information 8 Data on a total of 154 previous gene signatures.

10.7717/peerj.19121/supp-9 Supplemental Information 9 Supplementary material.

10.7717/peerj.19121/supp-10 Supplemental Information 10 MIQE checklist.

## Abbreviations

BP: Biological Process
CC: Cellular Component
CDF: cumulative distribution function
CIBERSORT: Cell-type Identification By Estimating Relative Subsets Of RNA Transcripts
CT: tumor core; E
net: elastic network
CTL: cytotoxic T lymphocyte
ESTIMATE: Estimation of STromal and Immune cells in MAlignant Tumours using Expression data
FDA: Food and Drug Administration
ICGs: immune checkpoint genes
ICIs: immune checkpoint inhibitors
GBM: generalized boosted regression modeling
GEO: Gene Expression Omnibus
GS: gene significance
IMMPS: immune prognostic signature
IM: invasive margins
LOOCV: leave-one-out cross-validation
LUAD: Lung adenocarcinoma
LUSC: lung squamous cell carcinoma
MAD: Median Absolute Deviation
MF: Molecular Function
MM: module membership
NSCLC: non-small cell lung cancer
PAM: partioning around medoids
PlsRcox: partial least squares regression for Cox
qRT-PCR: Quantitative real-time polymerase chain reaction
ROC: receiver operating characteristic
RSF: random survival forest
ssGSEA: Single-sample gene set enrichment analysis
SuperPC: supervised principal components
survival-SVM: survival support vector machine
TCGA: The Cancer Genome Atlas
TME: tumor microenvironment
TOM: topological overlap matrix
TPM: transcripts per kilobase million
WGCNA: Weighted gene co-expression network analysis

## Additional Information and Declarations

### Data Availability

The following information was supplied regarding data availability:

The datasets generated and/or analyzed during the current study are available at NCBI: GSE30219, GSE31210, GSE50081, GSE72094, GSE126044 and GSE135222.
