# Supplementary figures and images for: Development of an immune-related gene signature applying Ridge method for improving immunotherapy responses and clinical outcomes in lung adenocarcinoma
Source: PeerJ. 2025 May 8;13:e19121. doi: 10.7717/peerj.19121 (PMC12066106; doi:10.7717/peerj.19121)

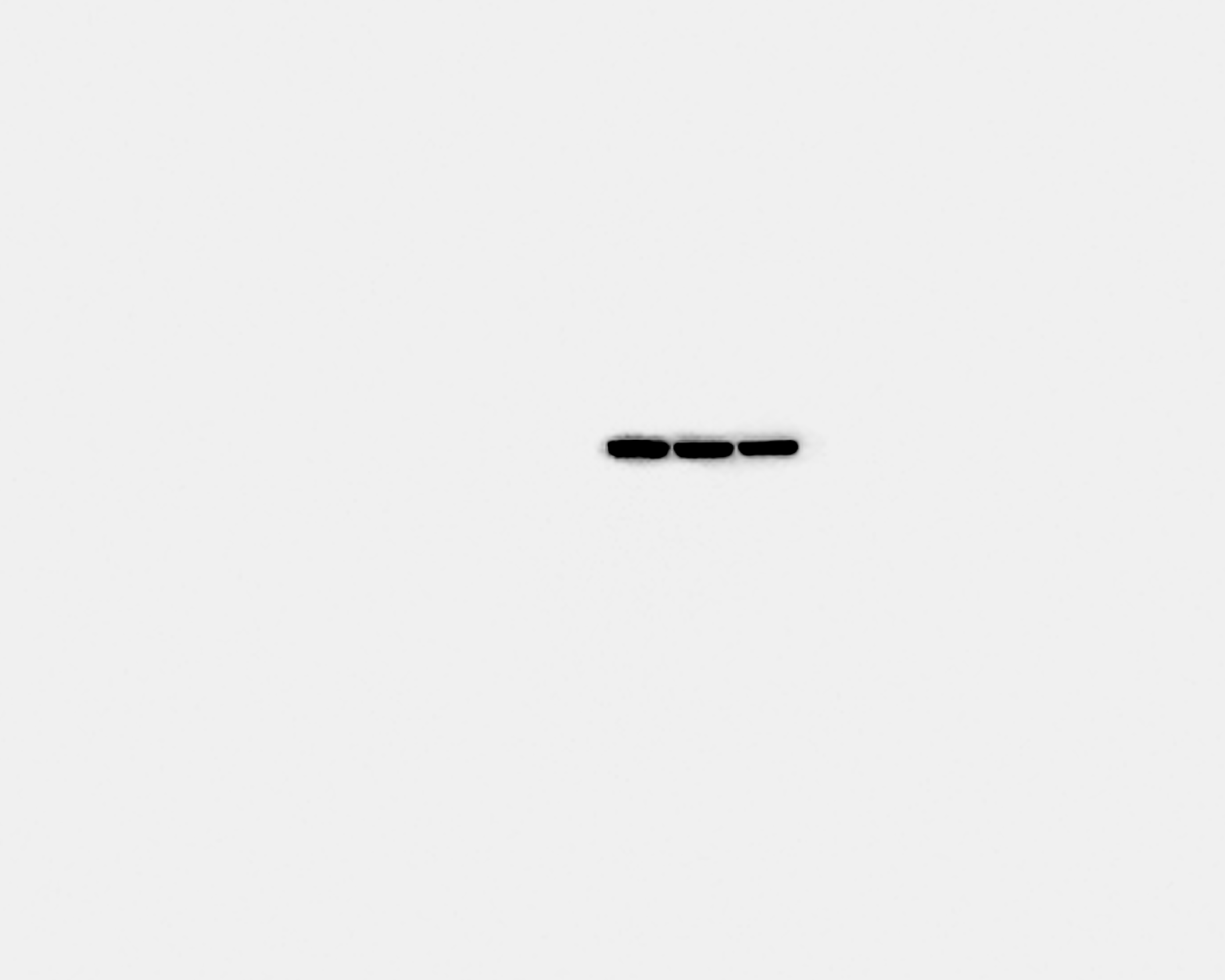

Supplement: Supplemental Information 1 [file peerj-13-19121-s001.zip › Figure 7/WB raw data/BEAS-2B/btnl9+gd 1_1(Chemiluminescence).tif]

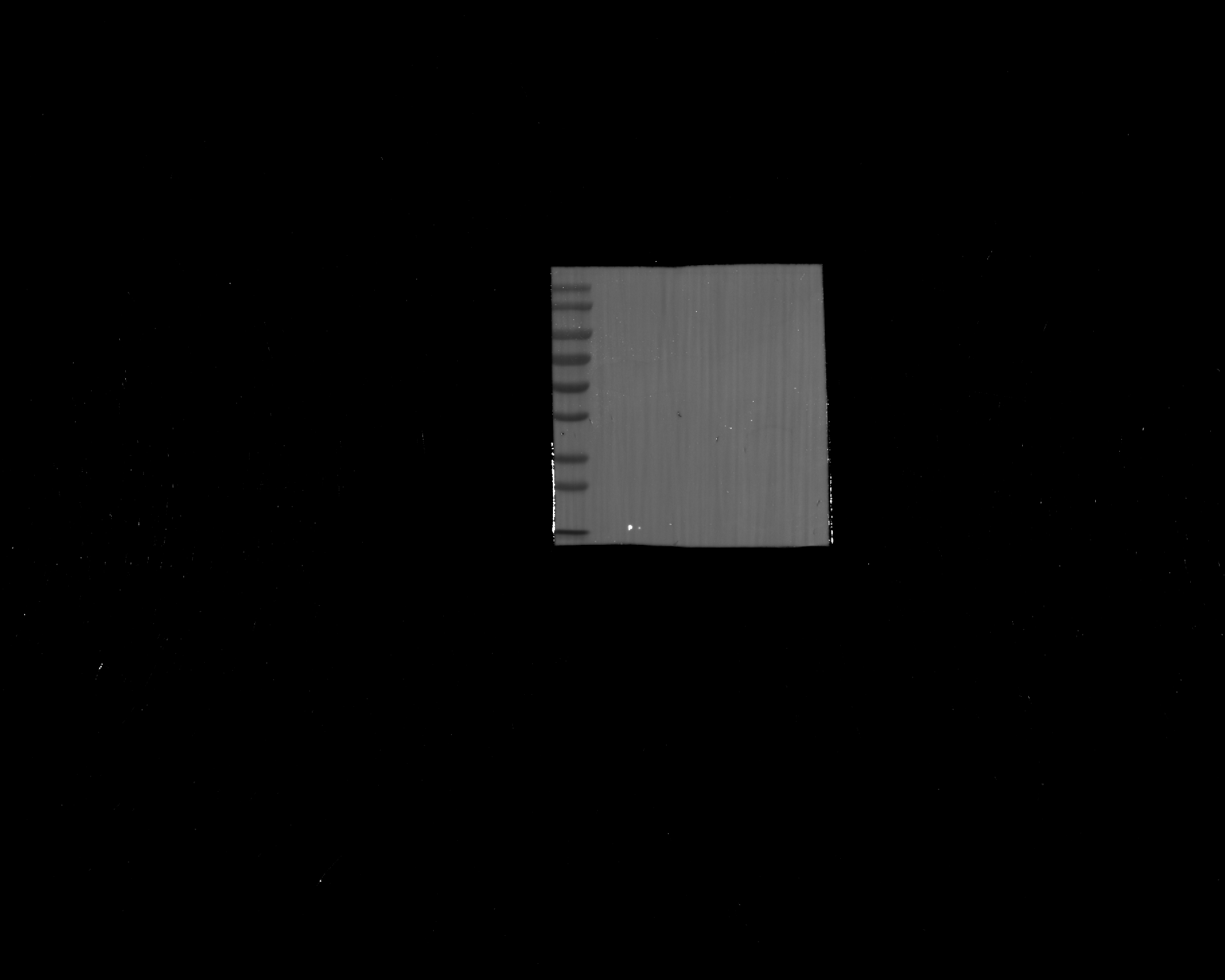

Supplement: Supplemental Information 1 [file peerj-13-19121-s001.zip › Figure 7/WB raw data/BEAS-2B/btnl9+gd 1_1(Colorimetric).tif]

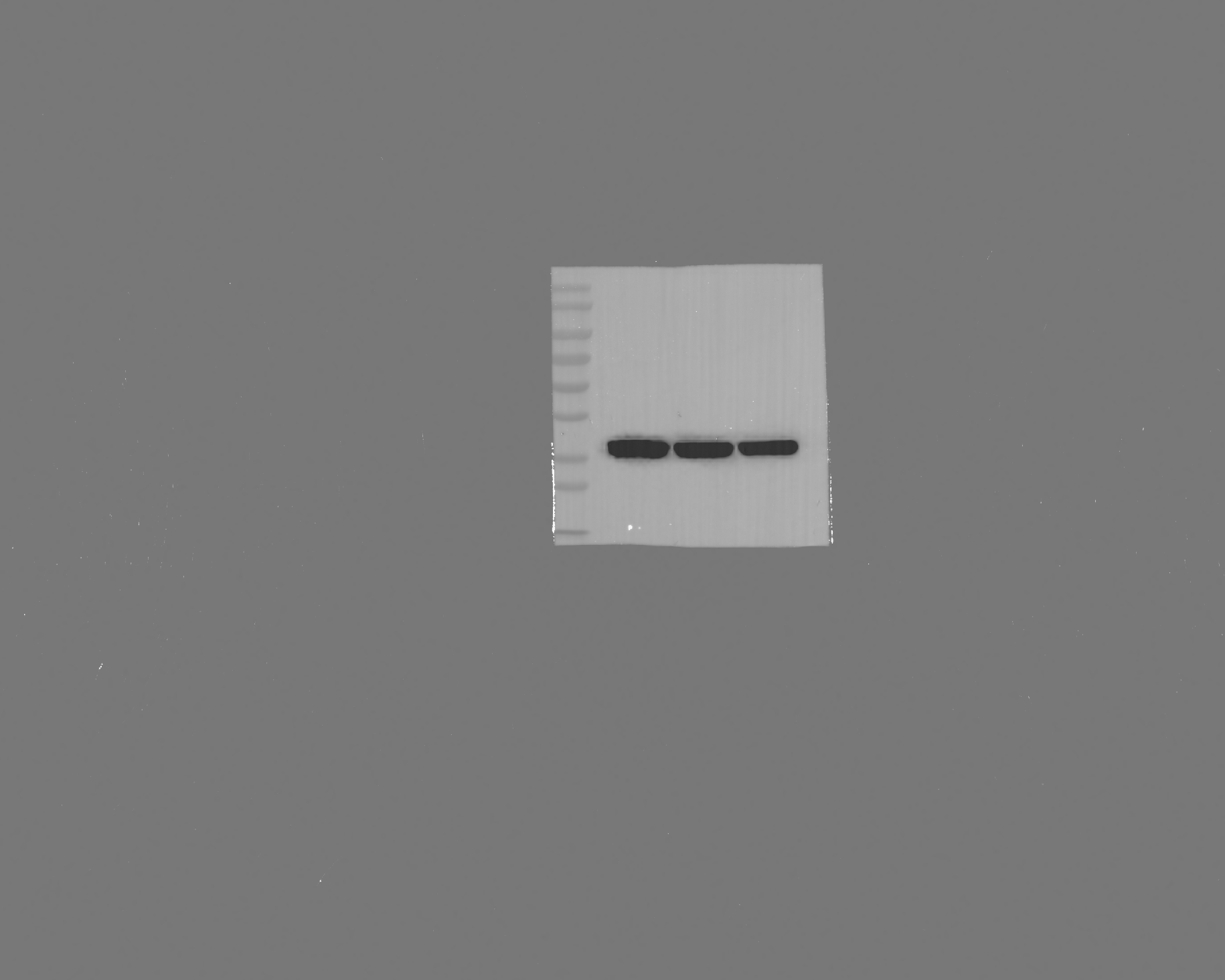

Supplement: Supplemental Information 1 [file peerj-13-19121-s001.zip › Figure 7/WB raw data/BEAS-2B/btnl9+gd 1_1(Composite).tif]

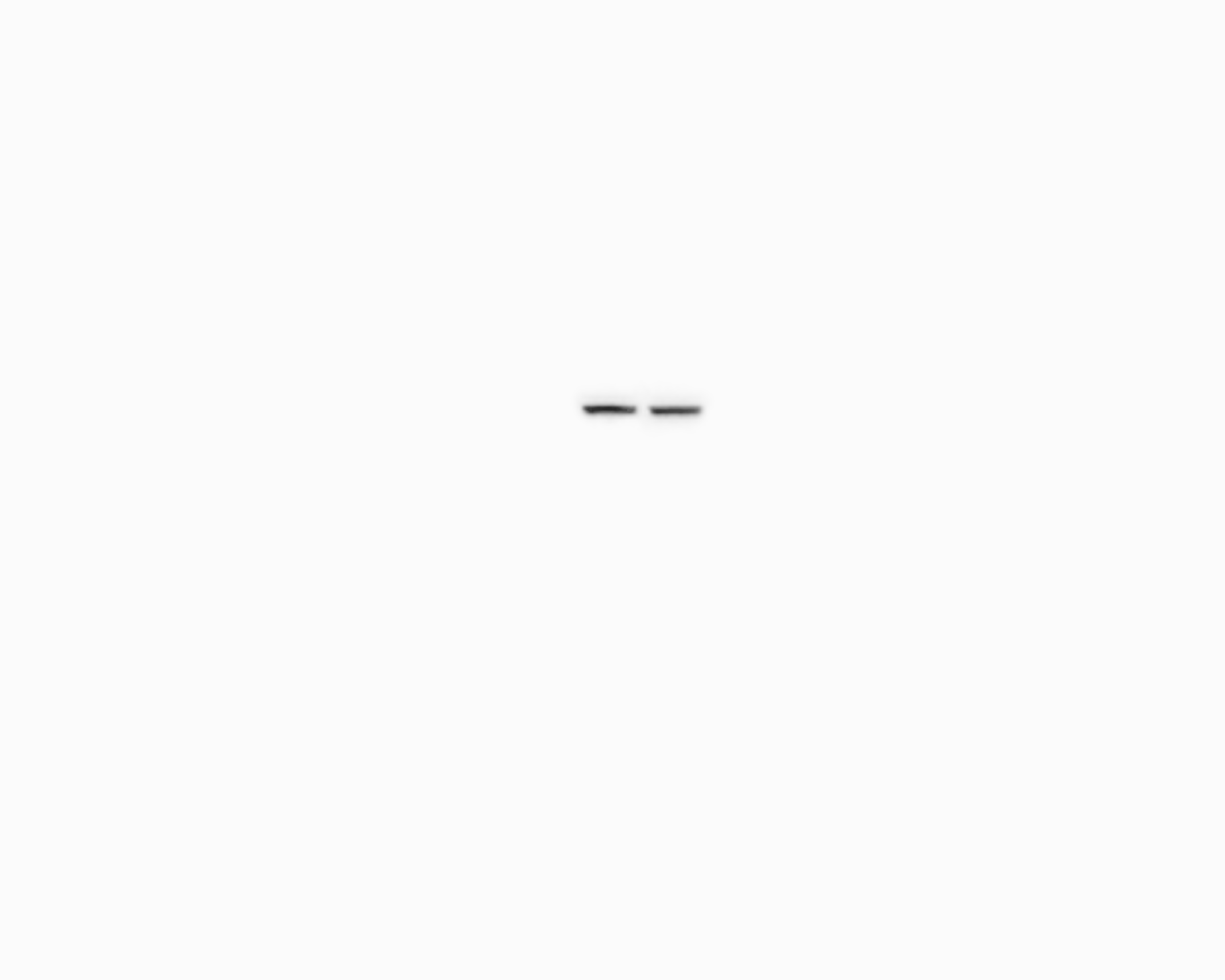

Supplement: Supplemental Information 1 [file peerj-13-19121-s001.zip › Figure 7/WB raw data/BEAS-2B/btnl9+gd 1_2(Chemiluminescence).tif]

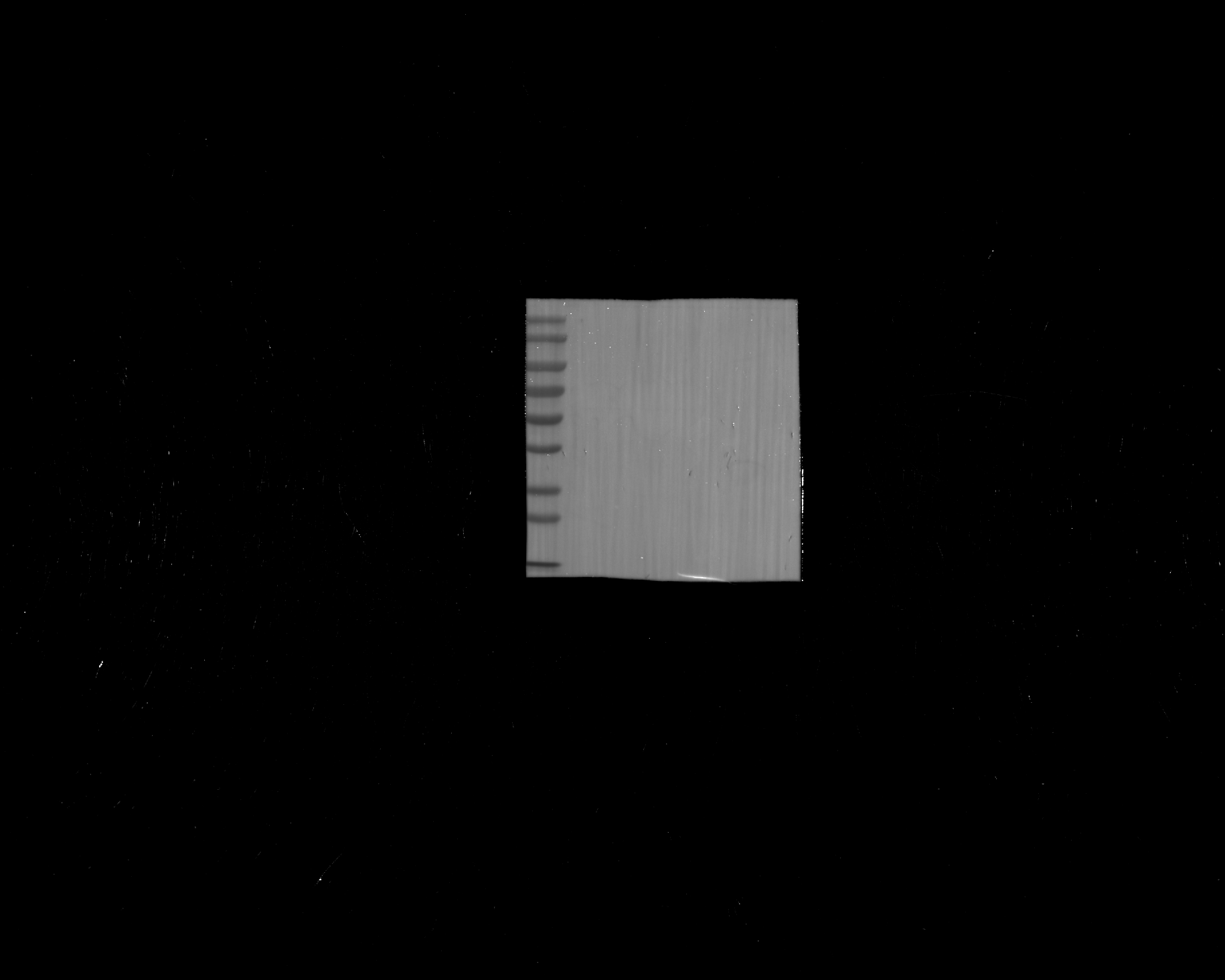

Supplement: Supplemental Information 1 [file peerj-13-19121-s001.zip › Figure 7/WB raw data/BEAS-2B/btnl9+gd 1_2(Colorimetric).tif]

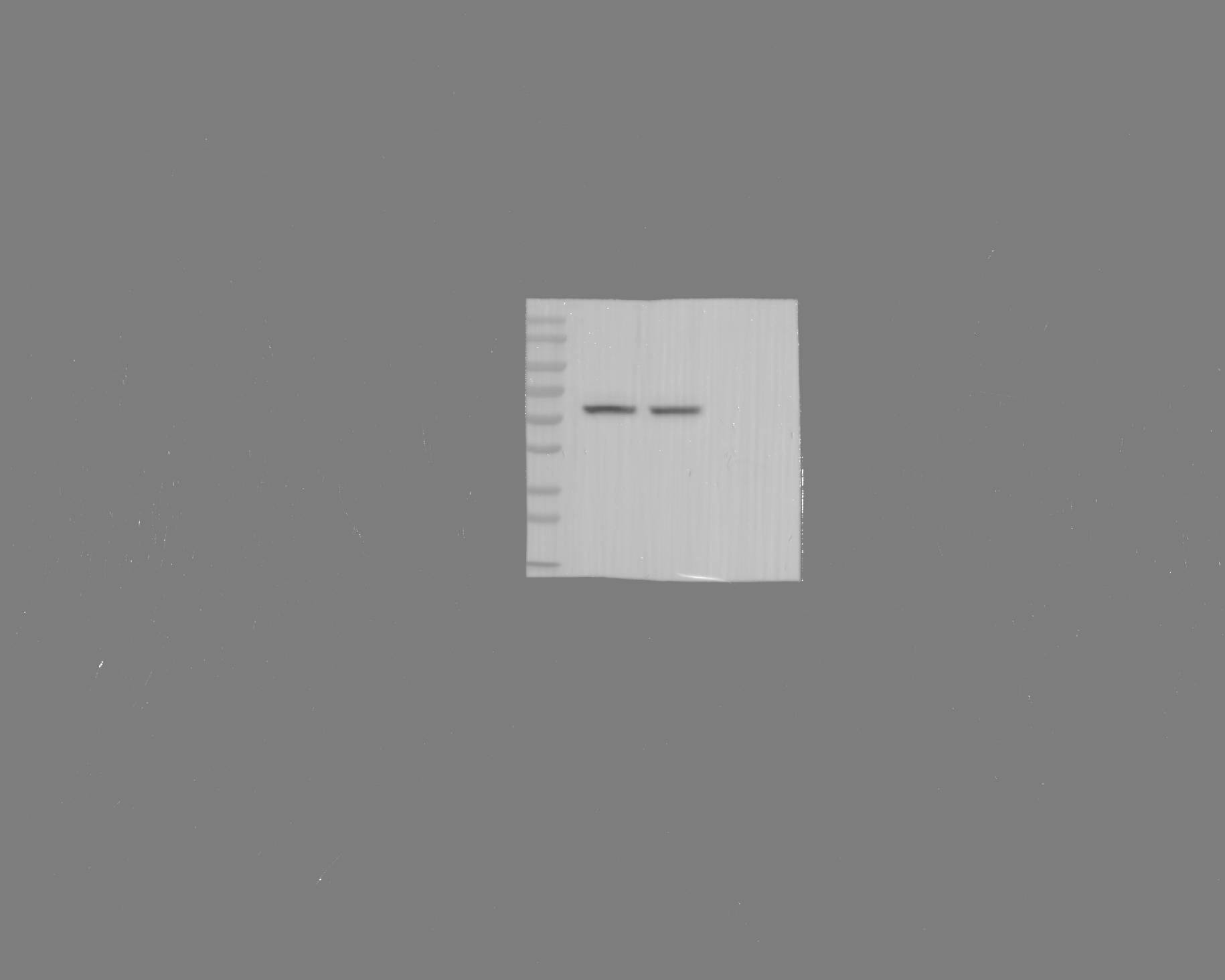

Supplement: Supplemental Information 1 [file peerj-13-19121-s001.zip › Figure 7/WB raw data/BEAS-2B/btnl9+gd 1_2(Composite).tif]

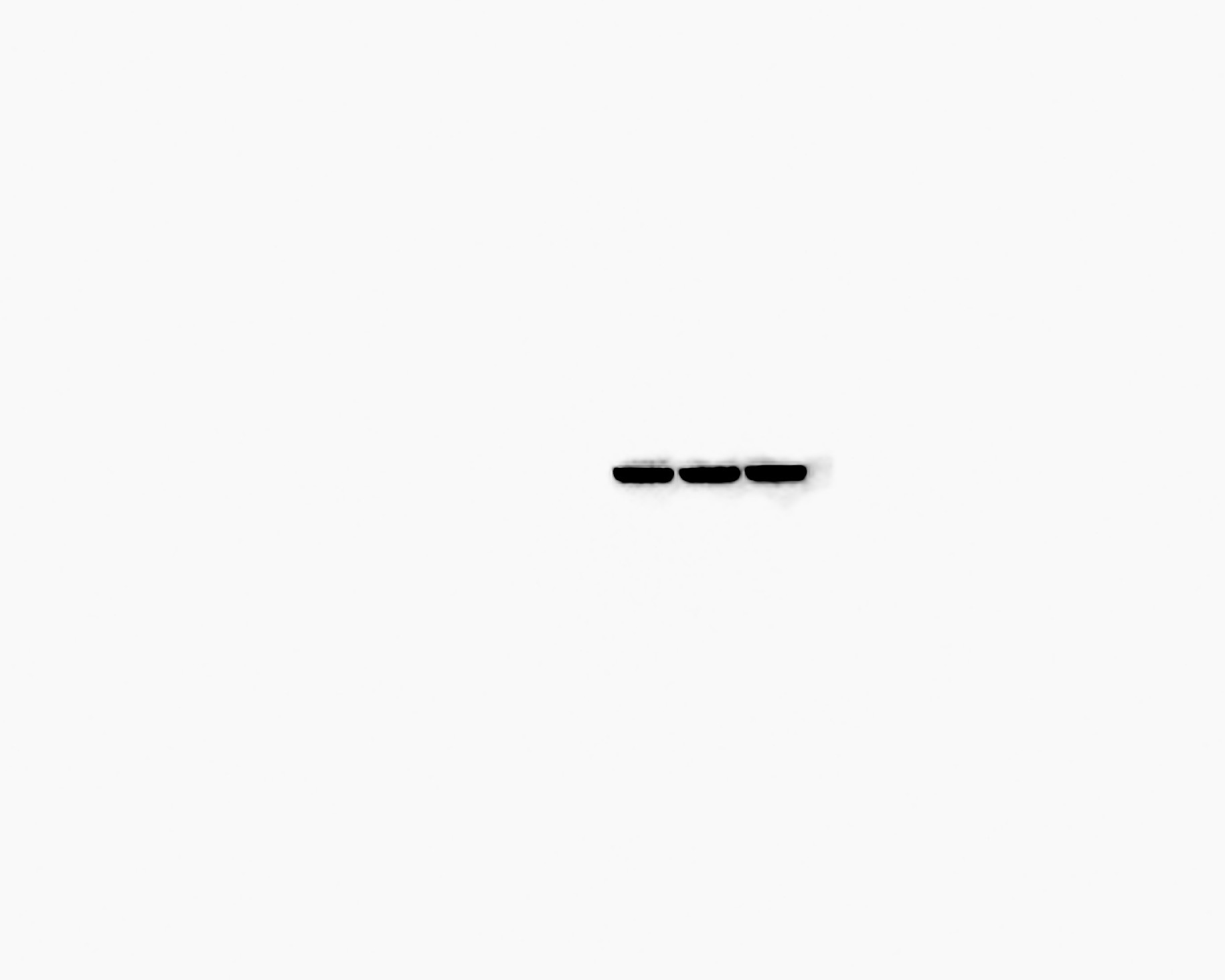

Supplement: Supplemental Information 1 [file peerj-13-19121-s001.zip › Figure 7/WB raw data/BEAS-2B/btnl9+gd 2_1(Chemiluminescence).tif]

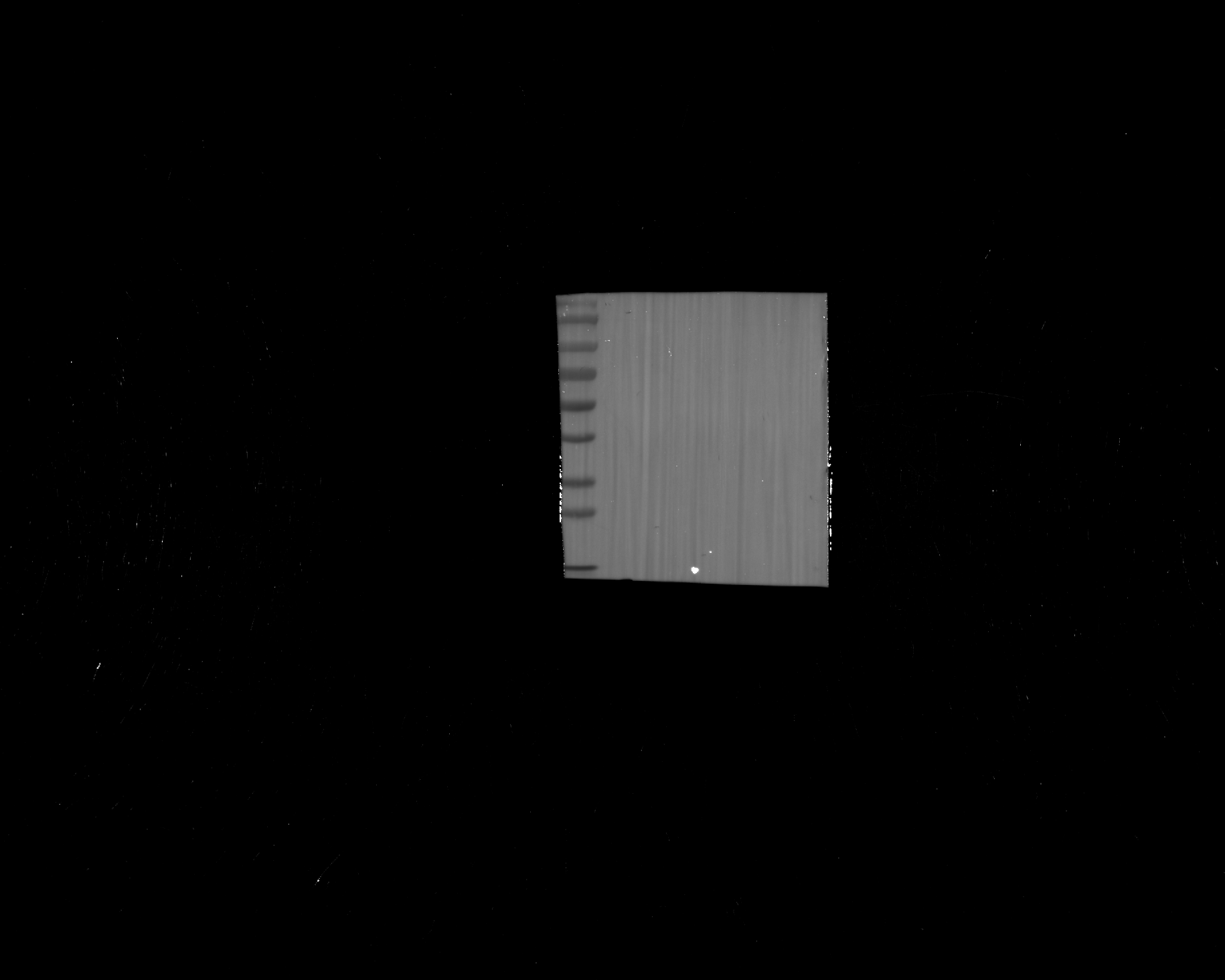

Supplement: Supplemental Information 1 [file peerj-13-19121-s001.zip › Figure 7/WB raw data/BEAS-2B/btnl9+gd 2_1(Colorimetric).tif]

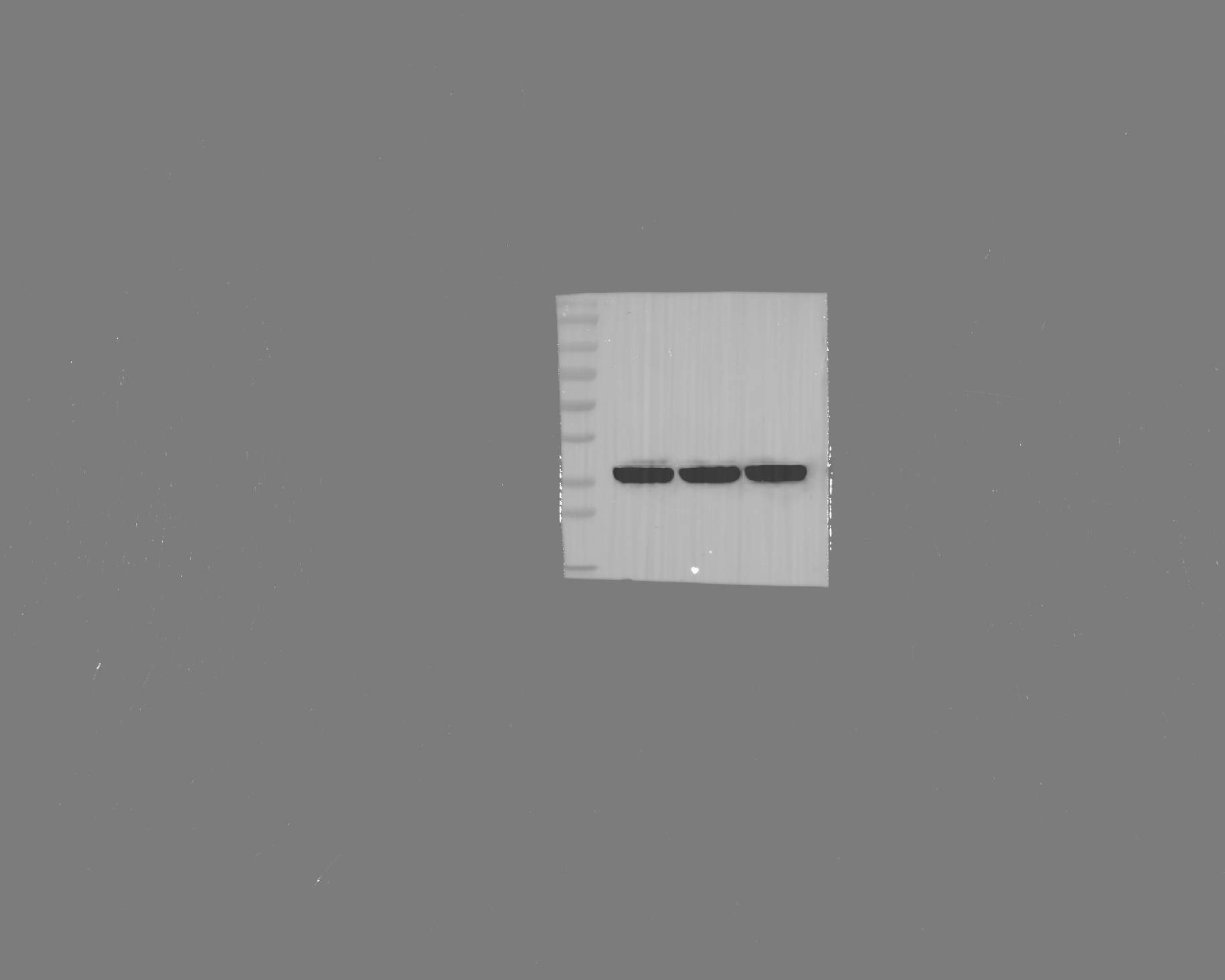

Supplement: Supplemental Information 1 [file peerj-13-19121-s001.zip › Figure 7/WB raw data/BEAS-2B/btnl9+gd 2_1(Composite).tif]

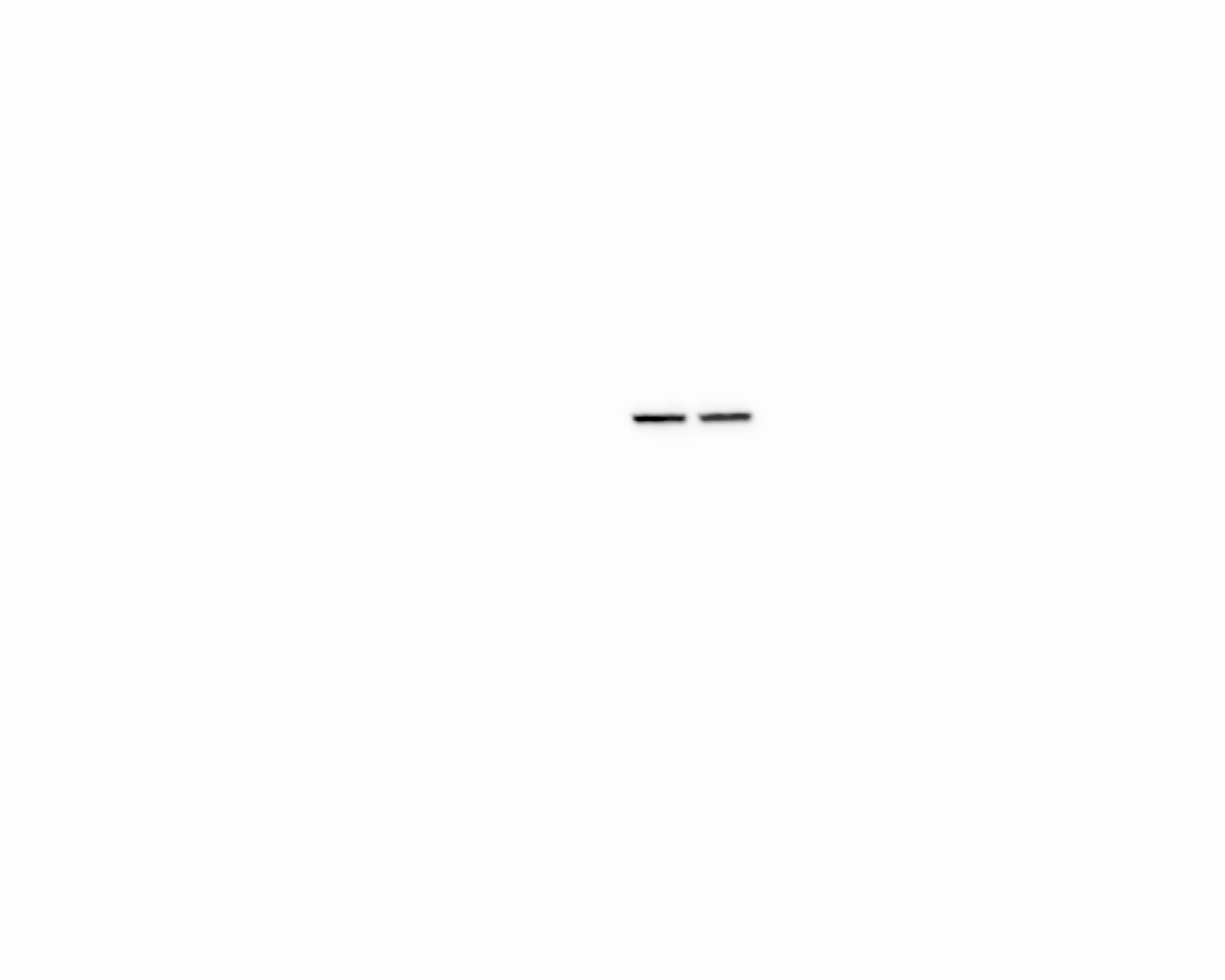

Supplement: Supplemental Information 1 [file peerj-13-19121-s001.zip › Figure 7/WB raw data/BEAS-2B/btnl9+gd 2_2(Chemiluminescence).tif]

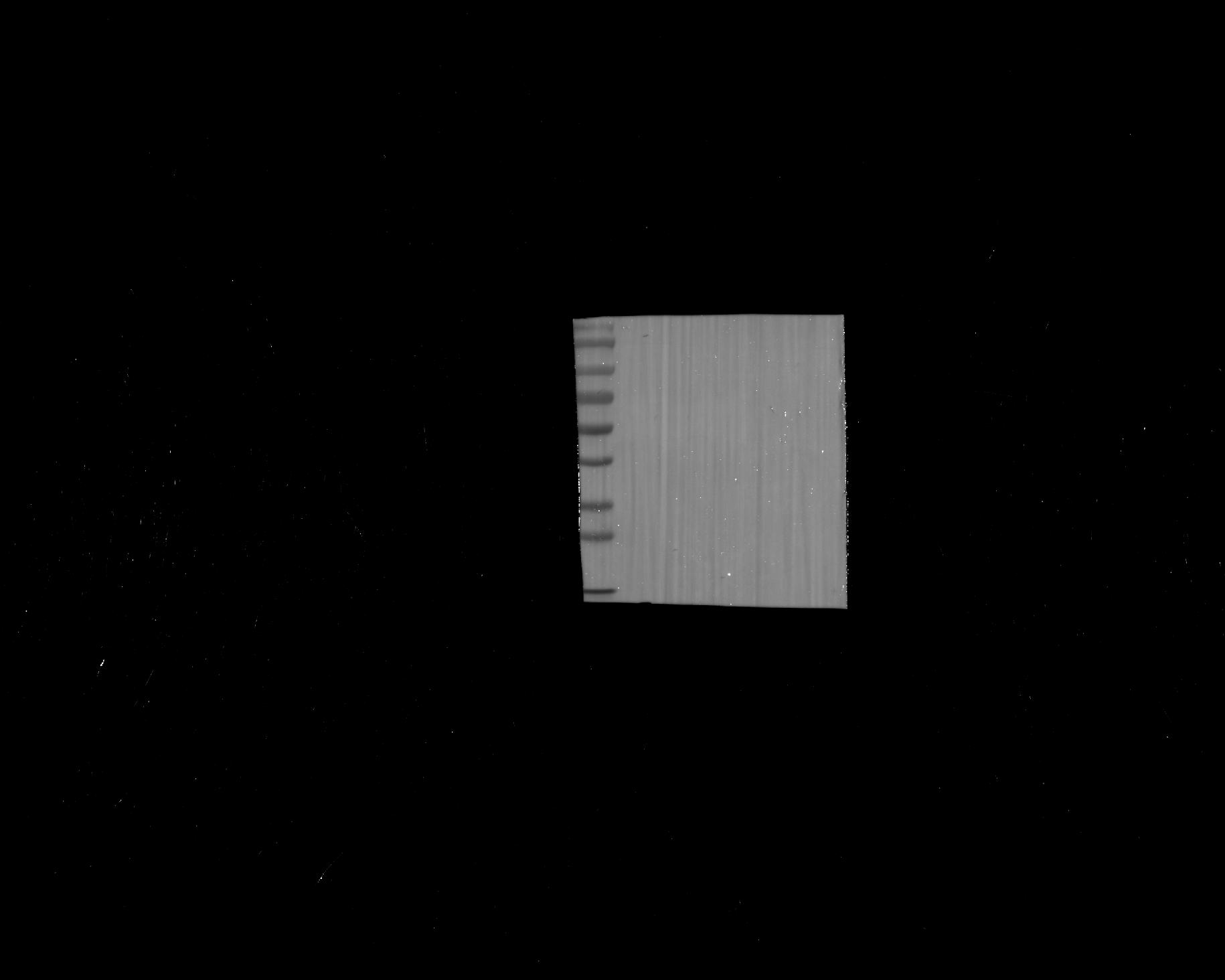

Supplement: Supplemental Information 1 [file peerj-13-19121-s001.zip › Figure 7/WB raw data/BEAS-2B/btnl9+gd 2_2(Colorimetric).tif]

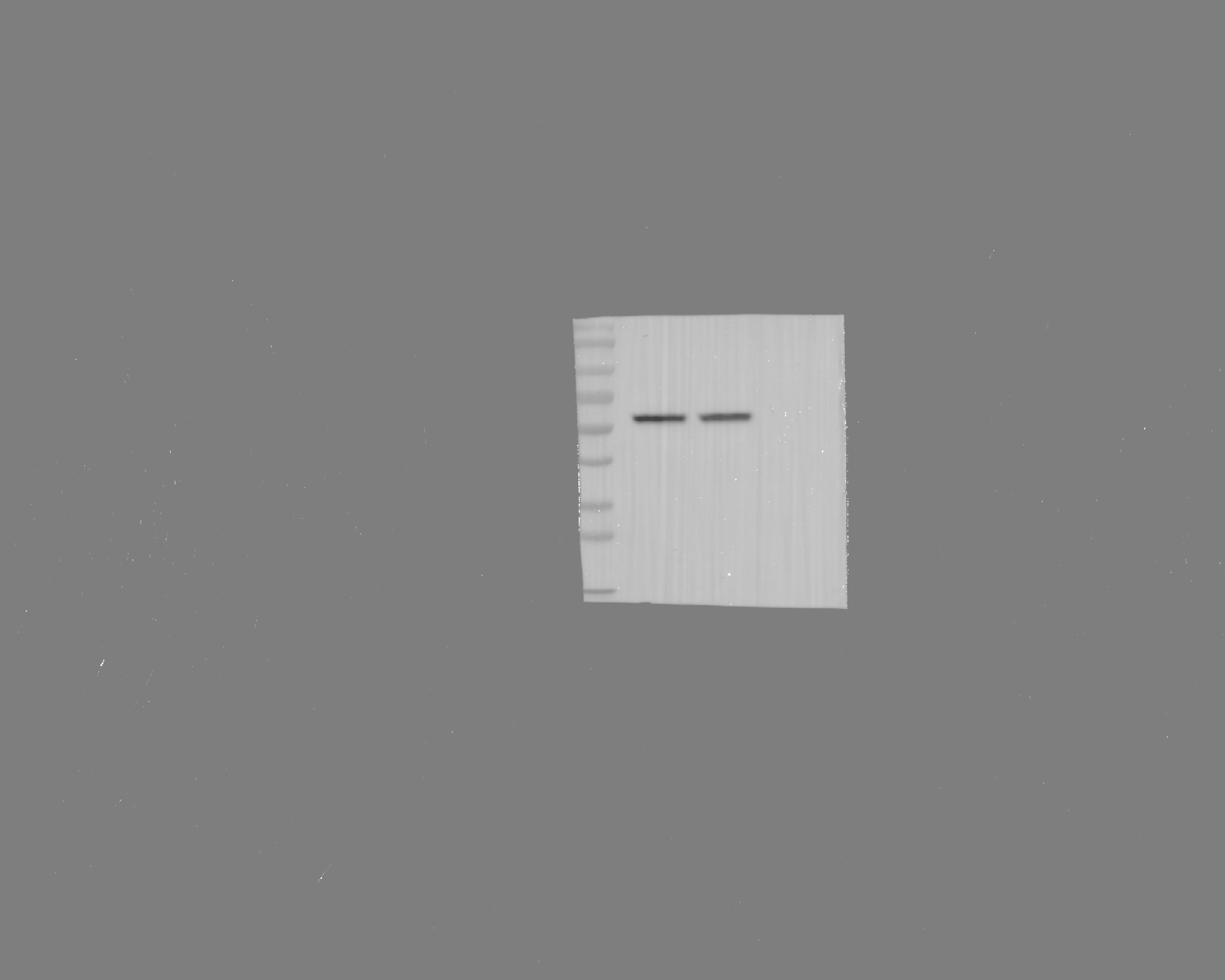

Supplement: Supplemental Information 1 [file peerj-13-19121-s001.zip › Figure 7/WB raw data/BEAS-2B/btnl9+gd 2_2(Composite).tif]

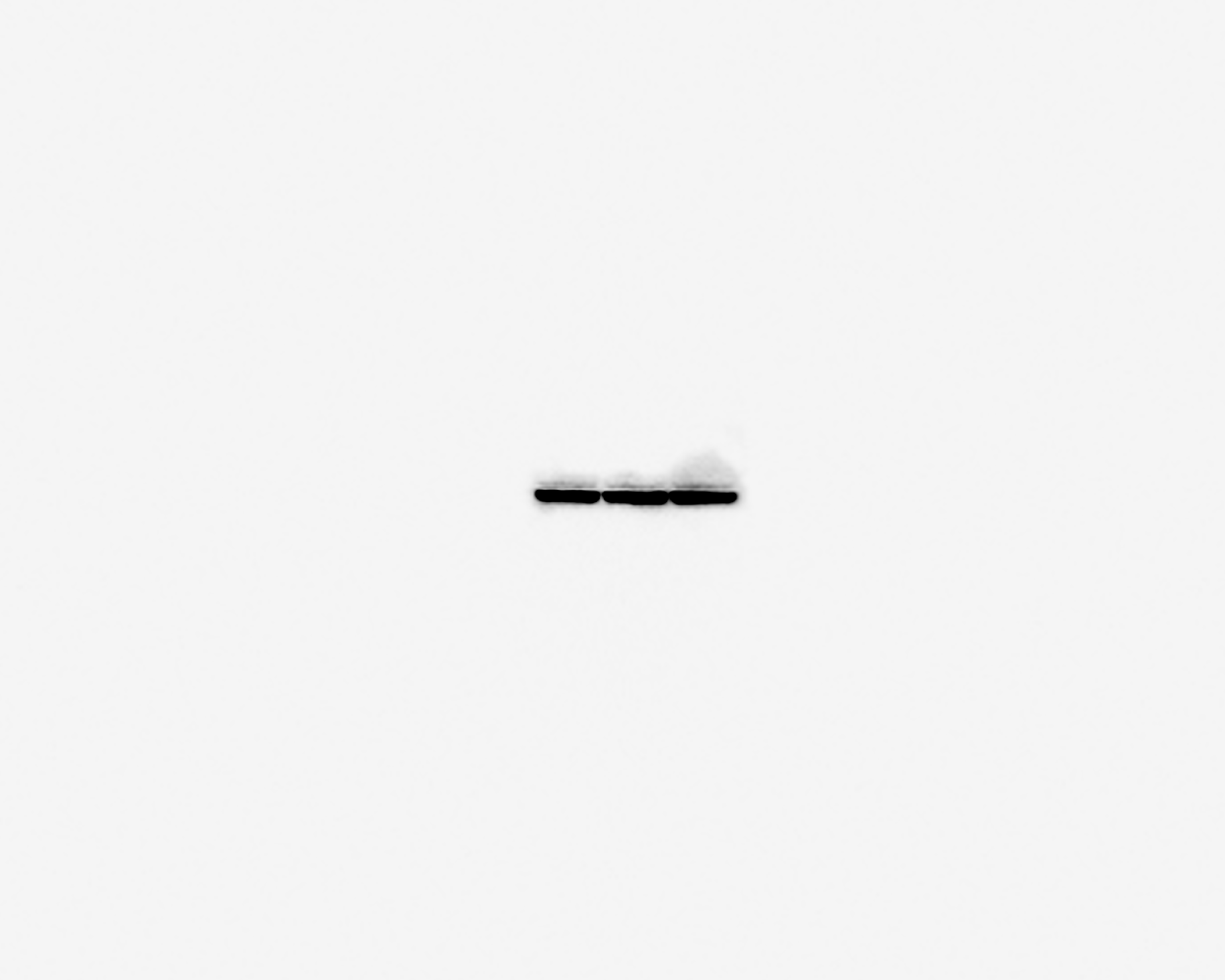

Supplement: Supplemental Information 1 [file peerj-13-19121-s001.zip › Figure 7/WB raw data/BEAS-2B/btnl9+gd 3_1(Chemiluminescence).tif]

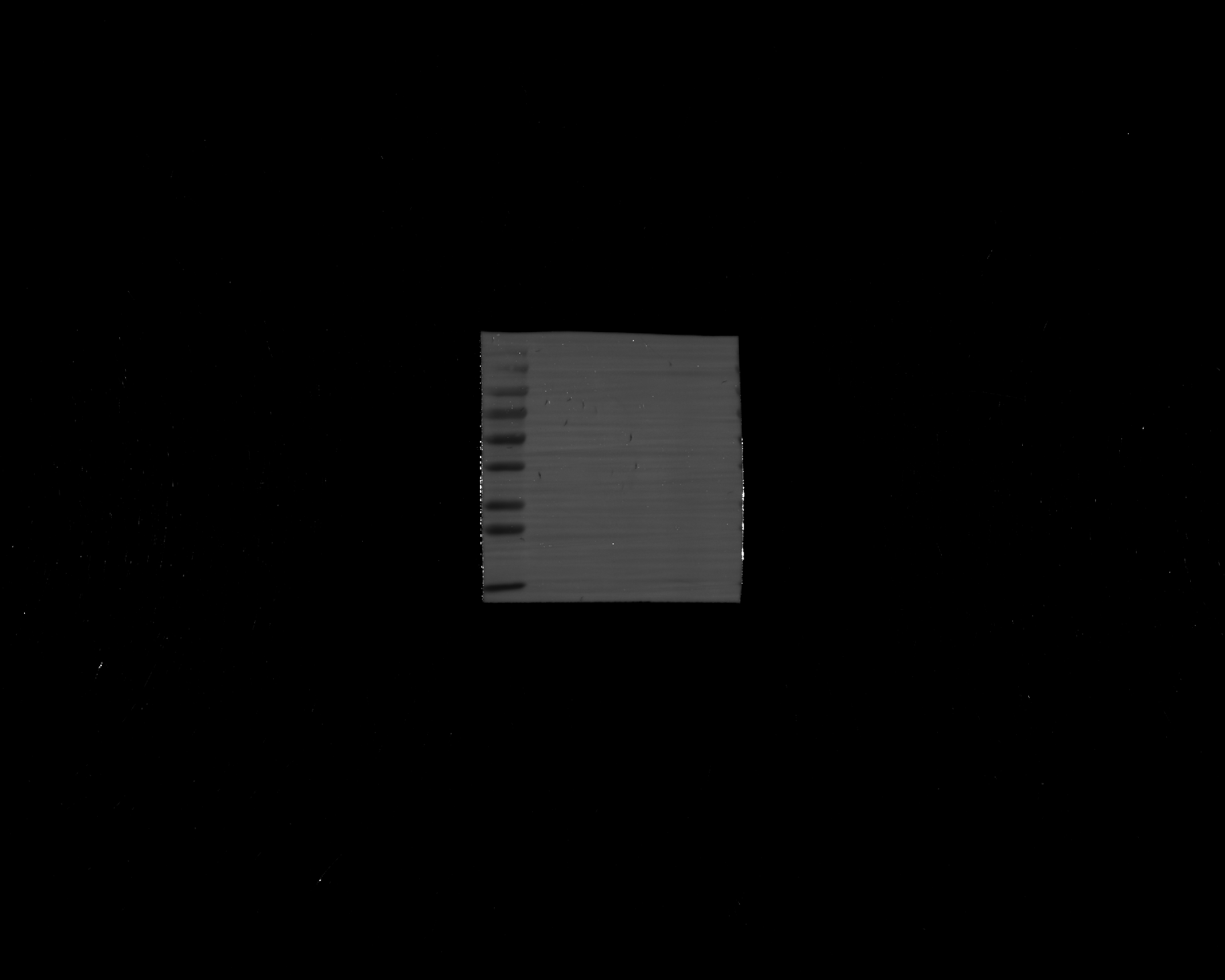

Supplement: Supplemental Information 1 [file peerj-13-19121-s001.zip › Figure 7/WB raw data/BEAS-2B/btnl9+gd 3_1(Colorimetric).tif]

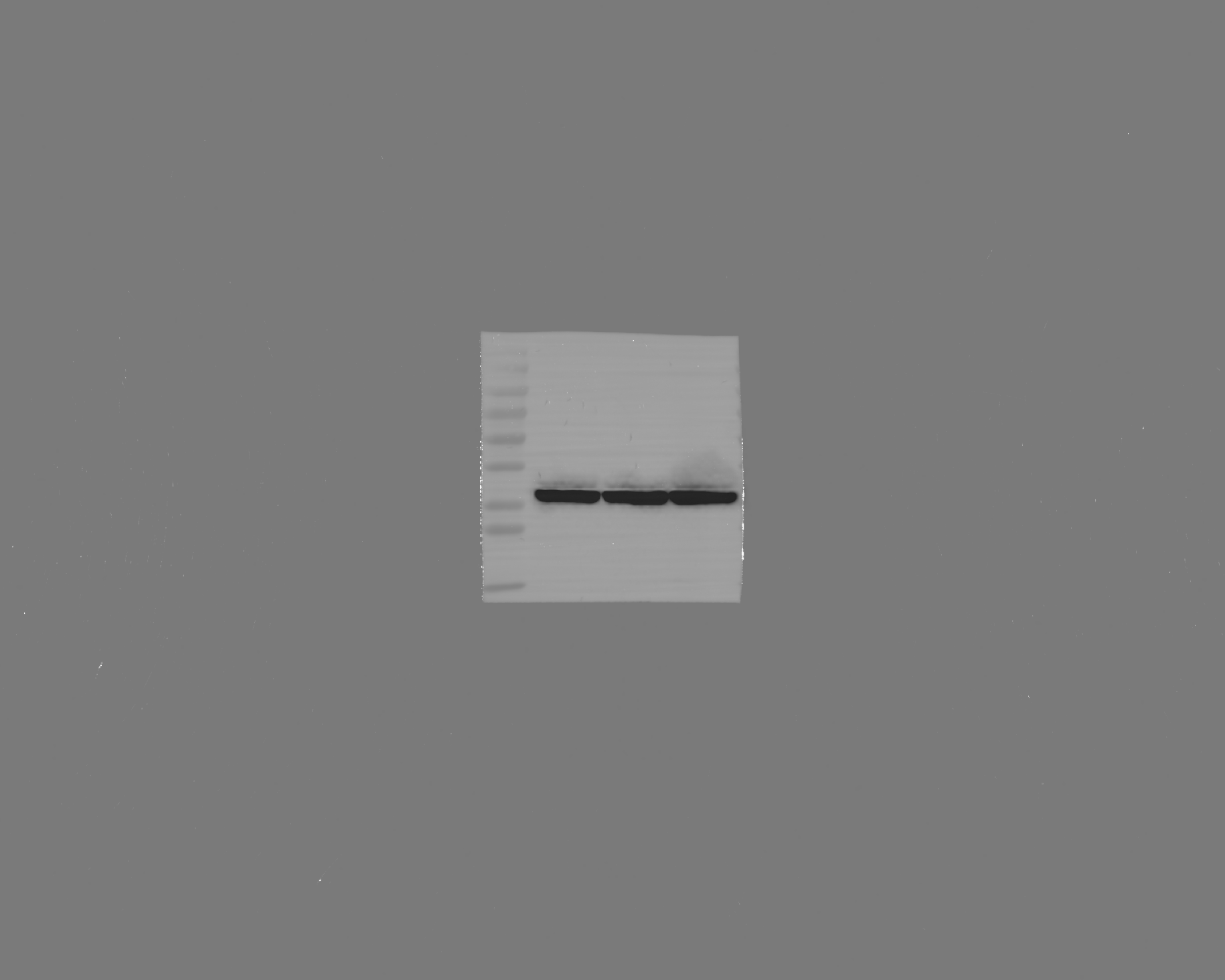

Supplement: Supplemental Information 1 [file peerj-13-19121-s001.zip › Figure 7/WB raw data/BEAS-2B/btnl9+gd 3_1(Composite).tif]

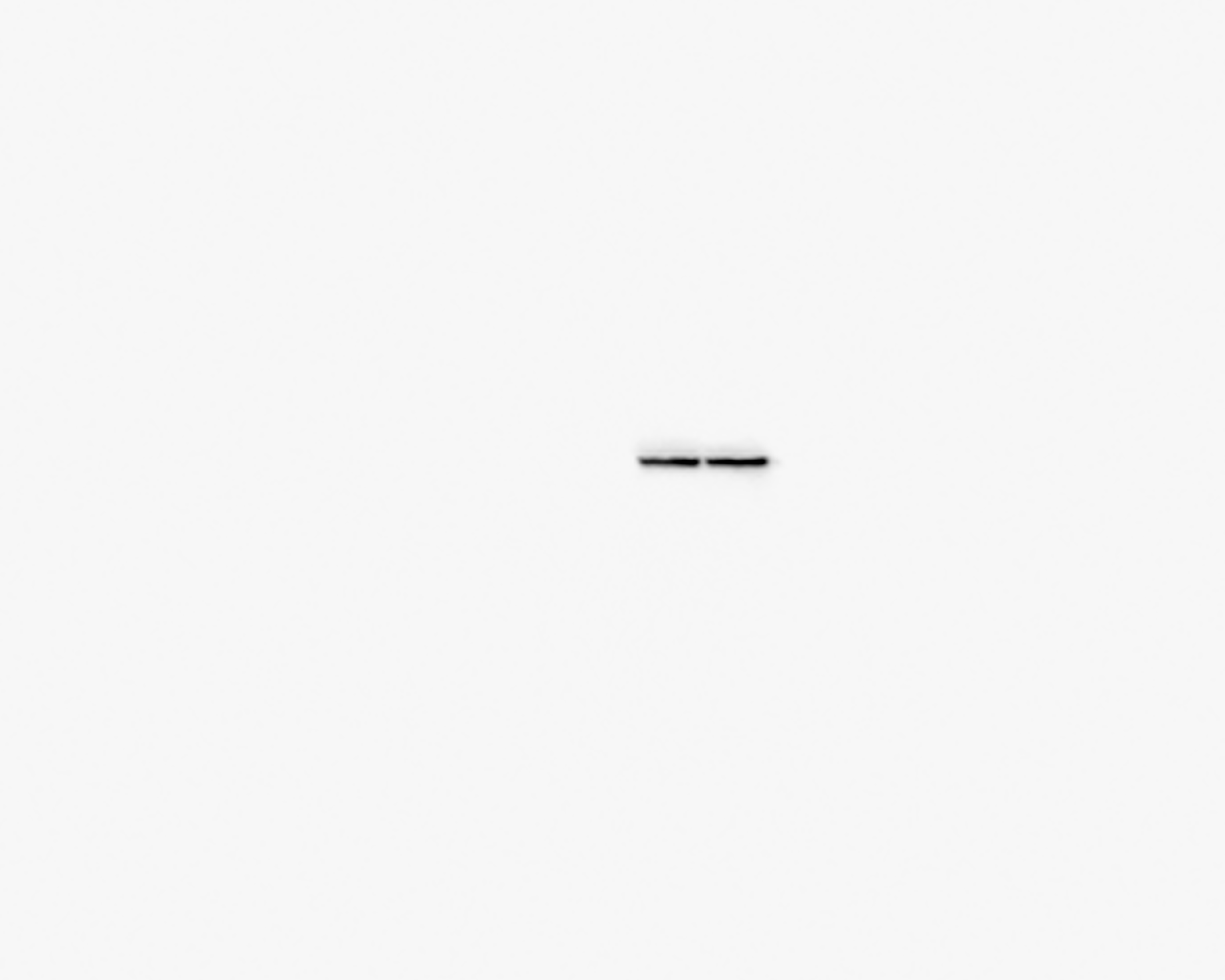

Supplement: Supplemental Information 1 [file peerj-13-19121-s001.zip › Figure 7/WB raw data/BEAS-2B/btnl9+gd 3_2(Chemiluminescence).tif]

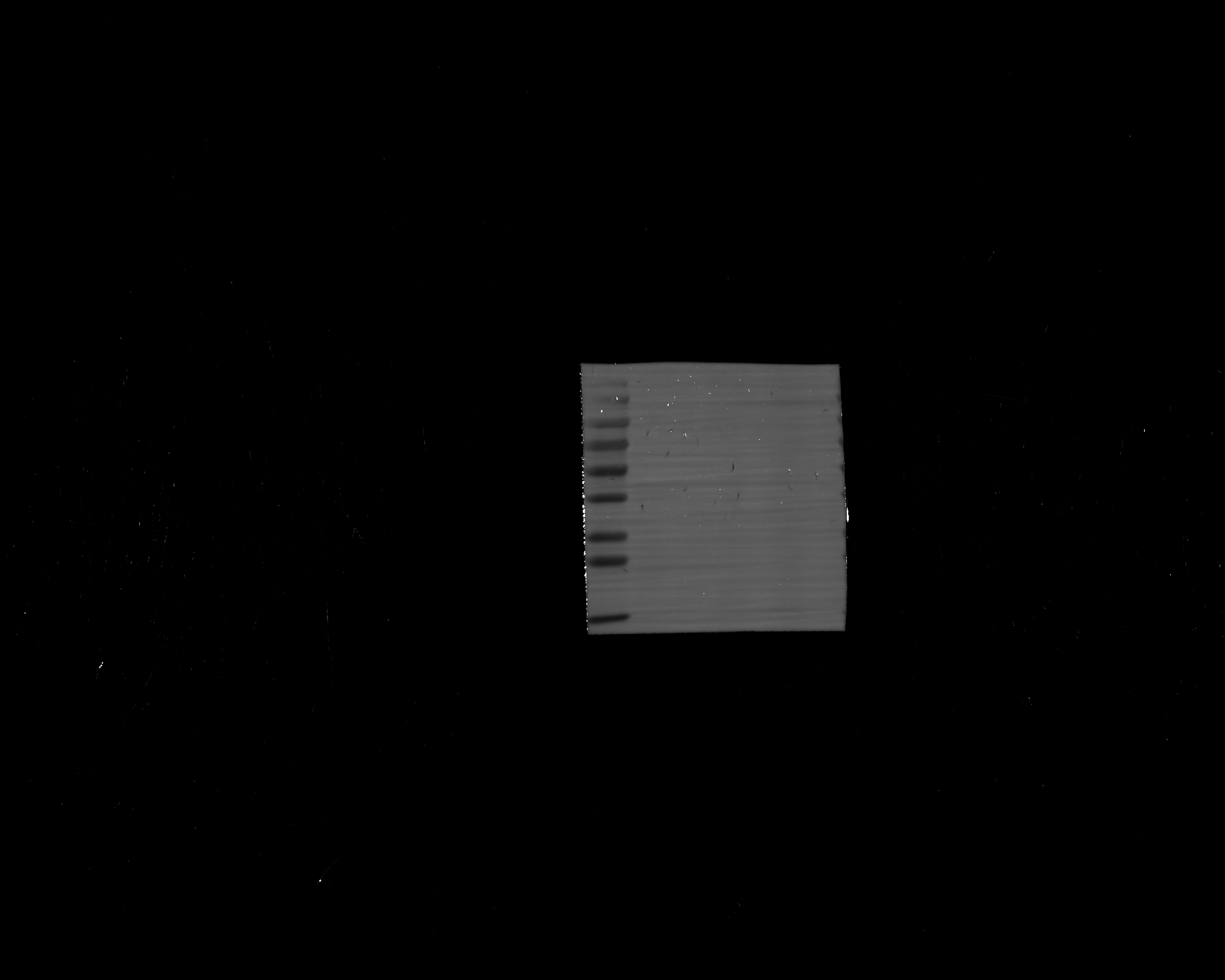

Supplement: Supplemental Information 1 [file peerj-13-19121-s001.zip › Figure 7/WB raw data/BEAS-2B/btnl9+gd 3_2(Colorimetric).tif]

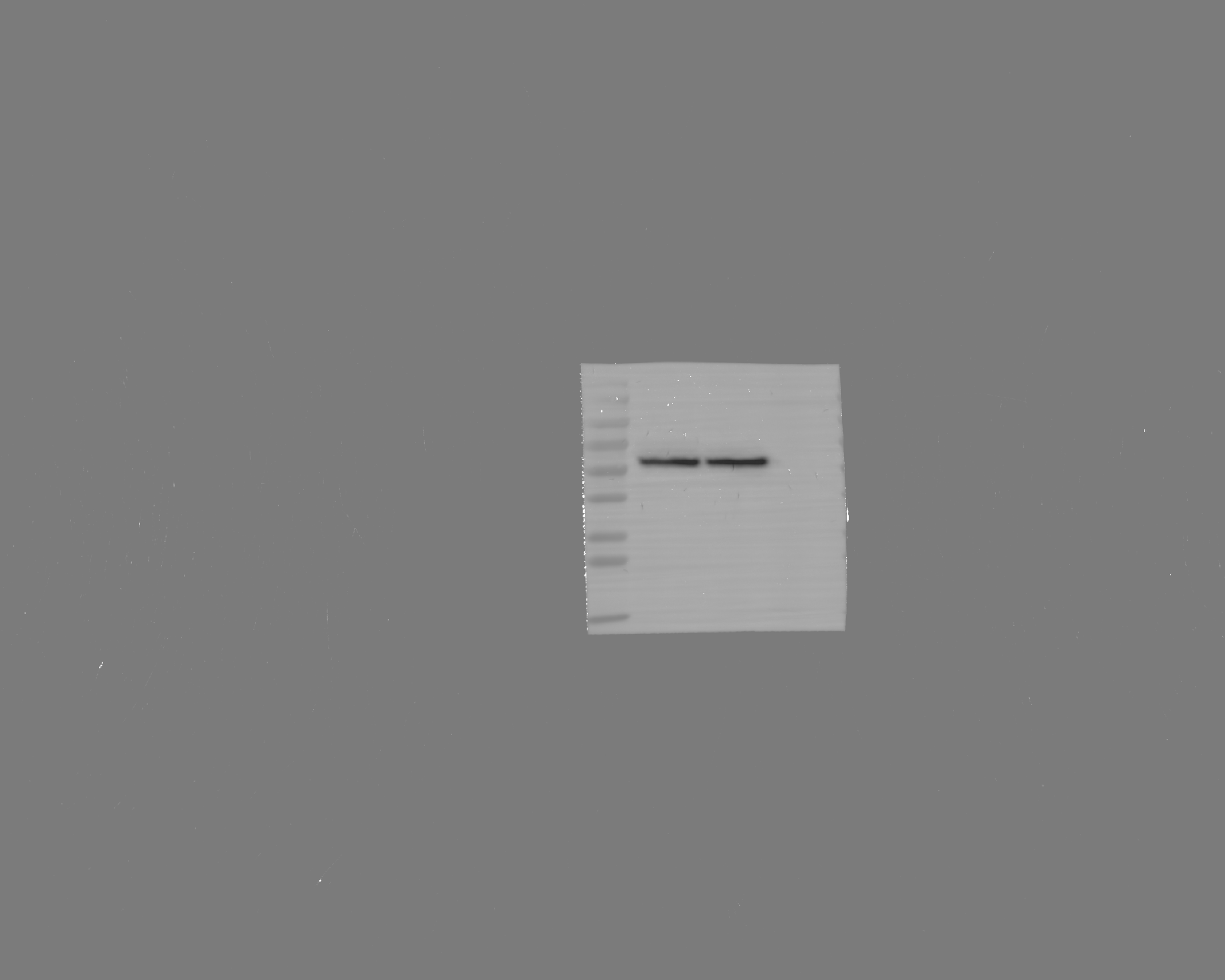

Supplement: Supplemental Information 1 [file peerj-13-19121-s001.zip › Figure 7/WB raw data/BEAS-2B/btnl9+gd 3_2(Composite).tif]

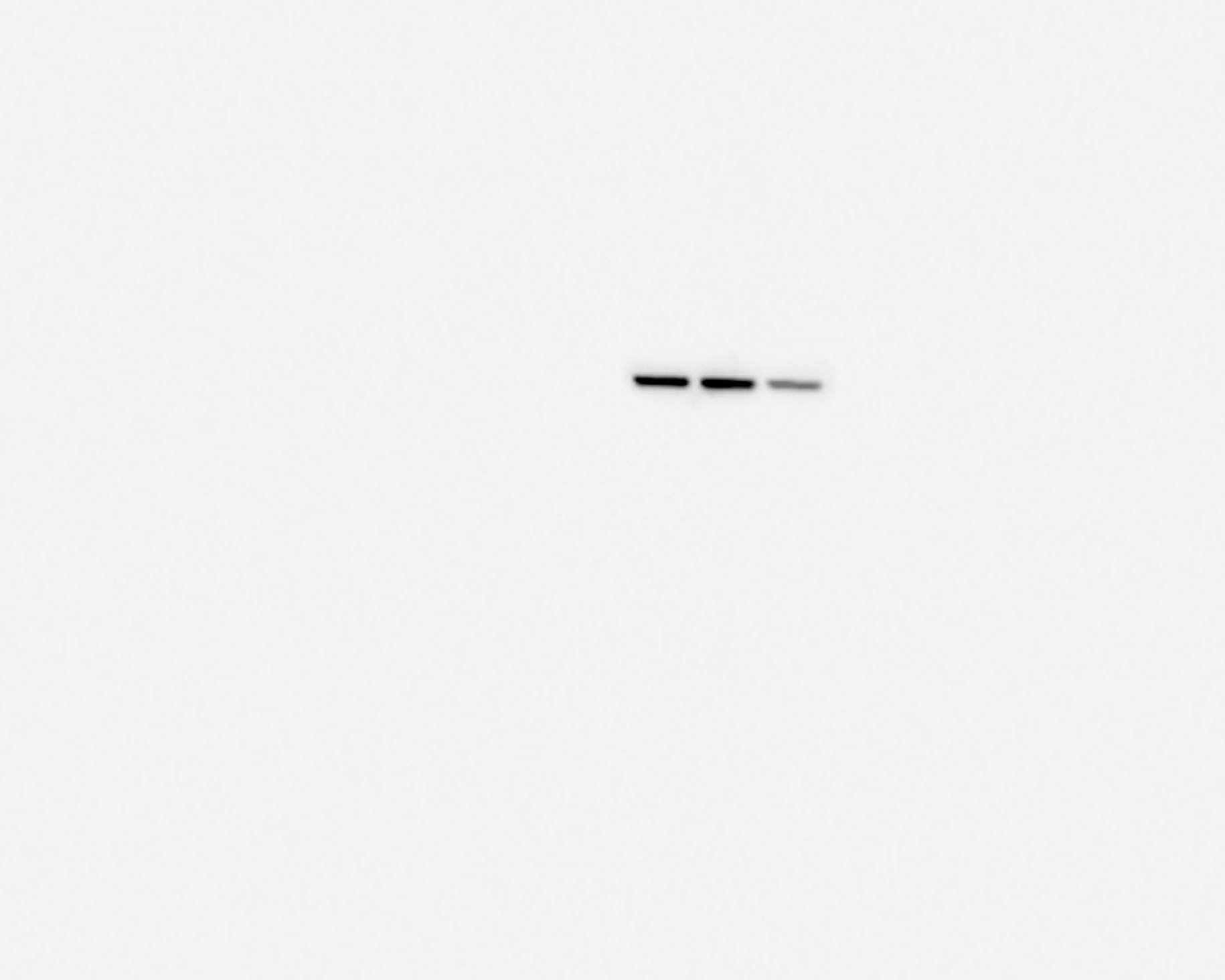

Supplement: Supplemental Information 1 [file peerj-13-19121-s001.zip › Figure 7/WB raw data/H1395/btnl9+gd 7_1(Chemiluminescence).tif]

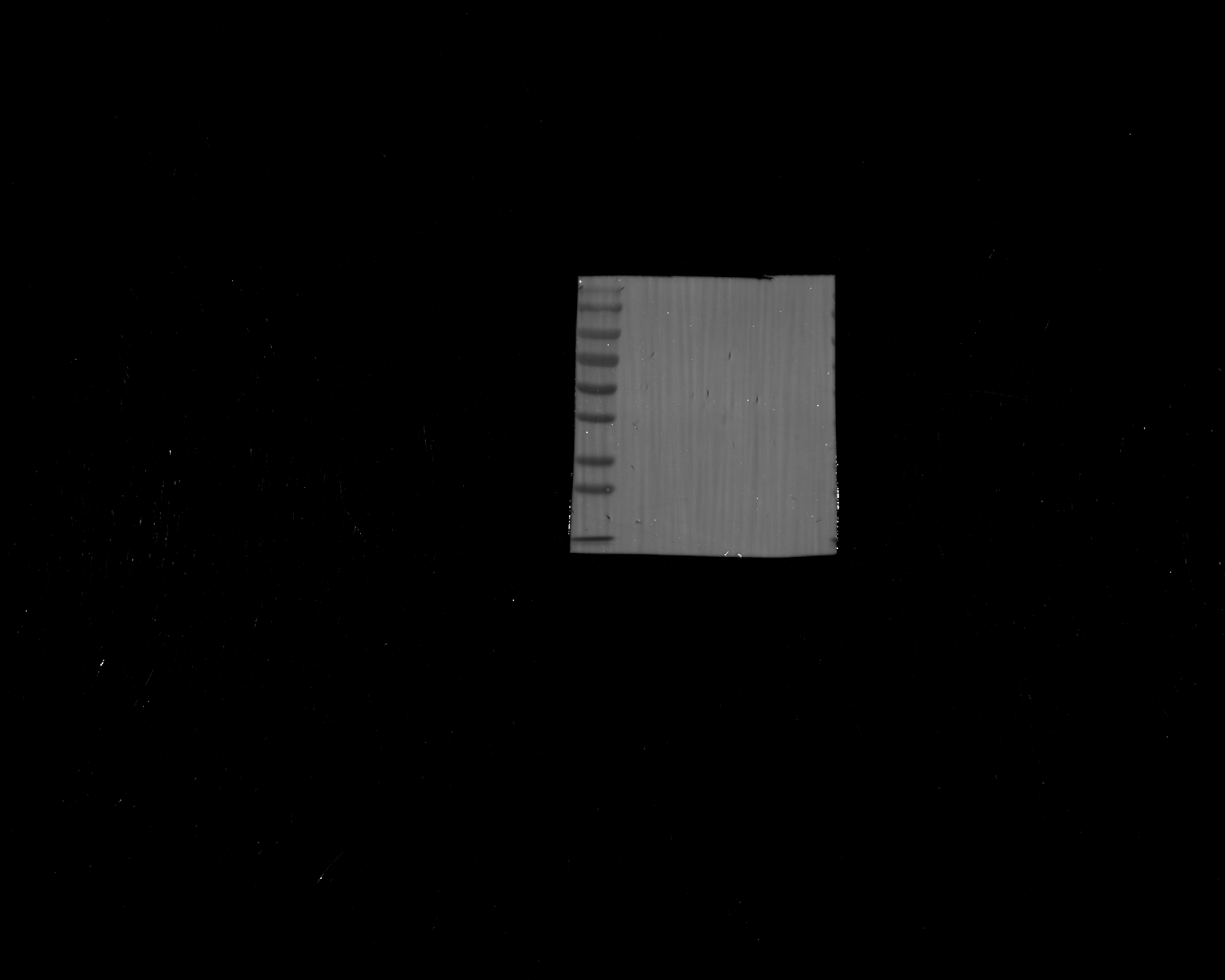

Supplement: Supplemental Information 1 [file peerj-13-19121-s001.zip › Figure 7/WB raw data/H1395/btnl9+gd 7_1(Colorimetric).tif]

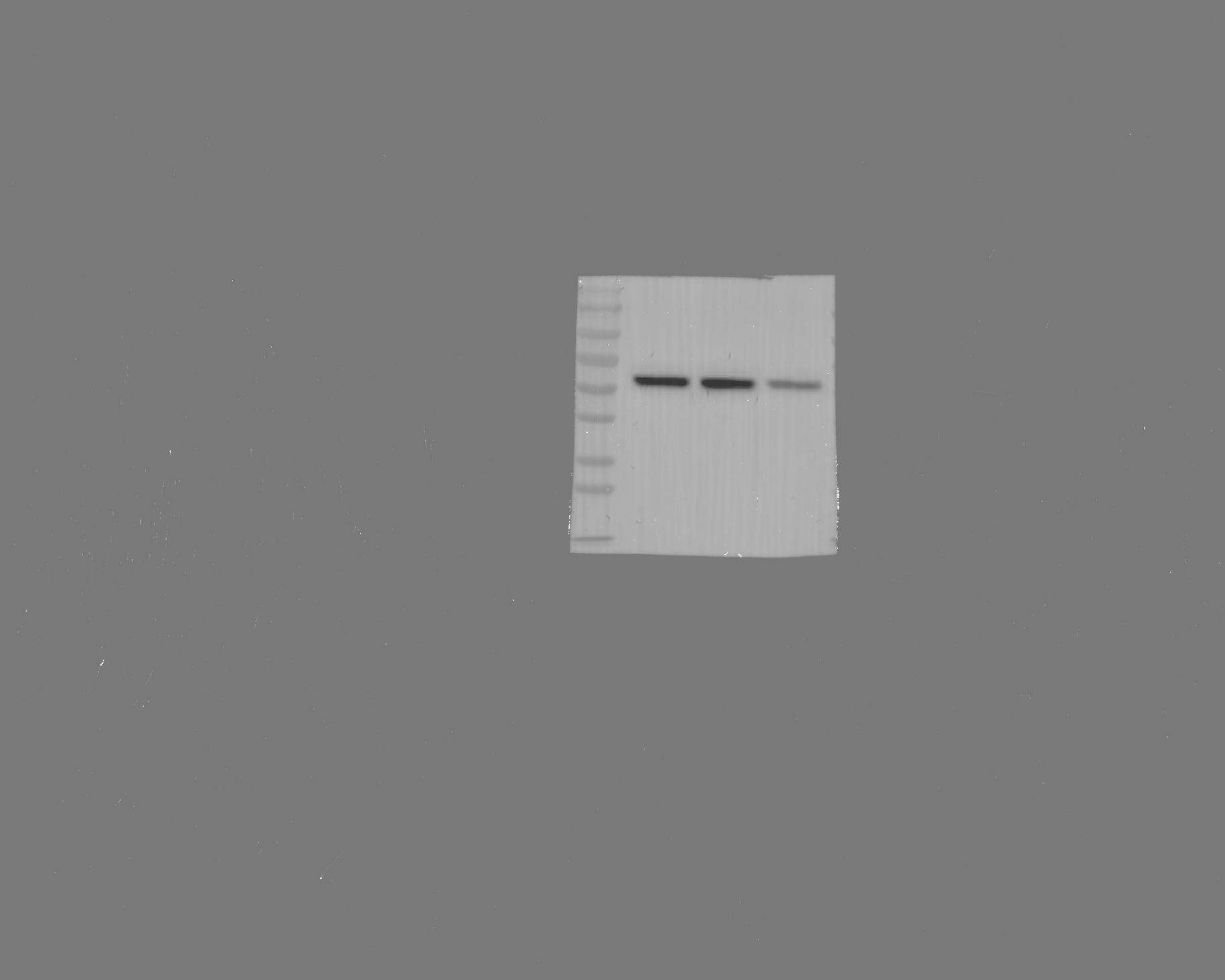

Supplement: Supplemental Information 1 [file peerj-13-19121-s001.zip › Figure 7/WB raw data/H1395/btnl9+gd 7_1(Composite).tif]

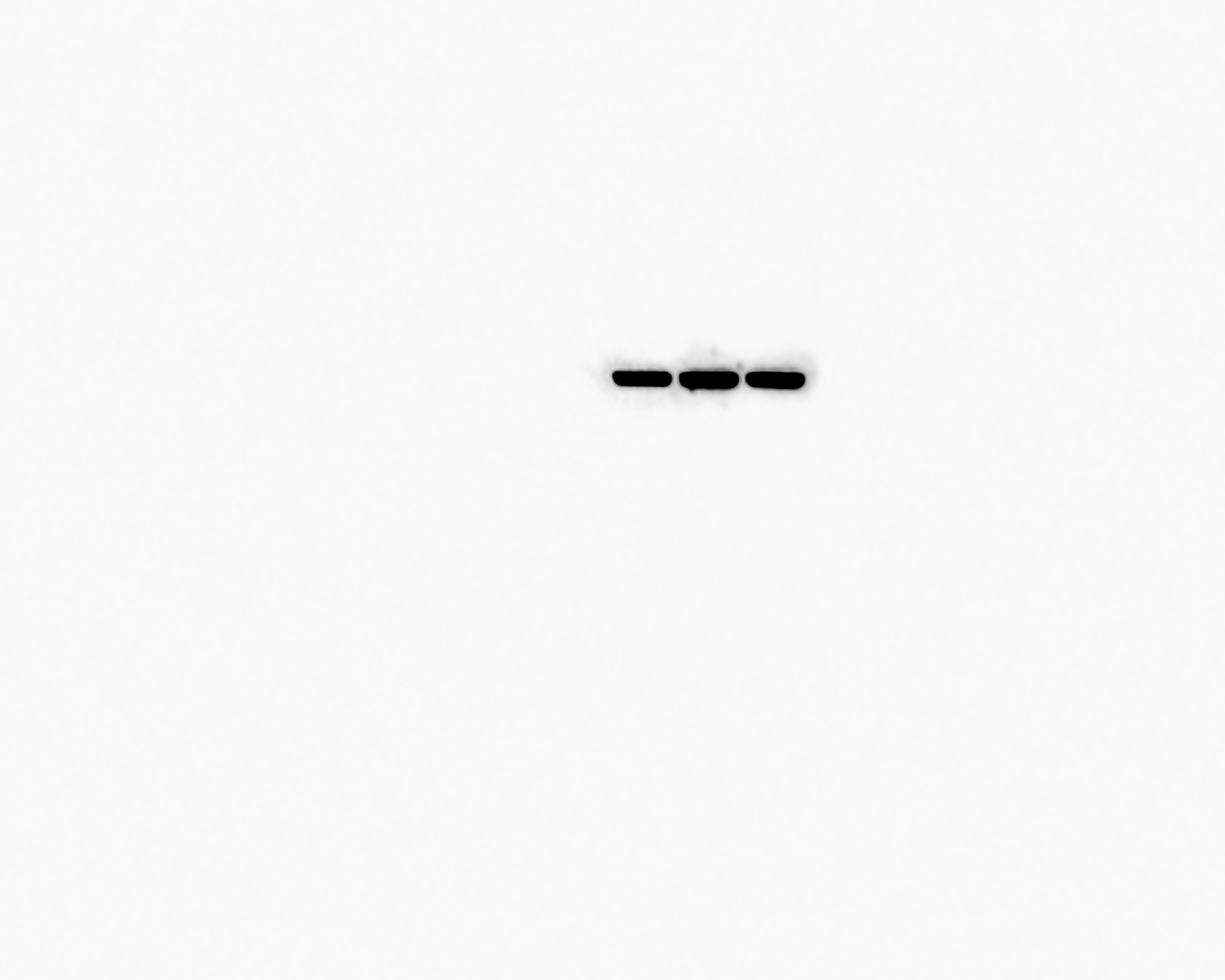

Supplement: Supplemental Information 1 [file peerj-13-19121-s001.zip › Figure 7/WB raw data/H1395/btnl9+gd 7_2(Chemiluminescence).tif]

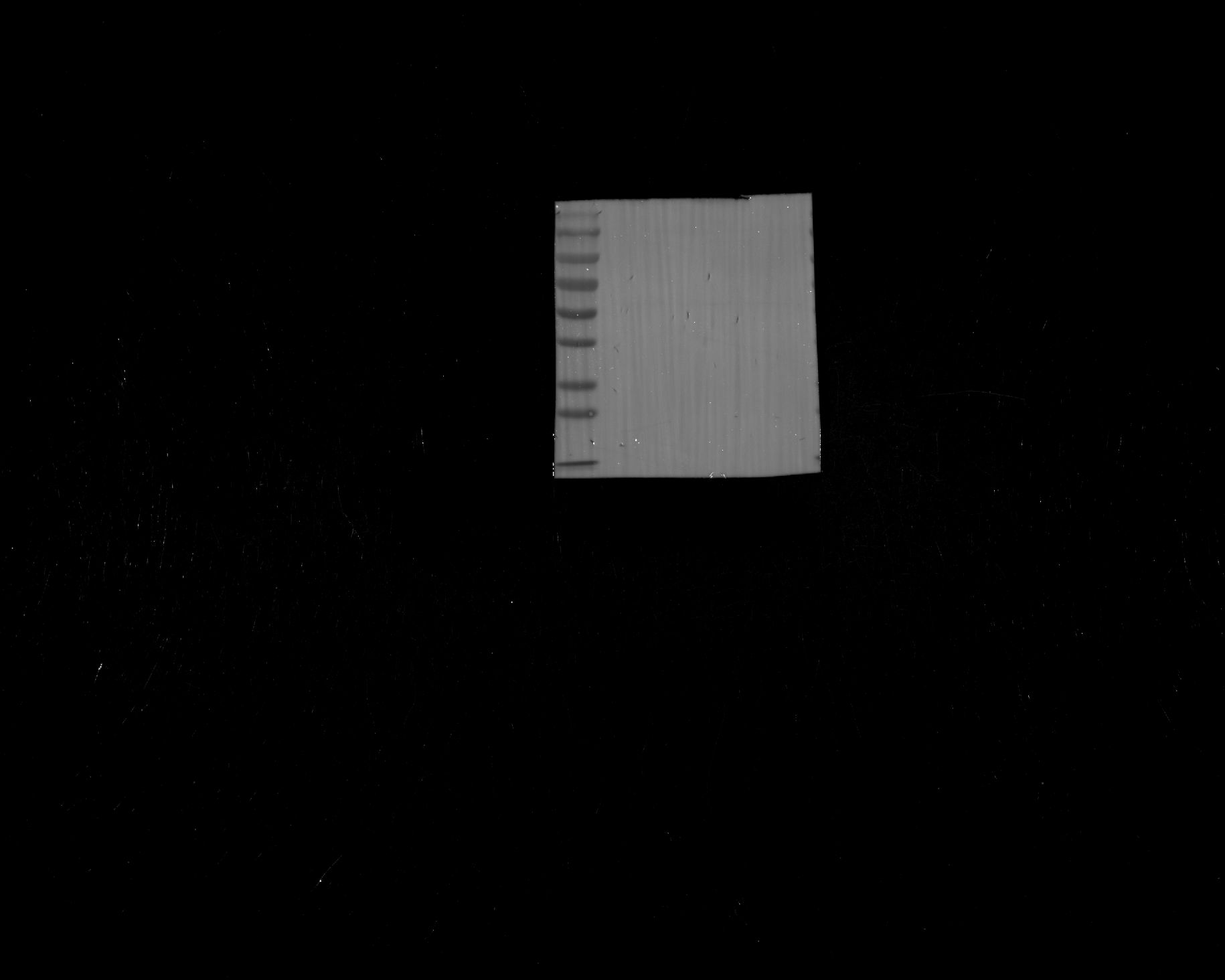

Supplement: Supplemental Information 1 [file peerj-13-19121-s001.zip › Figure 7/WB raw data/H1395/btnl9+gd 7_2(Colorimetric).tif]

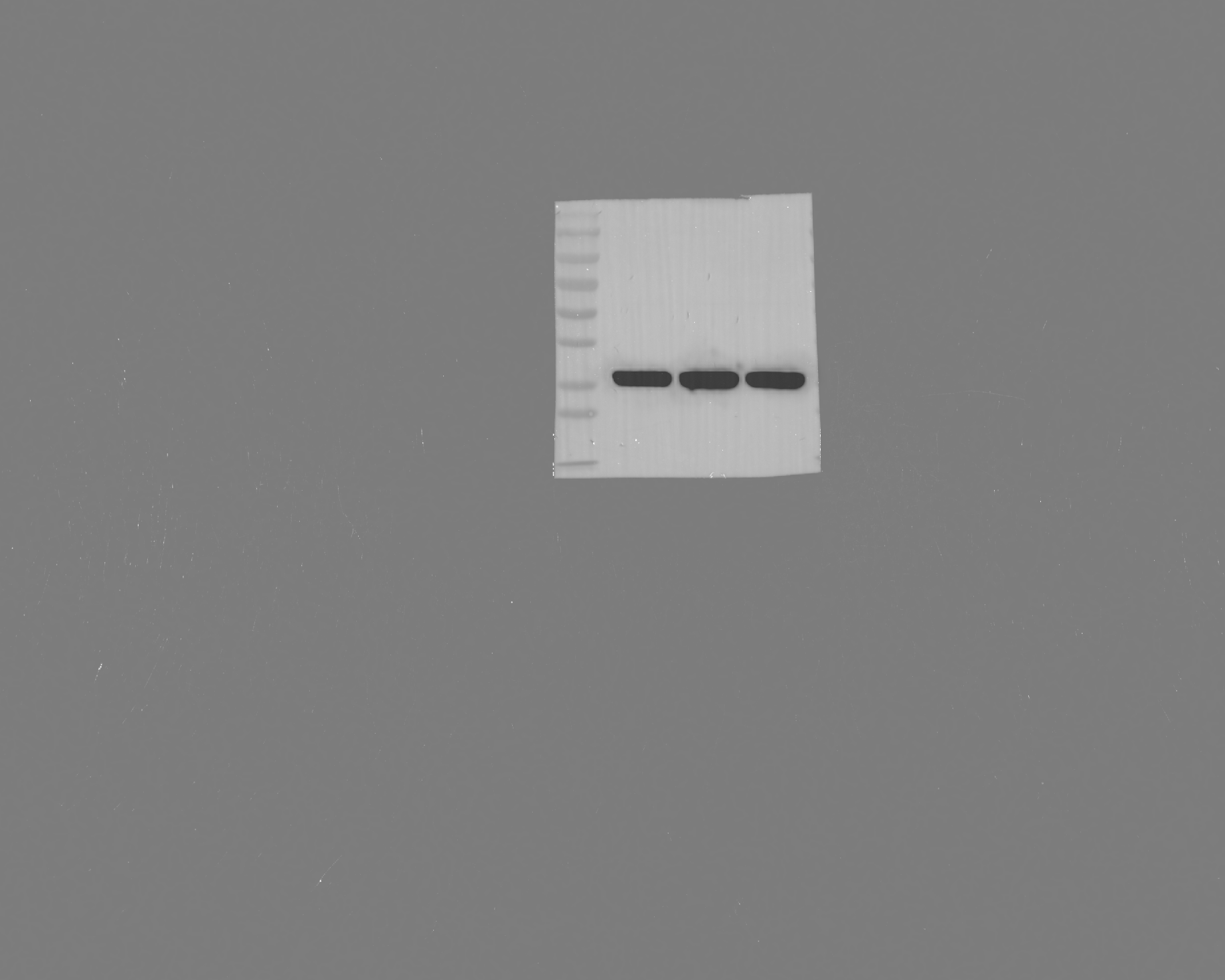

Supplement: Supplemental Information 1 [file peerj-13-19121-s001.zip › Figure 7/WB raw data/H1395/btnl9+gd 7_2(Composite).tif]

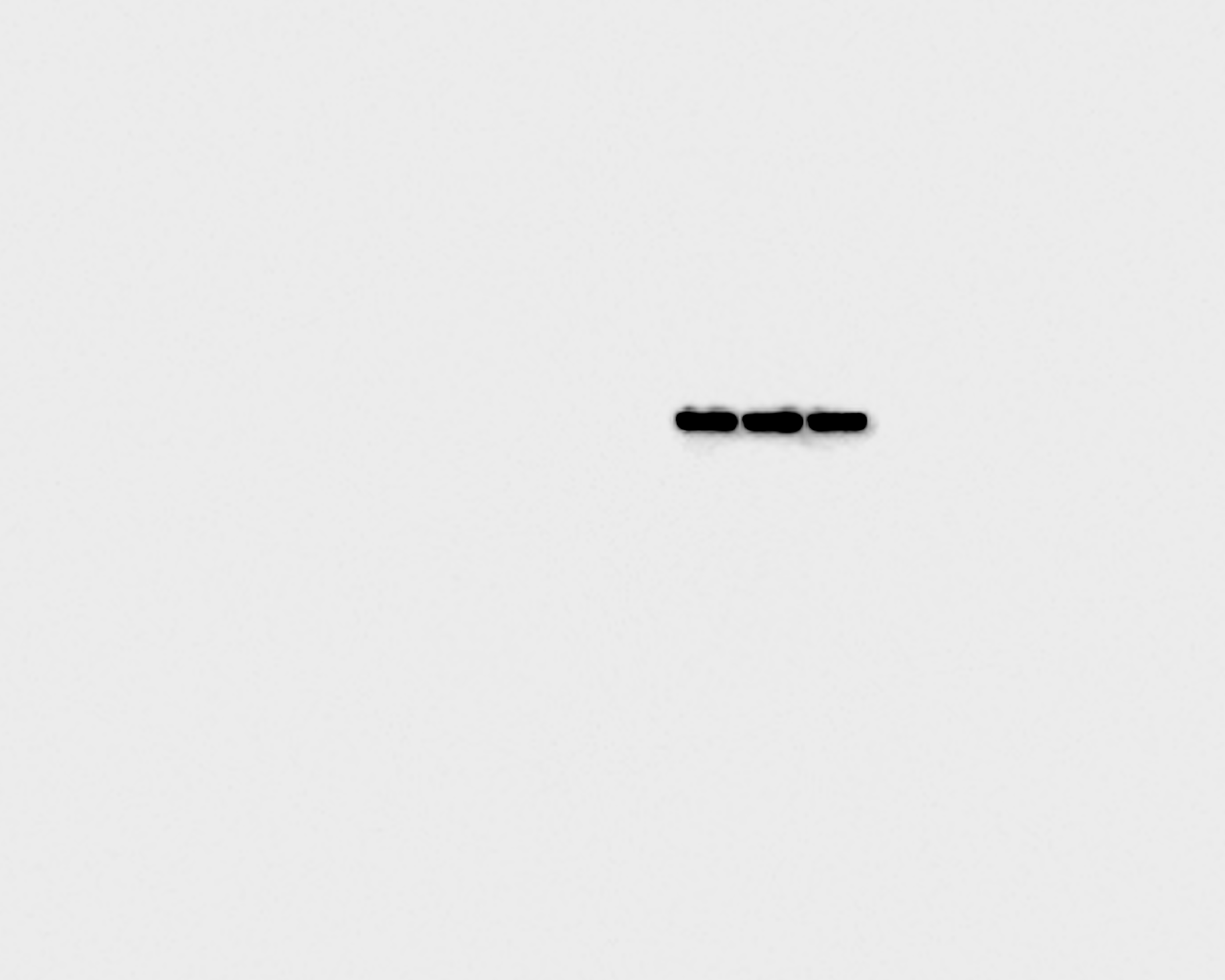

Supplement: Supplemental Information 1 [file peerj-13-19121-s001.zip › Figure 7/WB raw data/H1395/btnl9+gd 8_1(Chemiluminescence).tif]

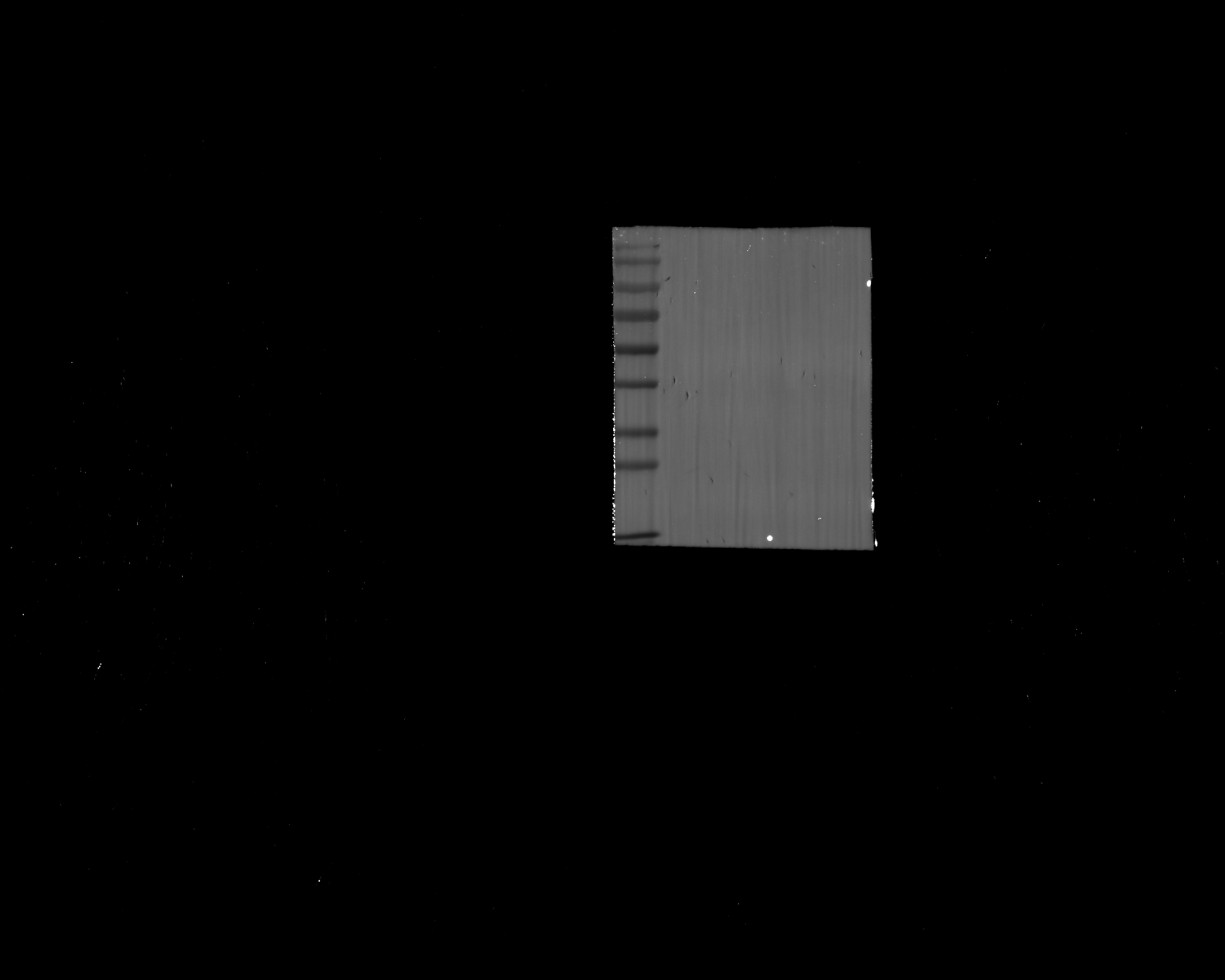

Supplement: Supplemental Information 1 [file peerj-13-19121-s001.zip › Figure 7/WB raw data/H1395/btnl9+gd 8_1(Colorimetric).tif]

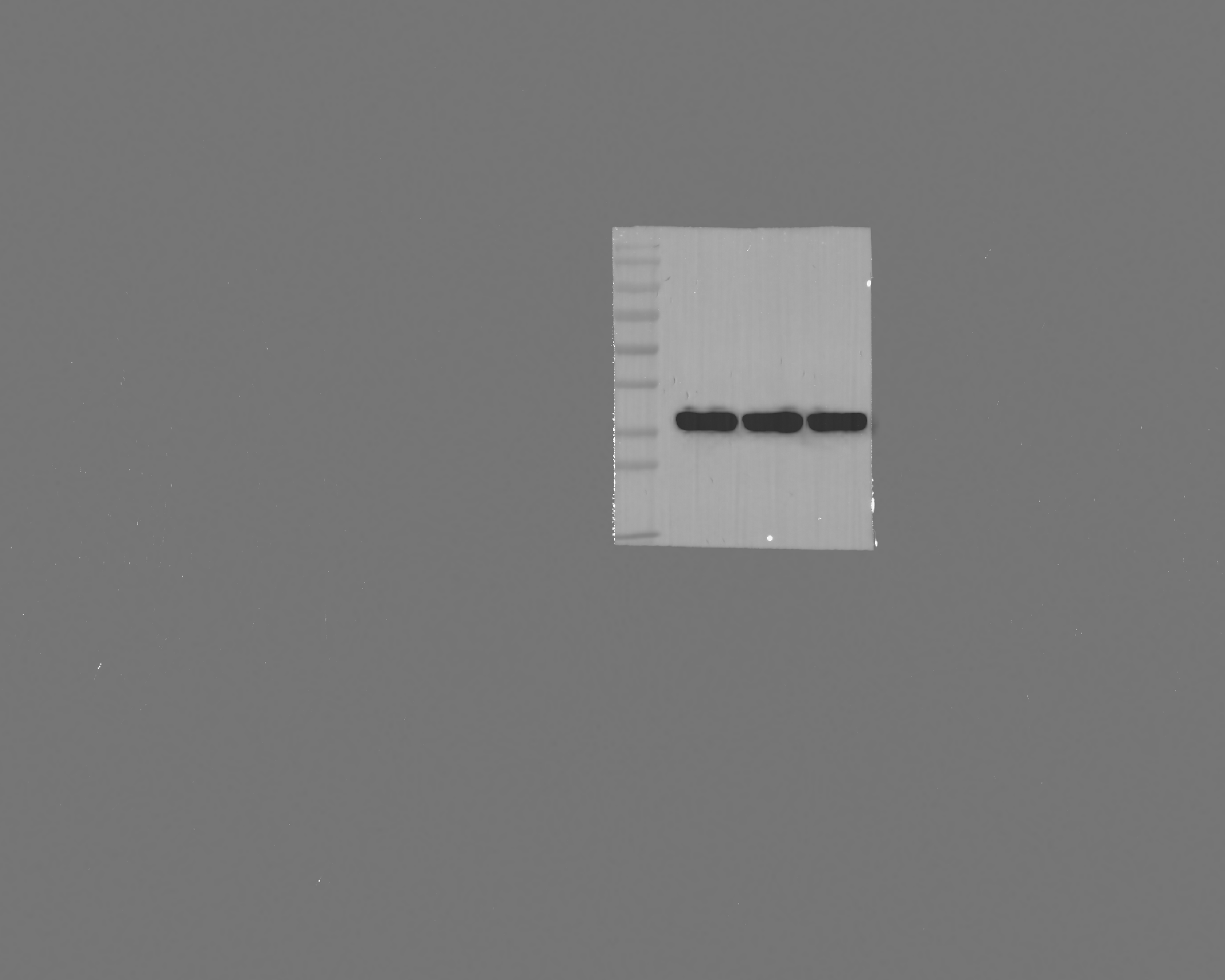

Supplement: Supplemental Information 1 [file peerj-13-19121-s001.zip › Figure 7/WB raw data/H1395/btnl9+gd 8_1(Composite).tif]

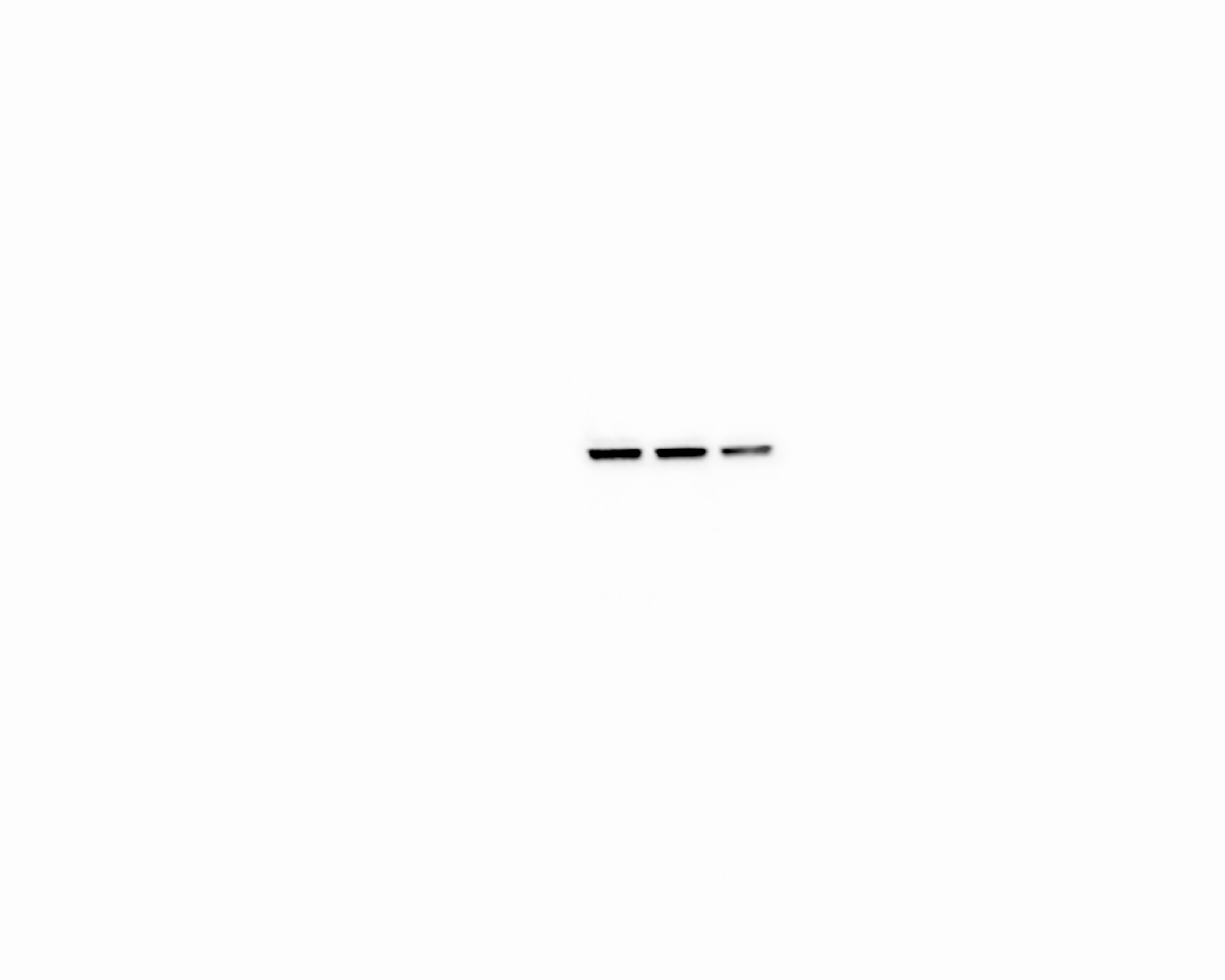

Supplement: Supplemental Information 1 [file peerj-13-19121-s001.zip › Figure 7/WB raw data/H1395/btnl9+gd 8_2(Chemiluminescence).tif]

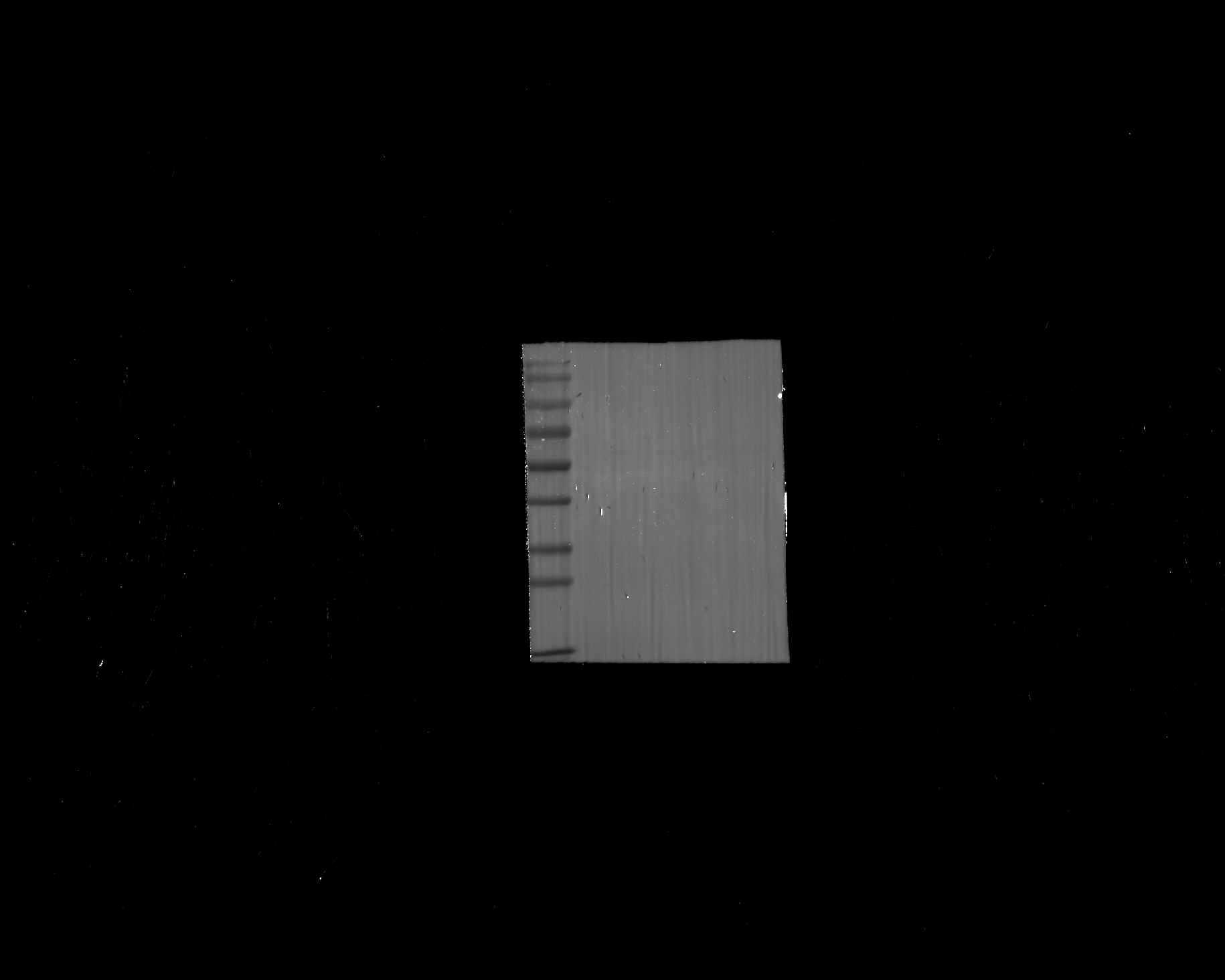

Supplement: Supplemental Information 1 [file peerj-13-19121-s001.zip › Figure 7/WB raw data/H1395/btnl9+gd 8_2(Colorimetric).tif]

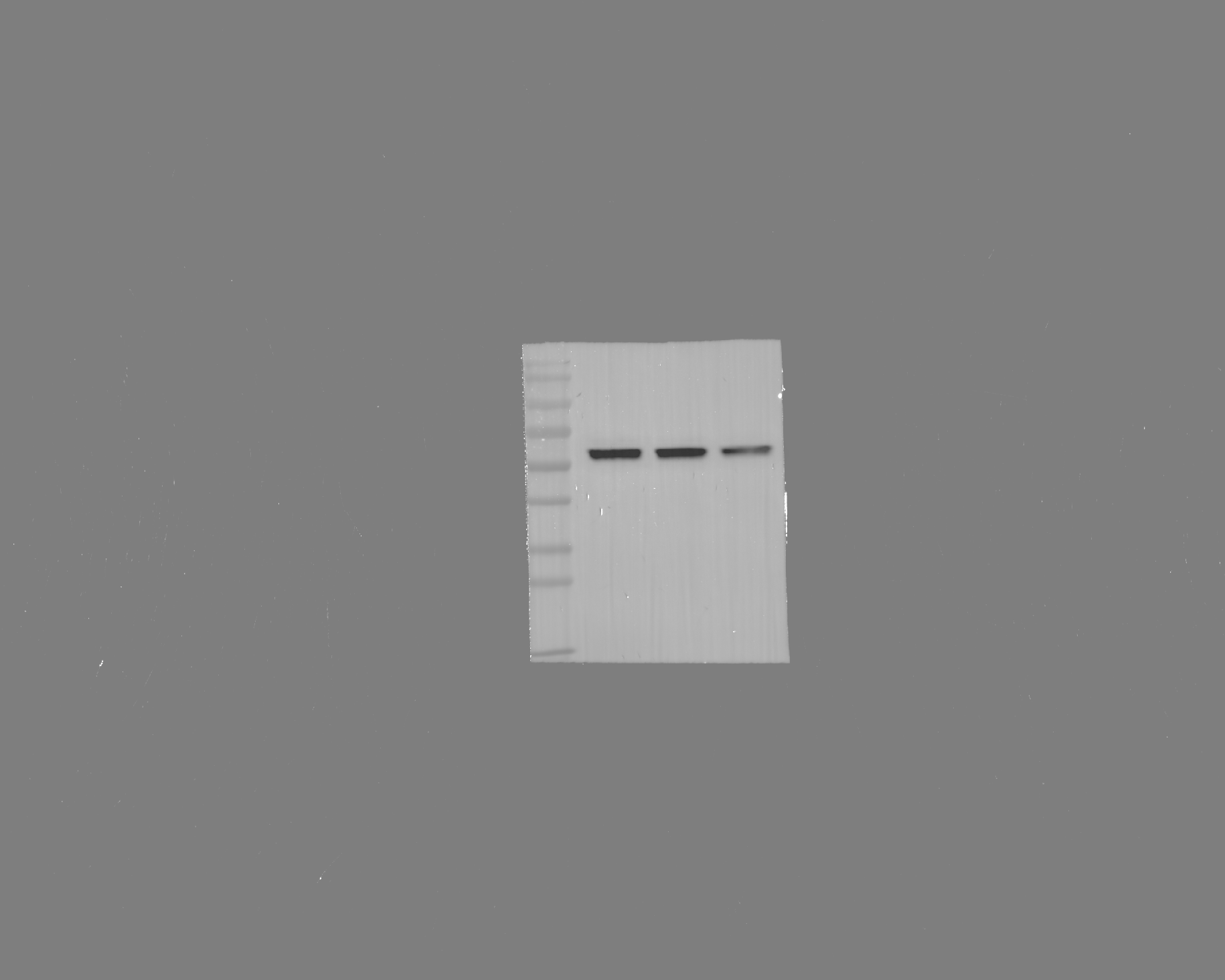

Supplement: Supplemental Information 1 [file peerj-13-19121-s001.zip › Figure 7/WB raw data/H1395/btnl9+gd 8_2(Composite).tif]

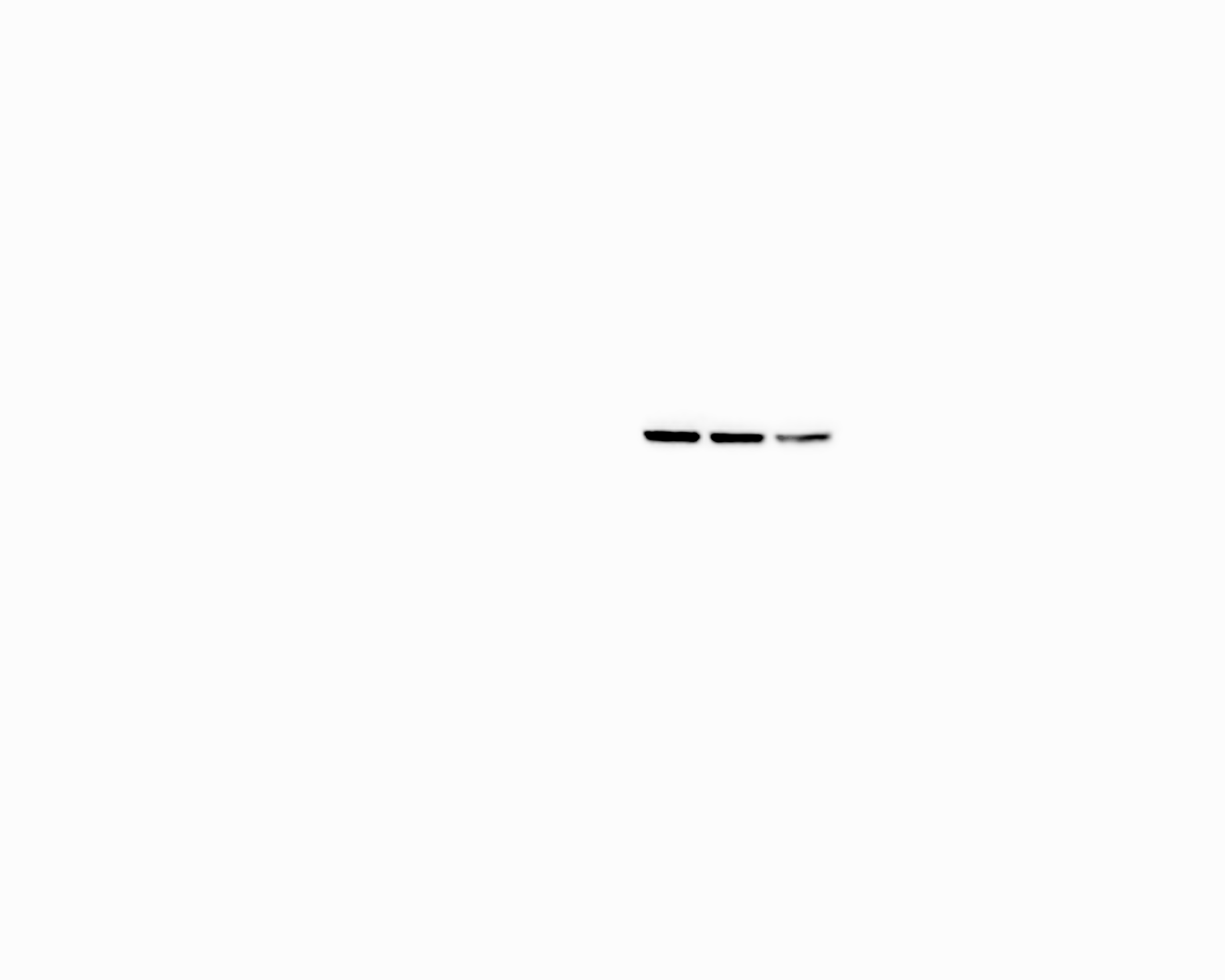

Supplement: Supplemental Information 1 [file peerj-13-19121-s001.zip › Figure 7/WB raw data/H1395/btnl9+gd 9_1(Chemiluminescence).tif]

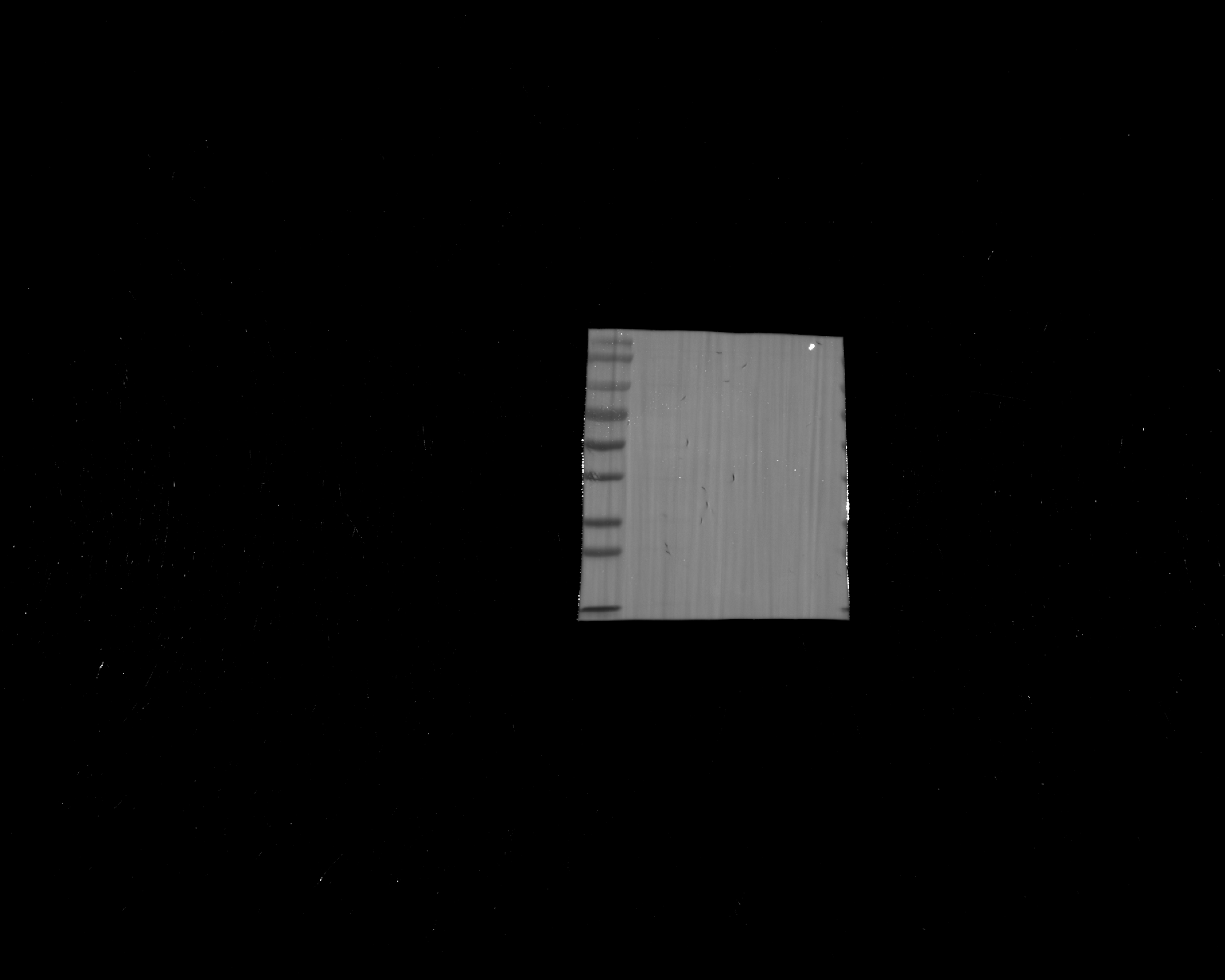

Supplement: Supplemental Information 1 [file peerj-13-19121-s001.zip › Figure 7/WB raw data/H1395/btnl9+gd 9_1(Colorimetric).tif]

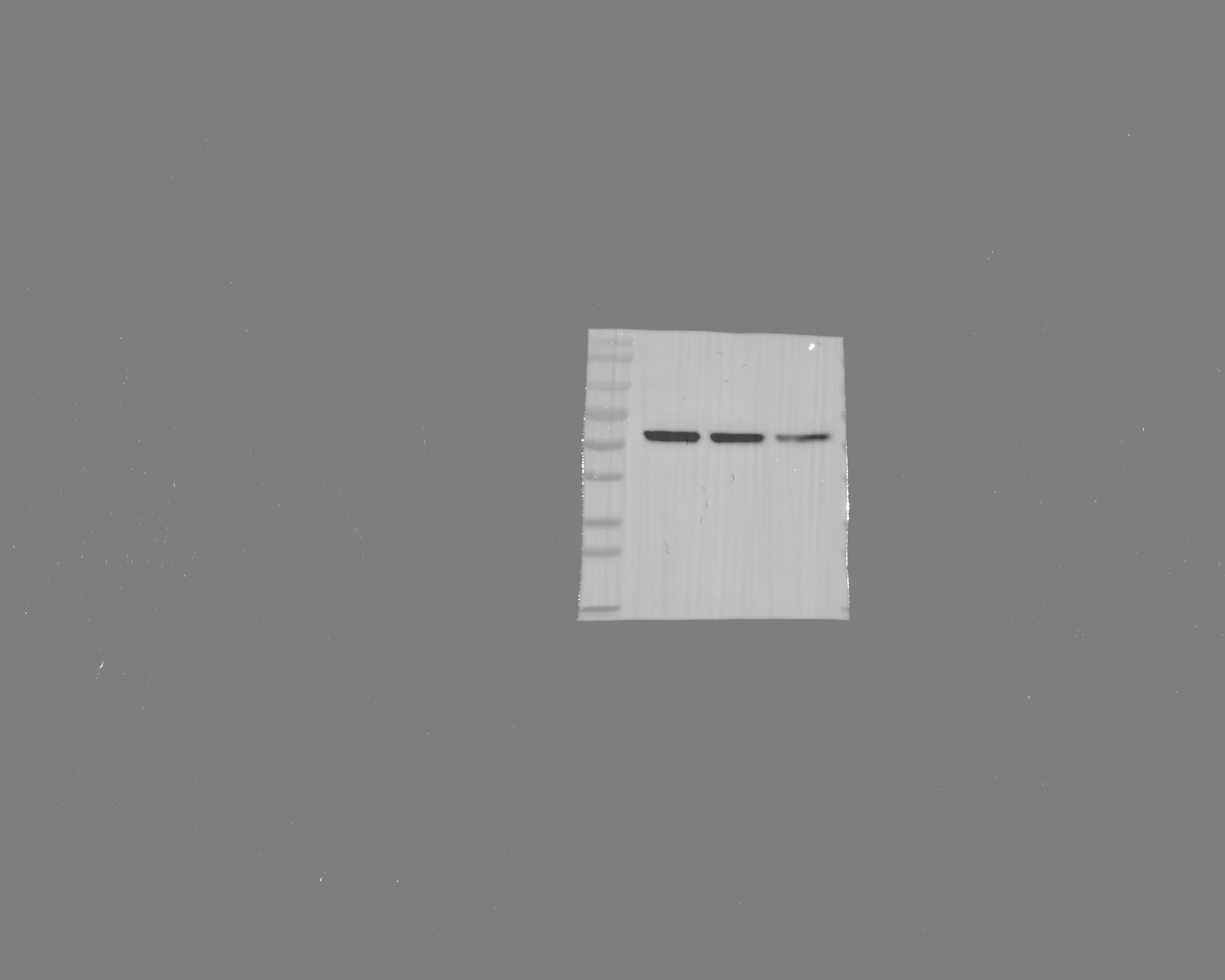

Supplement: Supplemental Information 1 [file peerj-13-19121-s001.zip › Figure 7/WB raw data/H1395/btnl9+gd 9_1(Composite).tif]

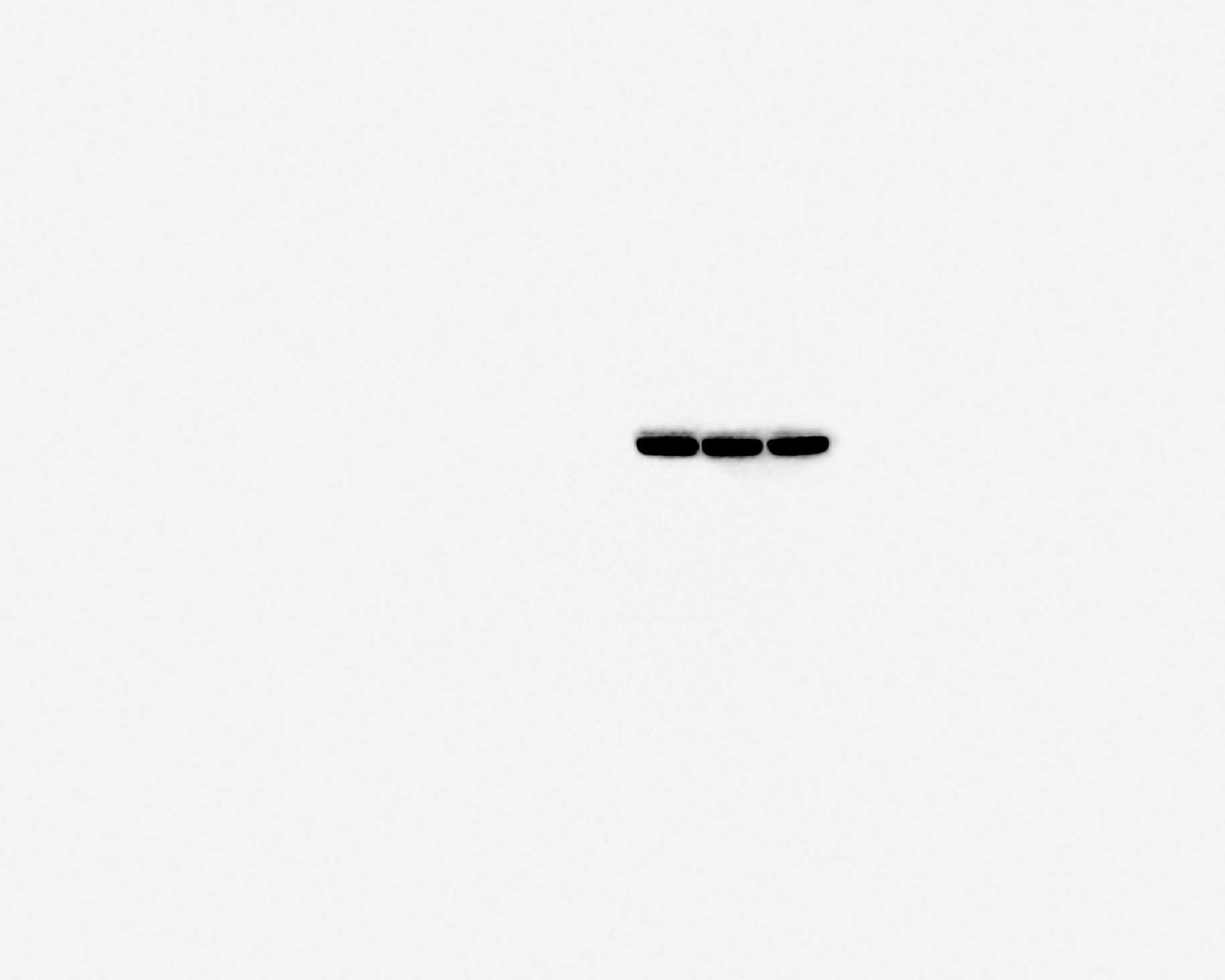

Supplement: Supplemental Information 1 [file peerj-13-19121-s001.zip › Figure 7/WB raw data/H1395/btnl9+gd 9_2(Chemiluminescence).tif]

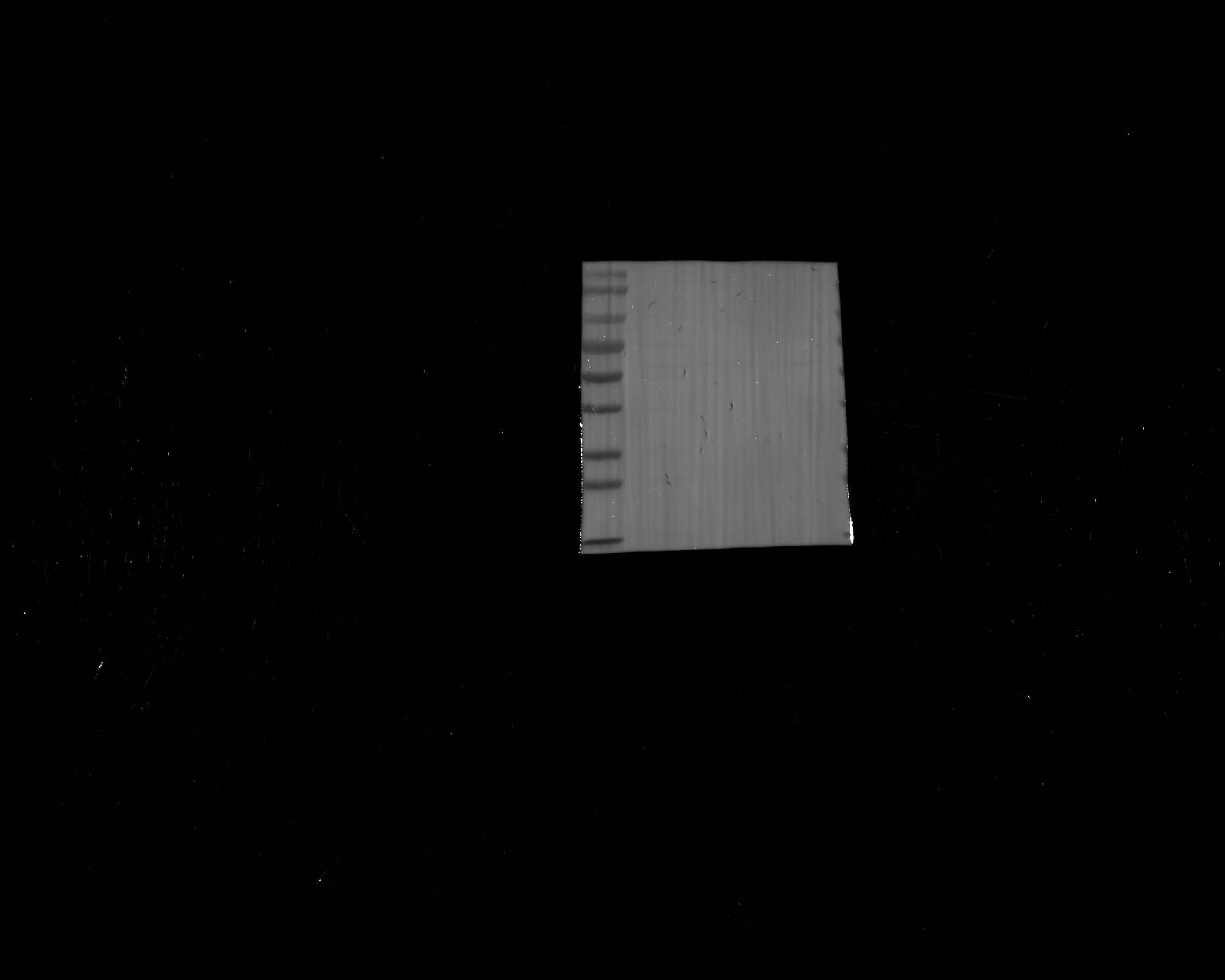

Supplement: Supplemental Information 1 [file peerj-13-19121-s001.zip › Figure 7/WB raw data/H1395/btnl9+gd 9_2(Colorimetric).tif]

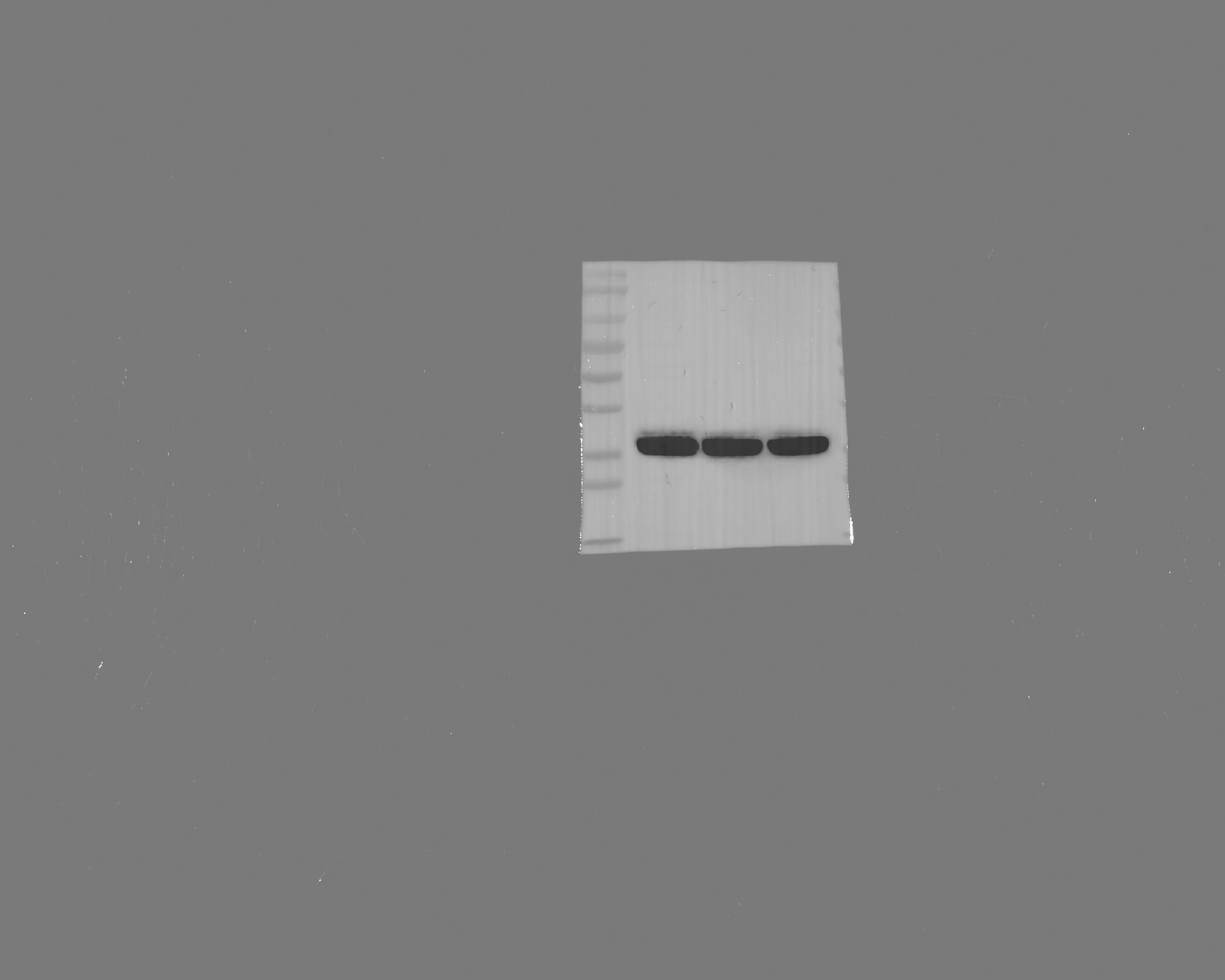

Supplement: Supplemental Information 1 [file peerj-13-19121-s001.zip › Figure 7/WB raw data/H1395/btnl9+gd 9_2(Composite).tif]

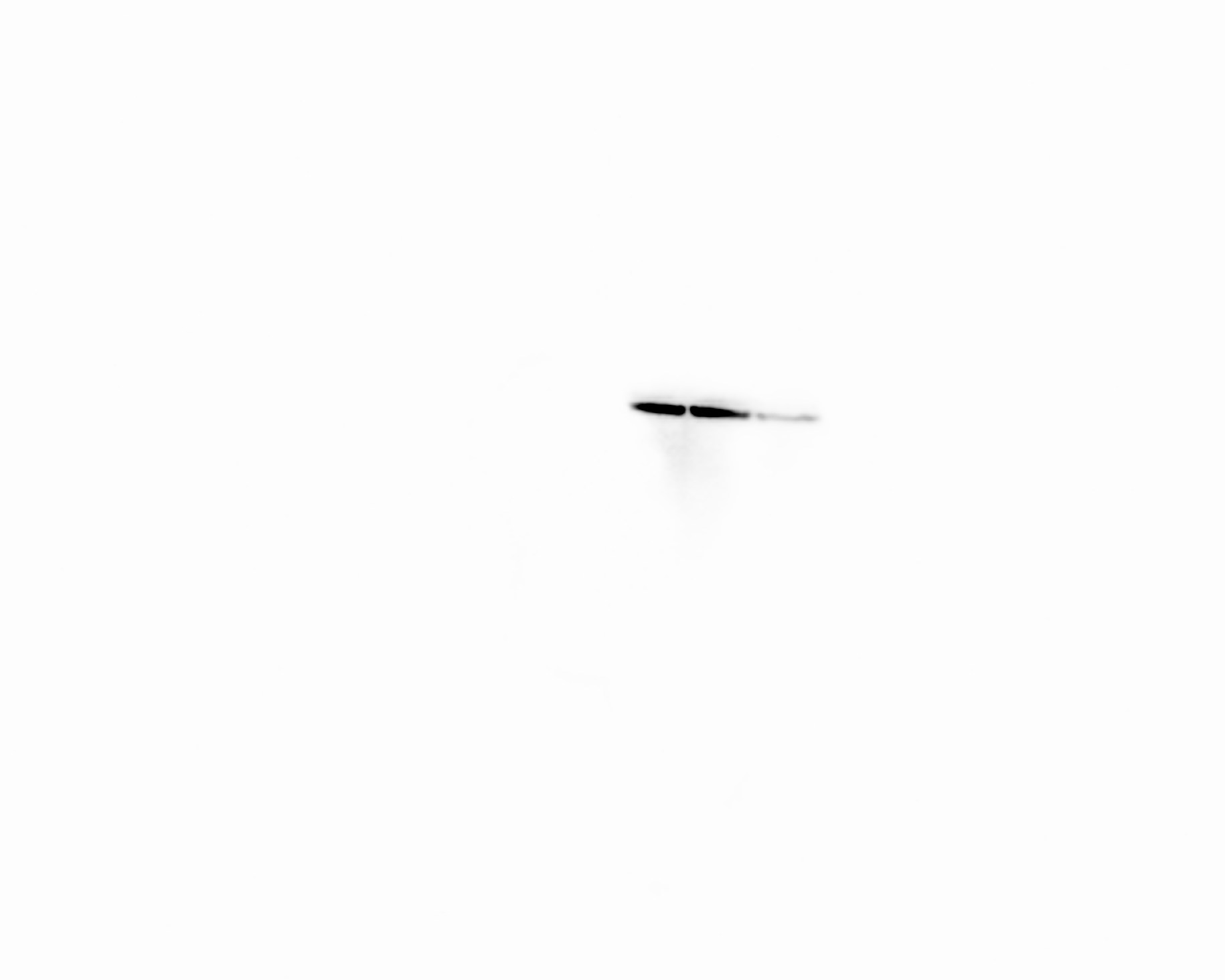

Supplement: Supplemental Information 1 [file peerj-13-19121-s001.zip › Figure 7/WB raw data/PC9/btnl9+gd 4_1(Chemiluminescence).tif]

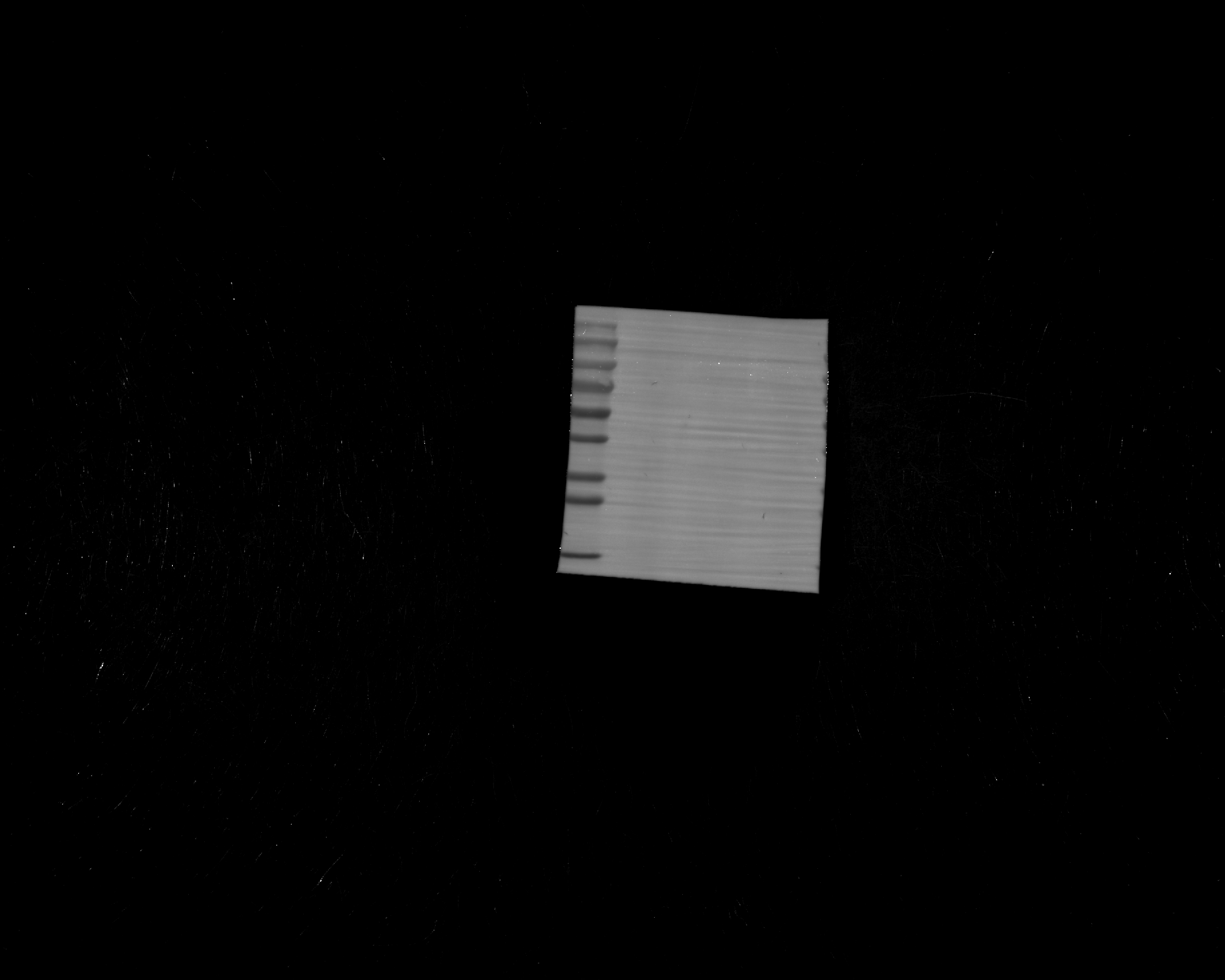

Supplement: Supplemental Information 1 [file peerj-13-19121-s001.zip › Figure 7/WB raw data/PC9/btnl9+gd 4_1(Colorimetric).tif]

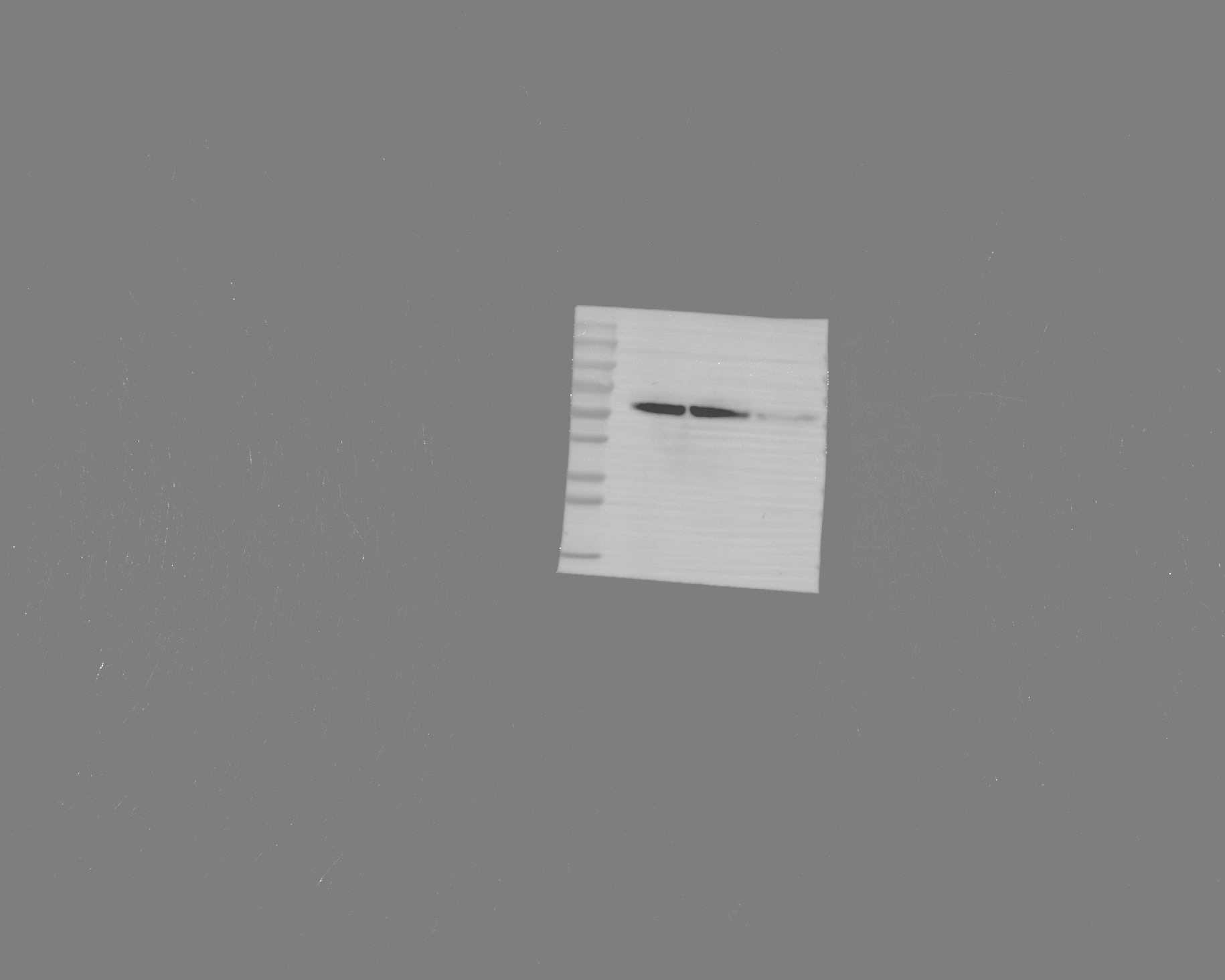

Supplement: Supplemental Information 1 [file peerj-13-19121-s001.zip › Figure 7/WB raw data/PC9/btnl9+gd 4_1(Composite).tif]

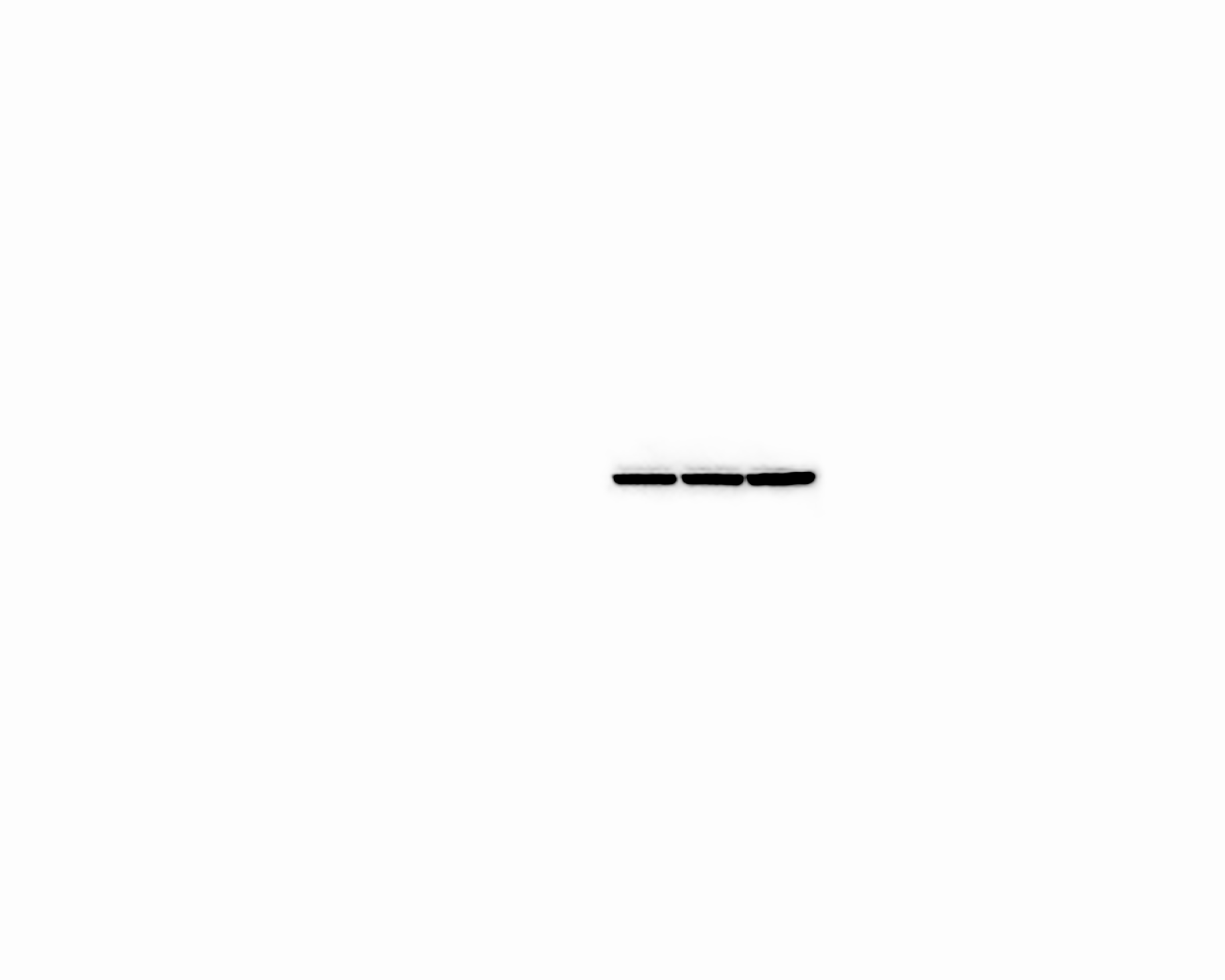

Supplement: Supplemental Information 1 [file peerj-13-19121-s001.zip › Figure 7/WB raw data/PC9/btnl9+gd 4_2(Chemiluminescence).tif]

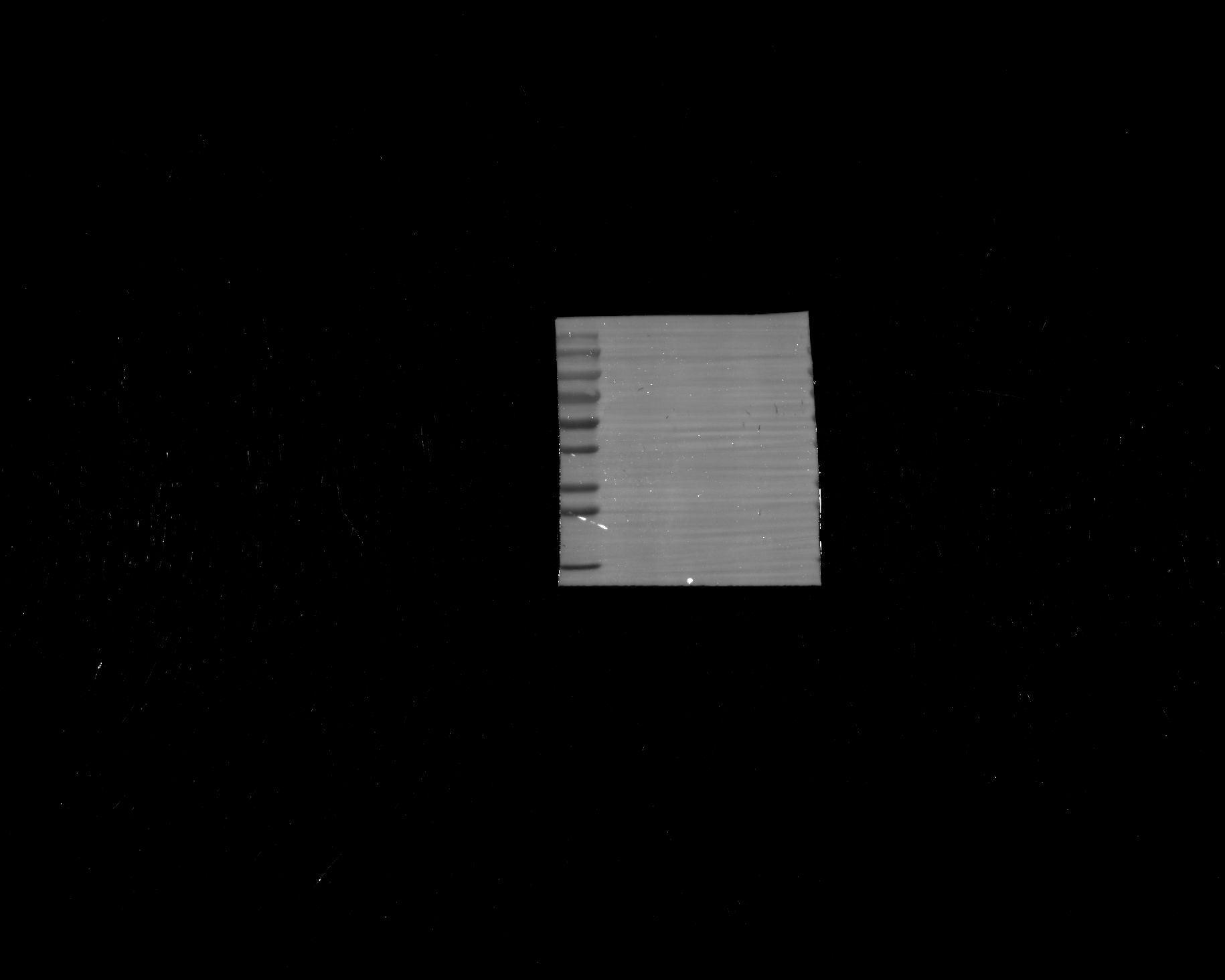

Supplement: Supplemental Information 1 [file peerj-13-19121-s001.zip › Figure 7/WB raw data/PC9/btnl9+gd 4_2(Colorimetric).tif]

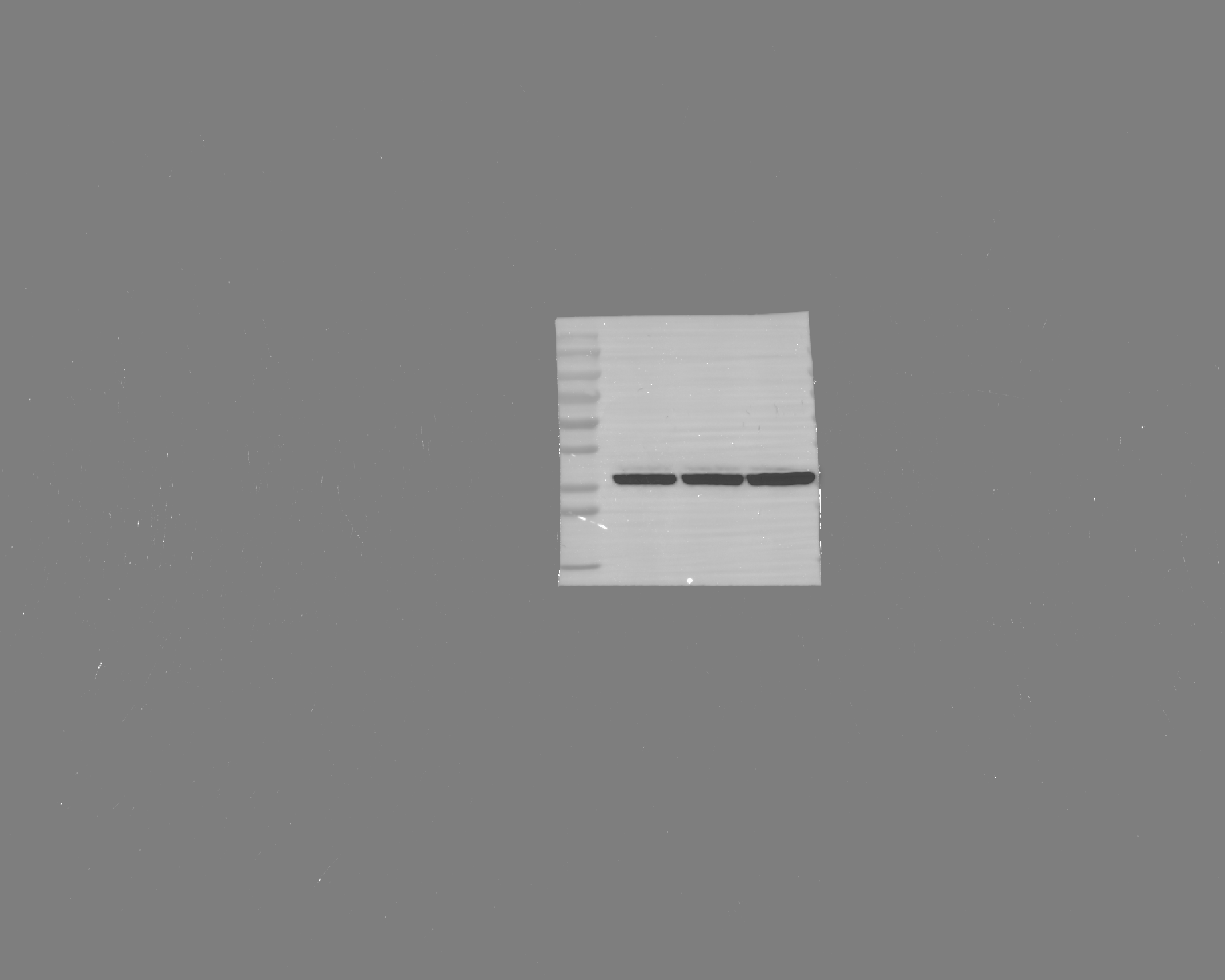

Supplement: Supplemental Information 1 [file peerj-13-19121-s001.zip › Figure 7/WB raw data/PC9/btnl9+gd 4_2(Composite).tif]

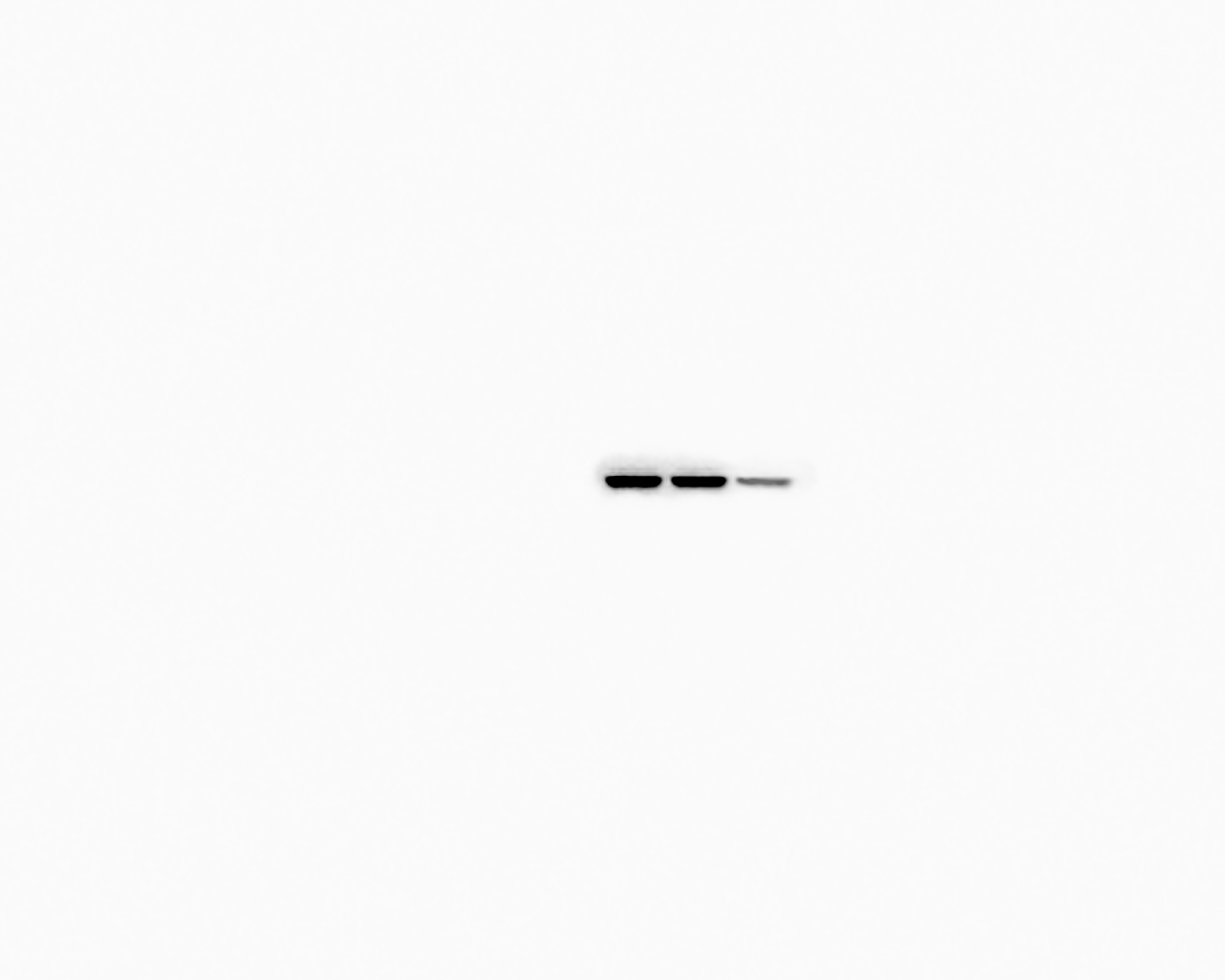

Supplement: Supplemental Information 1 [file peerj-13-19121-s001.zip › Figure 7/WB raw data/PC9/btnl9+gd 5_1(Chemiluminescence).tif]

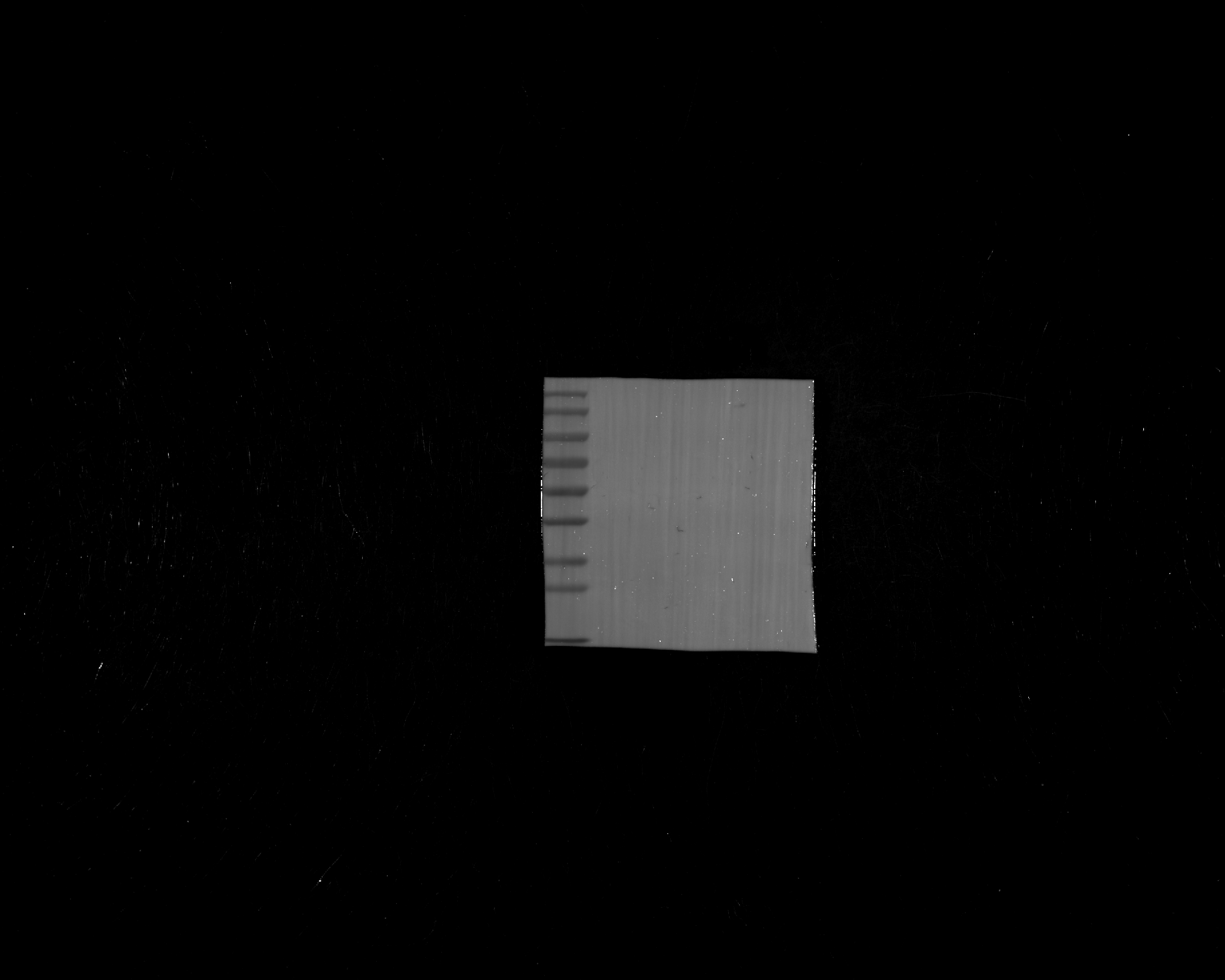

Supplement: Supplemental Information 1 [file peerj-13-19121-s001.zip › Figure 7/WB raw data/PC9/btnl9+gd 5_1(Colorimetric).tif]

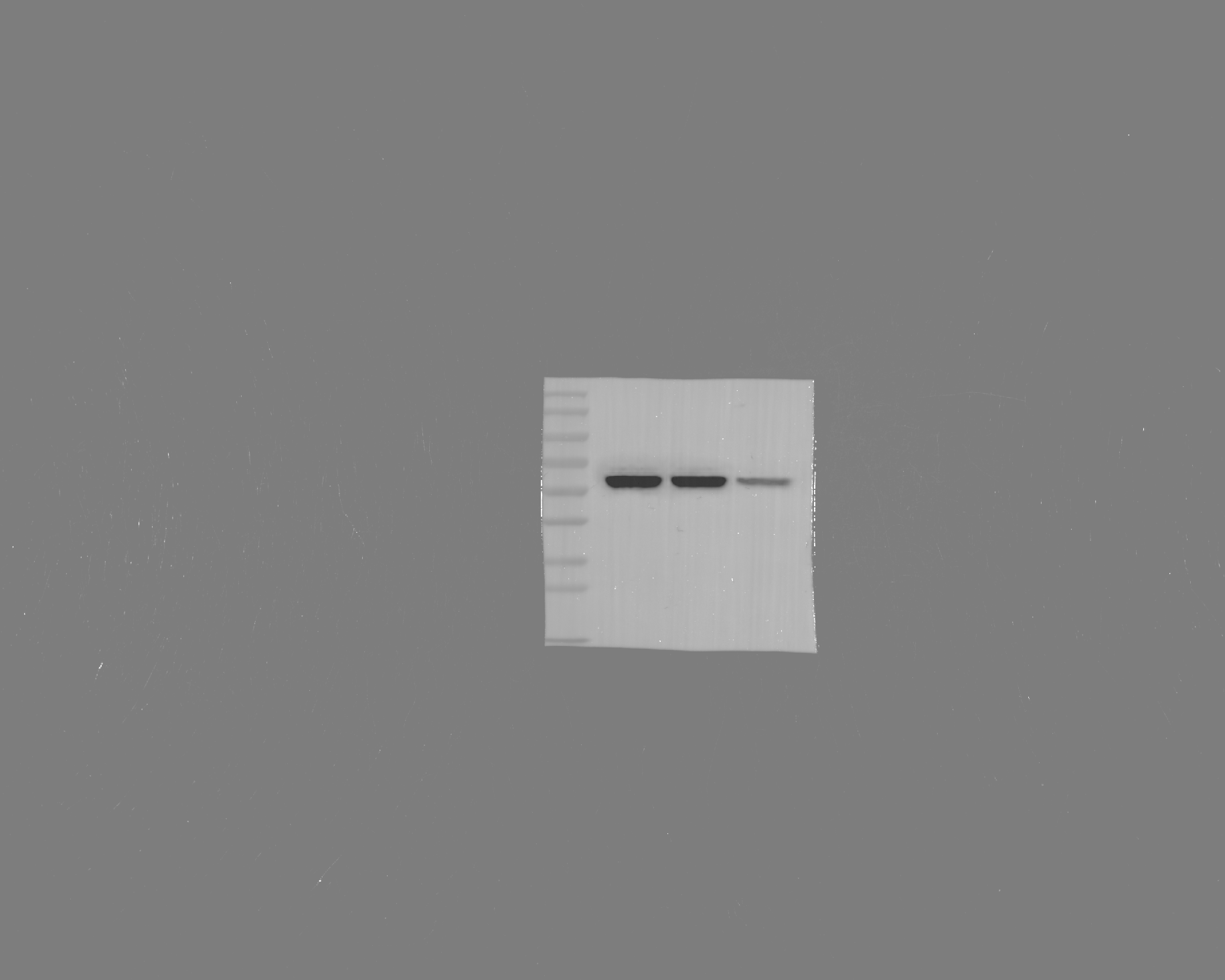

Supplement: Supplemental Information 1 [file peerj-13-19121-s001.zip › Figure 7/WB raw data/PC9/btnl9+gd 5_1(Composite).tif]

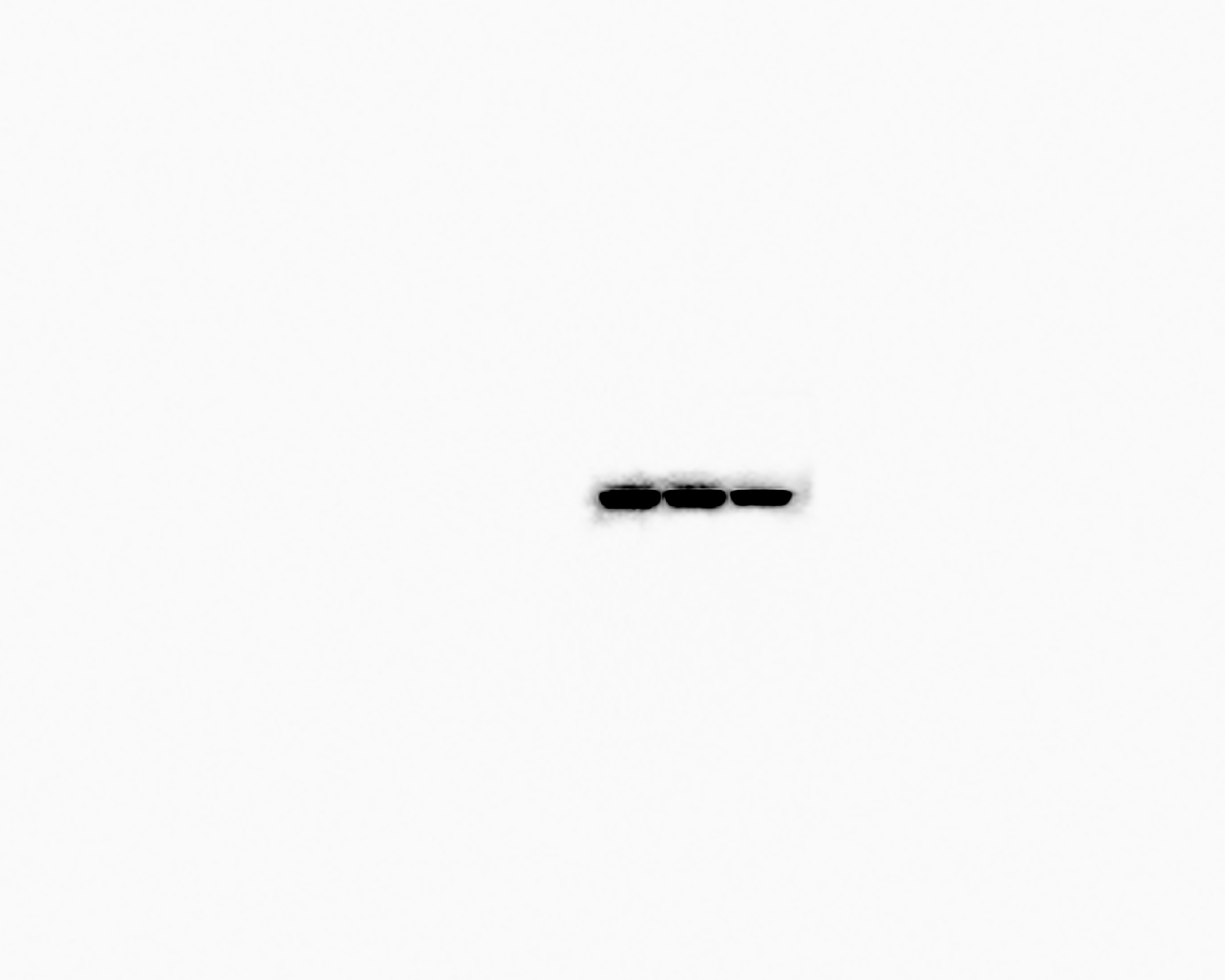

Supplement: Supplemental Information 1 [file peerj-13-19121-s001.zip › Figure 7/WB raw data/PC9/btnl9+gd 5_2(Chemiluminescence).tif]

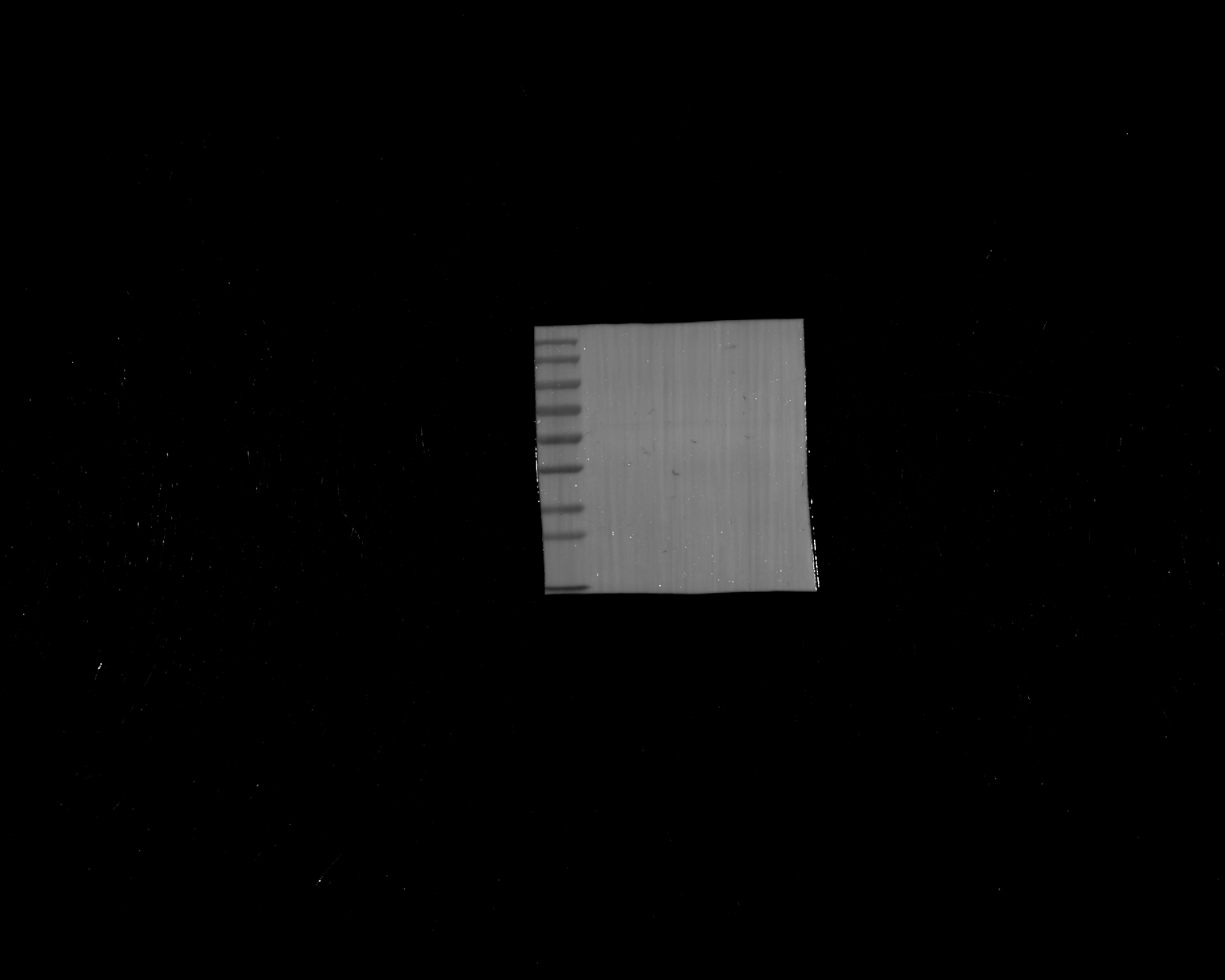

Supplement: Supplemental Information 1 [file peerj-13-19121-s001.zip › Figure 7/WB raw data/PC9/btnl9+gd 5_2(Colorimetric).tif]

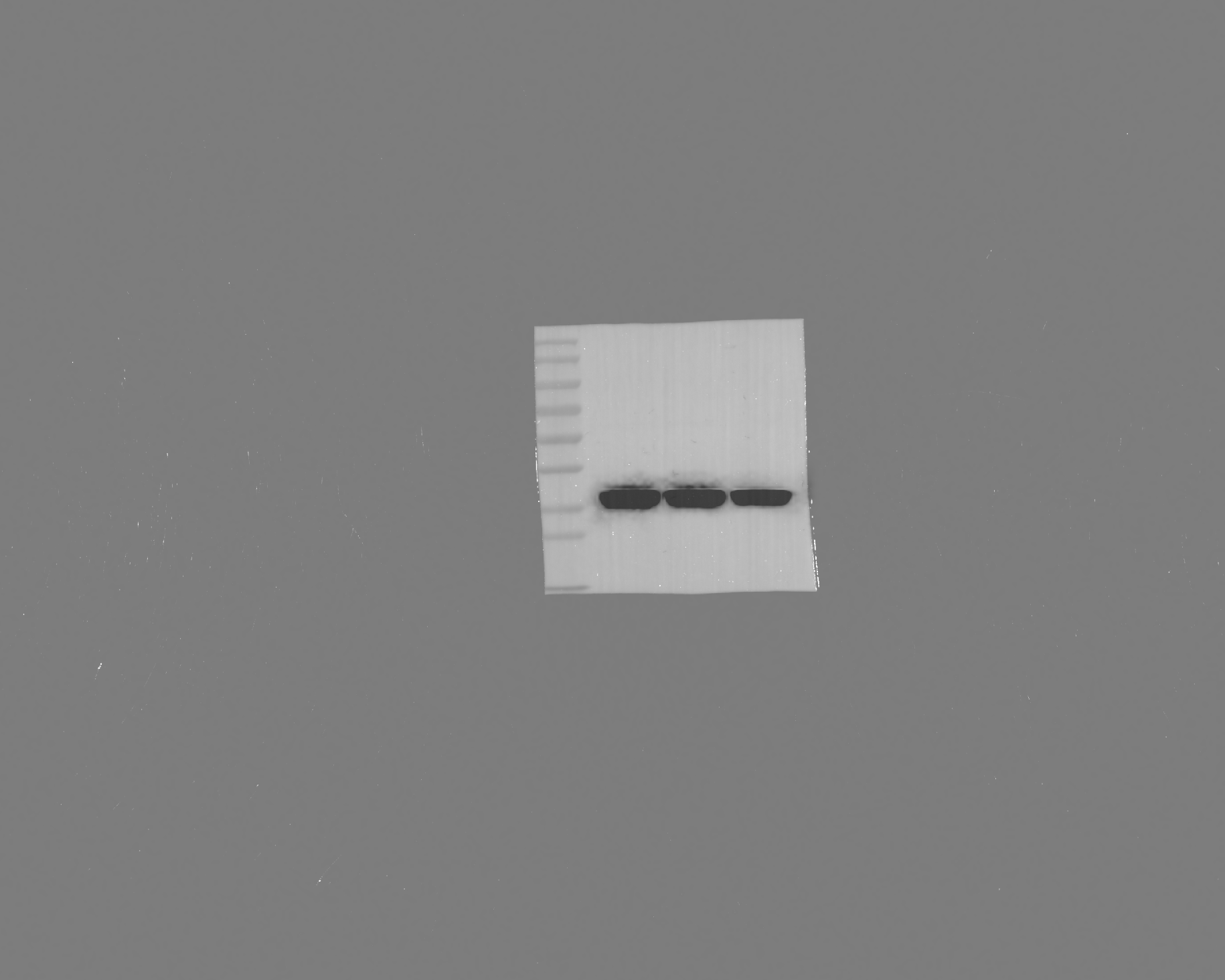

Supplement: Supplemental Information 1 [file peerj-13-19121-s001.zip › Figure 7/WB raw data/PC9/btnl9+gd 5_2(Composite).tif]

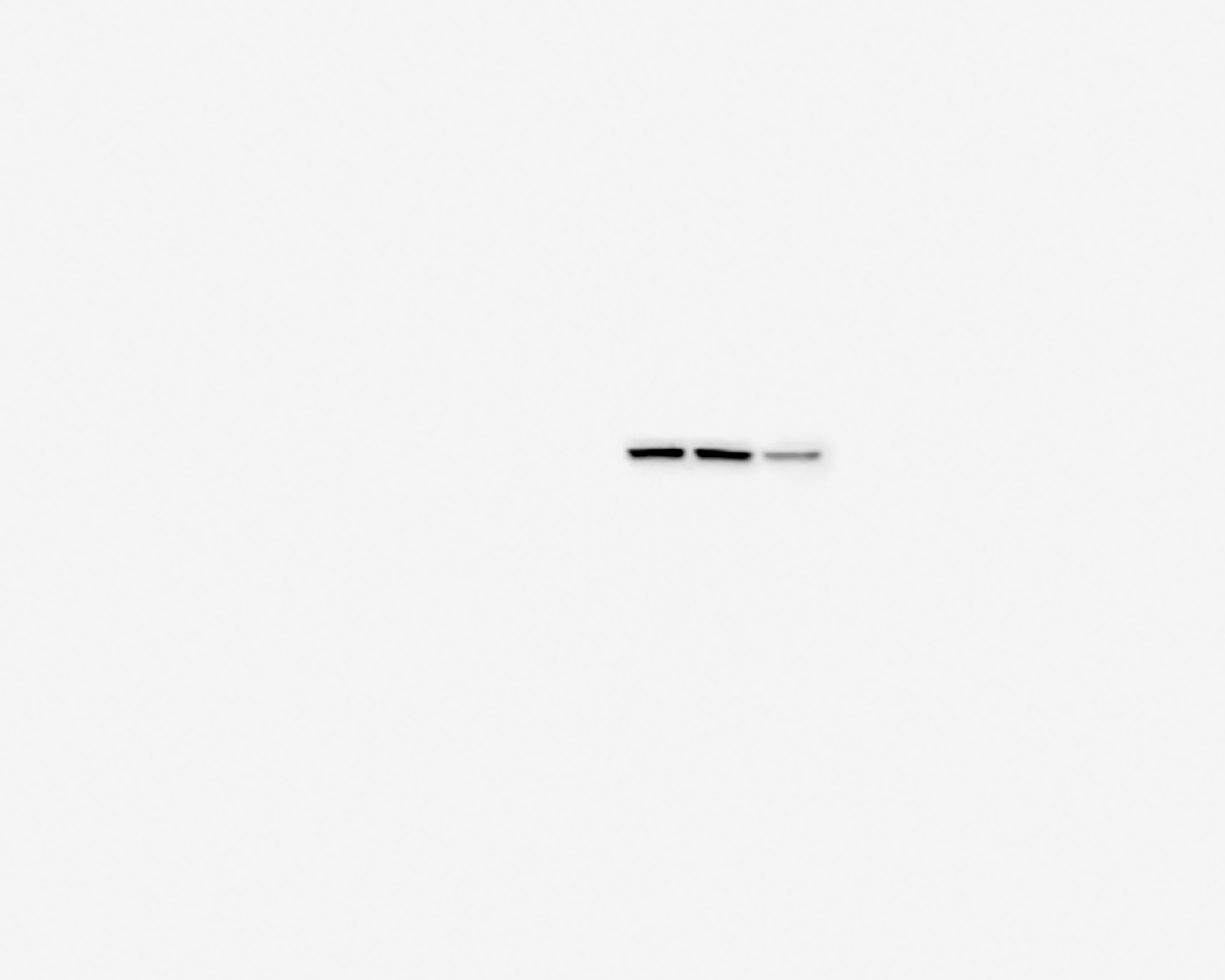

Supplement: Supplemental Information 1 [file peerj-13-19121-s001.zip › Figure 7/WB raw data/PC9/btnl9+gd 6_1(Chemiluminescence).tif]

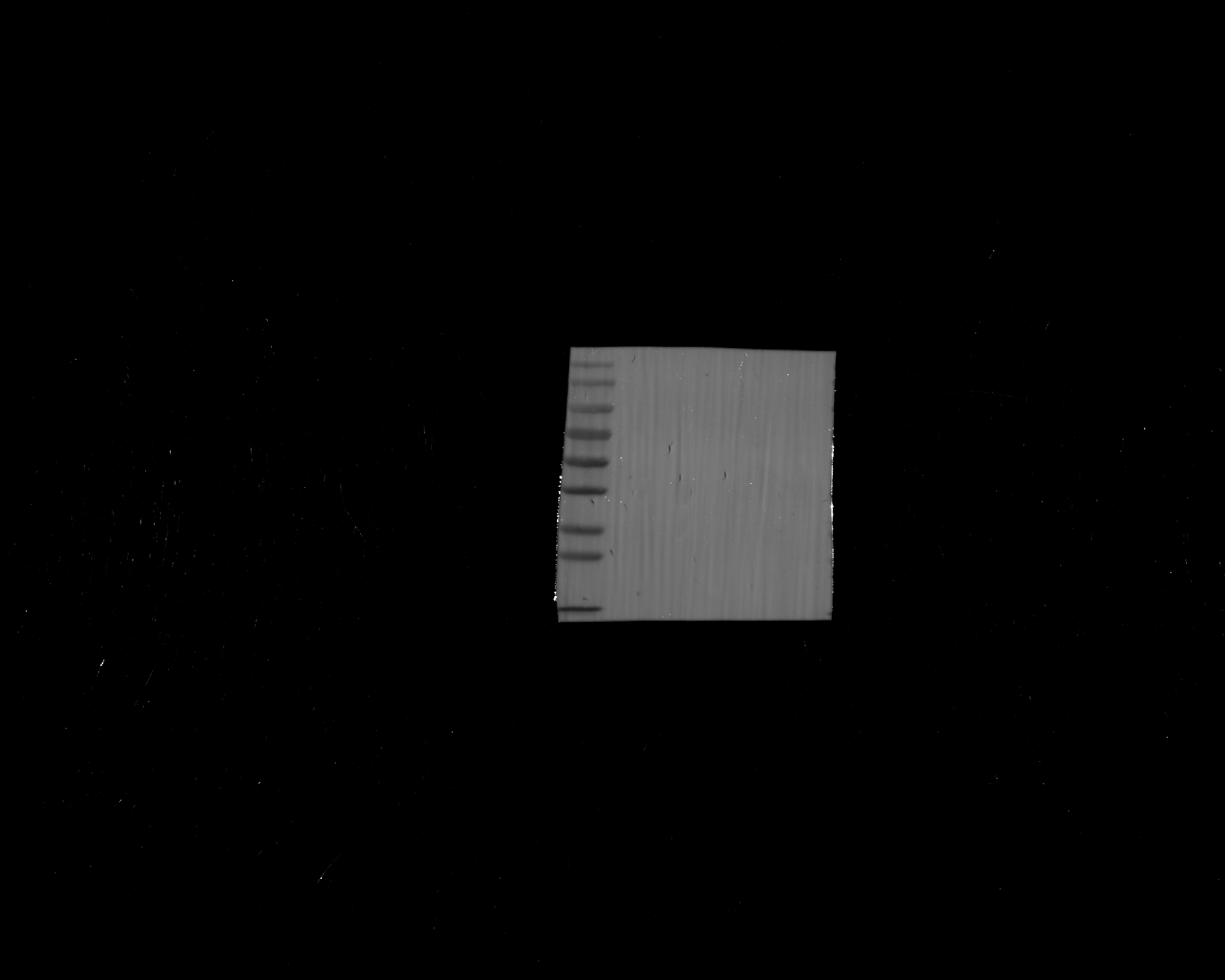

Supplement: Supplemental Information 1 [file peerj-13-19121-s001.zip › Figure 7/WB raw data/PC9/btnl9+gd 6_1(Colorimetric).tif]

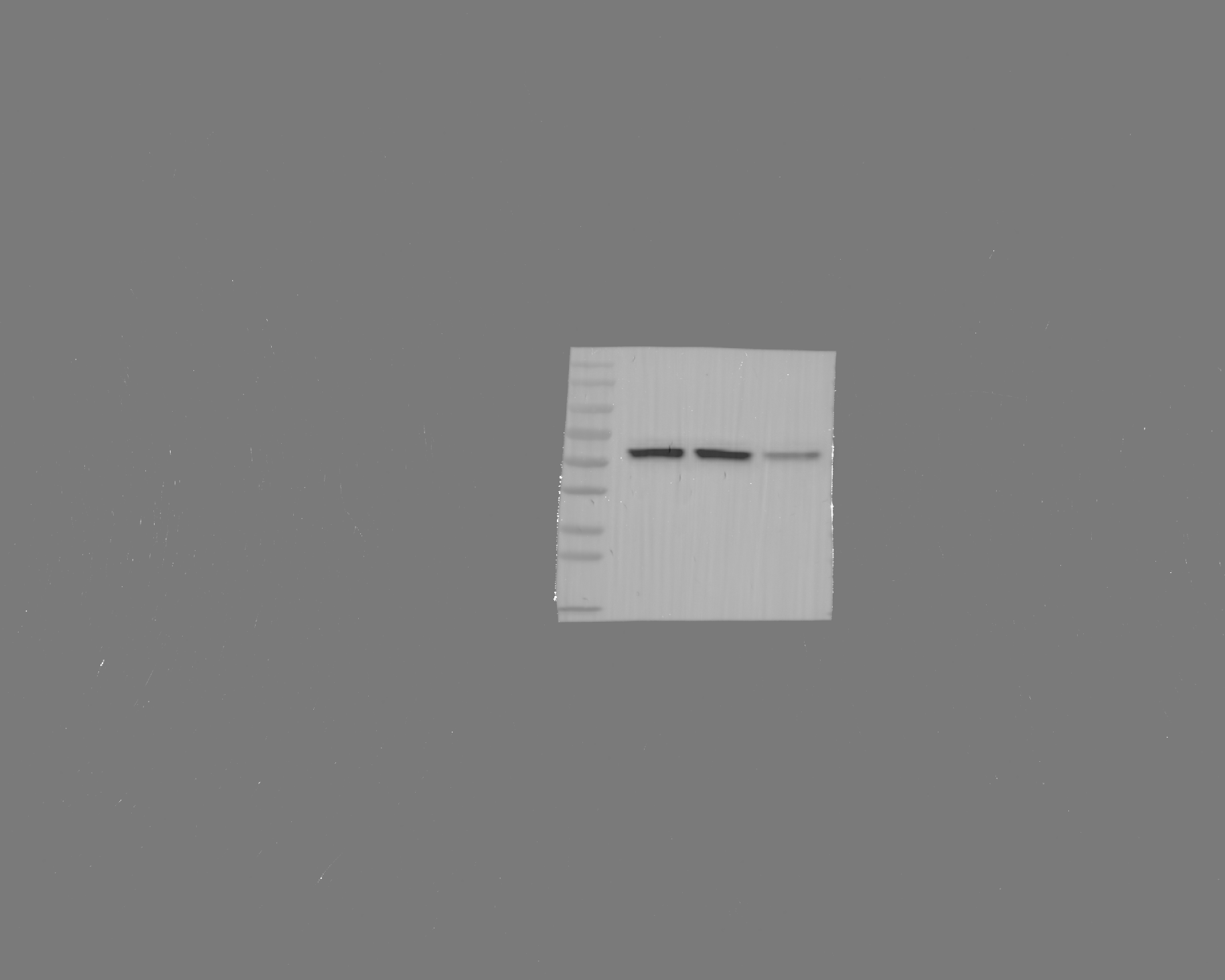

Supplement: Supplemental Information 1 [file peerj-13-19121-s001.zip › Figure 7/WB raw data/PC9/btnl9+gd 6_1(Composite).tif]

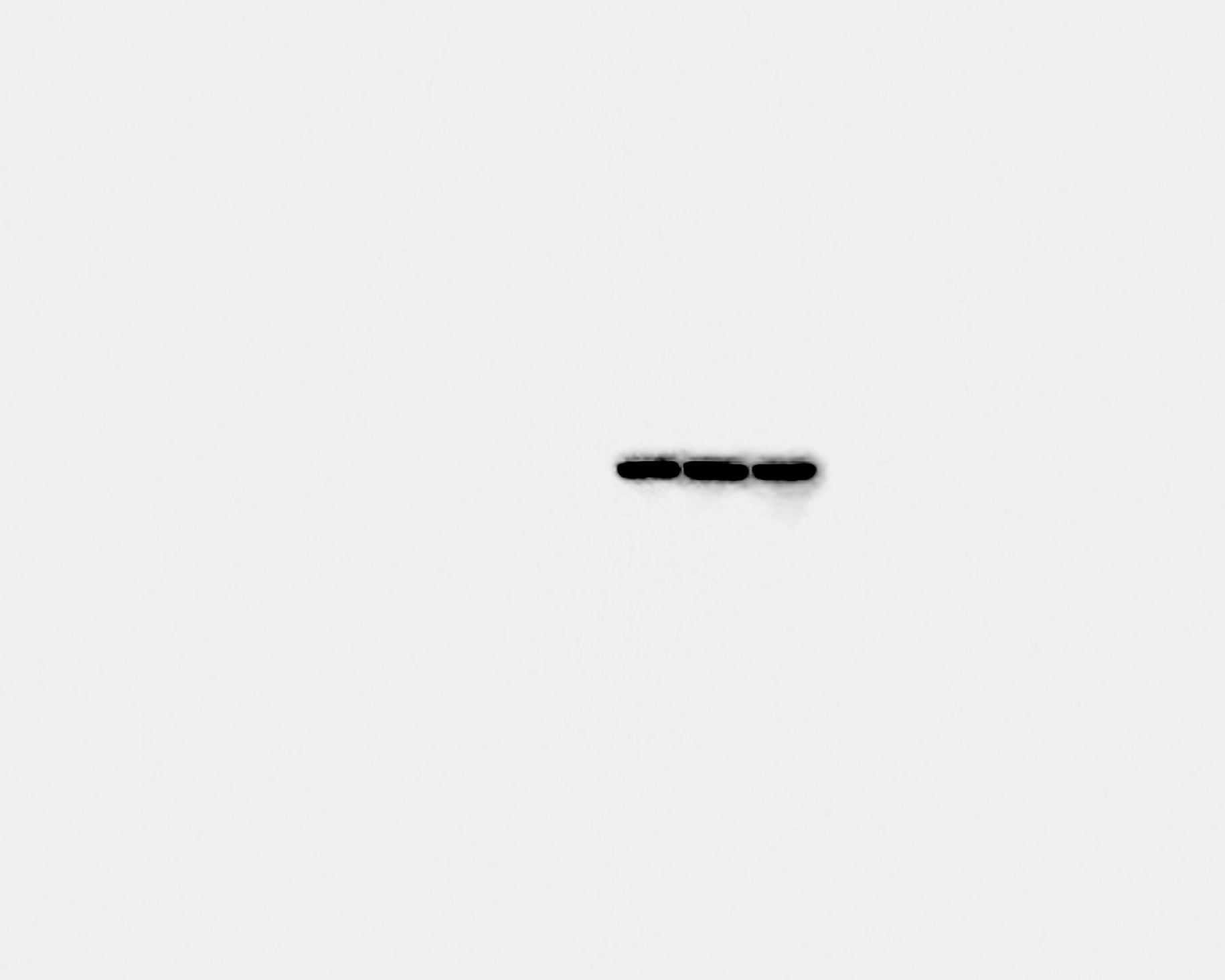

Supplement: Supplemental Information 1 [file peerj-13-19121-s001.zip › Figure 7/WB raw data/PC9/btnl9+gd 6_2(Chemiluminescence).tif]

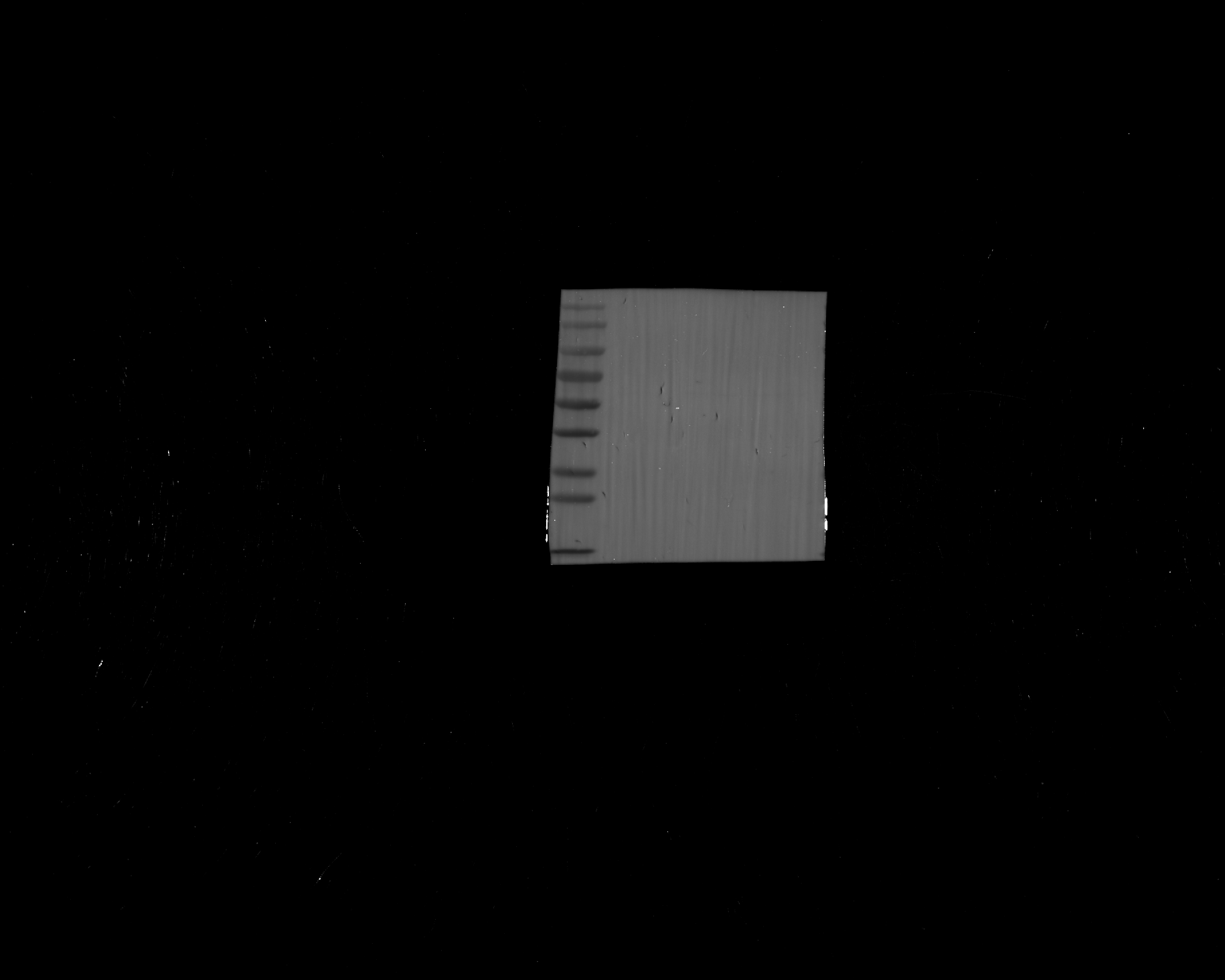

Supplement: Supplemental Information 1 [file peerj-13-19121-s001.zip › Figure 7/WB raw data/PC9/btnl9+gd 6_2(Colorimetric).tif]

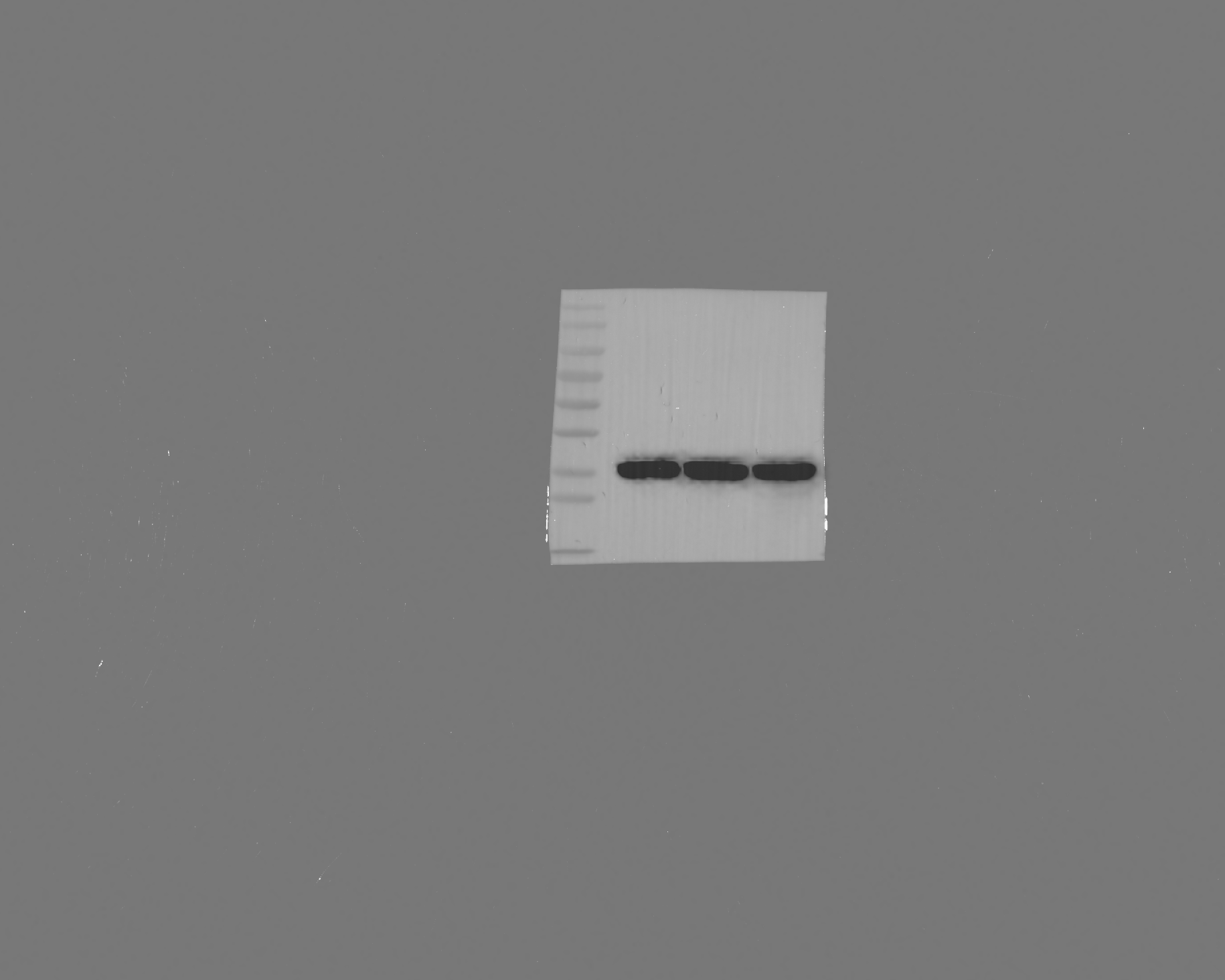

Supplement: Supplemental Information 1 [file peerj-13-19121-s001.zip › Figure 7/WB raw data/PC9/btnl9+gd 6_2(Composite).tif]

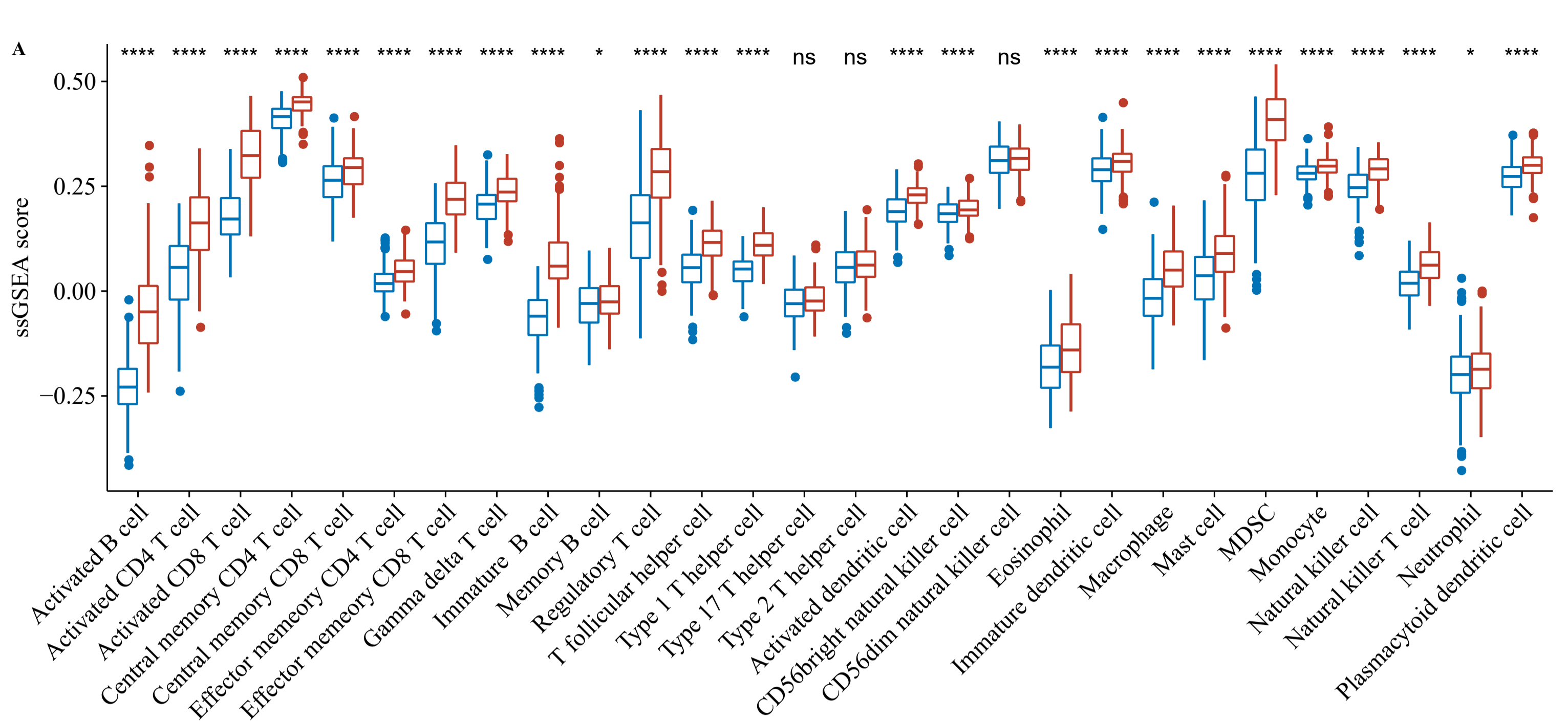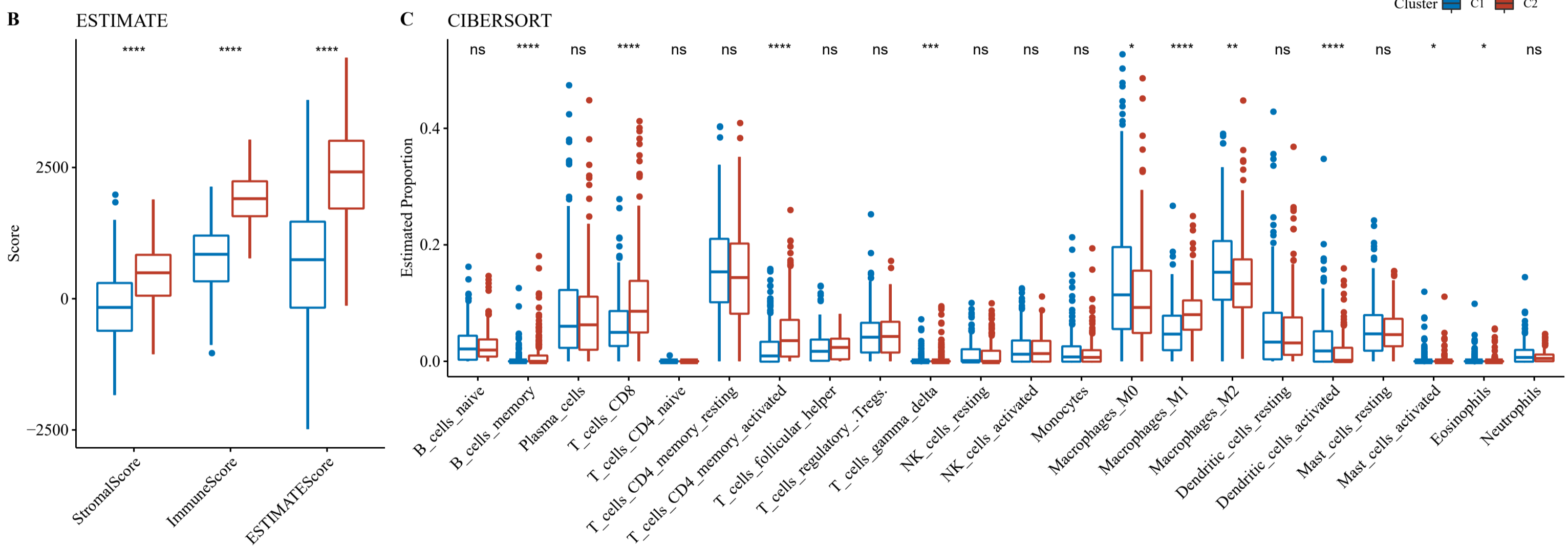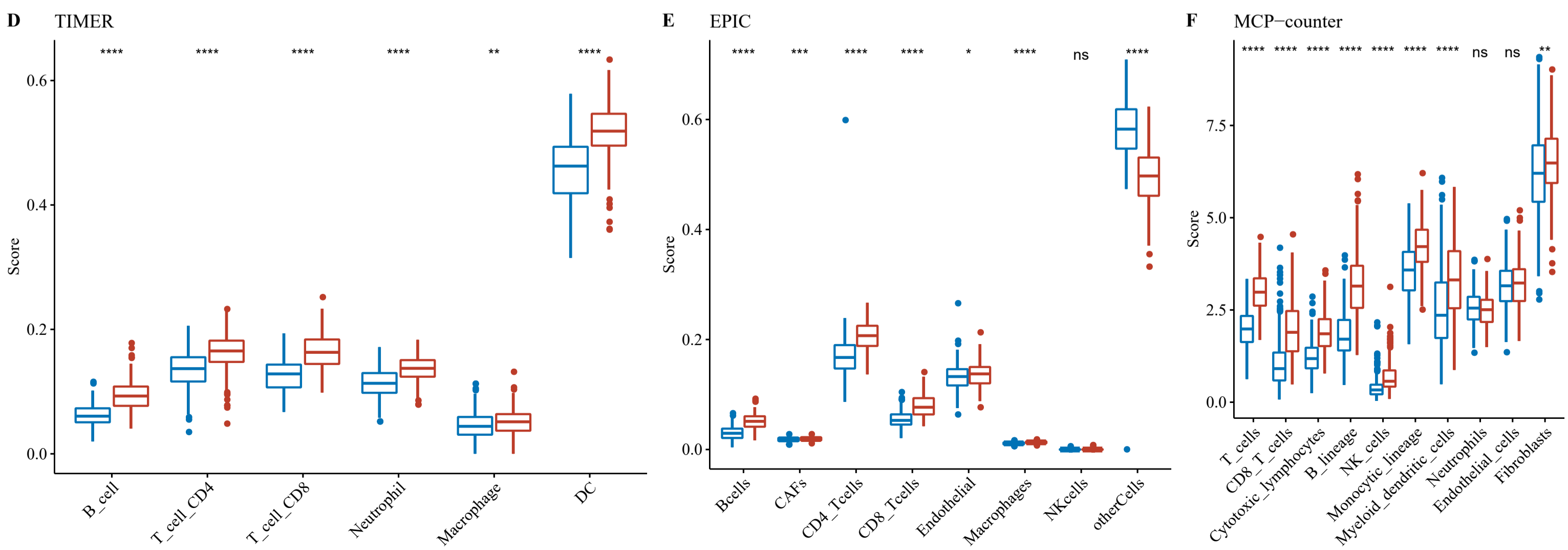

Supplement: Supplemental Information 2 — B. The distribution of immune score inferred by ESTIMATE algorithm between two clusters in the TCGA-LUAD cohort. C. The distribution of 22 immune cell subsets infiltration by CIBERSORT algorithm between two clusters in the TCGA-LUAD cohort. D. The distribution of 6 immune cell subsets infiltration by TIMER algorithm between two clusters in the TCGA-LUAD cohort. D. The distribution of 7 immune cell subsets infiltration by EPIC algorithm between two clusters in the TCGA-LUAD cohort. E. The distribution of 10 immune cell subsets infiltration by MCP-counter algorithm between two clusters in the TCGA-LUAD cohort. Here, * denotes p<0.05, ** denotes p<0.01, *** denotes p<0.001 and NS denotes p>0.05. [file peerj-13-19121-s002.pdf]

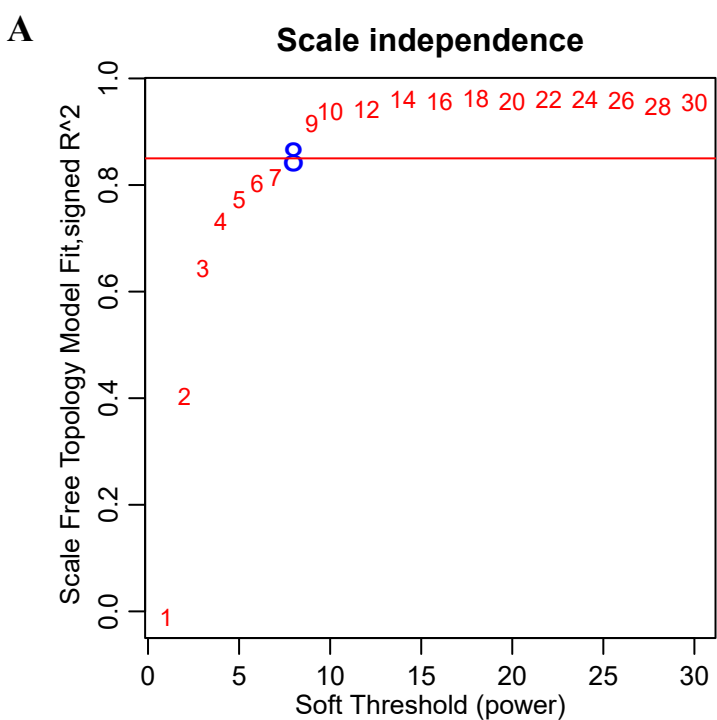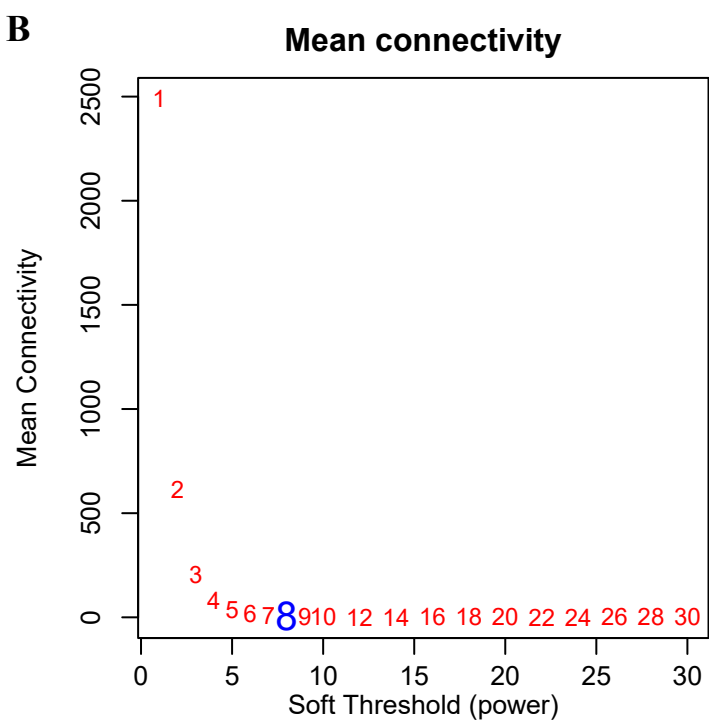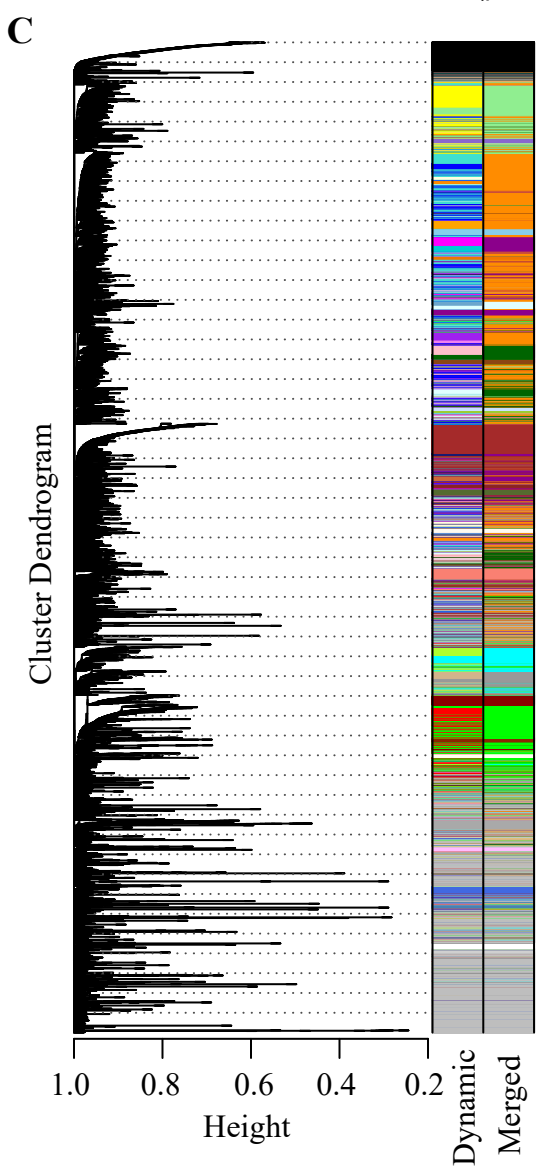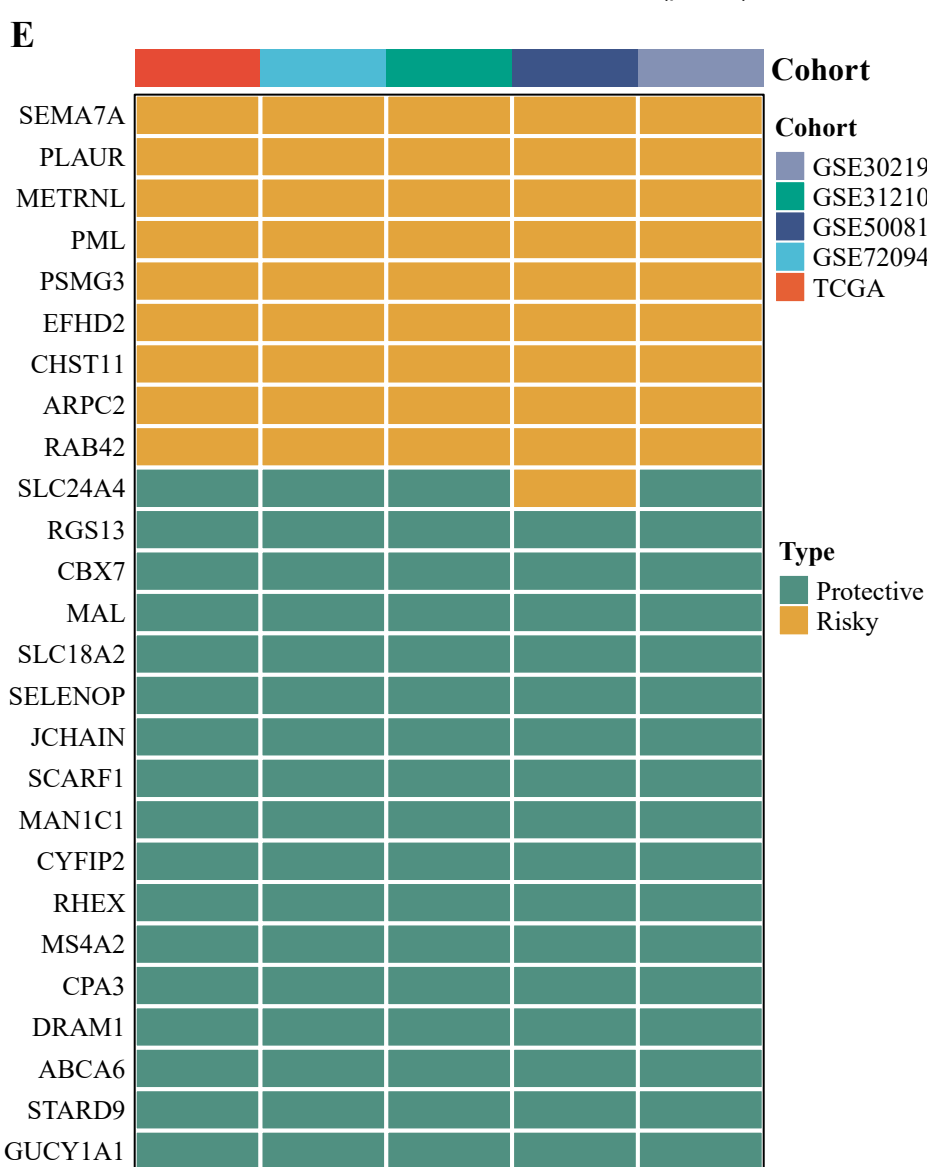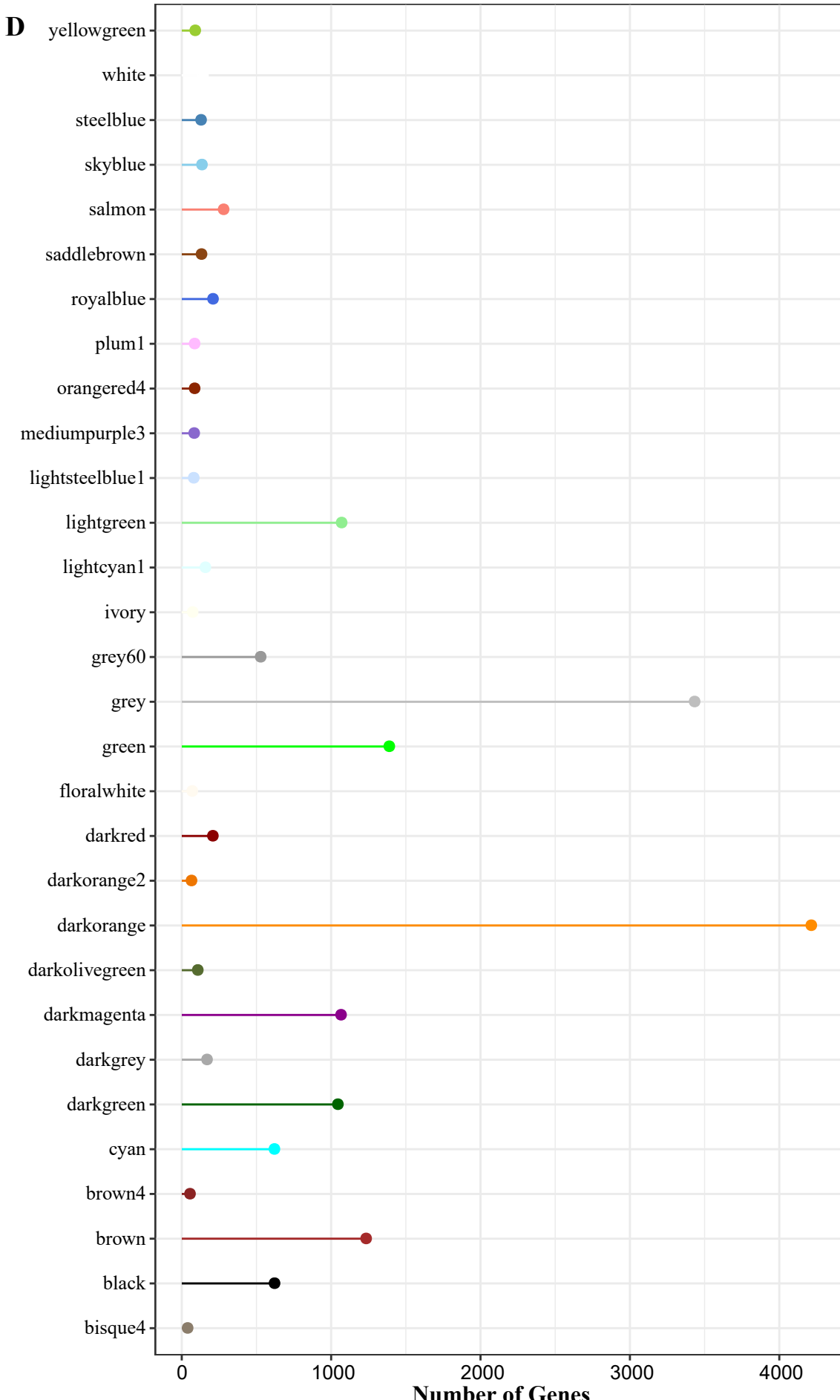

Supplement: Supplemental Information 3 — B. Analysis of the mean connectivity for various soft-thresholding powers. C. Heatmap of all differentially expressed genes clustered based on a dissimilarity measure (1-TOM). D. Distribution of the number of genes in each module. E. Risk status of the 26 genes in different cohorts. [file peerj-13-19121-s003.pdf]

## A TCGA

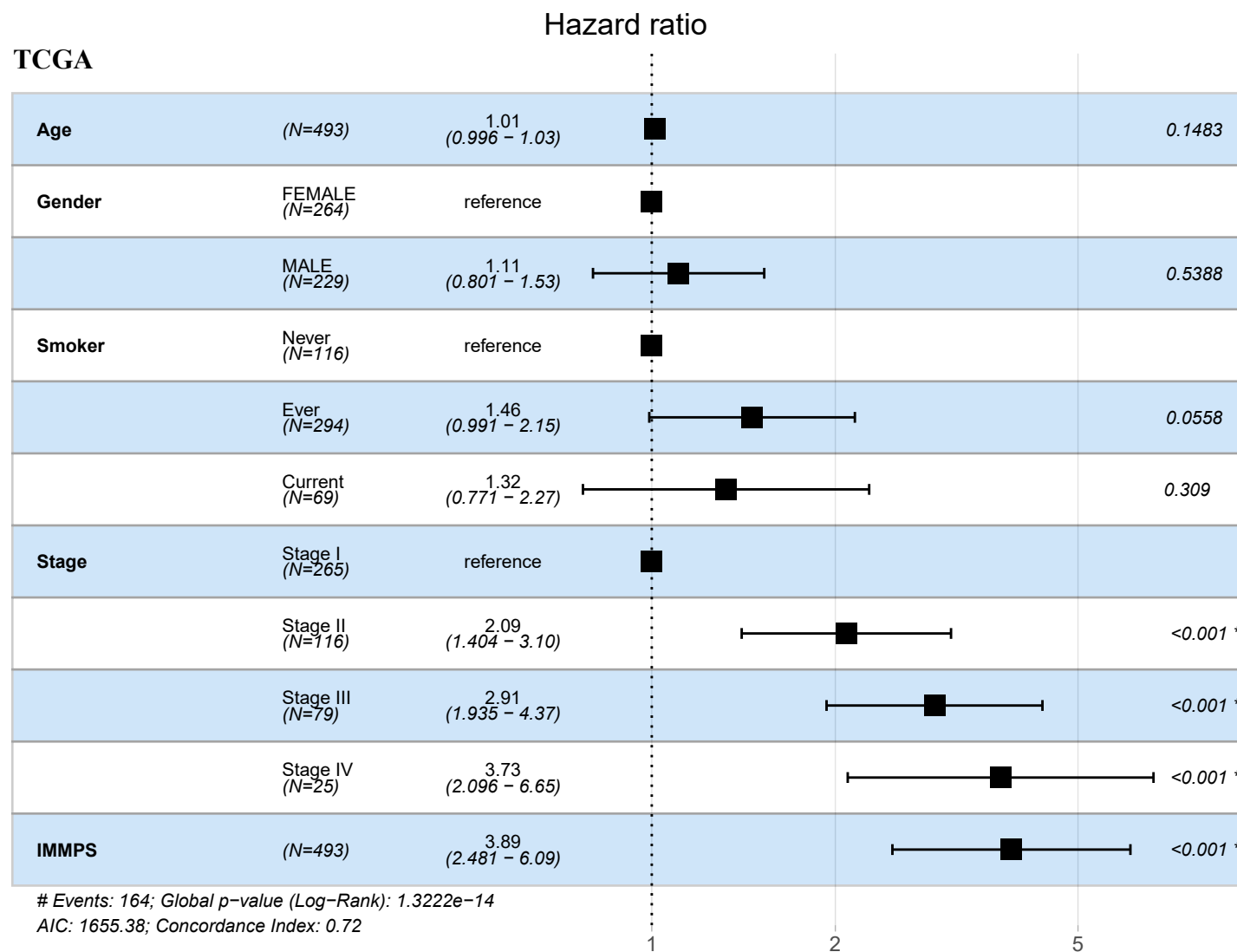

Supplement: Supplemental Information 4 [file peerj-13-19121-s004.pdf]

Group High Low

**A**

ESTIMATE

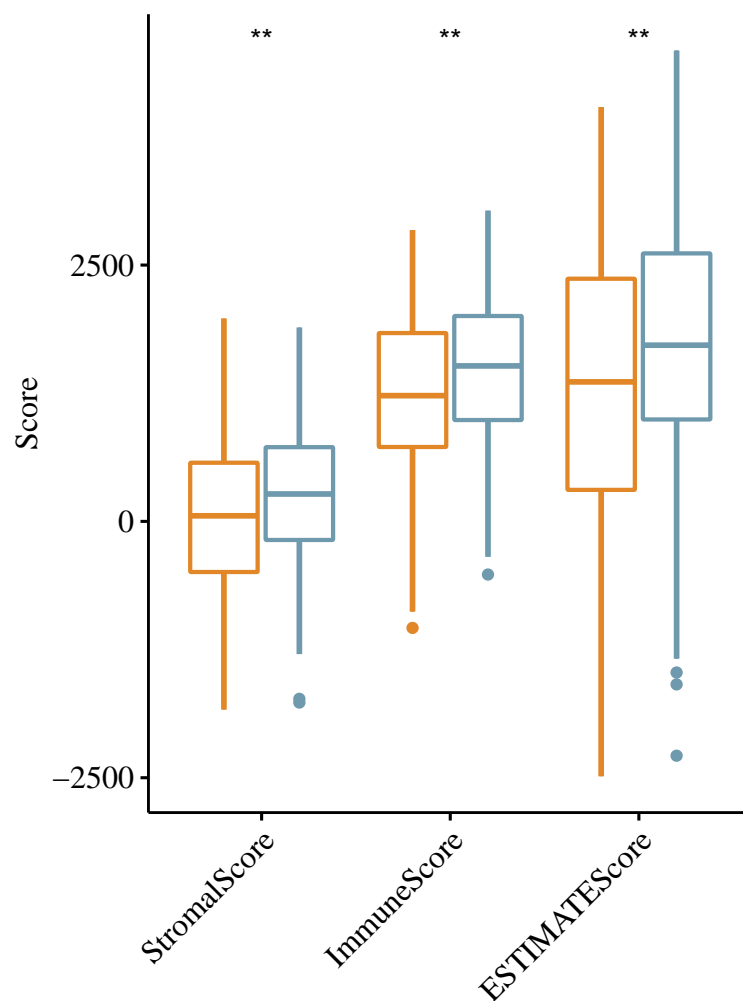

**B**

CIBERSORT

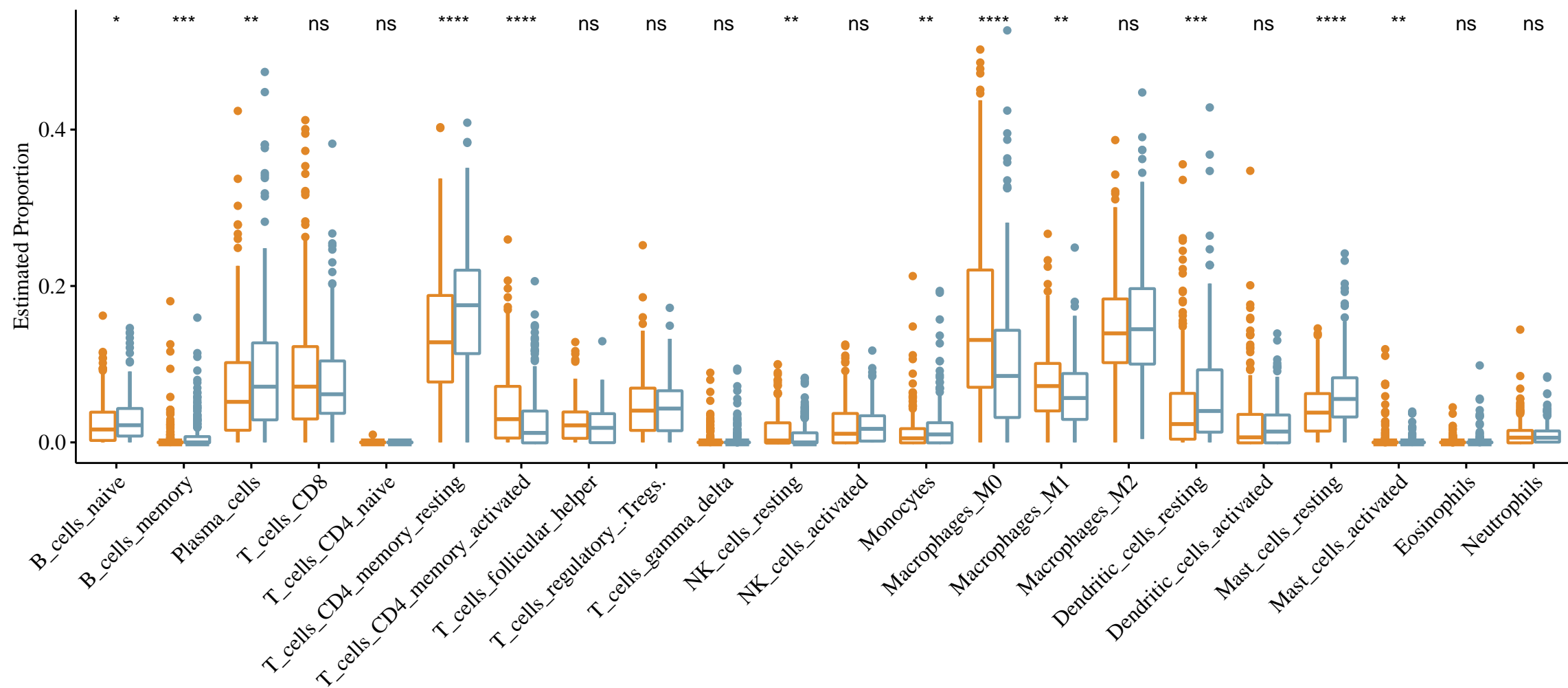

**C**

TIMER

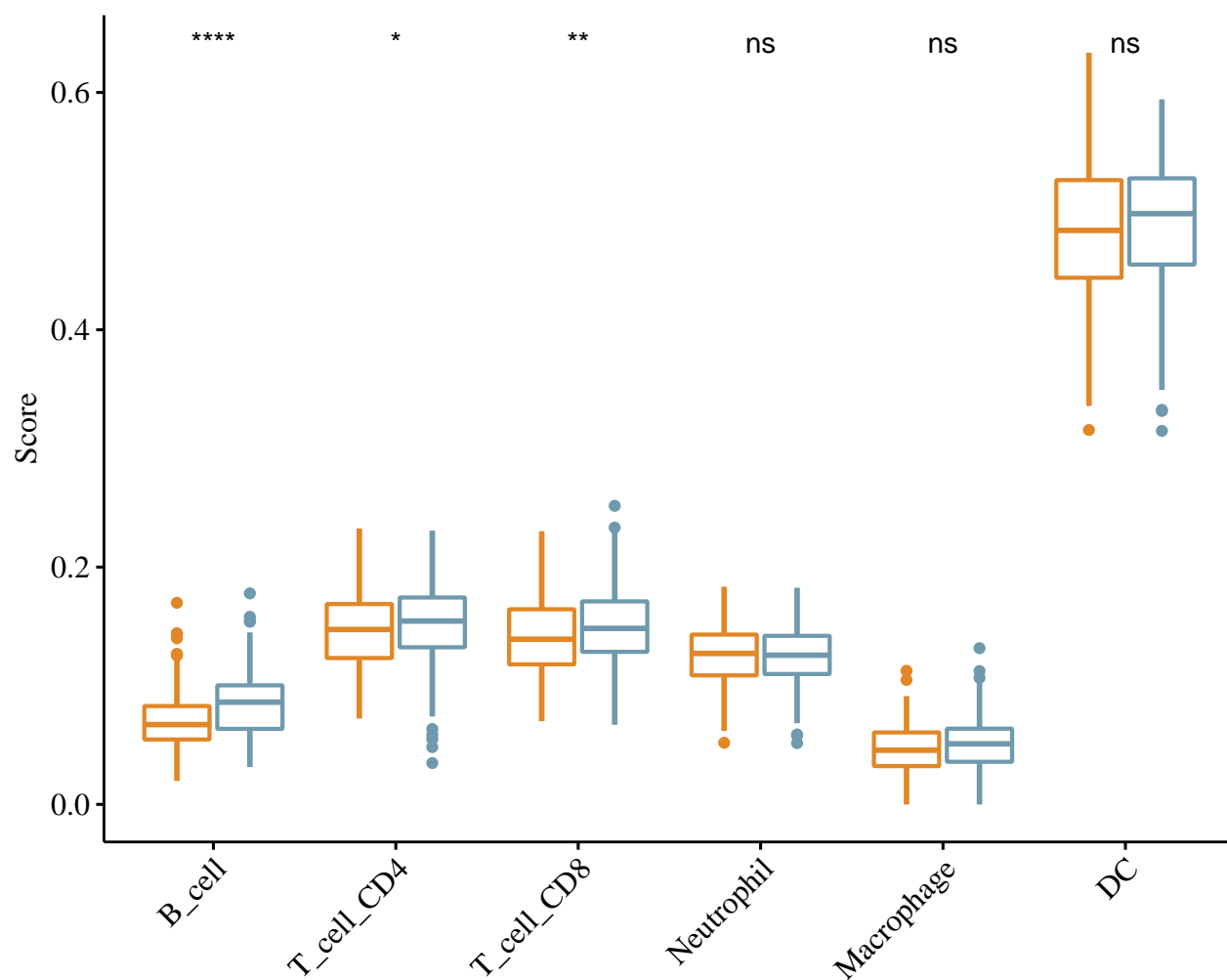

**D**

EPIC

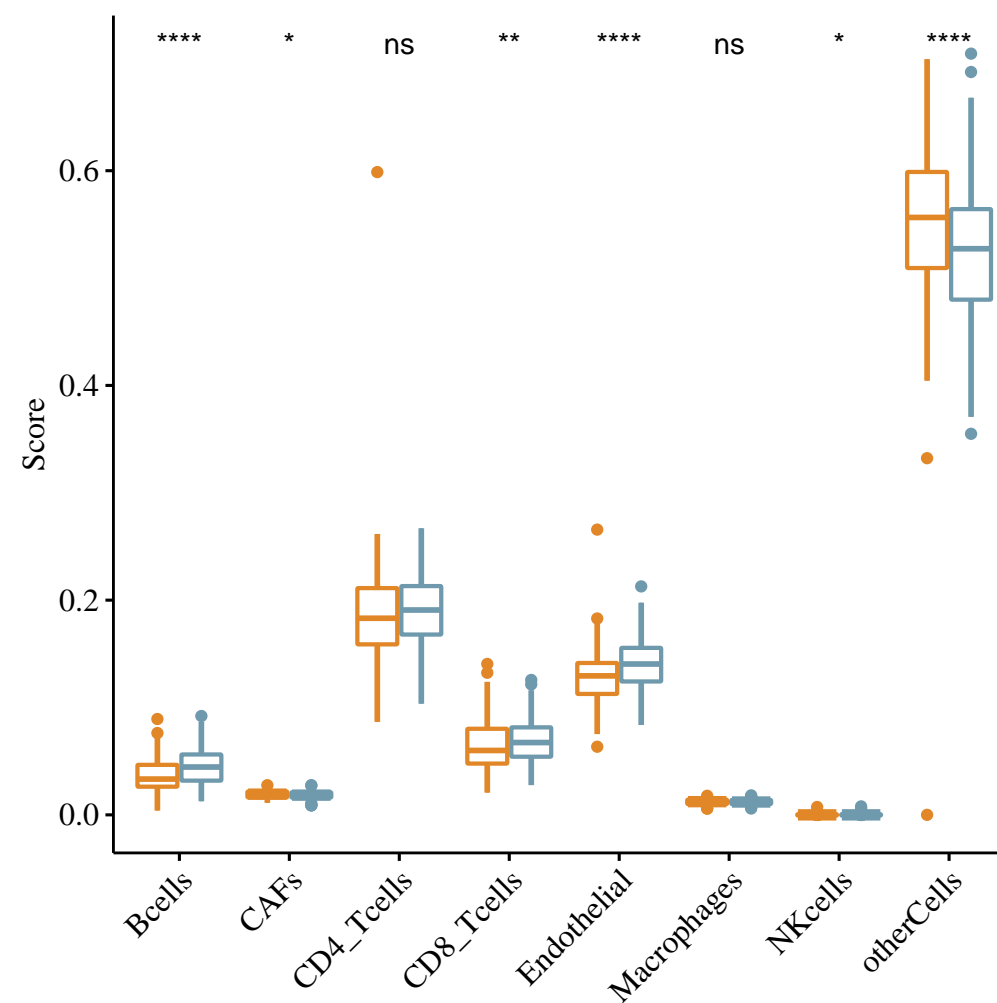

**E**

MCP-counter

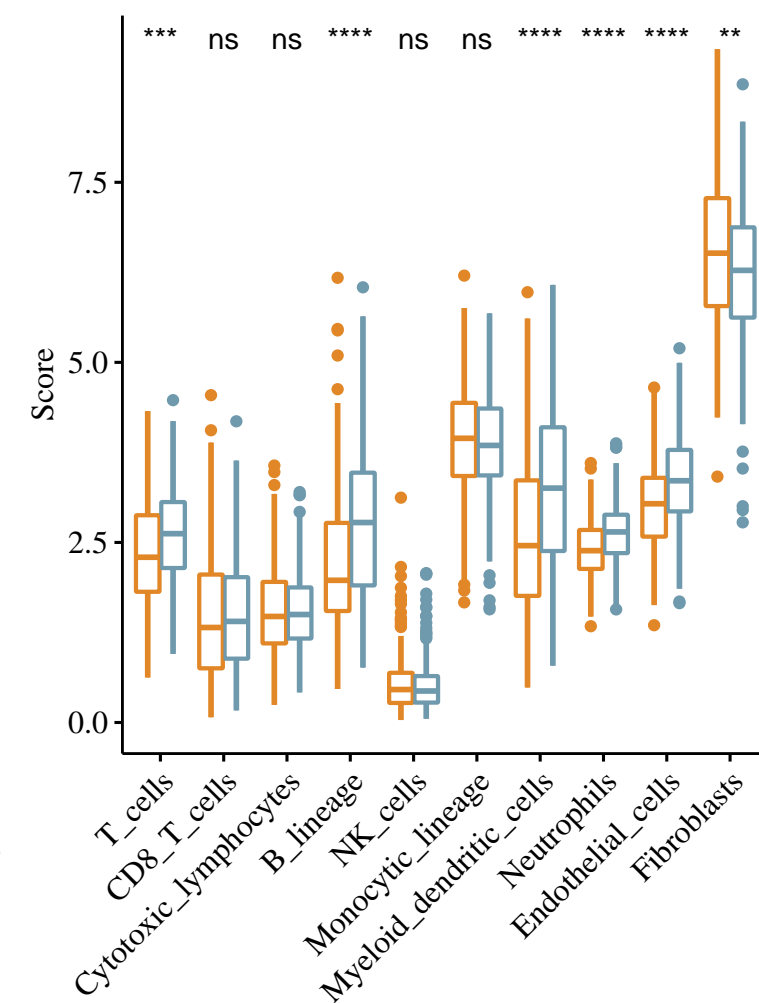

Supplement: Supplemental Information 6 — B. The distribution of 22 immune cell subsets infiltration by CIBERSORT algorithm between two clusters in the TCGA-LUAD cohort. C. The distribution of 6 immune cell subsets infiltration by TIMER algorithm between two clusters in the TCGA-LUAD cohort. D. The distribution of 7 immune cell subsets infiltration by EPIC algorithm between two clusters in the TCGA-LUAD cohort. E. The distribution of 10 immune cell subsets infiltration by MCP-counter algorithm between two clusters in the TCGA-LUAD cohort. *denotes p<0.05, ** denotes p<0.01, *** denotes p<0.001,NS denotes p>0.05. [file peerj-13-19121-s006.pdf]
