# Supplementary material for: Development of an immune-related gene signature applying Ridge method for improving immunotherapy responses and clinical outcomes in lung adenocarcinoma
Source: PeerJ. 2025 May 8;13:e19121. doi: 10.7717/peerj.19121 (PMC12066106; doi:10.7717/peerj.19121)
Supplement: Supplemental Information 7 [file peerj-13-19121-s007.docx]

| **Table S1. Summary of immune checkpoint genes collections.** | | | | | |
| --- | --- | --- | --- | --- | --- |
| Symbol | Type | Role with Immunity | Expression pattern demenstrate from FANTOM5 data | Family | Ligand receptor pairs |
| BTN2A2 | Ligand | Inhibit | TIC-ICGs | BTN | NA |
| BTNL3 | Ligand | TwoSide | TIC-ICGs | BTN | NA |
| BTNL9 | Ligand | TwoSide | TIC-ICGs | BTN | NA |
| CEACAM1 | Ligand&Receptor | Activate | TIC-ICGs | Other | NA |
| IDO1 | Ligand | Inhibit | TIC-ICGs | Other | NA |
| TDO2 | Ligand | Inhibit | TIC-ICGs | Other | NA |
| VTCN1 | Ligand | Inhibit | TIC-ICGs | Other | NA |
| ADORA2A | Receptor | Inhibit | IC-ICGs | Other | NA |
| BTN3A1 | Ligand | Activate | IC-ICGs | BTN | NA |
| C10orf54 | Ligand | Inhibit | IC-ICGs | Other | NA |
| CD276 | Ligand | Inhibit | TC-ICGs | Other | NA |
| CD274 | Ligand | TwoSide | TIC-ICGs | Other | Pair 1 |
| PDCD1LG2 | Ligand | TwoSide | TIC-ICGs | Other | Pair 1 |
| PDCD1 | Receptor | Inhibit | IC-ICGs | Other | Pair 1 |
| CD28 | Receptor | Activate | IC-ICGs | Other | Pair 2 |
| CD80 | Ligand | TwoSide | IC-ICGs | Other | Pair 2 |
| CD86 | Ligand | TwoSide | IC-ICGs | Other | Pair 2 |
| CTLA4 | Receptor | Inhibit | IC-ICGs | Other | Pair 2 |
| ICOS | Receptor | Activate | IC-ICGs | Other | Pair 3 |
| ICOSLG | Ligand | Activate | N | Other | Pair 3 |
| CD160 | Ligand | Inhibit | TIC-ICGs | Other | Pair 4 |
| BTLA | Receptor | Inhibit | IC-ICGs | Other | Pair 4 |
| TNFRSF14 | Ligand | TwoSide | IC-ICGs | Other | Pair 4 |
| TNFSF14 | Ligand | Activate | IC-ICGs | Other | Pair 4 |
| TNFSF9 | Ligand | Activate | TIC-ICGs | Other | Pair 5 |
| TNFRSF9 | Receptor | Activate | IC-ICGs | Other | Pair 5 |
| TNFSF4 | Ligand | Activate | TIC-ICGs | Other | Pair 6 |
| TNFRSF4 | Receptor | Activate | IC-ICGs | Other | Pair 6 |
| CD70 | Ligand | Activate | TIC-ICGs | Other | Pair 7 |
| CD27 | Receptor | Activate | IC-ICGs | Other | Pair 7 |
| CD40 | Receptor | Activate | IC-ICGs | Other | Pair 8 |
| CD40LG | Ligand | Activate | IC-ICGs | Other | Pair 8 |
| HAVCR2 | Receptor | Inhibit | TIC-ICGs | Other | Pair 9 |
| LGALS9 | Ligand | Inhibit | IC-ICGs | Other | Pair 9 |
| TNFSF18 | Ligand | Activate | TIC-ICGs | Other | Pair 10 |
| TNFRSF18 | Receptor | Activate | IC-ICGs | Other | Pair 10 |
| CD47 | Ligand | Inhibit | TIC-ICGs | Other | Pair 11 |
| SIRPA | Receptor | Inhibit | IC-ICGs | Other | Pair 11 |
| CD226 | Receptor | Activate | IC-ICGs | Other | Pair 12 |
| CD96 | Receptor | Activate | IC-ICGs | Other | Pair 12 |
| TIGIT | Receptor | Inhibit | IC-ICGs | Other | Pair 12 |
| PVR | Ligand | TwoSide | TC-ICGs | Other | Pair 12 |
| BTN2A1 | Ligand | TwoSide | TIC-ICGs | BTN | Pair 13 |
| CD209 | Ligand | TwoSide | TIC-ICGs | Other | Pair 13 |
| HLA-DOB | Ligand | TwoSide | TIC-ICGs | MHC class II | Pair 14 |
| HLA-G | Ligand | TwoSide | TIC-ICGs | MHC class I | Pair 14 |
| KIR2DL1 | Receptor | Inhibit | TIC-ICGs | KIR_inhibit | Pair 14 |
| KIR2DL2 | Receptor | Inhibit | TIC-ICGs | KIR_inhibit | Pair 14 |
| KIR2DL3 | Receptor | Inhibit | TIC-ICGs | KIR_inhibit | Pair 14 |
| KIR2DL4 | Receptor | Activate | TIC-ICGs | KIR_activate | Pair 14 |
| KIR2DS2 | Receptor | Activate | TIC-ICGs | KIR_activate | Pair 14 |
| KIR3DL2 | Receptor | Inhibit | TIC-ICGs | KIR_inhibit | Pair 14 |
| HLA-A | Ligand | TwoSide | IC-ICGs | MHC class I | Pair 14 |
| HLA-B | Ligand | TwoSide | IC-ICGs | MHC class I | Pair 14 |
| HLA-C | Ligand | TwoSide | IC-ICGs | MHC class I | Pair 14 |
| HLA-DMA | Ligand | TwoSide | IC-ICGs | MHC class II | Pair 14 |
| HLA-DMB | Ligand | TwoSide | IC-ICGs | MHC class II | Pair 14 |
| HLA-DOA | Ligand | TwoSide | IC-ICGs | MHC class II | Pair 14 |
| HLA-DPA1 | Ligand | TwoSide | IC-ICGs | MHC class II | Pair 14 |
| HLA-DPB1 | Ligand | TwoSide | IC-ICGs | MHC class II | Pair 14 |
| HLA-DQA1 | Ligand | TwoSide | IC-ICGs | MHC class II | Pair 14 |
| HLA-DQB1 | Ligand | TwoSide | IC-ICGs | MHC class II | Pair 14 |
| HLA-DRA | Ligand | TwoSide | IC-ICGs | MHC class II | Pair 14 |
| HLA-DRB1 | Ligand | TwoSide | IC-ICGs | MHC class II | Pair 14 |
| HLA-DRB4 | Ligand | TwoSide | IC-ICGs | MHC class II | Pair 14 |
| HLA-DRB5 | Ligand | TwoSide | IC-ICGs | MHC class II | Pair 14 |
| HLA-E | Ligand | TwoSide | IC-ICGs | MHC class I | Pair 14 |
| HLA-F | Ligand | TwoSide | IC-ICGs | MHC class I | Pair 14 |
| LAG3 | Receptor | Inhibit | IC-ICGs | Other | Pair 14 |
| HLA-DRB3 | Ligand | TwoSide | N | MHC class II | Pair 14 |
| KIR2DL5A | Receptor | Inhibit | N | KIR_inhibit | Pair 14 |
| KIR2DL5B | Receptor | Inhibit | N | KIR_inhibit | Pair 14 |
| KIR2DS1 | Receptor | Activate | N | KIR_activate | Pair 14 |
| KIR2DS3 | Receptor | Activate | N | KIR_activate | Pair 14 |
| KIR2DS4 | Receptor | Activate | N | KIR_activate | Pair 14 |
| KIR2DS5 | Receptor | Activate | N | KIR_activate | Pair 14 |
| KIR3DL1 | Receptor | Inhibit | N | KIR_inhibit | Pair 14 |
| KIR3DL3 | Receptor | Inhibit | N | KIR_inhibit | Pair 14 |
| KIR3DS1 | Receptor | Activate | N | KIR_activate | Pair 14 |
