# Supplementary material for: Development of an immune-related gene signature applying Ridge method for improving immunotherapy responses and clinical outcomes in lung adenocarcinoma
Source: PeerJ. 2025 May 8;13:e19121. doi: 10.7717/peerj.19121 (PMC12066106; doi:10.7717/peerj.19121)
Supplement: Supplemental Information 8 [file peerj-13-19121-s008.docx]

| **Table S2 Data on a total of 154 previous gene signatures** | | | | | | |
| --- | --- | --- | --- | --- | --- | --- |
| **Model** | **PMID** | **Author** | **Year** | **Type** | **Coef** | **Gene** |
| Model-1 | 34234827 | Bai M | 2021 | mRNA | -0.004 | CD53 |
| Model-1 | 34234827 | Bai M | 2021 | mRNA | -0.014 | SASH3 |
| Model-2 | 33193873 | Zhang J | 2020 | mRNA | 0.012119 | TYMS |
| Model-2 | 33193873 | Zhang J | 2020 | mRNA | -0.00056 | ALDH2 |
| Model-2 | 33193873 | Zhang J | 2020 | mRNA | 0.000501 | PKM |
| Model-2 | 33193873 | Zhang J | 2020 | mRNA | 0.022241 | GNPNAT1 |
| Model-2 | 33193873 | Zhang J | 2020 | mRNA | 0.002514 | LDHA |
| Model-2 | 33193873 | Zhang J | 2020 | mRNA | 0.061361 | ENTPD2 |
| Model-2 | 33193873 | Zhang J | 2020 | mRNA | 0.000165 | NT5E |
| Model-2 | 33193873 | Zhang J | 2020 | mRNA | -0.00403 | MAOB |
| Model-3 | 37025770 | Liu SY | 2023 | mRNA | 0.319807 | HSP90AA1 |
| Model-3 | 37025770 | Liu SY | 2023 | mRNA | -0.45711 | IL10 |
| Model-3 | 37025770 | Liu SY | 2023 | mRNA | 0.140221 | NT5E |
| Model-4 | 34178026 | Guo B | 2021 | mRNA | 1.725151 | ZCRB1 |
| Model-4 | 34178026 | Guo B | 2021 | mRNA | -1.96433 | ADH1C |
| Model-4 | 34178026 | Guo B | 2021 | mRNA | -2.01538 | YTHDC2 |
| Model-5 | 37169018 | Zhou Y | 2023 | mRNA | 0.3342 | ANGPTL4 |
| Model-5 | 37169018 | Zhou Y | 2023 | mRNA | 0.13667 | ITGB4 |
| Model-5 | 37169018 | Zhou Y | 2023 | mRNA | -0.33534 | COL13A1 |
| Model-5 | 37169018 | Zhou Y | 2023 | mRNA | 0.07749 | KRT14 |
| Model-5 | 37169018 | Zhou Y | 2023 | mRNA | 0.14982 | RAC3 |
| Model-5 | 37169018 | Zhou Y | 2023 | mRNA | 0.18313 | CDX2 |
| Model-5 | 37169018 | Zhou Y | 2023 | mRNA | 0.22797 | KIF18A |
| Model-6 | 36249053 | Shang X | 2022 | mRNA | -0.03942 | B3GALT2 |
| Model-6 | 36249053 | Shang X | 2022 | mRNA | -0.11329 | C17orf44 |
| Model-6 | 36249053 | Shang X | 2022 | mRNA | 0.060184 | CCT6A |
| Model-6 | 36249053 | Shang X | 2022 | mRNA | -0.00628 | CD40LG |
| Model-6 | 36249053 | Shang X | 2022 | mRNA | 0.061717 | FKBP4 |
| Model-6 | 36249053 | Shang X | 2022 | mRNA | -0.03694 | GNG7 |
| Model-6 | 36249053 | Shang X | 2022 | mRNA | 0.049301 | H2AFZ |
| Model-6 | 36249053 | Shang X | 2022 | mRNA | 0.027526 | IGF2BP1 |
| Model-6 | 36249053 | Shang X | 2022 | mRNA | -0.07839 | IVD |
| Model-6 | 36249053 | Shang X | 2022 | mRNA | -0.02033 | RCBTB2 |
| Model-6 | 36249053 | Shang X | 2022 | mRNA | -0.00623 | SLC34A2 |
| Model-7 | 36699465 | Wang GC | 2023 | mRNA | -0.08952 | VPREB3 |
| Model-7 | 36699465 | Wang GC | 2023 | mRNA | 0.130109 | LRFN4 |
| Model-7 | 36699465 | Wang GC | 2023 | mRNA | 0.07892 | F12 |
| Model-7 | 36699465 | Wang GC | 2023 | mRNA | -0.08407 | PRMT8 |
| Model-7 | 36699465 | Wang GC | 2023 | mRNA | 0.085357 | TPBG |
| Model-7 | 36699465 | Wang GC | 2023 | mRNA | -0.03849 | GIMAP6 |
| Model-7 | 36699465 | Wang GC | 2023 | mRNA | -0.08783 | CD83 |
| Model-7 | 36699465 | Wang GC | 2023 | mRNA | 0.043966 | WASF1 |
| Model-8 | 36093548 | You Q | 2022 | mRNA | 0.185096 | OAS3 |
| Model-8 | 36093548 | You Q | 2022 | mRNA | -0.3872 | PCF11 |
| Model-8 | 36093548 | You Q | 2022 | mRNA | -0.15166 | TLR7 |
| Model-8 | 36093548 | You Q | 2022 | mRNA | 0.214582 | EXO1 |
| Model-9 | 36093538 | Wu C | 2022 | mRNA | -0.2827 | NLRC4 |
| Model-9 | 36093538 | Wu C | 2022 | mRNA | -0.1227 | NLRP1 |
| Model-9 | 36093538 | Wu C | 2022 | mRNA | -0.157 | NOD1 |
| Model-9 | 36093538 | Wu C | 2022 | mRNA | -0.103 | PLCG1 |
| Model-9 | 36093538 | Wu C | 2022 | mRNA | 0.2021 | BAK1 |
| Model-10 | 31886214 | Xie H | 2019 | mRNA | -0.2491 | RRAGB |
| Model-10 | 31886214 | Xie H | 2019 | mRNA | -0.0679 | RSPH9 |
| Model-10 | 31886214 | Xie H | 2019 | mRNA | -0.2317 | RPS6KL1 |
| Model-10 | 31886214 | Xie H | 2019 | mRNA | -0.1035 | RXFP1 |
| Model-10 | 31886214 | Xie H | 2019 | mRNA | 0.1571 | RRM2 |
| Model-10 | 31886214 | Xie H | 2019 | mRNA | 0.1104 | RTL1 |
| Model-11 | 36703749 | Zhou HM | 2023 | mRNA | 0.215111 | FZD4 |
| Model-11 | 36703749 | Zhou HM | 2023 | mRNA | 0.231112 | FZD7 |
| Model-11 | 36703749 | Zhou HM | 2023 | mRNA | -0.15082 | LEF1 |
| Model-11 | 36703749 | Zhou HM | 2023 | mRNA | -0.10536 | FZD9 |
| Model-11 | 36703749 | Zhou HM | 2023 | mRNA | -0.22314 | CTNNBIP1 |
| Model-11 | 36703749 | Zhou HM | 2023 | mRNA | -0.15082 | AXIN1 |
| Model-11 | 36703749 | Zhou HM | 2023 | mRNA | 0.076961 | DKK4 |
| Model-11 | 36703749 | Zhou HM | 2023 | mRNA | 0.285179 | CSNK2A1 |
| Model-11 | 36703749 | Zhou HM | 2023 | mRNA | 0.14842 | TBL1Y |
| Model-11 | 36703749 | Zhou HM | 2023 | mRNA | -0.21072 | NFATC1 |
| Model-11 | 36703749 | Zhou HM | 2023 | mRNA | -0.18633 | PLCB2 |
| Model-11 | 36703749 | Zhou HM | 2023 | mRNA | 0.231112 | PLCB3 |
| Model-11 | 36703749 | Zhou HM | 2023 | mRNA | 0.19062 | PRKCG |
| Model-11 | 36703749 | Zhou HM | 2023 | mRNA | 0.10436 | FOSL1 |
| Model-11 | 36703749 | Zhou HM | 2023 | mRNA | 0.215111 | PSEN1 |
| Model-11 | 36703749 | Zhou HM | 2023 | mRNA | -0.16252 | CTNNB1 |
| Model-11 | 36703749 | Zhou HM | 2023 | mRNA | 0.625938 | PPARD |
| Model-11 | 36703749 | Zhou HM | 2023 | mRNA | -0.13926 | FZD2 |
| Model-12 | 36942234 | Sun K | 2023 | mRNA | -0.185 | LIFR |
| Model-12 | 36942234 | Sun K | 2023 | mRNA | -0.197 | FGR |
| Model-12 | 36942234 | Sun K | 2023 | mRNA | -0.127 | WFDC2 |
| Model-12 | 36942234 | Sun K | 2023 | mRNA | 0.164 | SEMA4B |
| Model-12 | 36942234 | Sun K | 2023 | mRNA | 0.728 | FGF2 |
| Model-12 | 36942234 | Sun K | 2023 | mRNA | 0.151 | CCL20 |
| Model-12 | 36942234 | Sun K | 2023 | mRNA | -0.211 | GDF10 |
| Model-13 | 36575346 | Li Q | 2023 | mRNA | 0.084365 | HSP90AA1 |
| Model-13 | 36575346 | Li Q | 2023 | mRNA | 0.102208 | EIF2S1 |
| Model-13 | 36575346 | Li Q | 2023 | mRNA | 0.257698 | PLK1 |
| Model-14 | 32508318 | Zhang Y | 2020 | mRNA | -0.08 | AZGP1 |
| Model-14 | 32508318 | Zhang Y | 2020 | mRNA | 0.24 | GUCA2A |
| Model-14 | 32508318 | Zhang Y | 2020 | mRNA | 0.03 | HTR1B |
| Model-14 | 32508318 | Zhang Y | 2020 | mRNA | -0.18 | INPP5J |
| Model-14 | 32508318 | Zhang Y | 2020 | mRNA | -0.12 | MYBPH |
| Model-14 | 32508318 | Zhang Y | 2020 | mRNA | 0.02 | SLC15A1 |
| Model-14 | 32508318 | Zhang Y | 2020 | mRNA | -0.13 | SPIB |
| Model-14 | 32508318 | Zhang Y | 2020 | mRNA | 0.03 | TNFSF11 |
| Model-15 | 36447119 | Zhang Y | 2023 | mRNA | 0.0717 | FLNC |
| Model-15 | 36447119 | Zhang Y | 2023 | mRNA | 0.0025 | FBN2 |
| Model-15 | 36447119 | Zhang Y | 2023 | mRNA | 0.0054 | CCL20 |
| Model-15 | 36447119 | Zhang Y | 2023 | mRNA | 0.1881 | NTSR1 |
| Model-15 | 36447119 | Zhang Y | 2023 | mRNA | 0.0265 | KRT6A |
| Model-15 | 36447119 | Zhang Y | 2023 | mRNA | 0.0869 | DKK1 |
| Model-15 | 36447119 | Zhang Y | 2023 | mRNA | 0.0004 | KYNU |
| Model-15 | 36447119 | Zhang Y | 2023 | mRNA | 0.0605 | TENM3 |
| Model-15 | 36447119 | Zhang Y | 2023 | mRNA | 0.0011 | ANGPTL4 |
| Model-15 | 36447119 | Zhang Y | 2023 | mRNA | -0.0952 | STAP1 |
| Model-15 | 36447119 | Zhang Y | 2023 | mRNA | 0.0606 | HMMR |
| Model-15 | 36447119 | Zhang Y | 2023 | mRNA | 0.0282 | IGFBP1 |
| Model-15 | 36447119 | Zhang Y | 2023 | mRNA | -0.0013 | C11orf16 |
| Model-15 | 36447119 | Zhang Y | 2023 | mRNA | 0.0623 | LDHA |
| Model-15 | 36447119 | Zhang Y | 2023 | mRNA | 0.0209 | PLEK2 |
| Model-16 | 36178365 | Fei Y | 2022 | mRNA | -1.08349 | EIF2AK3 |
| Model-16 | 36178365 | Fei Y | 2022 | mRNA | 0.518306 | ITGB1 |
| Model-17 | 32019546 | Shi X | 2020 | mRNA | -0.4343 | MAP3K8 |
| Model-17 | 32019546 | Shi X | 2020 | mRNA | 0.231231 | VEGFC |
| Model-17 | 32019546 | Shi X | 2020 | mRNA | 0.126647 | CCL20 |
| Model-17 | 32019546 | Shi X | 2020 | mRNA | 0.121578 | ANGPTL4 |
| Model-18 | 36072012 | Yang F | 2022 | mRNA | -0.14513 | CD69 |
| Model-18 | 36072012 | Yang F | 2022 | mRNA | -0.10314 | CLIC6 |
| Model-18 | 36072012 | Yang F | 2022 | mRNA | 0.28765 | CTSL |
| Model-18 | 36072012 | Yang F | 2022 | mRNA | -0.20727 | EPHX1 |
| Model-18 | 36072012 | Yang F | 2022 | mRNA | 0.101089 | LMO3 |
| Model-18 | 36072012 | Yang F | 2022 | mRNA | -0.27912 | MS4A7 |
| Model-19 | 36575396 | Liang JX | 2022 | mRNA | 0.096219 | B3GNT3 |
| Model-19 | 36575396 | Liang JX | 2022 | mRNA | -0.23699 | MFNG |
| Model-19 | 36575396 | Liang JX | 2022 | mRNA | -0.21816 | GYLTL1B |
| Model-19 | 36575396 | Liang JX | 2022 | mRNA | 0.362558 | ALG3 |
| Model-19 | 36575396 | Liang JX | 2022 | mRNA | 0.306013 | GALNT13 |
| Model-20 | 36650426 | Feng HM | 2023 | mRNA | -1.0068 | NFE2 |
| Model-20 | 36650426 | Feng HM | 2023 | mRNA | 0.2741 | MMP12 |
| Model-20 | 36650426 | Feng HM | 2023 | mRNA | 0.5986 | HOXC8 |
| Model-21 | 33447073 | Yao Y | 2021 | mRNA | 0.14842 | FOXN4 |
| Model-21 | 33447073 | Yao Y | 2021 | mRNA | 0.277632 | KLHL4 |
| Model-21 | 33447073 | Yao Y | 2021 | mRNA | 0.067659 | FAM83F |
| Model-21 | 33447073 | Yao Y | 2021 | mRNA | -0.16252 | CCR2 |
| Model-22 | 35281055 | Che Y | 2022 | mRNA | 0.34 | USP29 |
| Model-22 | 35281055 | Che Y | 2022 | mRNA | -0.2 | MPP7 |
| Model-22 | 35281055 | Che Y | 2022 | mRNA | 0.06 | TRIM40 |
| Model-22 | 35281055 | Che Y | 2022 | mRNA | -0.21 | HERC1 |
| Model-22 | 35281055 | Che Y | 2022 | mRNA | 0.36 | TLE1 |
| Model-22 | 35281055 | Che Y | 2022 | mRNA | -0.2 | ASB2 |
| Model-22 | 35281055 | Che Y | 2022 | mRNA | 0.41 | NEDD1 |
| Model-22 | 35281055 | Che Y | 2022 | mRNA | -0.1 | USP44 |
| Model-22 | 35281055 | Che Y | 2022 | mRNA | -0.22 | PHF1 |
| Model-23 | 36086730 | Li H | 2022 | mRNA | -0.149 | SLC18A2 |
| Model-23 | 36086730 | Li H | 2022 | mRNA | 0.04 | STX1A |
| Model-23 | 36086730 | Li H | 2022 | mRNA | -0.107 | UNC13B |
| Model-24 | 32298601 | Zhang S | 2020 | mRNA | 0.003126 | LDHA |
| Model-24 | 32298601 | Zhang S | 2020 | mRNA | 0.004761 | LPGAT1 |
| Model-24 | 32298601 | Zhang S | 2020 | mRNA | 0.020552 | GNPNAT1 |
| Model-24 | 32298601 | Zhang S | 2020 | mRNA | 0.000102 | PTGES |
| Model-24 | 32298601 | Zhang S | 2020 | mRNA | 0.008317 | TYMS |
| Model-24 | 32298601 | Zhang S | 2020 | mRNA | -4.3E-05 | ALDH2 |
| Model-25 | 33200655 | Huang W | 2020 | mRNA | -0.05166 | AADAT |
| Model-25 | 33200655 | Huang W | 2020 | mRNA | -0.16512 | ADH1C |
| Model-25 | 33200655 | Huang W | 2020 | mRNA | 0.131905 | AKR7A3 |
| Model-25 | 33200655 | Huang W | 2020 | mRNA | 0.046728 | CALML3 |
| Model-25 | 33200655 | Huang W | 2020 | mRNA | 0.12055 | CDK1 |
| Model-25 | 33200655 | Huang W | 2020 | mRNA | 0.00252 | DES |
| Model-25 | 33200655 | Huang W | 2020 | mRNA | -0.38803 | EHD3 |
| Model-25 | 33200655 | Huang W | 2020 | mRNA | -0.26539 | ENO3 |
| Model-25 | 33200655 | Huang W | 2020 | mRNA | -0.24554 | FBP2 |
| Model-25 | 33200655 | Huang W | 2020 | mRNA | -0.04172 | GPD1 |
| Model-25 | 33200655 | Huang W | 2020 | mRNA | 0.163857 | IL33 |
| Model-25 | 33200655 | Huang W | 2020 | mRNA | 0.259559 | LOXL2 |
| Model-25 | 33200655 | Huang W | 2020 | mRNA | 0.153385 | MDK |
| Model-25 | 33200655 | Huang W | 2020 | mRNA | 0.303236 | MKI67 |
| Model-25 | 33200655 | Huang W | 2020 | mRNA | 0.046807 | P4HA1 |
| Model-25 | 33200655 | Huang W | 2020 | mRNA | 0.144675 | P4HA2 |
| Model-25 | 33200655 | Huang W | 2020 | mRNA | -0.07404 | PGM5 |
| Model-25 | 33200655 | Huang W | 2020 | mRNA | -0.02187 | PHYHD1 |
| Model-25 | 33200655 | Huang W | 2020 | mRNA | 0.121054 | PLCXD3 |
| Model-25 | 33200655 | Huang W | 2020 | mRNA | 0.216349 | PLIN4 |
| Model-25 | 33200655 | Huang W | 2020 | mRNA | 0.063304 | PRKAR2B |
| Model-25 | 33200655 | Huang W | 2020 | mRNA | 0.069267 | PRPH |
| Model-25 | 33200655 | Huang W | 2020 | mRNA | 0.034229 | SFN |
| Model-25 | 33200655 | Huang W | 2020 | mRNA | 0.077424 | SRPX |
| Model-26 | 35813819 | Zhao Y | 2022 | mRNA | -0.0618 | PCNA |
| Model-26 | 35813819 | Zhao Y | 2022 | mRNA | 0.2175 | XRCC5 |
| Model-26 | 35813819 | Zhao Y | 2022 | mRNA | 0.1094 | XRCC6 |
| Model-26 | 35813819 | Zhao Y | 2022 | mRNA | -0.0929 | RFC3 |
| Model-26 | 35813819 | Zhao Y | 2022 | mRNA | 0.1894 | FANCL |
| Model-26 | 35813819 | Zhao Y | 2022 | mRNA | -0.0008 | NEIL1 |
| Model-26 | 35813819 | Zhao Y | 2022 | mRNA | 0.0095 | NEIL3 |
| Model-26 | 35813819 | Zhao Y | 2022 | mRNA | 0.1651 | NBN |
| Model-26 | 35813819 | Zhao Y | 2022 | mRNA | 0.1594 | ERCC1 |
| Model-26 | 35813819 | Zhao Y | 2022 | mRNA | -0.0817 | REV3L |
| Model-26 | 35813819 | Zhao Y | 2022 | mRNA | -0.064 | REV1 |
| Model-26 | 35813819 | Zhao Y | 2022 | mRNA | -0.0119 | HFM1 |
| Model-26 | 35813819 | Zhao Y | 2022 | mRNA | 0.16 | DDB1 |
| Model-26 | 35813819 | Zhao Y | 2022 | mRNA | 0.107 | EXO1 |
| Model-26 | 35813819 | Zhao Y | 2022 | mRNA | 0.2896 | RAD23B |
| Model-26 | 35813819 | Zhao Y | 2022 | mRNA | 0.0548 | POLD2 |
| Model-27 | 36193203 | Liu M | 2022 | mRNA | 0.456 | LDHA |
| Model-27 | 36193203 | Liu M | 2022 | mRNA | 0.278 | EIF3B |
| Model-27 | 36193203 | Liu M | 2022 | mRNA | 0.09 | TNS4 |
| Model-27 | 36193203 | Liu M | 2022 | mRNA | 0.08 | LY6K |
| Model-27 | 36193203 | Liu M | 2022 | mRNA | -0.385 | PDIK1L |
| Model-28 | 34869771 | Zhang G | 2021 | mRNA | -0.057 | NLRP1 |
| Model-28 | 34869771 | Zhang G | 2021 | mRNA | -0.139 | NLRC4 |
| Model-28 | 34869771 | Zhang G | 2021 | mRNA | -0.054 | NOD1 |
| Model-28 | 34869771 | Zhang G | 2021 | mRNA | 0.034 | CASP9 |
| Model-28 | 34869771 | Zhang G | 2021 | mRNA | -0.015 | PLCG1 |
| Model-29 | 33344071 | Cao Y | 2020 | mRNA | 0.00005 | PFKP |
| Model-29 | 33344071 | Cao Y | 2020 | mRNA | 0.00173 | PKM |
| Model-29 | 33344071 | Cao Y | 2020 | mRNA | 0.00038 | TPI1 |
| Model-29 | 33344071 | Cao Y | 2020 | mRNA | 0.00379 | LDHA |
| Model-29 | 33344071 | Cao Y | 2020 | mRNA | 0.00292 | PTGES |
| Model-29 | 33344071 | Cao Y | 2020 | mRNA | 0.0249 | TYMS |
| Model-30 | 35205284 | Wu Y | 2022 | mRNA | -0.034 | HLF |
| Model-30 | 35205284 | Wu Y | 2022 | mRNA | -0.06167 | CHRDL1 |
| Model-30 | 35205284 | Wu Y | 2022 | mRNA | -0.16551 | SELENBP1 |
| Model-30 | 35205284 | Wu Y | 2022 | mRNA | -0.01203 | TMEM163 |
| Model-31 | 31198635 | Songyang Y | 2019 | mRNA | -0.14 | ABAT |
| Model-31 | 31198635 | Songyang Y | 2019 | mRNA | 0.08 | BCAR3 |
| Model-31 | 31198635 | Songyang Y | 2019 | mRNA | -0.06 | CTSF |
| Model-31 | 31198635 | Songyang Y | 2019 | mRNA | -0.09 | DEAF1 |
| Model-31 | 31198635 | Songyang Y | 2019 | mRNA | -0.12 | ENC1 |
| Model-31 | 31198635 | Songyang Y | 2019 | mRNA | -0.09 | ETV5 |
| Model-31 | 31198635 | Songyang Y | 2019 | mRNA | 0.08 | FAM117A |
| Model-31 | 31198635 | Songyang Y | 2019 | mRNA | -0.12 | FZD2 |
| Model-31 | 31198635 | Songyang Y | 2019 | mRNA | 0.19 | GALNT12 |
| Model-31 | 31198635 | Songyang Y | 2019 | mRNA | 0.02 | GALNT3 |
| Model-31 | 31198635 | Songyang Y | 2019 | mRNA | 0.04 | GJB3 |
| Model-31 | 31198635 | Songyang Y | 2019 | mRNA | -0.14 | KDM6A |
| Model-31 | 31198635 | Songyang Y | 2019 | mRNA | 0.07 | KYNU |
| Model-31 | 31198635 | Songyang Y | 2019 | mRNA | 0.06 | PCNA |
| Model-31 | 31198635 | Songyang Y | 2019 | mRNA | 0.05 | PFKP |
| Model-31 | 31198635 | Songyang Y | 2019 | mRNA | 0.1 | PLEK2 |
| Model-31 | 31198635 | Songyang Y | 2019 | mRNA | -0.05 | RASGRP2 |
| Model-31 | 31198635 | Songyang Y | 2019 | mRNA | -0.08 | SERPIND1 |
| Model-31 | 31198635 | Songyang Y | 2019 | mRNA | -0.06 | SGSH |
| Model-31 | 31198635 | Songyang Y | 2019 | mRNA | 0.05 | TLE1 |
| Model-31 | 31198635 | Songyang Y | 2019 | mRNA | 0.07 | TMEM38B |
| Model-31 | 31198635 | Songyang Y | 2019 | mRNA | -0.08 | TMEM57 |
| Model-31 | 31198635 | Songyang Y | 2019 | mRNA | 0.16 | TRIM45 |
| Model-31 | 31198635 | Songyang Y | 2019 | mRNA | -0.07 | USP47 |
| Model-31 | 31198635 | Songyang Y | 2019 | mRNA | -0.06 | VWA1 |
| Model-32 | 35946526 | Wu L | 2022 | mRNA | -0.26524 | BTK |
| Model-32 | 35946526 | Wu L | 2022 | mRNA | -0.13769 | FCN1 |
| Model-32 | 35946526 | Wu L | 2022 | mRNA | -0.07342 | MS4A7 |
| Model-32 | 35946526 | Wu L | 2022 | mRNA | -0.03788 | HLA-DQA1 |
| Model-32 | 35946526 | Wu L | 2022 | mRNA | -0.02093 | LTF |
| Model-32 | 35946526 | Wu L | 2022 | mRNA | 0.02685 | TESC |
| Model-32 | 35946526 | Wu L | 2022 | mRNA | 0.08172 | S100P |
| Model-32 | 35946526 | Wu L | 2022 | mRNA | 0.29365 | LAIR1 |
| Model-33 | 37189138 | Wang Y | 2023 | mRNA | 0.3207 | HSPD1 |
| Model-33 | 37189138 | Wang Y | 2023 | mRNA | 0.124 | COL1A1 |
| Model-33 | 37189138 | Wang Y | 2023 | mRNA | 0.233 | PCSK9 |
| Model-33 | 37189138 | Wang Y | 2023 | mRNA | -0.159 | MAOB |
| Model-33 | 37189138 | Wang Y | 2023 | mRNA | -0.822 | GRIA1 |
| Model-33 | 37189138 | Wang Y | 2023 | mRNA | 0.251 | CAV1 |
| Model-34 | 37197625 | Ling X | 2023 | mRNA | 0.1371 | RPE |
| Model-34 | 37197625 | Ling X | 2023 | mRNA | -0.1338 | CLEC3B |
| Model-34 | 37197625 | Ling X | 2023 | mRNA | -0.0873 | METTL7A |
| Model-34 | 37197625 | Ling X | 2023 | mRNA | 0.2231 | PTGES3 |
| Model-34 | 37197625 | Ling X | 2023 | mRNA | -0.2774 | NMUR1 |
| Model-34 | 37197625 | Ling X | 2023 | mRNA | -0.007 | C1QTNF7 |
| Model-34 | 37197625 | Ling X | 2023 | mRNA | -0.0162 | DNAAF9 |
| Model-35 | 33256552 | Tian WJ | 2020 | mRNA | 0.001608 | S100A16 |
| Model-35 | 33256552 | Tian WJ | 2020 | mRNA | 0.004246 | CRABP1 |
| Model-35 | 33256552 | Tian WJ | 2020 | mRNA | 0.063071 | RBP2 |
| Model-35 | 33256552 | Tian WJ | 2020 | mRNA | 0.294875 | FGF2 |
| Model-35 | 33256552 | Tian WJ | 2020 | mRNA | -0.06332 | BTK |
| Model-35 | 33256552 | Tian WJ | 2020 | mRNA | 0.005656 | SEMA4B |
| Model-35 | 33256552 | Tian WJ | 2020 | mRNA | 0.12218 | IL11 |
| Model-35 | 33256552 | Tian WJ | 2020 | mRNA | 0.006085 | INHA |
| Model-35 | 33256552 | Tian WJ | 2020 | mRNA | 0.004722 | ANGPTL4 |
| Model-35 | 33256552 | Tian WJ | 2020 | mRNA | 0.013986 | LGR4 |
| Model-35 | 33256552 | Tian WJ | 2020 | mRNA | 0.215939 | TNFRSF11A |
| Model-35 | 33256552 | Tian WJ | 2020 | mRNA | -0.10309 | VIPR1 |
| Model-35 | 33256552 | Tian WJ | 2020 | mRNA | -0.15857 | SHC3 |
| Model-36 | 34012732 | Lin J | 2021 | mRNA | -0.7305 | DNAJC27 |
| Model-36 | 34012732 | Lin J | 2021 | mRNA | 0.4776 | NPAS2 |
| Model-36 | 34012732 | Lin J | 2021 | mRNA | 0.3941 | PHKA1 |
| Model-36 | 34012732 | Lin J | 2021 | mRNA | -0.5537 | CDADC1 |
| Model-36 | 34012732 | Lin J | 2021 | mRNA | 0.4792 | PTGFRN |
| Model-36 | 34012732 | Lin J | 2021 | mRNA | 0.398 | DDIT4 |
| Model-36 | 34012732 | Lin J | 2021 | mRNA | -0.4133 | SCAMP5 |
| Model-36 | 34012732 | Lin J | 2021 | mRNA | -0.4367 | SPRY2 |
| Model-36 | 34012732 | Lin J | 2021 | mRNA | 0.385 | CSE1L |
| Model-36 | 34012732 | Lin J | 2021 | mRNA | -0.394 | ELAVL4 |
| Model-36 | 34012732 | Lin J | 2021 | mRNA | 0.3293 | TRIM29 |
| Model-36 | 34012732 | Lin J | 2021 | mRNA | 0.3685 | LPGAT1 |
| Model-36 | 34012732 | Lin J | 2021 | mRNA | -0.3279 | TRPC3 |
| Model-36 | 34012732 | Lin J | 2021 | mRNA | 0.3257 | DCBLD2 |
| Model-37 | 36983658 | Huang Y | 2023 | mRNA | 0.113 | F2 |
| Model-37 | 36983658 | Huang Y | 2023 | mRNA | -0.178 | GPX3 |
| Model-37 | 36983658 | Huang Y | 2023 | mRNA | 0.143 | BCL2L10 |
| Model-37 | 36983658 | Huang Y | 2023 | mRNA | 0.079 | KRT18 |
| Model-37 | 36983658 | Huang Y | 2023 | mRNA | 0.007 | GCLC |
| Model-37 | 36983658 | Huang Y | 2023 | mRNA | -0.06 | BTG2 |
| Model-37 | 36983658 | Huang Y | 2023 | mRNA | 0.013 | SLC7A11 |
| Model-37 | 36983658 | Huang Y | 2023 | mRNA | 0.113 | ITGB4 |
| Model-37 | 36983658 | Huang Y | 2023 | mRNA | 0.406 | GAPDH |
| Model-37 | 36983658 | Huang Y | 2023 | mRNA | -0.01 | BIRC5 |
| Model-38 | 37100923 | Yi Y | 2023 | mRNA | 0.330023 | FAM83A-AS1 |
| Model-38 | 37100923 | Yi Y | 2023 | mRNA | 0.550431 | LDHA |
| Model-38 | 37100923 | Yi Y | 2023 | mRNA | 0.541161 | GNPNAT1 |
| Model-38 | 37100923 | Yi Y | 2023 | mRNA | 0.33146 | MELTF |
| Model-38 | 37100923 | Yi Y | 2023 | mRNA | 0.215111 | GPR37 |
| Model-38 | 37100923 | Yi Y | 2023 | mRNA | 0.279902 | IGF2BP1 |
| Model-38 | 37100923 | Yi Y | 2023 | mRNA | -0.4684 | AZIN2 |
| Model-38 | 37100923 | Yi Y | 2023 | mRNA | 0.227932 | FSCN1 |
| Model-39 | 36263421 | Li Y | 2022 | mRNA | 0.065146 | S100P |
| Model-39 | 36263421 | Li Y | 2022 | mRNA | -0.27105 | ARRB1 |
| Model-39 | 36263421 | Li Y | 2022 | mRNA | 0.104952 | S100A16 |
| Model-39 | 36263421 | Li Y | 2022 | mRNA | 0.152009 | PGK1 |
| Model-39 | 36263421 | Li Y | 2022 | mRNA | 0.088969 | TNFSF11 |
| Model-39 | 36263421 | Li Y | 2022 | mRNA | -0.17473 | NCR3 |
| Model-39 | 36263421 | Li Y | 2022 | mRNA | -0.05673 | TSLP |
| Model-40 | 36506331 | Wang Z | 2022 | mRNA | 0.118495 | ALDOA |
| Model-40 | 36506331 | Wang Z | 2022 | mRNA | -0.00361 | CASP12 |
| Model-40 | 36506331 | Wang Z | 2022 | mRNA | 0.011037 | CASP14 |
| Model-40 | 36506331 | Wang Z | 2022 | mRNA | -0.03671 | EXO1 |
| Model-40 | 36506331 | Wang Z | 2022 | mRNA | 0.074027 | FAT1 |
| Model-40 | 36506331 | Wang Z | 2022 | mRNA | 0.058166 | FEN1 |
| Model-40 | 36506331 | Wang Z | 2022 | mRNA | -0.04225 | GDF15 |
| Model-40 | 36506331 | Wang Z | 2022 | mRNA | 0.061475 | HOXB7 |
| Model-40 | 36506331 | Wang Z | 2022 | mRNA | 0.105367 | KRT18 |
| Model-40 | 36506331 | Wang Z | 2022 | mRNA | -0.10004 | MAL |
| Model-40 | 36506331 | Wang Z | 2022 | mRNA | 0.134121 | MSX1 |
| Model-40 | 36506331 | Wang Z | 2022 | mRNA | 0.099919 | NT5E |
| Model-40 | 36506331 | Wang Z | 2022 | mRNA | 0.014771 | NTSR1 |
| Model-40 | 36506331 | Wang Z | 2022 | mRNA | -0.11253 | SIX1 |
| Model-40 | 36506331 | Wang Z | 2022 | mRNA | 0.024867 | TXNRD1 |
| Model-40 | 36506331 | Wang Z | 2022 | mRNA | 0.042712 | UBE2S |
| Model-40 | 36506331 | Wang Z | 2022 | mRNA | -0.04043 | WFDC2 |
| Model-41 | 36975430 | Guo Z | 2023 | mRNA | -0.0652 | CACNA2D2 |
| Model-41 | 36975430 | Guo Z | 2023 | mRNA | -0.1039 | CYP2B7P |
| Model-41 | 36975430 | Guo Z | 2023 | mRNA | 0.0704 | KRT6A |
| Model-42 | 35903696 | Zhang Z | 2022 | mRNA | 0.129 | HMGA2 |
| Model-42 | 35903696 | Zhang Z | 2022 | mRNA | 0.044 | RHOV |
| Model-42 | 35903696 | Zhang Z | 2022 | mRNA | 0.003 | PRSS3 |
| Model-42 | 35903696 | Zhang Z | 2022 | mRNA | 0.025 | KCNF1 |
| Model-42 | 35903696 | Zhang Z | 2022 | mRNA | 0.112 | FAM83A |
| Model-42 | 35903696 | Zhang Z | 2022 | mRNA | 0.021 | CDH17 |
| Model-42 | 35903696 | Zhang Z | 2022 | mRNA | 0.131 | IGFBP1 |
| Model-42 | 35903696 | Zhang Z | 2022 | mRNA | -0.071 | ZNF493 |
| Model-42 | 35903696 | Zhang Z | 2022 | mRNA | -0.117 | P2RY13 |
| Model-42 | 35903696 | Zhang Z | 2022 | mRNA | 0.006 | IGF2BP1 |
| Model-42 | 35903696 | Zhang Z | 2022 | mRNA | 0.181 | FBN2 |
| Model-42 | 35903696 | Zhang Z | 2022 | mRNA | 0.007 | ABCC2 |
| Model-42 | 35903696 | Zhang Z | 2022 | mRNA | 0.06 | DNER |
| Model-42 | 35903696 | Zhang Z | 2022 | mRNA | 0.064 | MAPK4 |
| Model-42 | 35903696 | Zhang Z | 2022 | mRNA | 0.126 | HOXD8 |
| Model-42 | 35903696 | Zhang Z | 2022 | mRNA | 0.092 | KYNU |
| Model-43 | 36774456 | Wang G | 2023 | mRNA | 0.1231 | GJB3 |
| Model-43 | 36774456 | Wang G | 2023 | mRNA | 0.0268 | KIAA0319 |
| Model-43 | 36774456 | Wang G | 2023 | mRNA | -0.0606 | ZNF493 |
| Model-43 | 36774456 | Wang G | 2023 | mRNA | 0.0125 | KCNF1 |
| Model-43 | 36774456 | Wang G | 2023 | mRNA | -0.0869 | MS4A1 |
| Model-43 | 36774456 | Wang G | 2023 | mRNA | 0.1068 | FLNC |
| Model-43 | 36774456 | Wang G | 2023 | mRNA | 0.0202 | FSCN1 |
| Model-43 | 36774456 | Wang G | 2023 | mRNA | 0.055 | ANGPTL4 |
| Model-43 | 36774456 | Wang G | 2023 | mRNA | 0.0234 | AHNAK2 |
| Model-43 | 36774456 | Wang G | 2023 | mRNA | -0.0025 | ELF5 |
| Model-44 | 36186418 | Li F | 2022 | mRNA | -0.282 | DCN |
| Model-44 | 36186418 | Li F | 2022 | mRNA | 0.105 | LOXL2 |
| Model-44 | 36186418 | Li F | 2022 | mRNA | 0.041 | MMP14 |
| Model-44 | 36186418 | Li F | 2022 | mRNA | 0.071 | PLOD2 |
| Model-44 | 36186418 | Li F | 2022 | mRNA | 0.149 | PMEPA1 |
| Model-44 | 36186418 | Li F | 2022 | mRNA | 0.03 | SPOCK1 |
| Model-45 | 35731050 | Lin GY | 2022 | mRNA | -0.148 | MS4A1 |
| Model-45 | 35731050 | Lin GY | 2022 | mRNA | -0.148 | CPA3 |
| Model-45 | 35731050 | Lin GY | 2022 | mRNA | 0.173 | FSCN1 |
| Model-45 | 35731050 | Lin GY | 2022 | mRNA | 0.184 | PTPRH |
| Model-45 | 35731050 | Lin GY | 2022 | mRNA | 0.11 | DKK1 |
| Model-46 | 33195688 | Zhang Y | 2020 | mRNA | 0.000245 | NTSR1 |
| Model-46 | 33195688 | Zhang Y | 2020 | mRNA | 7.13E-05 | RHOV |
| Model-46 | 33195688 | Zhang Y | 2020 | mRNA | 0.000505 | KLK8 |
| Model-46 | 33195688 | Zhang Y | 2020 | mRNA | 7.01E-05 | TNS4 |
| Model-46 | 33195688 | Zhang Y | 2020 | mRNA | 0.000288 | C1QTNF6 |
| Model-46 | 33195688 | Zhang Y | 2020 | mRNA | 0.00044 | IVL |
| Model-46 | 33195688 | Zhang Y | 2020 | mRNA | 0.000161 | B4GALNT2 |
| Model-47 | 36387469 | Hu Y | 2022 | mRNA | -0.06627 | CXCL13 |
| Model-47 | 36387469 | Hu Y | 2022 | mRNA | 0.118971 | CCL20 |
| Model-47 | 36387469 | Hu Y | 2022 | mRNA | -0.19912 | CX3CR1 |
| Model-47 | 36387469 | Hu Y | 2022 | mRNA | -0.2435 | CXCL4L1 |
| Model-48 | 35968341 | Li L | 2022 | mRNA | 0.083 | IL1A |
| Model-48 | 35968341 | Li L | 2022 | mRNA | 0.152 | CLDN1 |
| Model-48 | 35968341 | Li L | 2022 | mRNA | 0.051 | ANLN |
| Model-48 | 35968341 | Li L | 2022 | mRNA | 0.036 | PKIB |
| Model-48 | 35968341 | Li L | 2022 | mRNA | 0.006 | GJB3 |
| Model-48 | 35968341 | Li L | 2022 | mRNA | 0.041 | MUC16 |
| Model-48 | 35968341 | Li L | 2022 | mRNA | 0.004 | TNS4 |
| Model-48 | 35968341 | Li L | 2022 | mRNA | 0.028 | DKK1 |
| Model-48 | 35968341 | Li L | 2022 | mRNA | 0.095 | CPS1 |
| Model-48 | 35968341 | Li L | 2022 | mRNA | -0.084 | HAS3 |
| Model-48 | 35968341 | Li L | 2022 | mRNA | -0.022 | CYP4B1 |
| Model-49 | 36225197 | Tang M | 2022 | mRNA | 0.034772 | CYP27C1 |
| Model-49 | 36225197 | Tang M | 2022 | mRNA | 0.025435 | CYP24A1 |
| Model-49 | 36225197 | Tang M | 2022 | mRNA | -0.03872 | CYP4B1 |
| Model-49 | 36225197 | Tang M | 2022 | mRNA | 0.378092 | FGF2 |
| Model-49 | 36225197 | Tang M | 2022 | mRNA | 0.063389 | SLC2A1 |
| Model-49 | 36225197 | Tang M | 2022 | mRNA | 0.030467 | CDKN3 |
| Model-49 | 36225197 | Tang M | 2022 | mRNA | 0.193865 | KRT8 |
| Model-49 | 36225197 | Tang M | 2022 | mRNA | 0.027231 | CCNB1 |
| Model-49 | 36225197 | Tang M | 2022 | mRNA | -0.06926 | OLR1 |
| Model-49 | 36225197 | Tang M | 2022 | mRNA | 0.026932 | MKI67 |
| Model-49 | 36225197 | Tang M | 2022 | mRNA | 0.002432 | FA2H |
| Model-49 | 36225197 | Tang M | 2022 | mRNA | -0.07869 | ADRB2 |
| Model-50 | 35372503 | Nai A | 2022 | mRNA | 0.151495 | MMP14 |
| Model-50 | 35372503 | Nai A | 2022 | mRNA | -0.13432 | BTG2 |
| Model-50 | 35372503 | Nai A | 2022 | mRNA | 0.10373 | LAMP3 |
| Model-50 | 35372503 | Nai A | 2022 | mRNA | 0.131301 | CCL20 |
| Model-50 | 35372503 | Nai A | 2022 | mRNA | -0.20083 | TLR2 |
| Model-50 | 35372503 | Nai A | 2022 | mRNA | -0.20673 | IL7R |
| Model-50 | 35372503 | Nai A | 2022 | mRNA | 0.241538 | PCDH7 |
| Model-51 | 35102130 | Wang Y | 2022 | mRNA | 0.158 | RELA |
| Model-51 | 35102130 | Wang Y | 2022 | mRNA | 0.146 | ACSL3 |
| Model-51 | 35102130 | Wang Y | 2022 | mRNA | 0.116 | RRM2 |
| Model-51 | 35102130 | Wang Y | 2022 | mRNA | 0.113 | ALOX12B |
| Model-51 | 35102130 | Wang Y | 2022 | mRNA | 0.078 | CISD1 |
| Model-51 | 35102130 | Wang Y | 2022 | mRNA | 0.068 | EIF2S1 |
| Model-51 | 35102130 | Wang Y | 2022 | mRNA | 0.002 | SLC2A1 |
| Model-51 | 35102130 | Wang Y | 2022 | mRNA | -0.09 | GDF15 |
| Model-51 | 35102130 | Wang Y | 2022 | mRNA | -0.067 | PHKG2 |
| Model-51 | 35102130 | Wang Y | 2022 | mRNA | -0.075 | SLC1A4 |
| Model-51 | 35102130 | Wang Y | 2022 | mRNA | -0.086 | TLR4 |
| Model-51 | 35102130 | Wang Y | 2022 | mRNA | -0.114 | FLT3 |
| Model-51 | 35102130 | Wang Y | 2022 | mRNA | -0.168 | TUBE1 |
| Model-51 | 35102130 | Wang Y | 2022 | mRNA | -0.21 | GLS2 |
| Model-51 | 35102130 | Wang Y | 2022 | mRNA | -0.214 | PEBP1 |
| Model-52 | 34631307 | Liu L | 2021 | mRNA | -0.28593 | TRAF3IP3 |
| Model-52 | 34631307 | Liu L | 2021 | mRNA | 0.21193 | PITX3 |
| Model-52 | 34631307 | Liu L | 2021 | mRNA | -0.47968 | MYLIP |
| Model-52 | 34631307 | Liu L | 2021 | mRNA | 0.26692 | CENPH |
| Model-53 | 35280857 | Feng H | 2022 | mRNA | -0.03235 | IL3RA |
| Model-53 | 35280857 | Feng H | 2022 | mRNA | -0.03132 | MAGEH1 |
| Model-53 | 35280857 | Feng H | 2022 | mRNA | -0.25855 | CCR2 |
| Model-53 | 35280857 | Feng H | 2022 | mRNA | -0.00105 | ACP5 |
| Model-53 | 35280857 | Feng H | 2022 | mRNA | 0.002917 | TGFBI |
| Model-53 | 35280857 | Feng H | 2022 | mRNA | -0.47341 | CHRNA6 |
| Model-53 | 35280857 | Feng H | 2022 | mRNA | -0.06609 | KSR1 |
| Model-53 | 35280857 | Feng H | 2022 | mRNA | 0.02076 | GIMAP6 |
| Model-53 | 35280857 | Feng H | 2022 | mRNA | -0.18895 | CD80 |
| Model-53 | 35280857 | Feng H | 2022 | mRNA | 0.128343 | IL16 |
| Model-53 | 35280857 | Feng H | 2022 | mRNA | -0.00022 | CD52 |
| Model-54 | 36212131 | Huang S | 2022 | mRNA | 0.3486 | E2F7 |
| Model-54 | 36212131 | Huang S | 2022 | mRNA | 0.3011 | EIF3H |
| Model-54 | 36212131 | Huang S | 2022 | mRNA | 0.2536 | INTS1 |
| Model-54 | 36212131 | Huang S | 2022 | mRNA | 0.3019 | LPGAT1 |
| Model-54 | 36212131 | Huang S | 2022 | mRNA | 0.3078 | MCAT |
| Model-54 | 36212131 | Huang S | 2022 | mRNA | 0.3006 | SEC61G |
| Model-54 | 36212131 | Huang S | 2022 | mRNA | -0.6044 | SS18L1 |
| Model-54 | 36212131 | Huang S | 2022 | mRNA | -0.2837 | TNYN1 |
| Model-55 | 36263018 | Ye Y | 2022 | mRNA | 0.125 | SMNDC1 |
| Model-55 | 36263018 | Ye Y | 2022 | mRNA | 0.107 | CBX5 |
| Model-55 | 36263018 | Ye Y | 2022 | mRNA | -0.172 | SETDB2 |
| Model-55 | 36263018 | Ye Y | 2022 | mRNA | 0.073 | PHF14 |
| Model-55 | 36263018 | Ye Y | 2022 | mRNA | 0.086 | SGF29 |
| Model-55 | 36263018 | Ye Y | 2022 | mRNA | -0.051 | PRDM16 |
| Model-55 | 36263018 | Ye Y | 2022 | mRNA | -0.03 | ZCWPW2 |
| Model-55 | 36263018 | Ye Y | 2022 | mRNA | 0.016 | PHF19 |
| Model-56 | 33072571 | Qi X | 2020 | mRNA | -0.13549 | CLEC17A |
| Model-56 | 33072571 | Qi X | 2020 | mRNA | 0.012072 | INHA |
| Model-56 | 33072571 | Qi X | 2020 | mRNA | 0.62635 | XIRP1 |
| Model-57 | 36845403 | Zhu X | 2023 | mRNA | 0.1021 | ACAN |
| Model-57 | 36845403 | Zhu X | 2023 | mRNA | 0.0162 | ADAMTS15 |
| Model-57 | 36845403 | Zhu X | 2023 | mRNA | -0.01 | ADAMTS8 |
| Model-57 | 36845403 | Zhu X | 2023 | mRNA | 0.0058 | BCAN |
| Model-57 | 36845403 | Zhu X | 2023 | mRNA | -0.007 | COL4A3 |
| Model-57 | 36845403 | Zhu X | 2023 | mRNA | -0.0405 | ITGA8 |
| Model-57 | 36845403 | Zhu X | 2023 | mRNA | 0.0017 | ITGB4 |
| Model-57 | 36845403 | Zhu X | 2023 | mRNA | 0.0043 | LAD1 |
| Model-57 | 36845403 | Zhu X | 2023 | mRNA | -0.0891 | TENM3 |
| Model-57 | 36845403 | Zhu X | 2023 | mRNA | 0.0003 | TIMP1 |
| Model-58 | 35308531 | Zhao Y | 2022 | mRNA | 0.102 | TERT |
| Model-58 | 35308531 | Zhao Y | 2022 | mRNA | 0.012 | PTTG1 |
| Model-58 | 35308531 | Zhao Y | 2022 | mRNA | 0.123 | SMUG1 |
| Model-58 | 35308531 | Zhao Y | 2022 | mRNA | 0.005 | PRKDC |
| Model-58 | 35308531 | Zhao Y | 2022 | mRNA | 0.002 | H2AFX |
| Model-58 | 35308531 | Zhao Y | 2022 | mRNA | 0.003 | PFKP |
| Model-58 | 35308531 | Zhao Y | 2022 | mRNA | 0.0006 | TXNRD1 |
| Model-58 | 35308531 | Zhao Y | 2022 | mRNA | -0.003 | CAT |
| Model-59 | 34416861 | Zhou H | 2021 | mRNA | -0.02948 | CLEC3B |
| Model-59 | 34416861 | Zhou H | 2021 | mRNA | 0.000591 | TENM3 |
| Model-59 | 34416861 | Zhou H | 2021 | mRNA | 0.020545 | IGF2BP1 |
| Model-59 | 34416861 | Zhou H | 2021 | mRNA | 0.168144 | E2F7 |
| Model-59 | 34416861 | Zhou H | 2021 | mRNA | 0.008597 | ANLN |
| Model-59 | 34416861 | Zhou H | 2021 | mRNA | 0.283523 | ANKRD18B |
| Model-59 | 34416861 | Zhou H | 2021 | mRNA | 0.017282 | FBN2 |
| Model-60 | 36793937 | Yu J | 2023 | mRNA | 0.0365 | ANGPTL4 |
| Model-60 | 36793937 | Yu J | 2023 | mRNA | 0.0158 | BARX2 |
| Model-60 | 36793937 | Yu J | 2023 | mRNA | -0.0131 | GPR98 |
| Model-60 | 36793937 | Yu J | 2023 | mRNA | 0.0232 | KRT6A |
| Model-60 | 36793937 | Yu J | 2023 | mRNA | 0.024 | PTPRH |
| Model-60 | 36793937 | Yu J | 2023 | mRNA | 0.0852 | RGS20 |
| Model-60 | 36793937 | Yu J | 2023 | mRNA | 0.0124 | TCN1 |
| Model-60 | 36793937 | Yu J | 2023 | mRNA | 0.0211 | TNS4 |
| Model-61 | 36728032 | Guo Z | 2023 | mRNA | 0.016 | S100P |
| Model-61 | 36728032 | Guo Z | 2023 | mRNA | 0.024 | INHA |
| Model-61 | 36728032 | Guo Z | 2023 | mRNA | 0.29 | SEMA7A |
| Model-61 | 36728032 | Guo Z | 2023 | mRNA | 0.082 | INSL4 |
| Model-61 | 36728032 | Guo Z | 2023 | mRNA | -0.051 | CD40LG |
| Model-61 | 36728032 | Guo Z | 2023 | mRNA | -0.033 | AGER |
| Model-61 | 36728032 | Guo Z | 2023 | mRNA | -0.029 | SERPIND1 |
| Model-61 | 36728032 | Guo Z | 2023 | mRNA | -0.042 | CD1D |
| Model-61 | 36728032 | Guo Z | 2023 | mRNA | -0.044 | CX3CR1 |
| Model-61 | 36728032 | Guo Z | 2023 | mRNA | -0.026 | SFTPD |
| Model-61 | 36728032 | Guo Z | 2023 | mRNA | -0.115 | CD79A |
| Model-62 | 35664356 | Li YP | 2022 | mRNA | 0.089163 | SERPINB5 |
| Model-62 | 35664356 | Li YP | 2022 | mRNA | 0.133421 | CCNB1 |
| Model-62 | 35664356 | Li YP | 2022 | mRNA | -0.05839 | FGR |
| Model-62 | 35664356 | Li YP | 2022 | mRNA | -0.09278 | MAOB |
| Model-62 | 35664356 | Li YP | 2022 | mRNA | -0.04419 | SH3BP5 |
| Model-62 | 35664356 | Li YP | 2022 | mRNA | 0.038967 | CYP24A1 |
| Model-63 | 36505472 | Huang H | 2022 | mRNA | 0.077 | GREM1 |
| Model-63 | 36505472 | Huang H | 2022 | mRNA | 0.068 | GJB2 |
| Model-63 | 36505472 | Huang H | 2022 | mRNA | -0.151 | CCR2 |
| Model-63 | 36505472 | Huang H | 2022 | mRNA | 0.002 | MMP1 |
| Model-63 | 36505472 | Huang H | 2022 | mRNA | -0.006 | IL7R |
| Model-63 | 36505472 | Huang H | 2022 | mRNA | -0.062 | MS4A1 |
| Model-63 | 36505472 | Huang H | 2022 | mRNA | -0.042 | HLA-DQB2 |
| Model-64 | 36506303 | Li Y | 2022 | mRNA | -0.00449 | AGER |
| Model-64 | 36506303 | Li Y | 2022 | mRNA | -0.07749 | CYP27A1 |
| Model-64 | 36506303 | Li Y | 2022 | mRNA | 0.113176 | CDK1 |
| Model-64 | 36506303 | Li Y | 2022 | mRNA | -0.04531 | CADM1 |
| Model-64 | 36506303 | Li Y | 2022 | mRNA | 0.342269 | FADD |
| Model-64 | 36506303 | Li Y | 2022 | mRNA | 0.133394 | ADA |
| Model-64 | 36506303 | Li Y | 2022 | mRNA | 0.024215 | LTBR |
| Model-64 | 36506303 | Li Y | 2022 | mRNA | -0.02296 | FYN |
| Model-64 | 36506303 | Li Y | 2022 | mRNA | -0.1999 | CRTAM |
| Model-65 | 36351297 | Zhang J | 2023 | mRNA | -0.12783 | CD79A |
| Model-65 | 36351297 | Zhang J | 2023 | mRNA | -0.10536 | KIAA1324 |
| Model-65 | 36351297 | Zhang J | 2023 | mRNA | 0.29267 | PLSCR1 |
| Model-65 | 36351297 | Zhang J | 2023 | mRNA | 0.207014 | CDCP1 |
| Model-65 | 36351297 | Zhang J | 2023 | mRNA | -0.19845 | CLEC7A |
| Model-65 | 36351297 | Zhang J | 2023 | mRNA | 0.182322 | ID1 |
| Model-65 | 36351297 | Zhang J | 2023 | mRNA | -0.21072 | CMTM7 |
| Model-66 | 36708655 | Zhang Z | 2023 | mRNA | 0.154153 | ACAN |
| Model-66 | 36708655 | Zhang Z | 2023 | mRNA | -0.04313 | COL4A3 |
| Model-66 | 36708655 | Zhang Z | 2023 | mRNA | -0.26455 | DCC |
| Model-66 | 36708655 | Zhang Z | 2023 | mRNA | -0.09259 | DCN |
| Model-66 | 36708655 | Zhang Z | 2023 | mRNA | 0.074093 | ITGB4 |
| Model-66 | 36708655 | Zhang Z | 2023 | mRNA | 0.006159 | SMOC1 |
| Model-66 | 36708655 | Zhang Z | 2023 | mRNA | 0.296025 | TENM3 |
| Model-66 | 36708655 | Zhang Z | 2023 | mRNA | 0.192085 | ADAMTS15 |
| Model-66 | 36708655 | Zhang Z | 2023 | mRNA | 0.252449 | BCAN |
| Model-66 | 36708655 | Zhang Z | 2023 | mRNA | -0.37678 | HMCN2 |
| Model-66 | 36708655 | Zhang Z | 2023 | mRNA | 0.048678 | ITGA2 |
| Model-66 | 36708655 | Zhang Z | 2023 | mRNA | 0.017944 | LAD1 |
| Model-66 | 36708655 | Zhang Z | 2023 | mRNA | 0.122918 | LOXL2 |
| Model-66 | 36708655 | Zhang Z | 2023 | mRNA | -0.17577 | NELL2 |
| Model-66 | 36708655 | Zhang Z | 2023 | mRNA | -0.05957 | OGN |
| Model-66 | 36708655 | Zhang Z | 2023 | mRNA | -0.02311 | SLIT3 |
| Model-66 | 36708655 | Zhang Z | 2023 | mRNA | 0.048678 | TINAG |
| Model-67 | 36467027 | Zhu Y | 2022 | mRNA | -0.168 | CYP2D6 |
| Model-67 | 36467027 | Zhu Y | 2022 | mRNA | -0.167 | FMO3 |
| Model-67 | 36467027 | Zhu Y | 2022 | mRNA | -0.059 | CAT |
| Model-67 | 36467027 | Zhu Y | 2022 | mRNA | 0.306 | GAPDH |
| Model-68 | 29928133 | Zhao K | 2018 | mRNA | 0.1375 | CERS4 |
| Model-68 | 29928133 | Zhao K | 2018 | mRNA | -0.5066 | FUT4 |
| Model-68 | 29928133 | Zhao K | 2018 | mRNA | 0.08425 | C3ORF18 |
| Model-68 | 29928133 | Zhao K | 2018 | mRNA | 0.291 | CYP17A1 |
| Model-68 | 29928133 | Zhao K | 2018 | mRNA | -0.436 | ASPM |
| Model-68 | 29928133 | Zhao K | 2018 | mRNA | 0.3888 | HJURP |
| Model-68 | 29928133 | Zhao K | 2018 | mRNA | -0.154 | LOC645166 |
| Model-68 | 29928133 | Zhao K | 2018 | mRNA | 0.3972 | DENND1C |
| Model-68 | 29928133 | Zhao K | 2018 | mRNA | -0.2404 | SLC25A42 |
| Model-68 | 29928133 | Zhao K | 2018 | mRNA | 0.1753 | CCNA2 |
| Model-68 | 29928133 | Zhao K | 2018 | mRNA | -0.06559 | LDHA |
| Model-68 | 29928133 | Zhao K | 2018 | mRNA | -0.09503 | IGFBP1 |
| Model-68 | 29928133 | Zhao K | 2018 | mRNA | 0.2518 | SLC2A1 |
| Model-68 | 29928133 | Zhao K | 2018 | mRNA | 0.02896 | DAAM2 |
| Model-68 | 29928133 | Zhao K | 2018 | mRNA | -0.03843 | RGS20 |
| Model-68 | 29928133 | Zhao K | 2018 | mRNA | -0.08505 | MFI2 |
| Model-68 | 29928133 | Zhao K | 2018 | mRNA | -0.2181 | LDLRAD3 |
| Model-68 | 29928133 | Zhao K | 2018 | mRNA | 0.1977 | KLHDC8B |
| Model-68 | 29928133 | Zhao K | 2018 | mRNA | 0.05795 | CREG2 |
| Model-68 | 29928133 | Zhao K | 2018 | mRNA | 0.10865 | SPATA6 |
| Model-69 | 35785179 | Lu F | 2022 | mRNA | 0.160674 | KIF20B |
| Model-69 | 35785179 | Lu F | 2022 | mRNA | 0.207218 | HMMR |
| Model-69 | 35785179 | Lu F | 2022 | mRNA | 0.157719 | ARNTL2 |
| Model-69 | 35785179 | Lu F | 2022 | mRNA | 0.080251 | DKK1 |
| Model-70 | 35620481 | Sun S | 2022 | mRNA | -0.00384 | CCL17 |
| Model-70 | 35620481 | Sun S | 2022 | mRNA | -0.20651 | CD40LG |
| Model-70 | 35620481 | Sun S | 2022 | mRNA | -0.09076 | CIITA |
| Model-70 | 35620481 | Sun S | 2022 | mRNA | 0.09594 | STC1 |
| Model-70 | 35620481 | Sun S | 2022 | mRNA | -0.01538 | SCGB3A1 |
| Model-70 | 35620481 | Sun S | 2022 | mRNA | -0.13689 | GDF10 |
| Model-71 | 35692818 | Zhao R | 2022 | mRNA | 0.00565 | FKBP4 |
| Model-71 | 35692818 | Zhao R | 2022 | mRNA | 0.03539 | HMMR |
| Model-71 | 35692818 | Zhao R | 2022 | mRNA | 0.00638 | B4GALT1 |
| Model-71 | 35692818 | Zhao R | 2022 | mRNA | 0.00332 | SLC2A1 |
| Model-71 | 35692818 | Zhao R | 2022 | mRNA | 0.00387 | STC1 |
| Model-72 | 35711827 | Huang H | 2022 | mRNA | 0.007277 | ADM |
| Model-72 | 35711827 | Huang H | 2022 | mRNA | -0.08605 | ARRB1 |
| Model-72 | 35711827 | Huang H | 2022 | mRNA | 0.014984 | DDIT4 |
| Model-72 | 35711827 | Huang H | 2022 | mRNA | 0.132006 | ERO1A |
| Model-72 | 35711827 | Huang H | 2022 | mRNA | 0.066597 | FURIN |
| Model-72 | 35711827 | Huang H | 2022 | mRNA | 0.181954 | LDHA |
| Model-72 | 35711827 | Huang H | 2022 | mRNA | -0.04757 | NAGK |
| Model-72 | 35711827 | Huang H | 2022 | mRNA | 0.077204 | NR2F2 |
| Model-72 | 35711827 | Huang H | 2022 | mRNA | 0.001257 | NRAS |
| Model-73 | 33850871 | Yang L | 2021 | mRNA | 0.239382 | CIRBP |
| Model-73 | 33850871 | Yang L | 2021 | mRNA | 0.24583 | DARS2 |
| Model-73 | 33850871 | Yang L | 2021 | mRNA | -0.38377 | DDX24 |
| Model-73 | 33850871 | Yang L | 2021 | mRNA | 0.30115 | GAPDH |
| Model-73 | 33850871 | Yang L | 2021 | mRNA | 0.21273 | LARP6 |
| Model-73 | 33850871 | Yang L | 2021 | mRNA | 0.37201 | SNRPE |
| Model-73 | 33850871 | Yang L | 2021 | mRNA | 0.31985 | WDR3 |
| Model-73 | 33850871 | Yang L | 2021 | mRNA | 0.41663 | ZC3H12C |
| Model-73 | 33850871 | Yang L | 2021 | mRNA | -0.41679 | ZC3H12D |
| Model-74 | 34798483 | Zhou H | 2021 | mRNA | 0.0961 | CENPH |
| Model-74 | 34798483 | Zhou H | 2021 | mRNA | 0.009 | CENPM |
| Model-74 | 34798483 | Zhou H | 2021 | mRNA | 0.0383 | CENPN |
| Model-74 | 34798483 | Zhou H | 2021 | mRNA | 0.005 | CENPU |
| Model-74 | 34798483 | Zhou H | 2021 | mRNA | -0.0129 | CENPA |
| Model-75 | 35117717 | He J | 2020 | mRNA | 0.012001 | TYMS |
| Model-75 | 35117717 | He J | 2020 | mRNA | 0.025097 | GNPNAT1 |
| Model-75 | 35117717 | He J | 2020 | mRNA | 0.010592 | LPGAT1 |
| Model-75 | 35117717 | He J | 2020 | mRNA | 0.029983 | INPP4B |
| Model-75 | 35117717 | He J | 2020 | mRNA | -0.01325 | MAOB |
| Model-75 | 35117717 | He J | 2020 | mRNA | -0.00569 | MTHFD2 |
| Model-75 | 35117717 | He J | 2020 | mRNA | -0.02076 | ADCY9 |
| Model-75 | 35117717 | He J | 2020 | mRNA | 0.008161 | PTGIS |
| Model-75 | 35117717 | He J | 2020 | mRNA | 0.110691 | ALOX12B |
| Model-75 | 35117717 | He J | 2020 | mRNA | -0.08313 | GSTA3 |
| Model-75 | 35117717 | He J | 2020 | mRNA | 0.001962 | LDHA |
| Model-75 | 35117717 | He J | 2020 | mRNA | 0.027402 | UCK2 |
| Model-75 | 35117717 | He J | 2020 | mRNA | 0.035619 | ENTPD2 |
| Model-75 | 35117717 | He J | 2020 | mRNA | 0.001614 | PTGES |
| Model-75 | 35117717 | He J | 2020 | mRNA | 0.003868 | NT5E |
| Model-75 | 35117717 | He J | 2020 | mRNA | 0.000733 | ALDOA |
| Model-76 | 35403268 | Wu Y | 2022 | mRNA | 0.11 | FAM83A |
| Model-76 | 35403268 | Wu Y | 2022 | mRNA | 0.07 | AKAP12 |
| Model-76 | 35403268 | Wu Y | 2022 | mRNA | 0.071 | PKP2 |
| Model-76 | 35403268 | Wu Y | 2022 | mRNA | -0.141 | CYP17A1 |
| Model-76 | 35403268 | Wu Y | 2022 | mRNA | -0.065 | GJB3 |
| Model-76 | 35403268 | Wu Y | 2022 | mRNA | 0.045 | TMPRSS11F |
| Model-76 | 35403268 | Wu Y | 2022 | mRNA | 0.05 | KRT81 |
| Model-76 | 35403268 | Wu Y | 2022 | mRNA | 0.109 | 44989 |
| Model-76 | 35403268 | Wu Y | 2022 | mRNA | 0.088 | STC1 |
| Model-77 | 32238163 | Wang X | 2020 | mRNA | 0.0063 | ITGB4 |
| Model-77 | 32238163 | Wang X | 2020 | mRNA | -0.354 | NLRC4 |
| Model-77 | 32238163 | Wang X | 2020 | mRNA | -0.3956 | ATG9B |
| Model-77 | 32238163 | Wang X | 2020 | mRNA | 0.0202 | CDKN2A |
| Model-77 | 32238163 | Wang X | 2020 | mRNA | 0.0122 | ERO1A |
| Model-78 | 34256900 | Zhou J | 2021 | mRNA | 0.042 | ADAM12 |
| Model-78 | 34256900 | Zhou J | 2021 | mRNA | -0.081 | CAMP |
| Model-78 | 34256900 | Zhou J | 2021 | mRNA | 0.036 | DKK1 |
| Model-78 | 34256900 | Zhou J | 2021 | mRNA | 0.027 | STRIP2 |
| Model-78 | 34256900 | Zhou J | 2021 | mRNA | 0.039 | TFAP2A |
| Model-79 | 34721973 | Liu L | 2021 | mRNA | -0.4294 | RAD54L |
| Model-79 | 34721973 | Liu L | 2021 | mRNA | -0.2411 | ZNF322 |
| Model-79 | 34721973 | Liu L | 2021 | mRNA | -0.4645 | CENPI |
| Model-79 | 34721973 | Liu L | 2021 | mRNA | 0.2242 | IGF2BP1 |
| Model-79 | 34721973 | Liu L | 2021 | mRNA | 0.2095 | IGF2BP3 |
| Model-79 | 34721973 | Liu L | 2021 | mRNA | -0.5093 | RAD51AP1 |
| Model-79 | 34721973 | Liu L | 2021 | mRNA | 0.7979 | E2F7 |
| Model-79 | 34721973 | Liu L | 2021 | mRNA | 0.7968 | HMMR |
| Model-79 | 34721973 | Liu L | 2021 | mRNA | -0.3272 | DNAJC5B |
| Model-79 | 34721973 | Liu L | 2021 | mRNA | 0.1997 | ADAMTS12 |
| Model-79 | 34721973 | Liu L | 2021 | mRNA | -0.5248 | NANOS1 |
| Model-80 | 36263430 | Wen H | 2022 | mRNA | 0.71295 | CTTNBP2NL |
| Model-80 | 36263430 | Wen H | 2022 | mRNA | -0.34249 | CYP2U1 |
| Model-80 | 36263430 | Wen H | 2022 | mRNA | -0.61619 | FAM13C |
| Model-80 | 36263430 | Wen H | 2022 | mRNA | 0.708036 | CDH23 |
| Model-80 | 36263430 | Wen H | 2022 | mRNA | 0.364643 | EXOC5 |
| Model-80 | 36263430 | Wen H | 2022 | mRNA | 0.444686 | CD300A |
| Model-80 | 36263430 | Wen H | 2022 | mRNA | 0.565314 | MRO |
| Model-80 | 36263430 | Wen H | 2022 | mRNA | -0.47804 | ARHGEF6 |
| Model-81 | 33585460 | Tang B | 2021 | mRNA | 1.1168 | RACGAP1 |
| Model-81 | 33585460 | Tang B | 2021 | mRNA | 0.474 | CDCA8 |
| Model-81 | 33585460 | Tang B | 2021 | mRNA | 1.4432 | RCC2 |
| Model-81 | 33585460 | Tang B | 2021 | mRNA | 1.7456 | PLK1 |
| Model-81 | 33585460 | Tang B | 2021 | mRNA | 1.6966 | KIF20B |
| Model-81 | 33585460 | Tang B | 2021 | mRNA | 1.1153 | ALG3 |
| Model-81 | 33585460 | Tang B | 2021 | mRNA | 0.6784 | BRCA1 |
| Model-81 | 33585460 | Tang B | 2021 | mRNA | 1.309 | CHAF1B |
| Model-82 | 37228630 | Tang B | 2023 | mRNA | 0.09935 | CCL20 |
| Model-82 | 37228630 | Tang B | 2023 | mRNA | -0.03346 | PGC |
| Model-82 | 37228630 | Tang B | 2023 | mRNA | 0.04047 | RAET1L |
| Model-82 | 37228630 | Tang B | 2023 | mRNA | -0.06405 | CD1E |
| Model-82 | 37228630 | Tang B | 2023 | mRNA | 0.1398 | FURIN |
| Model-82 | 37228630 | Tang B | 2023 | mRNA | -0.0891 | KL |
| Model-83 | 36276289 | Qi J | 2022 | mRNA | -0.2665 | LOXL2 |
| Model-83 | 36276289 | Qi J | 2022 | mRNA | 0.1905 | PTPRH |
| Model-83 | 36276289 | Qi J | 2022 | mRNA | 0.1281 | DKK1 |
| Model-83 | 36276289 | Qi J | 2022 | mRNA | 0.5798 | PKP2 |
| Model-83 | 36276289 | Qi J | 2022 | mRNA | 0.4434 | NKX2-1 |
| Model-83 | 36276289 | Qi J | 2022 | mRNA | 0.0103 | KRT6A |
| Model-84 | 36033490 | Chen B | 2022 | mRNA | -0.21567 | ARG2 |
| Model-84 | 36033490 | Chen B | 2022 | mRNA | 0.222343 | BIRC3 |
| Model-84 | 36033490 | Chen B | 2022 | mRNA | -0.57982 | BTN2A2 |
| Model-84 | 36033490 | Chen B | 2022 | mRNA | -0.48613 | CBFA2T3 |
| Model-84 | 36033490 | Chen B | 2022 | mRNA | -0.41249 | CCR2 |
| Model-84 | 36033490 | Chen B | 2022 | mRNA | -0.42159 | CD101 |
| Model-84 | 36033490 | Chen B | 2022 | mRNA | -0.81193 | CD200R1 |
| Model-84 | 36033490 | Chen B | 2022 | mRNA | 0.317726 | CDCP1 |
| Model-84 | 36033490 | Chen B | 2022 | mRNA | -0.19601 | CLEC7A |
| Model-84 | 36033490 | Chen B | 2022 | mRNA | -0.35382 | CMTM7 |
| Model-84 | 36033490 | Chen B | 2022 | mRNA | -0.5075 | FCRL6 |
| Model-84 | 36033490 | Chen B | 2022 | mRNA | 0.202124 | FURIN |
| Model-84 | 36033490 | Chen B | 2022 | mRNA | 0.24998 | L1A |
| Model-84 | 36033490 | Chen B | 2022 | mRNA | 0.286682 | KLF10 |
| Model-84 | 36033490 | Chen B | 2022 | mRNA | 0.374318 | NMI |
| Model-84 | 36033490 | Chen B | 2022 | mRNA | 0.123102 | NT5E |
| Model-84 | 36033490 | Chen B | 2022 | mRNA | 0.266203 | PARVB |
| Model-84 | 36033490 | Chen B | 2022 | mRNA | 0.172271 | PITPNC1 |
| Model-84 | 36033490 | Chen B | 2022 | mRNA | 0.231112 | PLSCR1 |
| Model-84 | 36033490 | Chen B | 2022 | mRNA | -0.23953 | SCPEP1 |
| Model-84 | 36033490 | Chen B | 2022 | mRNA | -0.21567 | SEPP1 |
| Model-84 | 36033490 | Chen B | 2022 | mRNA | 0.318454 | SLC9B2 |
| Model-84 | 36033490 | Chen B | 2022 | mRNA | 0.118672 | SMOC1 |
| Model-84 | 36033490 | Chen B | 2022 | mRNA | -0.48451 | STAP1 |
| Model-84 | 36033490 | Chen B | 2022 | mRNA | 0.223943 | UBE2E2 |
| Model-84 | 36033490 | Chen B | 2022 | mRNA | 0.225541 | VOPP1 |
| Model-85 | 37197492 | Zhu P | 2023 | mRNA | 3.041 | FLG |
| Model-85 | 37197492 | Zhu P | 2023 | mRNA | 0.41 | SLK |
| Model-85 | 37197492 | Zhu P | 2023 | mRNA | 0.656 | CFL1 |
| Model-85 | 37197492 | Zhu P | 2023 | mRNA | -0.287 | PECAM1 |
| Model-85 | 37197492 | Zhu P | 2023 | mRNA | 0.247 | ITGB1 |
| Model-86 | 34036102 | Xu Z | 2021 | mRNA | -0.12332 | BMP5 |
| Model-86 | 34036102 | Xu Z | 2021 | mRNA | 0.08118 | BMP7 |
| Model-86 | 34036102 | Xu Z | 2021 | mRNA | -0.35362 | ACVR2A |
| Model-87 | 33856140 | Liu C | 2021 | mRNA | -4.868 | ACADM |
| Model-87 | 33856140 | Liu C | 2021 | mRNA | -10.934 | RPS8 |
| Model-88 | 36324576 | Shao F | 2022 | mRNA | -0.0797 | CYP4B1 |
| Model-88 | 36324576 | Shao F | 2022 | mRNA | 0.1527 | KLF4 |
| Model-88 | 36324576 | Shao F | 2022 | mRNA | -0.1243 | DPEP2 |
| Model-88 | 36324576 | Shao F | 2022 | mRNA | -0.0165 | PTGDS |
| Model-88 | 36324576 | Shao F | 2022 | mRNA | -0.0057 | CYP27A1 |
| Model-88 | 36324576 | Shao F | 2022 | mRNA | -0.1551 | ACSS3 |
| Model-88 | 36324576 | Shao F | 2022 | mRNA | -0.0444 | HSD17B13 |
| Model-88 | 36324576 | Shao F | 2022 | mRNA | -0.0213 | HPGDS |
| Model-88 | 36324576 | Shao F | 2022 | mRNA | 0.0381 | FA2H |
| Model-89 | 35935579 | Xiong Z | 2022 | mRNA | 0.180594 | ABCD1 |
| Model-89 | 35935579 | Xiong Z | 2022 | mRNA | -0.14907 | ACAT1 |
| Model-89 | 35935579 | Xiong Z | 2022 | mRNA | -0.08015 | ACSL1 |
| Model-89 | 35935579 | Xiong Z | 2022 | mRNA | 0.26613 | ACSL3 |
| Model-89 | 35935579 | Xiong Z | 2022 | mRNA | -0.05257 | CAT |
| Model-89 | 35935579 | Xiong Z | 2022 | mRNA | 0.431596 | LDHA |
| Model-90 | 33968130 | Tu Z | 2021 | mRNA | -0.09431 | GPX3 |
| Model-90 | 33968130 | Tu Z | 2021 | mRNA | 0.058269 | TCN1 |
| Model-90 | 33968130 | Tu Z | 2021 | mRNA | 0.223144 | ASPM |
| Model-90 | 33968130 | Tu Z | 2021 | mRNA | -0.05129 | PCP4 |
| Model-90 | 33968130 | Tu Z | 2021 | mRNA | 0.14842 | CAV2 |
| Model-90 | 33968130 | Tu Z | 2021 | mRNA | 0.058269 | S100P |
| Model-90 | 33968130 | Tu Z | 2021 | mRNA | 0.086178 | COL1A1 |
| Model-90 | 33968130 | Tu Z | 2021 | mRNA | -0.13926 | SPOCK2 |
| Model-91 | 35464867 | Hou S | 2022 | mRNA | 0.1574 | ANLN |
| Model-91 | 35464867 | Hou S | 2022 | mRNA | 0.0163 | ASPM |
| Model-91 | 35464867 | Hou S | 2022 | mRNA | -0.1315 | CDCA8 |
| Model-91 | 35464867 | Hou S | 2022 | mRNA | 0.0241 | CENPE |
| Model-91 | 35464867 | Hou S | 2022 | mRNA | 0.0598 | KIF14 |
| Model-91 | 35464867 | Hou S | 2022 | mRNA | -0.0431 | MCM2 |
| Model-91 | 35464867 | Hou S | 2022 | mRNA | 0.0907 | PLK1 |
| Model-91 | 35464867 | Hou S | 2022 | mRNA | -0.0916 | RACGAP1 |
| Model-91 | 35464867 | Hou S | 2022 | mRNA | -0.0478 | STIL |
| Model-92 | 34259140 | Wang Y | 2022 | mRNA | 0.010221 | CPS1 |
| Model-92 | 34259140 | Wang Y | 2022 | mRNA | 0.072698 | RHOV |
| Model-92 | 34259140 | Wang Y | 2022 | mRNA | 0.009635 | TNNT1 |
| Model-92 | 34259140 | Wang Y | 2022 | mRNA | 0.053753 | FAM83A |
| Model-92 | 34259140 | Wang Y | 2022 | mRNA | 0.044193 | IGF2BP1 |
| Model-92 | 34259140 | Wang Y | 2022 | mRNA | -0.0223 | GRIN2A |
| Model-93 | 35154287 | Jiang A | 2022 | mRNA | 0.088 | CP |
| Model-93 | 35154287 | Jiang A | 2022 | mRNA | 0.15 | GOLM1 |
| Model-93 | 35154287 | Jiang A | 2022 | mRNA | -0.064 | CYP4B1 |
| Model-93 | 35154287 | Jiang A | 2022 | mRNA | -0.082 | DAPK2 |
| Model-93 | 35154287 | Jiang A | 2022 | mRNA | -0.059 | NFIX |
| Model-93 | 35154287 | Jiang A | 2022 | mRNA | 0.086 | FHL2 |
| Model-94 | 36353226 | Chen Y | 2022 | mRNA | 0.275171 | ENTPD2 |
| Model-94 | 36353226 | Chen Y | 2022 | mRNA | -0.22807 | KHDRBS2 |
| Model-94 | 36353226 | Chen Y | 2022 | mRNA | 0.176096 | BARX1 |
| Model-94 | 36353226 | Chen Y | 2022 | mRNA | -0.34099 | GFRA3 |
| Model-94 | 36353226 | Chen Y | 2022 | mRNA | -0.44594 | MYOZ1 |
| Model-95 | 31779055 | Luo C | 2020 | mRNA | 0.2518 | ANLN |
| Model-95 | 31779055 | Luo C | 2020 | mRNA | 0.0879 | F2 |
| Model-96 | 33552145 | Ren J | 2021 | mRNA | -0.26047 | SLC24A4 |
| Model-96 | 33552145 | Ren J | 2021 | mRNA | -0.17309 | ZNF677 |
| Model-96 | 33552145 | Ren J | 2021 | mRNA | -0.1065 | ASCL2 |
| Model-96 | 33552145 | Ren J | 2021 | mRNA | -0.02993 | WNT3A |
| Model-96 | 33552145 | Ren J | 2021 | mRNA | -0.01703 | NKD1 |
| Model-96 | 33552145 | Ren J | 2021 | mRNA | -0.01405 | CCDC81 |
| Model-96 | 33552145 | Ren J | 2021 | mRNA | -0.00262 | ITPKB |
| Model-96 | 33552145 | Ren J | 2021 | mRNA | 0.004106 | ITGA5 |
| Model-96 | 33552145 | Ren J | 2021 | mRNA | 0.012075 | TMEM171 |
| Model-96 | 33552145 | Ren J | 2021 | mRNA | 0.02178 | TK1 |
| Model-96 | 33552145 | Ren J | 2021 | mRNA | 0.038934 | PTPRH |
| Model-96 | 33552145 | Ren J | 2021 | mRNA | 0.040348 | ITGA2 |
| Model-96 | 33552145 | Ren J | 2021 | mRNA | 0.044243 | SLC6A17 |
| Model-96 | 33552145 | Ren J | 2021 | mRNA | 0.066877 | CLDN6 |
| Model-96 | 33552145 | Ren J | 2021 | mRNA | 0.076749 | TYMS |
| Model-96 | 33552145 | Ren J | 2021 | mRNA | 0.090804 | FKBP5 |
| Model-96 | 33552145 | Ren J | 2021 | mRNA | 0.094482 | SPDL1 |
| Model-96 | 33552145 | Ren J | 2021 | mRNA | 0.09715 | FAM83A |
| Model-96 | 33552145 | Ren J | 2021 | mRNA | 0.116564 | E2F7 |
| Model-96 | 33552145 | Ren J | 2021 | mRNA | 0.142748 | TRIM58 |
| Model-96 | 33552145 | Ren J | 2021 | mRNA | 0.175469 | NFE2L3 |
| Model-97 | 34409029 | Liu TT | 2021 | mRNA | 0.255264 | SIT1 |
| Model-97 | 34409029 | Liu TT | 2021 | mRNA | -0.40045 | SNAI3 |
| Model-97 | 34409029 | Liu TT | 2021 | mRNA | -0.79932 | ASB2 |
| Model-97 | 34409029 | Liu TT | 2021 | mRNA | 0.203711 | CDK2 |
| Model-98 | 32087603 | Li W | 2020 | mRNA | 0.1362 | IGF2BP1 |
| Model-98 | 32087603 | Li W | 2020 | mRNA | 1.6799 | IFIT1B |
| Model-98 | 32087603 | Li W | 2020 | mRNA | 0.2843 | PABPC1 |
| Model-98 | 32087603 | Li W | 2020 | mRNA | -0.2663 | TLR8 |
| Model-98 | 32087603 | Li W | 2020 | mRNA | 0.3882 | GAPDH |
| Model-98 | 32087603 | Li W | 2020 | mRNA | 0.8073 | PIWIL4 |
| Model-98 | 32087603 | Li W | 2020 | mRNA | -0.3219 | RNPC3 |
| Model-98 | 32087603 | Li W | 2020 | mRNA | 0.4965 | ZC3H12C |
| Model-99 | 33314730 | Wu C | 2021 | mRNA | 0.098 | ENTPD2 |
| Model-99 | 33314730 | Wu C | 2021 | mRNA | 0.168 | MIF |
| Model-100 | 33816503 | Zheng Y | 2021 | mRNA | -0.1773 | PLEKHB1 |
| Model-100 | 33816503 | Zheng Y | 2021 | mRNA | -0.2011 | LY75 |
| Model-100 | 33816503 | Zheng Y | 2021 | mRNA | 0.0869 | PHGR1 |
| Model-100 | 33816503 | Zheng Y | 2021 | mRNA | 0.445 | TMEM194B |
| Model-100 | 33816503 | Zheng Y | 2021 | mRNA | 0.1999 | APOL1 |
| Model-100 | 33816503 | Zheng Y | 2021 | mRNA | -0.1034 | PPP2R2B |
| Model-100 | 33816503 | Zheng Y | 2021 | mRNA | -0.1767 | CD160 |
| Model-100 | 33816503 | Zheng Y | 2021 | mRNA | -0.2573 | GPR31 |
| Model-100 | 33816503 | Zheng Y | 2021 | mRNA | -0.2125 | CLEC12B |
| Model-101 | 35571564 | Yang C | 2022 | mRNA | -0.20477 | ADRB2 |
| Model-101 | 35571564 | Yang C | 2022 | mRNA | -0.01476 | AGER |
| Model-101 | 35571564 | Yang C | 2022 | mRNA | 0.132931 | CDKN3 |
| Model-101 | 35571564 | Yang C | 2022 | mRNA | 0.080656 | GJB2 |
| Model-101 | 35571564 | Yang C | 2022 | mRNA | -0.00612 | SFTPC |
| Model-101 | 35571564 | Yang C | 2022 | mRNA | 0.071989 | SLC2A1 |
| Model-101 | 35571564 | Yang C | 2022 | mRNA | -0.10624 | SLC6A4 |
| Model-101 | 35571564 | Yang C | 2022 | mRNA | -0.21875 | SSR4 |
| Model-102 | 34805165 | Liu LP | 2021 | mRNA | 1.35299 | ZSCAN5B |
| Model-102 | 34805165 | Liu LP | 2021 | mRNA | 0.18929 | E2F7 |
| Model-102 | 34805165 | Liu LP | 2021 | mRNA | -0.50357 | OR2A7 |
| Model-102 | 34805165 | Liu LP | 2021 | mRNA | 0.21535 | GLI2 |
| Model-102 | 34805165 | Liu LP | 2021 | mRNA | -0.11685 | EIF2AK3 |
| Model-102 | 34805165 | Liu LP | 2021 | mRNA | 0.39199 | SRGAP1 |
| Model-102 | 34805165 | Liu LP | 2021 | mRNA | -0.3032 | RUBCNL |
| Model-102 | 34805165 | Liu LP | 2021 | mRNA | 0.08238 | EMC6 |
| Model-103 | 35399245 | Lin X | 2022 | mRNA | 0.13 | MKI67 |
| Model-103 | 35399245 | Lin X | 2022 | mRNA | -0.27 | BTK |
| Model-103 | 35399245 | Lin X | 2022 | mRNA | 0.23 | TUBB6 |
| Model-103 | 35399245 | Lin X | 2022 | mRNA | -0.14 | MST1 |
| Model-104 | 33386920 | Yi M | 2021 | mRNA | -0.08876 | VIPR1 |
| Model-104 | 33386920 | Yi M | 2021 | mRNA | 0.16676 | BIRC5 |
| Model-104 | 33386920 | Yi M | 2021 | mRNA | 0.12945 | GDF10 |
| Model-104 | 33386920 | Yi M | 2021 | mRNA | 0.03706 | ADRB2 |
| Model-104 | 33386920 | Yi M | 2021 | mRNA | 0.06621 | IL20RB |
| Model-104 | 33386920 | Yi M | 2021 | mRNA | 0.15962 | LGR4 |
| Model-104 | 33386920 | Yi M | 2021 | mRNA | 0.06204 | INHA |
| Model-104 | 33386920 | Yi M | 2021 | mRNA | 0.01529 | CD19 |
| Model-104 | 33386920 | Yi M | 2021 | mRNA | 0.04467 | S100P |
| Model-104 | 33386920 | Yi M | 2021 | mRNA | -0.05711 | IGKV1.8 |
| Model-104 | 33386920 | Yi M | 2021 | mRNA | -0.02639 | IGKV1D.43 |
| Model-104 | 33386920 | Yi M | 2021 | mRNA | -0.30831 | ADRB1 |
| Model-104 | 33386920 | Yi M | 2021 | mRNA | 0.01446 | HTR3A |
| Model-104 | 33386920 | Yi M | 2021 | mRNA | -0.09179 | ADM2 |
| Model-104 | 33386920 | Yi M | 2021 | mRNA | -0.19053 | TLR8 |
| Model-104 | 33386920 | Yi M | 2021 | mRNA | 0.08239 | GREM1 |
| Model-104 | 33386920 | Yi M | 2021 | mRNA | -0.10157 | IGHV3.64 |
| Model-105 | 35954390 | Jiang Z | 2022 | mRNA | 0.081 | HMMR |
| Model-105 | 35954390 | Jiang Z | 2022 | mRNA | 0.074 | PLK1 |
| Model-105 | 35954390 | Jiang Z | 2022 | mRNA | -0.011 | SFTA3 |
| Model-105 | 35954390 | Jiang Z | 2022 | mRNA | -0.011 | CLIC6 |
| Model-105 | 35954390 | Jiang Z | 2022 | mRNA | 0.043 | ABCC2 |
| Model-105 | 35954390 | Jiang Z | 2022 | mRNA | -0.019 | CXCL17 |
| Model-106 | 35720127 | Wang G | 2022 | mRNA | 0.0113 | CHST4 |
| Model-106 | 35720127 | Wang G | 2022 | mRNA | -0.0817 | GLP1R |
| Model-107 | 35785155 | Li H | 2022 | mRNA | -0.46605 | CASC3 |
| Model-107 | 35785155 | Li H | 2022 | mRNA | -0.64556 | USP4 |
| Model-107 | 35785155 | Li H | 2022 | mRNA | 0.11549 | CTCFL |
| Model-107 | 35785155 | Li H | 2022 | mRNA | -0.34872 | SETDB2 |
| Model-107 | 35785155 | Li H | 2022 | mRNA | 0.09105 | 44989 |
| Model-107 | 35785155 | Li H | 2022 | mRNA | 0.16502 | KIRREL3 |
| Model-107 | 35785155 | Li H | 2022 | mRNA | 0.12956 | GRIK2 |
| Model-107 | 35785155 | Li H | 2022 | mRNA | -0.6074 | EIF2AK3 |
| Model-107 | 35785155 | Li H | 2022 | mRNA | -0.15933 | SNTG2 |
| Model-107 | 35785155 | Li H | 2022 | mRNA | 0.0645 | LINGO2 |
| Model-107 | 35785155 | Li H | 2022 | mRNA | -0.24452 | ZNF708 |
| Model-108 | 36245988 | Liu C | 2022 | mRNA | -0.1733 | VGLL3 |
| Model-108 | 36245988 | Liu C | 2022 | mRNA | -0.13625 | SNX30 |
| Model-108 | 36245988 | Liu C | 2022 | mRNA | -0.10397 | WFDC2 |
| Model-108 | 36245988 | Liu C | 2022 | mRNA | -0.06852 | STK32A |
| Model-108 | 36245988 | Liu C | 2022 | mRNA | -0.05899 | PHLDB2 |
| Model-108 | 36245988 | Liu C | 2022 | mRNA | -0.03667 | KLK11 |
| Model-108 | 36245988 | Liu C | 2022 | mRNA | -0.02289 | SMAD9 |
| Model-108 | 36245988 | Liu C | 2022 | mRNA | 0.019922 | LAMA3 |
| Model-108 | 36245988 | Liu C | 2022 | mRNA | 0.034212 | FSTL3 |
| Model-108 | 36245988 | Liu C | 2022 | mRNA | 0.037058 | ANGPTL4 |
| Model-108 | 36245988 | Liu C | 2022 | mRNA | 0.083741 | TNS4 |
| Model-108 | 36245988 | Liu C | 2022 | mRNA | 0.154147 | ITGA2 |
| Model-108 | 36245988 | Liu C | 2022 | mRNA | 0.333951 | KCNQ3 |
| Model-108 | 36245988 | Liu C | 2022 | mRNA | 0.449172 | MAPK4 |
| Model-109 | 36293001 | Tang B | 2022 | mRNA | -0.4126 | BIRC5 |
| Model-109 | 36293001 | Tang B | 2022 | mRNA | 0.39986 | PLK1 |
| Model-109 | 36293001 | Tang B | 2022 | mRNA | 0.2651 | CDKN3 |
| Model-109 | 36293001 | Tang B | 2022 | mRNA | -0.0955 | CYP4B1 |
| Model-110 | 36400857 | Li F | 2022 | mRNA | 0.101362 | IGFBP1 |
| Model-110 | 36400857 | Li F | 2022 | mRNA | 0.197865 | DDIT4 |
| Model-110 | 36400857 | Li F | 2022 | mRNA | -0.12713 | DERL3 |
| Model-110 | 36400857 | Li F | 2022 | mRNA | 0.17584 | PPP1R3G |
| Model-110 | 36400857 | Li F | 2022 | mRNA | -0.11459 | CFTR |
| Model-110 | 36400857 | Li F | 2022 | mRNA | -0.17628 | NUPR1 |
| Model-110 | 36400857 | Li F | 2022 | mRNA | 0.055218 | PDX1 |
| Model-111 | 35149175 | Shi Y | 2022 | mRNA | 0.003369 | DDIT4 |
| Model-111 | 35149175 | Shi Y | 2022 | mRNA | 0.006717 | FSCN1 |
| Model-111 | 35149175 | Shi Y | 2022 | mRNA | 0.012026 | OAS3 |
| Model-112 | 36647508 | Shi Y | 2022 | mRNA | 0.269 | FGF2 |
| Model-112 | 36647508 | Shi Y | 2022 | mRNA | 0.177 | HMMR |
| Model-112 | 36647508 | Shi Y | 2022 | mRNA | -0.076 | NR0B2 |
| Model-113 | 35480896 | Zheng P | 2022 | mRNA | -0.377 | CLEC7A |
| Model-113 | 35480896 | Zheng P | 2022 | mRNA | 0.17 | BIRC3 |
| Model-113 | 35480896 | Zheng P | 2022 | mRNA | 0.483 | SOD1 |
| Model-113 | 35480896 | Zheng P | 2022 | mRNA | 0.156 | VCAN |
| Model-113 | 35480896 | Zheng P | 2022 | mRNA | 0.132 | KYNU |
| Model-113 | 35480896 | Zheng P | 2022 | mRNA | 0.099 | CCL20 |
| Model-113 | 35480896 | Zheng P | 2022 | mRNA | 0.877 | CTAG1B |
| Model-113 | 35480896 | Zheng P | 2022 | mRNA | -0.201 | CD69 |
| Model-114 | 36042374 | Luo D | 2022 | mRNA | 0.1241 | CCL20 |
| Model-114 | 36042374 | Luo D | 2022 | mRNA | -0.6192 | CCR2 |
| Model-114 | 36042374 | Luo D | 2022 | mRNA | 0.366 | GNAI3 |
| Model-114 | 36042374 | Luo D | 2022 | mRNA | 0.124 | ITGA5 |
| Model-114 | 36042374 | Luo D | 2022 | mRNA | 0.205 | NMI |
| Model-114 | 36042374 | Luo D | 2022 | mRNA | 0.133 | PCDH7 |
| Model-114 | 36042374 | Luo D | 2022 | mRNA | 0.43 | PSEN1 |
| Model-114 | 36042374 | Luo D | 2022 | mRNA | -0.3382 | SLC11A2 |
| Model-115 | 35582412 | Jiang A | 2022 | mRNA | 0.108 | ANGPTL4 |
| Model-115 | 35582412 | Jiang A | 2022 | mRNA | 0.265 | NPAS2 |
| Model-115 | 35582412 | Jiang A | 2022 | mRNA | 0.083 | SLCO1B3 |
| Model-115 | 35582412 | Jiang A | 2022 | mRNA | -0.261 | ACOXL |
| Model-115 | 35582412 | Jiang A | 2022 | mRNA | -0.191 | ALOX15 |
| Model-115 | 35582412 | Jiang A | 2022 | mRNA | 0.177 | B3GALNT1 |
| Model-116 | 36389327 | Wang W | 2022 | mRNA | -0.08807 | KAT2B |
| Model-116 | 36389327 | Wang W | 2022 | mRNA | -0.03254 | KAT8 |
| Model-116 | 36389327 | Wang W | 2022 | mRNA | -0.0364 | HDAC2 |
| Model-116 | 36389327 | Wang W | 2022 | mRNA | -0.03734 | SIRT2 |
| Model-116 | 36389327 | Wang W | 2022 | mRNA | -0.04152 | HDAC5 |
| Model-117 | 34630529 | Liang M | 2021 | mRNA | 3.18E-05 | TXNRD1 |
| Model-117 | 34630529 | Liang M | 2021 | mRNA | 0.006636 | TRIB3 |
| Model-117 | 34630529 | Liang M | 2021 | mRNA | 0.004573 | SLC2A1 |
| Model-117 | 34630529 | Liang M | 2021 | mRNA | 0.009214 | CDKN2A |
| Model-117 | 34630529 | Liang M | 2021 | mRNA | 0.004028 | RRM2 |
| Model-117 | 34630529 | Liang M | 2021 | mRNA | 0.006819 | SLC7A11 |
| Model-117 | 34630529 | Liang M | 2021 | mRNA | 0.002893 | G6PD |
| Model-118 | 34635925 | Zhou B | 2022 | mRNA | 0.181582 | F2RL1 |
| Model-118 | 34635925 | Zhou B | 2022 | mRNA | 0.224132 | CTSL |
| Model-118 | 34635925 | Zhou B | 2022 | mRNA | 0.62023 | PPIA |
| Model-118 | 34635925 | Zhou B | 2022 | mRNA | 0.299065 | TAP2 |
| Model-118 | 34635925 | Zhou B | 2022 | mRNA | -0.23725 | HLA-DMB |
| Model-119 | 34707942 | Liu Y | 2021 | mRNA | 0.19062 | TNFRSF11A |
| Model-119 | 34707942 | Liu Y | 2021 | mRNA | 0.425268 | GALNT4 |
| Model-119 | 34707942 | Liu Y | 2021 | mRNA | 0.04879 | PCDH7 |
| Model-119 | 34707942 | Liu Y | 2021 | mRNA | -0.06188 | STK32A |
| Model-119 | 34707942 | Liu Y | 2021 | mRNA | 0.086178 | CDK5R1 |
| Model-119 | 34707942 | Liu Y | 2021 | mRNA | 0.039221 | MAFF |
| Model-119 | 34707942 | Liu Y | 2021 | mRNA | -0.03046 | ERO1B |
| Model-119 | 34707942 | Liu Y | 2021 | mRNA | 0.029559 | GNPNAT1 |
| Model-119 | 34707942 | Liu Y | 2021 | mRNA | 0.277632 | ASAH2B |
| Model-119 | 34707942 | Liu Y | 2021 | mRNA | -0.16252 | RASGEF1B |
| Model-119 | 34707942 | Liu Y | 2021 | mRNA | 0 | LDHA |
| Model-119 | 34707942 | Liu Y | 2021 | mRNA | -0.8675 | GCSAML |
| Model-120 | 34877770 | Song X | 2022 | mRNA | -0.12159 | CRHR2 |
| Model-120 | 34877770 | Song X | 2022 | mRNA | -0.04838 | BPIFB2 |
| Model-120 | 34877770 | Song X | 2022 | mRNA | 0.045865 | INHA |
| Model-120 | 34877770 | Song X | 2022 | mRNA | -0.01847 | SSTR5 |
| Model-120 | 34877770 | Song X | 2022 | mRNA | -0.01165 | SCGB3A1 |
| Model-120 | 34877770 | Song X | 2022 | mRNA | -0.00219 | BPIFB1 |
| Model-121 | 36543847 | Zhang P | 2022 | mRNA | 0.05682 | Plk1 |
| Model-121 | 36543847 | Zhang P | 2022 | mRNA | 0.00878 | HMMR |
| Model-121 | 36543847 | Zhang P | 2022 | mRNA | 0.10474 | ANLN |
| Model-121 | 36543847 | Zhang P | 2022 | mRNA | 0.01988 | SLC2A1 |
| Model-121 | 36543847 | Zhang P | 2022 | mRNA | -0.00501 | SFTPB |
| Model-121 | 36543847 | Zhang P | 2022 | mRNA | -0.00608 | CYP4B1 |
| Model-122 | 35836921 | Li W | 2022 | mRNA | 0.066 | PITX3 |
| Model-122 | 35836921 | Li W | 2022 | mRNA | 0.087 | RHOV |
| Model-122 | 35836921 | Li W | 2022 | mRNA | 0.111 | 44989 |
| Model-122 | 35836921 | Li W | 2022 | mRNA | -0.033 | ZNF536 |
| Model-122 | 35836921 | Li W | 2022 | mRNA | -0.047 | SLC14A2 |
| Model-122 | 35836921 | Li W | 2022 | mRNA | -0.079 | CYP17A1 |
| Model-122 | 35836921 | Li W | 2022 | mRNA | 0.053 | IGFBP1 |
| Model-122 | 35836921 | Li W | 2022 | mRNA | 0.044 | KRT76 |
| Model-122 | 35836921 | Li W | 2022 | mRNA | -0.072 | GFI1B |
| Model-123 | 35547263 | Gao H | 2022 | mRNA | -0.0461 | TLR8 |
| Model-123 | 35547263 | Gao H | 2022 | mRNA | 0.0992 | FGF2 |
| Model-123 | 35547263 | Gao H | 2022 | mRNA | 0.0467 | F12 |
| Model-123 | 35547263 | Gao H | 2022 | mRNA | 0.3515 | ST6GALNAC3 |
| Model-123 | 35547263 | Gao H | 2022 | mRNA | 0.0198 | PTPRH |
| Model-123 | 35547263 | Gao H | 2022 | mRNA | 0.0368 | EXO1 |
| Model-123 | 35547263 | Gao H | 2022 | mRNA | 0.0182 | FRMD3 |
| Model-123 | 35547263 | Gao H | 2022 | mRNA | 0.1891 | E2F7 |
| Model-123 | 35547263 | Gao H | 2022 | mRNA | -0.1644 | ABHD6 |
| Model-123 | 35547263 | Gao H | 2022 | mRNA | -0.0423 | STK32A |
| Model-123 | 35547263 | Gao H | 2022 | mRNA | -0.0203 | COL4A3 |
| Model-123 | 35547263 | Gao H | 2022 | mRNA | 0.0178 | PLEK2 |
| Model-123 | 35547263 | Gao H | 2022 | mRNA | -0.0222 | LIFR |
| Model-123 | 35547263 | Gao H | 2022 | mRNA | 0.04453 | CYS1 |
| Model-124 | 35837383 | Zhang X | 2022 | mRNA | 0.14 | EXO1 |
| Model-124 | 35837383 | Zhang X | 2022 | mRNA | -0.199 | CD40LG |
| Model-124 | 35837383 | Zhang X | 2022 | mRNA | 0.082 | COCH |
| Model-125 | 35251577 | Xiao L | 2022 | mRNA | 0.0387 | AHNAK2 |
| Model-125 | 35251577 | Xiao L | 2022 | mRNA | 0.1666 | CDC25C |
| Model-125 | 35251577 | Xiao L | 2022 | mRNA | 0.0029 | CPS1 |
| Model-125 | 35251577 | Xiao L | 2022 | mRNA | 0.1617 | CDX2 |
| Model-125 | 35251577 | Xiao L | 2022 | mRNA | 0.0268 | NTSR1 |
| Model-125 | 35251577 | Xiao L | 2022 | mRNA | 0.0066 | SLC2A1 |
| Model-126 | 34484331 | Chen Y | 2021 | mRNA | 0.1798 | UHRF1 |
| Model-126 | 34484331 | Chen Y | 2021 | mRNA | -0.1435 | CLSPN |
| Model-126 | 34484331 | Chen Y | 2021 | mRNA | 0.2179 | CENPE |
| Model-126 | 34484331 | Chen Y | 2021 | mRNA | -0.2046 | POLQ |
| Model-126 | 34484331 | Chen Y | 2021 | mRNA | -0.06 | MCM4 |
| Model-126 | 34484331 | Chen Y | 2021 | mRNA | 0.0325 | BRIP1 |
| Model-126 | 34484331 | Chen Y | 2021 | mRNA | -0.1421 | HELLS |
| Model-126 | 34484331 | Chen Y | 2021 | mRNA | -0.0996 | ATAD2 |
| Model-126 | 34484331 | Chen Y | 2021 | mRNA | 0.138 | ZNF367 |
| Model-126 | 34484331 | Chen Y | 2021 | mRNA | -0.2166 | ESCO2 |
| Model-126 | 34484331 | Chen Y | 2021 | mRNA | 0.1384 | TMPO |
| Model-126 | 34484331 | Chen Y | 2021 | mRNA | 0.2106 | POP1 |
| Model-126 | 34484331 | Chen Y | 2021 | mRNA | 0.1902 | NUP107 |
| Model-126 | 34484331 | Chen Y | 2021 | mRNA | 0.1177 | FXYD1 |
| Model-126 | 34484331 | Chen Y | 2021 | mRNA | -0.0408 | GGTLC1 |
| Model-126 | 34484331 | Chen Y | 2021 | mRNA | 0.0288 | HIF1A |
| Model-126 | 34484331 | Chen Y | 2021 | mRNA | -0.0186 | LDHD |
| Model-126 | 34484331 | Chen Y | 2021 | mRNA | -0.2112 | MUSTN1 |
| Model-126 | 34484331 | Chen Y | 2021 | mRNA | -0.0533 | GPD1L |
| Model-126 | 34484331 | Chen Y | 2021 | mRNA | 0.0408 | TDRD10 |
| Model-126 | 34484331 | Chen Y | 2021 | mRNA | -0.0311 | TMEM130 |
| Model-126 | 34484331 | Chen Y | 2021 | mRNA | -0.0908 | FBP1 |
| Model-126 | 34484331 | Chen Y | 2021 | mRNA | -0.0839 | ATAD3C |
| Model-126 | 34484331 | Chen Y | 2021 | mRNA | 0.1504 | IL1R2 |
| Model-127 | 32799112 | Wang Z | 2020 | mRNA | 0.139 | CCL20 |
| Model-127 | 32799112 | Wang Z | 2020 | mRNA | 0.276 | SCG2 |
| Model-127 | 32799112 | Wang Z | 2020 | mRNA | 0.096 | S100P |
| Model-127 | 32799112 | Wang Z | 2020 | mRNA | -0.327 | CAT |
| Model-128 | 33648465 | Zhai Y | 2021 | mRNA | -0.01692 | CAMP |
| Model-128 | 33648465 | Zhai Y | 2021 | mRNA | 0.12492 | CCT6A |
| Model-128 | 33648465 | Zhai Y | 2021 | mRNA | 0.027753 | CDH17 |
| Model-128 | 33648465 | Zhai Y | 2021 | mRNA | 0.063466 | EFNB2 |
| Model-128 | 33648465 | Zhai Y | 2021 | mRNA | 0.168033 | FKBP3 |
| Model-128 | 33648465 | Zhai Y | 2021 | mRNA | -0.04737 | GATA2 |
| Model-128 | 33648465 | Zhai Y | 2021 | mRNA | -0.0019 | ITIH4 |
| Model-128 | 33648465 | Zhai Y | 2021 | mRNA | -0.00976 | SMAD9 |
| Model-128 | 33648465 | Zhai Y | 2021 | mRNA | -0.05753 | P2RX1 |
| Model-128 | 33648465 | Zhai Y | 2021 | mRNA | 0.005527 | PFKP |
| Model-128 | 33648465 | Zhai Y | 2021 | mRNA | 0.000291 | PKP2 |
| Model-128 | 33648465 | Zhai Y | 2021 | mRNA | 0.165327 | PTGFRN |
| Model-128 | 33648465 | Zhai Y | 2021 | mRNA | 0.012224 | PTPRH |
| Model-128 | 33648465 | Zhai Y | 2021 | mRNA | 0.044296 | CCL20 |
| Model-128 | 33648465 | Zhai Y | 2021 | mRNA | -0.00496 | SSR4 |
| Model-129 | 32989393 | Wang H | 2020 | mRNA | 0.00326 | CRABP1 |
| Model-129 | 32989393 | Wang H | 2020 | mRNA | 0.00475 | INHA |
| Model-129 | 32989393 | Wang H | 2020 | mRNA | 0.01757 | LGR4 |
| Model-129 | 32989393 | Wang H | 2020 | mRNA | -0.17506 | VIPR1 |
| Model-130 | 35685695 | Fu B | 2022 | mRNA | 0.139 | DEPDC1B |
| Model-130 | 35685695 | Fu B | 2022 | mRNA | 0.1 | TPSB2 |
| Model-130 | 35685695 | Fu B | 2022 | mRNA | 0.088 | GJB3 |
| Model-130 | 35685695 | Fu B | 2022 | mRNA | 0.07 | RHOV |
| Model-130 | 35685695 | Fu B | 2022 | mRNA | 0.051 | CPS1 |
| Model-131 | 35958325 | Zhou Y | 2022 | mRNA | 0.144605 | COL1A1 |
| Model-131 | 35958325 | Zhou Y | 2022 | mRNA | -0.24268 | GPX3 |
| Model-131 | 35958325 | Zhou Y | 2022 | mRNA | 0.269751 | PLEK2 |
| Model-132 | 35418778 | Li Z | 2022 | mRNA | -0.09803 | CD1B |
| Model-132 | 35418778 | Li Z | 2022 | mRNA | -0.08144 | VEGFD |
| Model-132 | 35418778 | Li Z | 2022 | mRNA | -0.00023 | SCGB3A1 |
| Model-133 | 35957802 | Liu W | 2022 | mRNA | 1.32004 | HMOX1 |
| Model-133 | 35957802 | Liu W | 2022 | mRNA | -1.36259 | ARRB1 |
| Model-133 | 35957802 | Liu W | 2022 | mRNA | 1.17434 | ADM |
| Model-133 | 35957802 | Liu W | 2022 | mRNA | 1.44023 | PDIA3 |
| Model-134 | 36408131 | Li J | 2022 | mRNA | 0.06062 | FURIN |
| Model-134 | 36408131 | Li J | 2022 | mRNA | 0.02622 | KIF23 |
| Model-134 | 36408131 | Li J | 2022 | mRNA | -0.05976 | SASH3 |
| Model-134 | 36408131 | Li J | 2022 | mRNA | 0.08207 | GNPNAT1 |
| Model-134 | 36408131 | Li J | 2022 | mRNA | -0.07163 | ITGAL |
| Model-135 | 36712848 | Zhou J | 2023 | mRNA | -0.04459 | ANKRD29 |
| Model-135 | 36712848 | Zhou J | 2023 | mRNA | -0.01395 | C4BPA |
| Model-135 | 36712848 | Zhou J | 2023 | mRNA | -0.03307 | CDC7 |
| Model-135 | 36712848 | Zhou J | 2023 | mRNA | 0.040134 | CDH17 |
| Model-135 | 36712848 | Zhou J | 2023 | mRNA | 0.028746 | CDKN3 |
| Model-135 | 36712848 | Zhou J | 2023 | mRNA | -0.00731 | CLDN2 |
| Model-135 | 36712848 | Zhou J | 2023 | mRNA | 0.086427 | DKK1 |
| Model-135 | 36712848 | Zhou J | 2023 | mRNA | 0.079158 | FOSL1 |
| Model-135 | 36712848 | Zhou J | 2023 | mRNA | 0.047347 | GPR37 |
| Model-135 | 36712848 | Zhou J | 2023 | mRNA | 0.073751 | GPRIN1 |
| Model-135 | 36712848 | Zhou J | 2023 | mRNA | -0.03852 | GSTA1 |
| Model-135 | 36712848 | Zhou J | 2023 | mRNA | 0.00427 | HJURP |
| Model-135 | 36712848 | Zhou J | 2023 | mRNA | -0.00462 | HLF |
| Model-135 | 36712848 | Zhou J | 2023 | mRNA | 0.039974 | KIF20A |
| Model-135 | 36712848 | Zhou J | 2023 | mRNA | -0.00503 | KLK11 |
| Model-135 | 36712848 | Zhou J | 2023 | mRNA | 0.002399 | LPL |
| Model-135 | 36712848 | Zhou J | 2023 | mRNA | 0.122509 | PRC1 |
| Model-135 | 36712848 | Zhou J | 2023 | mRNA | -0.04249 | RFC4 |
| Model-135 | 36712848 | Zhou J | 2023 | mRNA | -0.02698 | RNASEH2A |
| Model-135 | 36712848 | Zhou J | 2023 | mRNA | -0.05902 | TCF19 |
| Model-135 | 36712848 | Zhou J | 2023 | mRNA | 0.047388 | TNNT1 |
| Model-135 | 36712848 | Zhou J | 2023 | mRNA | 0.059587 | UBE2S |
| Model-136 | 36467089 | Cheng T | 2022 | mRNA | 0.231137 | PIR |
| Model-136 | 36467089 | Cheng T | 2022 | mRNA | -0.54955 | PEBP1 |
| Model-136 | 36467089 | Cheng T | 2022 | mRNA | 0.237483 | PPP1R13L |
| Model-136 | 36467089 | Cheng T | 2022 | mRNA | 0.073528 | CA9 |
| Model-136 | 36467089 | Cheng T | 2022 | mRNA | -0.50853 | GLS2 |
| Model-136 | 36467089 | Cheng T | 2022 | mRNA | 0.331639 | DECR1 |
| Model-136 | 36467089 | Cheng T | 2022 | mRNA | 0.340344 | OTUB1 |
| Model-136 | 36467089 | Cheng T | 2022 | mRNA | 0.38634 | YWHAE |
| Model-137 | 35991543 | Lan X | 2022 | mRNA | 0.319907 | KPNA4 |
| Model-137 | 35991543 | Lan X | 2022 | mRNA | -0.32021 | KPNA5 |
| Model-138 | 36519025 | Zou Y | 2022 | mRNA | 0.223537 | DKK1 |
| Model-138 | 36519025 | Zou Y | 2022 | mRNA | -0.23874 | TSPAN7 |
| Model-138 | 36519025 | Zou Y | 2022 | mRNA | 0.215841 | ID1 |
| Model-138 | 36519025 | Zou Y | 2022 | mRNA | 0.182602 | DLGAP5 |
| Model-138 | 36519025 | Zou Y | 2022 | mRNA | 0.116091 | HHIPL2 |
| Model-138 | 36519025 | Zou Y | 2022 | mRNA | -0.36646 | CD40 |
| Model-138 | 36519025 | Zou Y | 2022 | mRNA | 0.180364 | SEMA3C |
| Model-139 | 34737767 | Zhang C | 2021 | mRNA | 0.086178 | GRIK2 |
| Model-139 | 34737767 | Zhang C | 2021 | mRNA | -0.06188 | CYP4B1 |
| Model-139 | 34737767 | Zhang C | 2021 | mRNA | -0.11653 | SLC24A4 |
| Model-139 | 34737767 | Zhang C | 2021 | mRNA | 0.04879 | ABCC2 |
| Model-139 | 34737767 | Zhang C | 2021 | mRNA | 0.239017 | GAPDH |
| Model-140 | 34745947 | Shi J | 2021 | mRNA | 0.0822 | KRT8 |
| Model-140 | 34745947 | Shi J | 2021 | mRNA | 0.0563 | ADM |
| Model-140 | 34745947 | Shi J | 2021 | mRNA | 0.0099 | ECT2 |
| Model-140 | 34745947 | Shi J | 2021 | mRNA | 0.0869 | CCNA2 |
| Model-140 | 34745947 | Shi J | 2021 | mRNA | 0.0418 | TYMS |
| Model-140 | 34745947 | Shi J | 2021 | mRNA | 0.0951 | FSTL3 |
| Model-140 | 34745947 | Shi J | 2021 | mRNA | 0.0374 | GOLM1 |
| Model-140 | 34745947 | Shi J | 2021 | mRNA | 0.0083 | EGLN3 |
| Model-140 | 34745947 | Shi J | 2021 | mRNA | 0.1054 | LGR4 |
| Model-140 | 34745947 | Shi J | 2021 | mRNA | -0.1263 | HGF |
| Model-140 | 34745947 | Shi J | 2021 | mRNA | -0.006 | KL |
| Model-140 | 34745947 | Shi J | 2021 | mRNA | 0.0367 | S100P |
| Model-140 | 34745947 | Shi J | 2021 | mRNA | -0.013 | AGER |
| Model-140 | 34745947 | Shi J | 2021 | mRNA | 0.0513 | NT5E |
| Model-140 | 34745947 | Shi J | 2021 | mRNA | -0.0113 | NDRG2 |
| Model-140 | 34745947 | Shi J | 2021 | mRNA | -0.0022 | GPC3 |
| Model-140 | 34745947 | Shi J | 2021 | mRNA | 0.0034 | CA9 |
| Model-140 | 34745947 | Shi J | 2021 | mRNA | 0.0975 | TWIST2 |
| Model-140 | 34745947 | Shi J | 2021 | mRNA | -0.0198 | FHL1 |
| Model-140 | 34745947 | Shi J | 2021 | mRNA | 0.0829 | PTX3 |
| Model-141 | 35795208 | Zhu K | 2022 | mRNA | 0.1072 | FOSL1 |
| Model-141 | 35795208 | Zhu K | 2022 | mRNA | 0.09327 | KRT6A |
| Model-141 | 35795208 | Zhu K | 2022 | mRNA | -0.1144 | GPR133 |
| Model-141 | 35795208 | Zhu K | 2022 | mRNA | 0.04062 | TMPRSS2 |
| Model-141 | 35795208 | Zhu K | 2022 | mRNA | -0.1238 | PRDM16 |
| Model-141 | 35795208 | Zhu K | 2022 | mRNA | -0.02503 | SFTPB |
| Model-141 | 35795208 | Zhu K | 2022 | mRNA | -0.04079 | SFTA3 |
| Model-142 | 31803536 | Yue C | 2019 | mRNA | 0.3619 | ADAM12 |
| Model-142 | 31803536 | Yue C | 2019 | mRNA | -0.4048 | BTK |
| Model-142 | 31803536 | Yue C | 2019 | mRNA | 0.2396 | ERG |
| Model-143 | 34517527 | Niu W | 2021 | mRNA | -9.322 | AMPD1 |
| Model-143 | 34517527 | Niu W | 2021 | mRNA | -0.169 | CLECL1 |
| Model-143 | 34517527 | Niu W | 2021 | mRNA | -0.176 | DPT |
| Model-143 | 34517527 | Niu W | 2021 | mRNA | -0.029 | DUSP26 |
| Model-143 | 34517527 | Niu W | 2021 | mRNA | -0.056 | ITGAD |
| Model-143 | 34517527 | Niu W | 2021 | mRNA | -0.063 | NELL2 |
| Model-143 | 34517527 | Niu W | 2021 | mRNA | 0.077 | SYT13 |
| Model-144 | 33744872 | Li W | 2021 | mRNA | 0.1362 | IGF2BP1 |
| Model-144 | 33744872 | Li W | 2021 | mRNA | 1.6799 | IFIT1B |
| Model-144 | 33744872 | Li W | 2021 | mRNA | 0.2843 | PABPC1 |
| Model-144 | 33744872 | Li W | 2021 | mRNA | -0.2633 | TLR8 |
| Model-144 | 33744872 | Li W | 2021 | mRNA | 0.3882 | GAPDH1 |
| Model-144 | 33744872 | Li W | 2021 | mRNA | 0.8073 | PIWIL4 |
| Model-144 | 33744872 | Li W | 2021 | mRNA | -0.3219 | RNPC3 |
| Model-144 | 33744872 | Li W | 2021 | mRNA | -0.4965 | ZC3H12C |
| Model-145 | 36505456 | Huang J | 2022 | mRNA | 0.177738 | UBE2C |
| Model-145 | 36505456 | Huang J | 2022 | mRNA | 0.110354 | KRT6A |
| Model-145 | 36505456 | Huang J | 2022 | mRNA | -0.11257 | IRX2 |
| Model-145 | 36505456 | Huang J | 2022 | mRNA | -0.25013 | CD3D |
| Model-146 | 35238419 | Nie GJ | 2022 | mRNA | 0.476234 | EXO1 |
| Model-146 | 35238419 | Nie GJ | 2022 | mRNA | -0.38126 | DLC1 |
| Model-146 | 35238419 | Nie GJ | 2022 | mRNA | 2.923162 | TPTE |
| Model-146 | 35238419 | Nie GJ | 2022 | mRNA | -0.67924 | KAT2B |
| Model-146 | 35238419 | Nie GJ | 2022 | mRNA | 0.86289 | BMS1 |
| Model-146 | 35238419 | Nie GJ | 2022 | mRNA | -0.42618 | LAMA2 |
| Model-147 | 36291937 | Zhang B | 2022 | mRNA | 0.53357 | TMEM44 |
| Model-147 | 36291937 | Zhang B | 2022 | mRNA | 0.38098 | ZNF146 |
| Model-147 | 36291937 | Zhang B | 2022 | mRNA | 0.48044 | FAF2 |
| Model-147 | 36291937 | Zhang B | 2022 | mRNA | 0.37444 | PANX1 |
| Model-147 | 36291937 | Zhang B | 2022 | mRNA | 0.33212 | MLKL |
| Model-147 | 36291937 | Zhang B | 2022 | mRNA | 0.38542 | PPIA |
| Model-147 | 36291937 | Zhang B | 2022 | mRNA | 0.43709 | PMAIP1 |
| Model-147 | 36291937 | Zhang B | 2022 | mRNA | 0.23428 | TRAF2 |
| Model-147 | 36291937 | Zhang B | 2022 | mRNA | -0.30466 | PLCG1 |
| Model-148 | 36003401 | Jiao M | 2022 | mRNA | -0.00037 | SLC7A7 |
| Model-148 | 36003401 | Jiao M | 2022 | mRNA | 0.004761 | KIR2DL4 |
| Model-149 | 34111567 | Lu A | 2021 | mRNA | -0.1163 | GJB2 |
| Model-149 | 34111567 | Lu A | 2021 | mRNA | 0.1327 | SCNN1B |
| Model-150 | 36051357 | Wang N | 2022 | mRNA | -0.033 | METTL3 |
| Model-150 | 36051357 | Wang N | 2022 | mRNA | 0.0028 | KIAA1429 |
| Model-150 | 36051357 | Wang N | 2022 | mRNA | 0.0087 | HNRNPC |
| Model-150 | 36051357 | Wang N | 2022 | mRNA | -0.0053 | YTHDF1 |
| Model-150 | 36051357 | Wang N | 2022 | mRNA | -0.0101 | YTHDF2 |
| Model-150 | 36051357 | Wang N | 2022 | mRNA | 0.0413 | IGF2BP1 |
| Model-150 | 36051357 | Wang N | 2022 | mRNA | 0.001 | IGF2BP2 |
| Model-150 | 36051357 | Wang N | 2022 | mRNA | 0.0033 | IGFBP3 |
| Model-150 | 36051357 | Wang N | 2022 | mRNA | -0.0165 | FMR1 |
| Model-150 | 36051357 | Wang N | 2022 | mRNA | -0.0056 | LRPPRC |
| Model-150 | 36051357 | Wang N | 2022 | mRNA | 0.0039 | HNRNPA2B1 |
| Model-151 | 36389112 | Yang Y | 2022 | mRNA | 0.038773 | SNRPB |
| Model-151 | 36389112 | Yang Y | 2022 | mRNA | 0.222163 | SNRPD2 |
| Model-151 | 36389112 | Yang Y | 2022 | mRNA | 0.316667 | SNRPE |
| Model-151 | 36389112 | Yang Y | 2022 | mRNA | 0.001024 | SNRPF |
| Model-151 | 36389112 | Yang Y | 2022 | mRNA | -0.0274 | SNRPG |
| Model-151 | 36389112 | Yang Y | 2022 | mRNA | -0.10456 | SNRPN |
| Model-151 | 36389112 | Yang Y | 2022 | mRNA | -0.33238 | LSM2 |
| Model-151 | 36389112 | Yang Y | 2022 | mRNA | 0.085957 | LSM4 |
| Model-151 | 36389112 | Yang Y | 2022 | mRNA | 0.170066 | LSM5 |
| Model-151 | 36389112 | Yang Y | 2022 | mRNA | -0.1028 | LSM7 |
| Model-151 | 36389112 | Yang Y | 2022 | mRNA | -0.08966 | LSM8 |
| Model-152 | 35711936 | Zeng Z | 2022 | mRNA | 0.263 | COL4A6 |
| Model-152 | 35711936 | Zeng Z | 2022 | mRNA | 0.0232 | FSCN1 |
| Model-152 | 35711936 | Zeng Z | 2022 | mRNA | 0.0037 | FGA |
| Model-153 | 36798829 | Peng H | 2023 | mRNA | 1.0002 | CYCS |
| Model-153 | 36798829 | Peng H | 2023 | mRNA | -0.9272 | CAT |
| Model-153 | 36798829 | Peng H | 2023 | mRNA | 1.7096 | MRPL44 |
| Model-154 | 35924143 | Luo Y | 2022 | mRNA | -0.0482 | SFTPB |
| Model-154 | 35924143 | Luo Y | 2022 | mRNA | -0.08633 | WFDC2 |
| Model-154 | 35924143 | Luo Y | 2022 | mRNA | 0.267656 | TIMP1 |
| Model-154 | 35924143 | Luo Y | 2022 | mRNA | 0.255672 | MS4A7 |
| Model-154 | 35924143 | Luo Y | 2022 | mRNA | 0.121445 | VCAN |
| Model-154 | 35924143 | Luo Y | 2022 | mRNA | 0.260152 | KRT8 |
| Model-154 | 35924143 | Luo Y | 2022 | mRNA | 0.225274 | FABP5 |
