# Supplementary material for: Development of an immune-related gene signature applying Ridge method for improving immunotherapy responses and clinical outcomes in lung adenocarcinoma
Source: PeerJ. 2025 May 8;13:e19121. doi: 10.7717/peerj.19121 (PMC12066106; doi:10.7717/peerj.19121)
Supplement: Supplemental Information 9 [file peerj-13-19121-s009.docx]

**Supplementary material description**

**Figure S1. A.** The distribution of 28 immune cell subsets infiltration between two clusters. **B.** The distribution of immune score inferred by ESTIMATE algorithm between two clusters in the TCGA-LUAD cohort. **C.** The distribution of 22 immune cell subsets infiltration by CIBERSORT algorithm between two clusters in the TCGA-LUAD cohort. **D.** The distribution of 6 immune cell subsets infiltration by TIMER algorithm between two clusters in the TCGA-LUAD cohort. D. The distribution of 7 immune cell subsets infiltration by EPIC algorithm between two clusters in the TCGA-LUAD cohort. **E.** The distribution of 10 immune cell subsets infiltration by MCP-counter algorithm between two clusters in the TCGA-LUAD cohort. Here, * denotes p<0.05, ** denotes p<0.01, *** denotes p<0.001 and NS denotes p>0.05.

**FigureS2. A:** Analysis of the scale-free fit index for various soft-thresholding powers (β). **B.** Analysis of the mean connectivity for various soft-thresholding powers. **C.** Heatmap of all differentially expressed genes clustered based on a dissimilarity measure (1-TOM). **D.** Distribution of the number of genes in each module. **E.** Risk status of the 26 genes in different cohorts.

**Figure S3.** **A-F.** Forest plot of prognostic characteristics from multifactorial survival analyses to compare AJCC Stage, Smoker, Age, Gender, and IMMPS in different cohorts.

**Figure S4.** 154 gene signatures in five datasets with c-index sorted above or below the IMMPS statistics. Green indicates that the c-index of the signature in the corresponding dataset is lower than the IMMPS. Pink color indicates that the c-index of the signature is above than IMMPS in the corresponding dataset. The five data cohorts are represented horizontally and the corresponding 154 signatures are represented vertically.

**Figure S5. A**. The distribution of immune score inferred by ESTIMATE algorithm between two clusters in the TCGA-LUAD cohort. **B**. The distribution of 22 immune cell subsets infiltration by CIBERSORT algorithm between two clusters in the TCGA-LUAD cohort. **C**. The distribution of 6 immune cell subsets infiltration by TIMER algorithm between two clusters in the TCGA-LUAD cohort. **D**. The distribution of 7 immune cell subsets infiltration by EPIC algorithm between two clusters in the TCGA-LUAD cohort. **E**. The distribution of 10 immune cell subsets infiltration by MCP-counter algorithm between two clusters in the TCGA-LUAD cohort. *denotes p<0.05, ** denotes p<0.01, *** denotes p<0.001,NS denotes p>0.05.

**Table S1.** Summary of immune checkpoint genes collections.

**Table S2** Data on a total of 154 previous gene signatures
