# Supplementary material for: Development of an immune-related gene signature applying Ridge method for improving immunotherapy responses and clinical outcomes in lung adenocarcinoma
Source: PeerJ. 2025 May 8;13:e19121. doi: 10.7717/peerj.19121 (PMC12066106; doi:10.7717/peerj.19121)
Supplement: Supplemental Information 5 — Green indicates that the c-index of the signature in the corresponding dataset is lower than the IMMPS. Pink color indicates that the c-index of the signature is above than IMMPS in the corresponding dataset. The five data cohorts are represented horizontally and the corresponding 154 signatures are represented vertically. [file peerj-13-19121-s005.pdf]

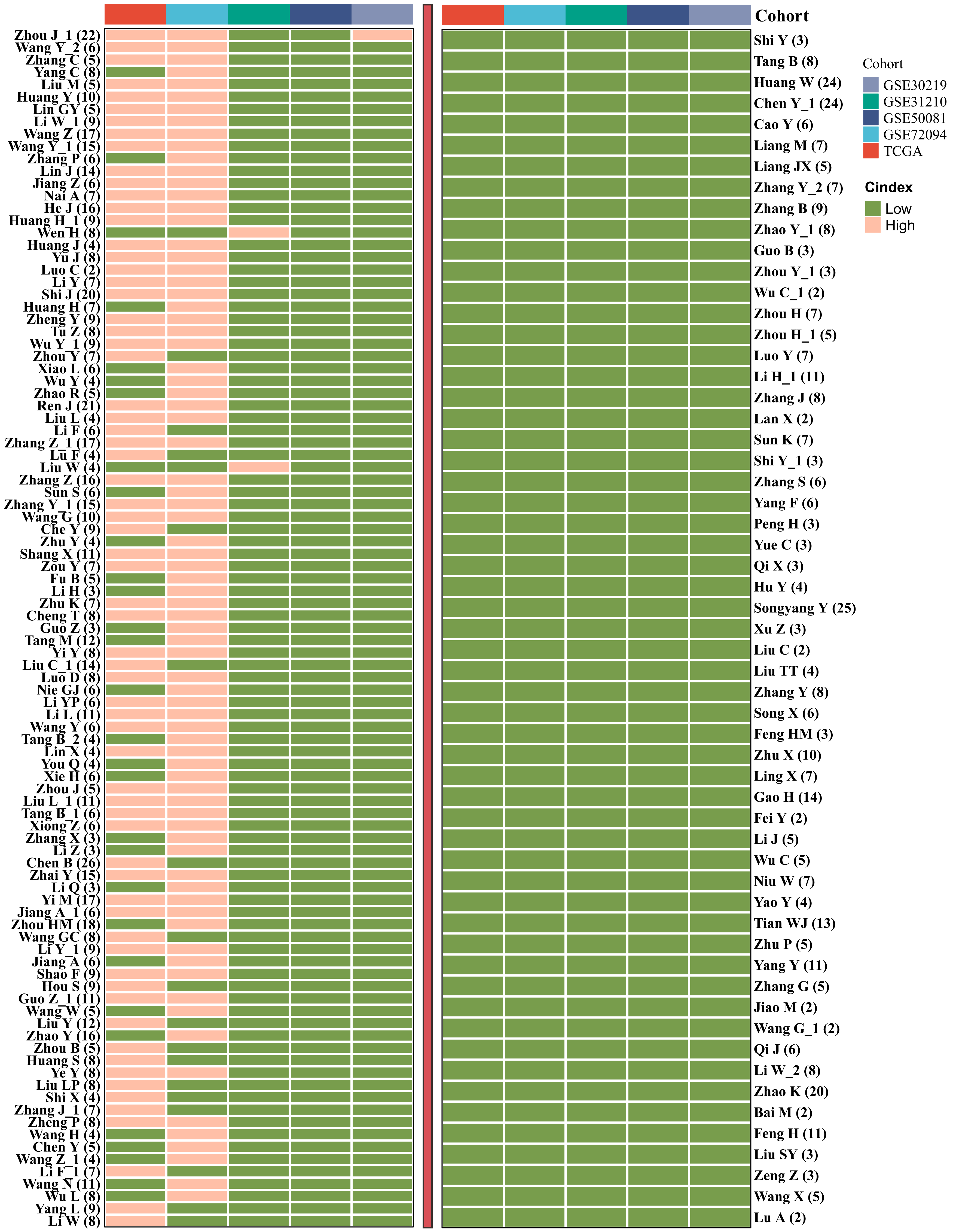
Cohort
Zhou J_1 (22)
Wang Y_2 (6)
Zhang C (5)
Yang C (8)
Liu M (5)
Huang Y (10)
Lin GY (5)
Li W_1 (9)
Wang Z (17)
Wang Y_1 (15)
Zhang P (6)
Lin J (14)
Jiang Z (6)
Nai A (7)
He J (16)
Huang H_1 (9)
Wen H (8)
Huang J (4)
Yu J (8)
Luo C (2)
Li Y (7)
Shi J (20)
Huang H (7)
Zheng Y (9)
Tu Z (8)
Wu Y_1 (9)
Zhou Y (7)
Xiao L (6)
Wu Y (4)
Zhao R (5)
Ren J (21)
Liu L (4)
Li F (6)
Zhang Z_1 (17)
Lu F (4)
Liu W (4)
Zhang Z (16)
Sun S (6)
Zhang Y_1 (15)
Wang G (10)
Che Y (9)
Zhu Y (4)
Shang X (11)
Zou Y (7)
Fu B (5)
Li H (3)
Zhu K (7)
Cheng T (8)
Guo Z (3)
Tang M (12)
Yi Y (8)
Liu C_1 (14)
Luo D (8)
Nie GJ (6)
Li YP (6)
Li L (11)
Wang Y (6)
Tang B_2 (4)
Lin X (4)
You Q (4)
Xie H (6)
Zhou J (5)
Liu L_1 (11)
Tang B_1 (6)
Xiong Z (6)
Zhang X (3)
Li Z (3)
Chen B (26)
Zhai Y (15)
Li Q (3)
Yi M (17)
Jiang A_1 (6)
Zhou HM (18)
Wang GC (8)
Li Y_1 (9)
Jiang A (6)
Shao F (9)
Hou S (9)
Guo Z_1 (11)
Wang W (5)
Liu Y (12)
Zhao Y (16)
Zhou B (5)
Huang S (8)
Ye Y (8)
Liu LP (8)
Shi X (4)
Zhang J_1 (7)
Zheng P (8)
Wang H (4)
Chen Y (5)
Wang Z_1 (4)
Li F_1 (7)
Wang N (11)
Wu L (8)
Yang L (9)
Li W (8)
Shi Y (3)
Tang B (8)
Huang W (24)
Chen Y_1 (24)
Cao Y (6)
Liang M (7)
Liang JX (5)
Zhang Y_2 (7)
Zhang B (9)
Zhao Y_1 (8)
Guo B (3)
Zhou Y_1 (3)
Wu C_1 (2)
Zhou H (7)
Zhou H_1 (5)
Luo Y (7)
Li H_1 (11)
Zhang J (8)
Lan X (2)
Sun K (7)
Shi Y_1 (3)
Zhang S (6)
Yang F (6)
Peng H (3)
Yue C (3)
Qi X (3)
Hu Y (4)
Songyang Y (25)
Xu Z (3)
Liu C (2)
Liu TT (4)
Zhang Y (8)
Song X (6)
Feng HM (3)
Zhu X (10)
Ling X (7)
Gao H (14)
Fei Y (2)
Li J (5)
Wu C (5)
Niu W (7)
Yao Y (4)
Tian WJ (13)
Zhu P (5)
Yang Y (11)
Zhang G (5)
Jiao M (2)
Wang G_1 (2)
Qi J (6)
Li W_2 (8)
Zhao K (20)
Bai M (2)
Feng H (11)
Liu SY (3)
Zeng Z (3)
Wang X (5)
Lu A (2)
Cohort
GSE30219
GSE31210
GSE50081
GSE72094
TCGA
Cindex
Low
High
